# Computational Study of the Peroxyl Radical Scavenging Ability of Phenolic Antioxidants

**DOI:** 10.3390/antiox15070868

**Published:** 2026-07-11

**Authors:** Ainsley Barisoff, Max Walton-Raaby, Paula Jofily, Nelaine Mora-Diez

**Affiliations:** 1 Department of Chemistry, Thompson Rivers University, Kamloops, BC V2C 0C8, Canada; ainsley.barisoff@gmail.com; 2 Department of Chemistry, University of Waterloo, Waterloo, ON N2L 3G1, Canada; max.walton-raaby@uwaterloo.ca (M.W.-R.); paula.jofily@uwaterloo.ca (P.J.)

**Keywords:** DFT, M06-2X, SMD, antioxidants, phenols, hydroperoxyl radical, methylperoxyl radical, rate constants, HAT, SET, thermodynamics, kinetics

## Abstract

The physiological implications of chronic oxidative stress motivate research on antioxidant activity: the cellular defence and repair mechanism for oxidative damage. Through evaluation of thermodynamic and kinetic quantities of hydroperoxyl (^•^OOH) and methylperoxyl (^•^OOCH_3_) radical scavenging reactions, the primary antioxidant activity of twenty phenolic compounds, previously investigated for tertiary antioxidant activity, is evaluated at the M06-2X(SMD)/6-31++G(d,p) level of theory in water and pentyl ethanoate. The formal-hydrogen atom transfer (f-HAT) and single electron transfer (SET) mechanisms are considered. In aqueous environments, SET proves to be the dominant mechanism for most phenols studied, as nearly half of the phenols produced rate constants within the diffusion limit, implicating biochemical relevance. Only three of the phenols also exhibited significant activity via f-HAT. The phenols show greater SET scavenging of ^•^OOH than ^•^OOCH_3_, but the more efficient f-HAT target is variable. Through comparison with their tertiary activity, we show that thermodynamics are a better predictor for antioxidant activity when the radical is a less complex species (i.e., ^•^OOH versus protein radical); however, several inconsistencies in the Bell–Evans–Polanyi principle still appear between and within solvents and phenolic compounds. This work examines the differences that appear when studying primary versus tertiary antioxidant activity and highlights the importance of using kinetic strategies to investigate antioxidant activity.

## 1. Introduction

Free radicals are labile species in biological systems which exist in a tightly regulated balance between physiological functions and harmful oxidative effects. Reactive oxygen species (ROS) act in crucial processes such as cell signalling and apotosis, immune protection, and gene expression [1,2]. High concentrations of ROS enable the oxidation of biomolecules, leading to adverse effects on cellular function [3]. Oxidative stress is the term used to describe the state in which oxidants exist in excess relative to antioxidants [2]. Accordingly, oxidative stress is associated with an increased risk of chronic health conditions and diseases such as cardiovascular disease, cancer, and neurodegenerative disorders [2,3,4]. Furthermore, the free radical theory of ageing links accumulative oxidative damage to levels of cell senescence, which in turn determines lifespan [5]. ROS can be obtained exogenously from environmental inputs (e.g., pollution, alcohol, radiation) or endogenously through cellular processes [2]. The physiological implication of oxidative stress invigorates a breadth of research regarding antioxidant activity.

The biochemical mechanism of defence against oxidative stress is antioxidant action. Antioxidants can be enzymatic (e.g., SOD, catalase, glutathione peroxidase), or non-enzymatic [2]. Some biologically significant non-enzymatic antioxidants include ascorbic acid (vitamin C), tocopherols (vitamin E), and glutathione (GSH) [1,2,3]. Antioxidant action can occur through the scavenging of free radicals (primary activity), the prevention of free radical formation (secondary activity), and by the repair of oxidatively damaged biomolecules (tertiary activity) [6].

An array of experimental assays evaluates in vitro antioxidant capacity based on a sample’s ability to neutralize reactive radical species by reaction mechanisms like single electron transfer (SET) or formal-hydrogen atom transfer (f-HAT) [7,8]. However, no single method can fully capture antioxidant behaviour, as measured activity varies between assays based on experimental conditions, limiting inter-method and therefore inter-mechanism comparisons [7]. Establishing a standardized antioxidant activity assay is a challenge that motivates the use of computational methods.

Galano et al. detailed and validated a computational methodology used to evaluate the feasibility of radical-molecule reactions based on thermodynamics and kinetics [8]. Common metrics for the investigation of antioxidant capacity include calculation of ionization potential (IP) and bond dissociation energy (BDE), which reflect SET and f-HAT reaction mechanisms, respectively. Further calculation of rate constants provides a kinetic approach that accounts for factors such as pH, solvent polarity, reaction site, quantum tunnelling, and transition state stability, while establishing a consistent basis for comparing reaction mechanisms [8]. Determining the radical scavenging ability of individual compounds through a standardized framework is valuable for identifying antioxidants with potential pharmacological applications [8]. This methodology has been applied to the study of antioxidant activity in many contexts [9,10,11,12,13,14].

A series of studies evaluated the tertiary antioxidant activity of several species by calculating thermodynamic and kinetic quantities associated with the repair of an oxidatively damaged leucine protein model (N-formyl-leucinamide) at the same level of theory [9,10,11]. By calculating rate constants for its repair at carbon-radical sites (α, β, γ, and δ) in hydrophobic and hydrophilic solvents (pentyl ethanoate and water, respectively), the physiological relevance of dihydrolipoic acid (DHLA) [9], glutathione (GSH) and Trolox [10] was evaluated. The physiological relevance of the reactions was determined according to whether the rate constants were within the diffusion limit of the solvent (1 × 10^8^ M^−1^ s^−1^), indicative that the rate-limiting step is the speed at which the solutes can diffuse through solvent and react. Diffusion-limited or near-diffusion-limited rate constants indicate that the reactions are kinetically viable under the modelled conditions; however, their biochemical relevance will depend on local concentrations, speciation, and competition with other radical-consuming pathways. DHLA and GSH exhibited f-HAT rate constants within the diffusion limit, whereas Trolox did not.

Through similar investigation, but for twenty phenolic compounds, Walton-Raaby et al. [11] found that all f-HAT reactions were exergonic, but only repair by one of the compounds yielded a rate constant on the level of DHLA and GSH. Furthermore, the considered SET reactions yielded negligible rate constants, indicating SET is not a viable repair mechanism in water for the phenols studied, when the repaired amino acid lacks heteroatoms [11]. In addition to establishing internal trends, they paralleled their results with those of Wright et al. [15]. Wright et al. [15] selected the phenols and determined BDE and IP values in the gas phase and used these properties to predict their relative rates of reaction with free radicals. They used BDE of the phenolic O-H bond as a predictor of antioxidant activity under the assumption that the weaker the OH bond, the faster f-HAT can occur. They studied the IP associated with the donation of an electron from the neutral phenolic species to predict its capacity to scavenge free radicals via SET. Several contradictions between gas-phase BDE and rate constants of protein repair were identified [11,15]. As Wright et al. [15] often discussed their results in context of reaction with peroxyl radicals, it would be of interest to compare how the kinetics of such reactions compare to their results.

Another key point of discussion by Walton-Raaby et al. [11] was the breakdown of the Bell–Evans–Polanyi principle, which indicates that the more exergonic a reaction, the faster it should be [16,17]. The thermodynamically predicted order of repair did not consistently match that of the kinetically determined order, further highlighting the value of kinetic studies to assess antioxidant activity. They call for insight on the primary activity of these phenols towards biochemically pertinent radicals [11].

The selection of the free radical of interest in a study of antioxidant activity is important in defining the biological context in which the compound acts as a radical scavenger. It may seem intuitive to determine primary antioxidant activity based on the scavenging of the most damaging ROS (^•^OH), but it is so reactive that its oxidation reactions are usually diffusion-controlled. Hence, all phenols investigated would show similarly large rate constants and consequently, similar levels of primary antioxidant activity [6,18]. The hydroperoxyl radical (^•^OOH) has been widely used to study the primary antioxidant activity of several compounds such as Trolox [19], stilbenes [18], estrogens [20], alkylresorcinols [21], and isoflavonoids [22]. It is the simplest of peroxyl radicals, with a long half-life in comparison to ^•^OH, allowing it to travel to more distant locations [18]. It is a known propagator of oxidative stress, especially through its role in lipid peroxidation [1,3,20,23]. During lipid peroxidation, lipid peroxyl radicals form as an intermediate (^•^OOR) [23]; hence, using a model, like the methylperoxyl radical (^•^OOCH_3_), to evaluate the chain-breaking character of a compound is of biological interest (as in [22]).

**Figure 1:**
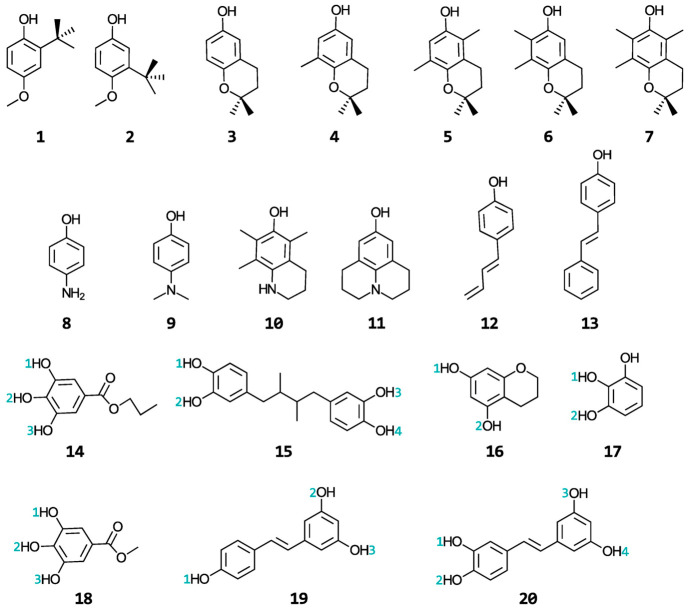
Phenolic antioxidants studied and their labels (taken and modified from [11]).

While the antioxidant activity of this set of twenty phenolic compounds has been previously quantified in terms of thermodynamic gas-phase properties [15] and tertiary antioxidant behaviour during protein repair [11], the primary activity of this group of antioxidants at the same level of theory remains unexplored. The antioxidant activity predictions made by [15] do not pertain to specific reactions. Given that antioxidants can act through multiple mechanisms and thermodynamic calculations alone struggle to predict their behaviour, the determination of activity through kinetic means in multiple reaction contexts is of value. The phenols of interest, as seen in Figure 1 and as named in Table S1, were chosen by Wright et al. [15] to represent a variety of chemical families with varying structural features and relevant antioxidant properties. Molecules **1** and **2** are butylated hydroxyanisoles (BHAs) which are synthetic compounds used as food preservatives in lipid-containing products to prevent oxidation through radical scavenging [24]. Molecules **3–7** are analogues of vitamin E (tocopherols) which are well-known biological inhibitors of lipid peroxidation, present in human blood plasma [25]. Molecules **8–11** are aminophenols, which are synthetic compounds used in dyes and pigments, photographic developers, and pharmaceuticals [26,27]. Molecules **12–13** and **19–20** are stilbenes, which are secondary metabolites of plants that have shown therapeutic potential toward conditions such as obesity, type 2 diabetes, and neurodegenerative diseases [28]. Molecules **14** and **15** are often used in conjugation with BHAs to preserve food. Molecules **16–18** are components of epigallocatechin-3-gallate (EGCG), a well-known antioxidant present in green tea that has shown promising anticancer and neuroprotective properties [29]. As the modern expectations of an antioxidant shift towards compounds which are nontoxic, versatile, and naturally derived or logically designed [30], the potential of these compounds varies. However, the purpose of understanding how certain structural features may influence biochemical reactivity remains a priority.

In this work, we aim to delineate the thermodynamics and kinetics of peroxyl radical (^•^OOH and ^•^OOCH_3_) scavenging by phenolic compounds to define and establish a relative ranking of their primary antioxidant activity in hydrophobic and hydrophilic environments during f-HAT and SET reactions. By utilizing the same methodology as in [11], the phenols can be classified according to their relative effectiveness as primary or tertiary antioxidants. Further, the relative rankings and general conclusions will be contrasted with reactivities predicted through BDE and IP calculated in [15]. Wright et al. [15] expect that all but molecules **10** and **11** are more likely to react by f-HAT than through SET, a finding which is partially corroborated by Walton-Raaby et al. [11] as all SET repair rate constants were negligible. Furthermore, the validity of the Bell–Evans–Polanyi principle will be tested through thermodynamic and kinetic comparison. Additionally, this study provides the opportunity to assess how thermodynamic gas-phase properties and overall reaction thermodynamics correlate with antioxidant activity toward radical species of varying complexity (i.e., ROS versus protein-derived radicals). For these twenty phenolic compounds, this work will broaden the understanding of their reactivity and reaction mechanisms in biological systems, contribute to a bank of kinetic antioxidant data using a consistent methodology, and contextualize and guide their application in industry or medicine.

## 2. Computational Methodology

Calculations were executed at the M06-2X(SMD)/6-31++G(d,p) level of theory at 298.15 K using the Gaussian16 package [31]. Geometry optimization and subsequent frequency calculations at the same level of theory were performed for each reactant, product, and transition state species. Transition state (TS) geometries were confirmed as such by the presence of one imaginary frequency with the accompanying animation of the expected hydrogen transfer. Reactants and products were confirmed as energy minima by the absence of imaginary frequencies.

The M06-2X functional is optimal for execution of main group thermochemistry and kinetic calculations [32]. Solvent effects were considered in all calculations by the inclusion of the SMD continuum solvation method [33] as previously recommended and used in a host of antioxidant studies [9,10,11,18,19,20,21,22]. Water and pentyl ethanoate (PE) were the solvents selected to simulate a hydrophilic and hydrophobic cellular microenvironment, respectively. These selections reflect the possibility of radical scavenging reactions to occur in aqueous environments, like the cytoplasm, or lipid environments, like cellular membranes. In a benchmark study assessing its ability to produce accurate kinetic results for radical-molecule reactions in solution, the SMD model in conjunction with M06-2X was one of the best combinations tested [34]. To maintain consistency, and therefore comparability, with the study of tertiary antioxidant activity of these twenty phenols [11], 6-31++G(d,p) was the basis set used.

The scavenging of ^•^OOH or ^•^OOCH_3_ can be achieved through f-HAT, where the hydrogen atom from the hydroxyl group of the phenol is transferred to the peroxyl radical (^•^OOR, R = H or CH_3_) resulting in a phenoxyl radical and a peroxide as shown in Reaction R1. Rate constants were calculated using conventional transition state theory (TST) via Equation (1), in which *σ* denotes reaction path degeneracy (*σ* = 1 for the f-HAT reactions studied, except for **17**^(2)^ where *σ* = 2), *κ* is the tunnelling factor calculated using the Brown’s numeric integration programme [35] assuming a one-dimensional asymmetrical Eckart barrier, *k_B_* is Boltzmann’s constant, *T* is the absolute temperature, *h* is Plank’s constants, and *R* is the ideal gas constant. Gibbs free energies of activation (ΔG^≠^) at the 1 M reference state were calculated considering corrections for solvent cage effects proposed by Okuno [36], which accounts for the free volume theory [37].

Phenol-OH + ^•^OOR ⇌ Phenol-O^•^ + HOOR (R1)

(1) $$k=\sigma \kappa \frac{k_{B}T}{h}e^{\frac{-\Delta G^{\neq }}{RT}}$$

In cases where the rate constant is greater than 10^8^ M^−1^ s^−1^, the apparent rate constant (k_app_) is calculated according to Kimball–Collins theory [38] as represented in Equation (2). To acquire k_D_, the steady-state Smoluchowski rate constant for an irreversible diffusion-controlled bimolecular reaction [39] can be calculated via Equation (3). The inputs include the distance between atoms of the hydrogen atom transfer (R), Avogadro’s number (N_A_), and the mutual diffusion coefficient of the reactants (D_AB_). D_AB_ is determined through summation of D_A_ and D_B_, which are found via the Stokes–Einstein approach [40,41] in Equation (4). The solvent viscosity (η) is 8.9 × 10^−4^ Pa s for water and 8.62 × 10^−4^ Pa s for PE, whereas α is the radius of the spherical solute A or B.

(2) $$k_{app}=\frac{k_{D}k}{k_{D}+k}$$

(3) $$k_{D}=4\pi RD_{AB}N_{A}$$

(4) $$D_{A\;or\;B}=\frac{k_{B}T}{6\pi \eta \alpha_{A\;or\;B}}$$

The SET reactions studied involve an anionic phenol reacting with the peroxyl radical to form the respective peroxyl anion (^−^OOR = ^−^OOCH_3_ or ^−^OOH), as shown in Reaction R2.

Phenol-O^−^ + ^•^OOR ⇌ Phenol-O^•^ + ^−^OOR (R2)

Calculation of rate constants for SET reactions employs Equation (1) while inserting σ and κ as equal to 1 and the SET activation barrier ($∆G_{SET}^{\neq }$ as determined through the Marcus theory [42,43] using Equation (5). Such a calculation requires the nuclear reorganization energy (λ), which represents the nonadiabatic transfer of an electron from reactants to vertical products, as written in Equation (6).

(5) $$∆G_{SET}^{\neq }=\frac{\lambda }{4}(1+\frac{∆G_{ET}^{0}}{\lambda })$$

(6) $$\lambda \approx ∆E_{ET}-∆G_{ET}^{0}$$

As the phenolic compound must be in its deprotonated state to act according to this mechanism, the molar ratio of anion at physiological pH (7.4) is multiplied by the SET rate constant to reflect that fact. Such ratios were obtained using pK_a_ values which were established in a previous publication by our group [44] and used in the study of tertiary antioxidant activity [11].

## 3. Results

### 3.1. The Formal-Hydrogen Atom Transfer (f-HAT) Reactions

The thermodynamic and kinetic quantities associated with f-HAT reactions (in water and PE) involving ^•^OOH and ^•^OOCH_3_ are presented in Table 1 and Table 2, respectively. The changes in Gibbs free energy (∆G°) provide a measure of the energetic favourability, where a more negative (exergonic) value corresponds to greater favourability of the forward reaction. Additionally, the kinetic parameters, including the Gibbs free energy of activation (∆G^≠^), rate constant (k), and total rate constants for polyphenols, are reported. Tables S4 and S5 (Tables S6 and S7) contain standard enthalpies (H°) and Gibbs free energies (G°) for the species involved in the reactions with ^•^OOH (^•^OOCH_3_) in water and PE, respectively. Tables S8 and S9 (Tables S10 and S11) report the tunnelling factors (κ) and the parameters which were necessary for its calculation during reaction with ^•^OOH (^•^OOCH_3_) in water and PE, respectively, including the imaginary frequencies corresponding to, and simultaneously confirming the identity of, transition states.

**Figure 2:**
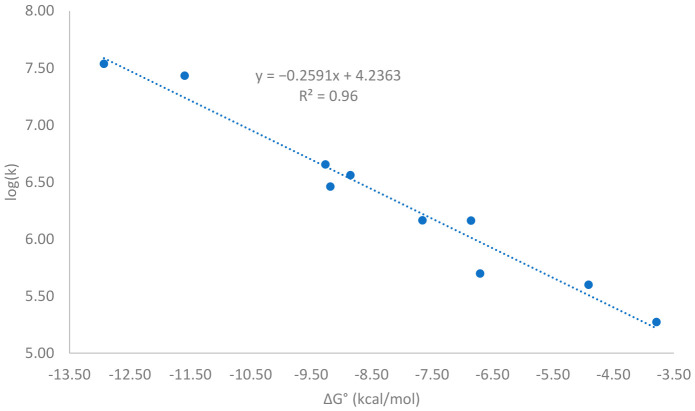
Plot of log(k) versus the ∆G° of f-HAT reactions of monophenols with ^•^OOH at the M06-2X(SMD)/6-31++G(d,p) level of theory in water.

**Figure 3:**
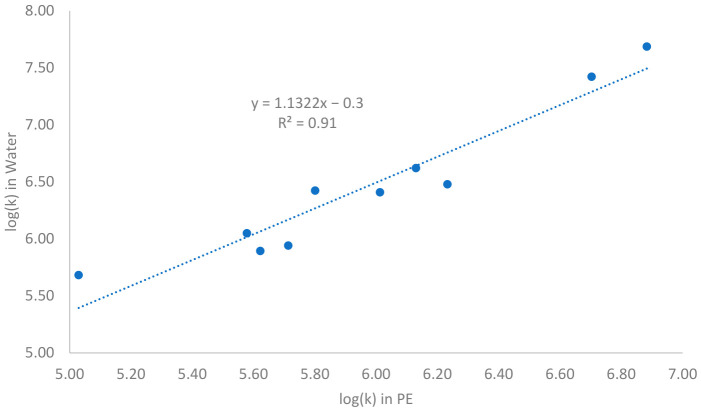
Plot of log(k) in water versus log(k) in PE for f-HAT reactions of monophenols with ^•^OOCH_3_ at the M06-2X(SMD)/6-31++G(d,p) level of theory.

### 3.2. Radical Scavenging by Single Electron Transfer (SET) Reactions

Table 3 contains the Gibbs free energy of reaction (∆G°) and activation (∆G^≠^), and the k values pertaining to SET reactions with each peroxyl radical, alongside the gas-phase ionization potentials changes (∆IP) reported by Wright et al. [15]. Calculation of rate constants in this context requires multiplication by the molar fraction of anion that exists in solution at a physiological pH of 7.4 via reported pK_a_ values of monophenols [44]. For polyphenols, pK_a_ values for individual monoanions were predicted using the ∆G–pK_a_ correlation equations at M06-2X(PCM)/6-311++G(d,p) level of theory provided by [44] (see Table S12). The rate constants incorporating a −6.1 kcal/mol correction to the ∆G°_SET_ for the stabilization of the ^•^OOH anion by explicit water molecules, as utilized in [22], are reported as *k_SET_ alongside the original values.

## 4. Discussion

### 4.1. Investigating the Formal-Hydrogen Atom Transfer (f-HAT) Reactions

In f-HAT reactions, a hydrogen atom is transferred from the hydroxyl group of the phenol to the hydroperoxyl (^•^OOH) or methylperoxyl (^•^OOCH_3_) radical. Molecules **1–13** are monophenolic, whereas molecules **14–20** are polyphenolic, warranting several f-HAT reaction sites. This study excludes non-hydroxyl site reactions as they make negligible contributions to f-HAT reactivity.

Several TS conformations were tested regarding the positioning of the peroxyl radical relative to the previously optimized phenol. The tested orientations with lower Gibbs energy are reported; however, it is impossible to assure that these are the most thermodynamically stable structures. Chemical knowledge of stabilizing and destabilizing interactions via intermolecular forces and steric repulsions was consistently used to guide the geometry optimizations. Of particular importance were the polyphenols with adjacent hydroxyl groups as they presented potential for intermolecular hydrogen bonding. In five of sixteen ^•^OOH cases, the non-hydrogen bonding configuration yielded a higher stability (lower ∆G^≠^) but a smaller rate constant in relation to the hydrogen bonding configuration, which exhibited slightly lower stability (differences of ∆G^≠^ ranging from 0.12 to 0.40 kcal/mol) but a significantly (up to an order) higher rate constant. Standard absolute energies, enthalpies, and Gibbs free energy values, including values associated with both situations, are reported in Tables S4 and S5, but the TS configuration leading to the highest rate constant was always used in the kinetic analysis (Section 4.1.2).

#### 4.1.1. Thermodynamic Study of f-HAT Reactions

The f-HAT reactions with ^•^OOH were consistently more exergonic than with ^•^OOCH_3_, being, on average, 1.7–1.8 kcal/mol (in water) and 1.2–2.2 kcal/mol (in PE) more exergonic, which directly reflects the Gibbs energy difference between ^•^OOH/HOOH and ^•^OOCH_3_/HOOCH_3_. The majority of the ^•^OOH scavenging was exergonic in both solvents apart from **16^(1)^** and **16^(2)^** in PE and **19^(2)^**, **19^(3)^**, **20^(3)^**, and **20^(4)^** in both solvents. Scavenging of ^•^OOCH_3_ was similar, but endergonic for a few more sites and solvents (e.g., **18^(3)^** in PE, **18^(1)^** in both, **16^(1)^** and **16^(2)^** in both, and **19^(1)^** in PE). Radical scavenging was more exergonic in a hydrophilic environment than a hydrophobic one, except in three cases (**14^(2)^**, **15^(4)^**, **18^(2)^**). The ∆G° values were, on average, 1.6 kcal/mol and 2.0 kcal/mol more exergonic in water for ^•^OOH and ^•^OOCH_3_, respectively. This finding contrasts with their protein model repair abilities, which were slightly more thermodynamically favourable in PE [11]. This distinction arises from the calculated G° differences between the damaged and repaired protein model in both solvents and that of ^•^OOR and HOOR (polar species are always more stabilized in more polar solvents). The validity of the Bell–Evans–Polanyi principle will be assessed in the next section, where exergonicities (∆G° values) will be contrasted with rate constants to establish whether thermodynamic quantities are reliable kinetic predictors. Inconsistencies in the principle have been highlighted in previous antioxidant studies [8,11,18,20,21,22].

Of molecules **1** and **2**, f-HAT scavenging by **1** was more exergonic in both solvents, consistent with the reactivity predicted by bond dissociation enthalpy (ΔBDE values are also listed in Table 1 and Table 2) of the O-H bond reported by Wright et al. [15] and with the protein repair ability reported by Walton-Raaby et al. [11]. Molecule **1** is 2.1–2.2 kcal/mol and 1.7–2.7 kcal/mol more exergonic in water and PE, respectively, than **2**, for reasons previously explained [11].

Exergonicities of the tocopherol molecules **3–7**, when scavenging both OOH and OOCH_3_, followed their level of methyl-group substitution closely. The order of substitution (**7** > **6** = **5** > **4** > **3**) is almost identical to that of exergonicities (**7** > **5** ≈ **6** > **4** > **3**). This order was also reflected in the tertiary antioxidant activity study [11] and the gas-phase predicted ΔBDE [15].

The aminophenols (molecules **8–11**) exhibited the most exergonic scavenging among the phenols studied, with **10** producing the most negative ∆G° of all (−19.9 kcal/mol in water and −14.3 kcal/mol in PE for ^•^OOH, and −17.3 kcal/mol in water and −12.1 kcal/mol in PE for ^•^OOCH_3_). Again, the order of exergonicities (10 > 11 > 9 > 8) held a relationship with the level of phenol substitution in both water and PE. As mentioned previously [15], methylation of the amino group plays a role in its electron-donating ability, also contributing to the aminophenols trend of gas-phase ΔBDE values, the ∆G° values for protein repair [11], and ^•^OOR scavenging. Of the monophenols, molecules **12** and **13** displayed the least exergonic f-HAT scavenging and produced the only contradiction to gas-phase trends in ∆BDE [15]: radical scavenging by **12** was more exergonic whereas **13** had a more negative ∆BDE.

Analysis of polyphenolic compounds (**14–20**) involves identification of the most thermodynamically favoured hydroxyl to participate in a hydrogen transfer. Reactions from the middle OH groups (**14^(2)^**, **17^(1)^** and **18^(2)^**) were significantly more exergonic than from the adjacent OH ones (average differences of 4.4 kcal/mol in water and 5.8 kcal/mol in PE, including both free radicals). This trend can be attributed to their position as the “internal” hydrogen, located between two hydroxyls which stabilize the radical species, as previously identified [15] and observed [11]. Due to the ester-oriented *para* to the internal hydrogen in **14^(2)^** and **18^(2)^**, which removes electron density and strengthens the O-H bond, molecule **17^(1)^** exhibited the most exergonic f-HAT reactions of all polyphenols (−9.4 kcal/mol in water and PE for ^•^OOH, and −7.7 kcal/mol in water and −7.2 kcal/mol in PE for ^•^OOCH_3_), consistent with thermodynamic results of protein repair [11]. The order of f-HAT exergonicities of epigallocatechin gallate substructures (EGCG) (**17** > **18** > **16**) follow trends in ∆BDE [15]. Each phenoxyl radical resulting from f-HAT scavenging by molecule **15** can be stabilized by one adjacent hydroxyl group. **15^(1)^** and **15^(4)^** are *para* relative to the hydrocarbon link between catechol groups, meaning that the resulting radical can delocalize to that site, resulting in stabilization by donation of electron density via inductive effects/hyperconjugation. Hydroxyls situated *meta* to the alkyl group (**15^(2)^** and **15^(3)^**) do not experience this stabilization, resulting in less exergonic f-HAT reactions. Therefore, **15^(2)^** and **15^(3)^** share similar exergonicities in respective solvents, as do **15^(1)^** and **15^(4)^** in water (within 0.2 kcal/mol). Unexpectedly, **15^(4)^** is more exergonic in PE (−8.9 kcal/mol) than water (−8.1 kcal/mol) for ^•^OOH, a characteristic not shared with the structurally similar **15^(1)^.** The same trend is observed for ^•^OOCH_3_.

Molecules **16**, **19**, and **20** exhibited the least thermodynamically favourable f-HAT scavenging reactions in cases with *meta*-situated pairs of hydroxyl groups (**16^(1)^** and **16^(2)^**, **19^(2)^** and **19^(3)^**, **20^(3)^** and **20^(4)^**). On the contrary, **20^(2)^** was the second/third most exergonic polyphenolic ^•^OOR scavenger in water/PE, respectively (−9.1 kcal/mol in water and −8.4 kcal/mol in PE, for ^•^OOH). A structural rationale for this thermodynamically preferred hydrogen abstraction is the *para*-situated conjugated substituent, as observed with **19^(1)^** as well. Zeppilli et al. [45] corroborate this result in their systematic topological study of radical scavenging by phenols, finding that *meta-*hydroxyl groups produce similar ∆G° to phenol, attributing it to the fact that such positioning does not enable substantial spin delocalization in the resulting phenoxyl radical, unlike *para*- or *ortho*-substituents.

#### 4.1.2. Kinetic Study of f-HAT Reactions

Transition states (TS) were calculated to study the kinetics of f-HAT reactions relative to the thermodynamic study as they account for the presence of electron donating/withdrawing group, local and through bond effects, conjugation, hyperconjugation, solvent effects, and quantum tunnelling. While minimal experimental rate constants exist for the studied reactions, the methodology for their determination applied in this work has been thoroughly explained and validated [8] and the level of theory and solvation method has shown good replication of experimental results [34]. It should be noted that continuum solvation models do not explicitly account for hydrogen-bonding interactions or solvent-structure effects, which may impact the energetics of certain species and reactions.

In Table 1 and Table 2, the ∆G^≠^ and k values for the f-HAT reactions in water and PE for ^•^OOH and ^•^OOCH_3_ scavenging reactions are respectively listed, and total k values for polyphenols are also reported. Due to unsuccessful geometry optimizations of the TS for molecules **9**–**11** with both peroxyl radicals in water, correlations of log(k) in water versus ∆G° (Figure 2; R^2^ = 0.96 and Figure S2; R^2^ = 0.67), and log(k) in water versus log(k) in PE (Figure S1; R^2^ = 0.76 and Figure 3; R^2^ = 0.91) were attempted as an alternative to predict their rate constants. The approach which produced the best correlation for each radical was used to make the rate constant prediction in water as reported in Table 1 and Table 2 for these molecules. These correlations involved the kinetic values of all other monophenols (**1**–**8**, **12** and **13**), and when the values for the polyphenols were included for ^•^OOH scavenging reactions, the R^2^ values decreased from 0.76 to 0.72 and from 0.96 to 0.88. It is unsurprising that the correlations are better when structurally similar phenols (e.g., monophenols) are considered.

When inspecting solvent trends for ^•^OOH, all monophenols had lower ∆G^≠^ in PE than in water, but the k values were not always higher in PE: that is the case of molecules **8**, **12**, and **13**. In agreement with a larger k in water than in PE for the aminophenol **8**, the predicted k values for **9–11** in water followed the same trend. Interestingly, the monophenols exhibited more exergonic reactions in water. In summary, molecules **1–7** produced rate constants up to one order greater in PE, whereas aminophenols (**8**–**11**) and stilbenes (**12** and **13**) showed faster ^•^OOH scavenging in water. A commonality shared for **1–7** is a *para*-oriented ether, suggesting that BHAs and tocopherols may be better at ^•^OOH scavenging in hydrophobic cellular environments, like the lipid bilayer, due to this structural feature. Conversely, aminophenols and those particular stilbenes may do so more efficiently in hydrophilic environments. This nuance between ∆G^≠^ and k solvent trends highlights the importance of calculating rate constants.

Evaluation of the same concepts but regarding ^•^OOCH_3_, results in unclear solvent trends. For the ten TSs with ∆G^≠^ values determined for each solvent, five were more stable in PE, including the stilbenes (**12**–**13**) and three of the tocopherols (**2**–**4**). However, rate constants in water (including predicted values for **9**–**11**) were greater than in PE for all monophenols, by an average of approximately half an order of magnitude.

Of the butylated hydroxyanisoles (**1**–**2**), molecule **1** scavenging of ^•^OOH in PE had the largest rate constant (5.1 × 10^6^ M^−1^s^−1^). ^•^OOH scavenging rates in PE by both **1** and **2** were higher than in water (the opposite is observed with ^•^OOCH_3_), inconsistent with thermodynamic studies which favoured reaction in water. The higher reactivity of **1** relative to **2** is consistent with the thermodynamic results, their tertiary activity [11], their -∆BDE in the gas phase [15], and commercial BHA mixture compositions (9:1 ratio of **1**:**2**) [24]. However, **2** is predicted to be more reactive than **1** in both solvents when reacting with ^•^OOCH_3_. The rate constant for ^•^OOCH_3_ scavenging by **1** in PE (4.19 × 10^5^ M^−1^ s^−1^) is 4.1 times higher than the experimental value for a similar molecule (**1** but with an additional *ortho*-tertbutyl group) when scavenging ^•^OOR in chlorobenzene (1.2 × 10^5^ M^−1^ s^−1^) determined via oxygen consumption during styrene autoxidation [46].

Analogues of vitamin E (**3–7**) had rate constant trends which closely fit the expectations of the thermodynamic study. The fastest peroxyl radical scavenging was performed by **7**, with ^•^OOCH_3_ in water exhibiting the largest tocopherol rate constant, which was almost half an order of magnitude below the diffusion limit (4.9 × 10^7^ M^−1^s^−1^). Interestingly, in PE, the rate constant for the same reaction is notably lower (7.7 × 10^6^ M^−1^s^−1^), a difference which was not observed for ^•^OOH scavenging performed by **7** in water (2.7 × 10^7^ M^−1^s^−1^) and PE (2.9 × 10^7^ M^−1^s^−1^). A kinetic deviation occurs from thermodynamic expectations for molecules 5 and 6, as their exergonicities are nearly the same but the rate constant for **5** is significantly larger (by 1.2–1.6 × 10^6^ M^−1^s^−1^) when scavenging either radical in water, whereas similar rate constants resulted in PE. This indicates that the *para* (**5**) versus *ortho* (**6**) orientation of methyl substituents, relative to each other, affects the peroxyl scavenging ability of tocopherols in water, but not in PE. In PE, ^•^OOH is consistently scavenged faster than ^•^OOCH_3_, according to larger rate constants (by up to an order). In water, the k values between radicals are similar, except for **4** and **7** which are 1.8 times larger for ^•^OOCH_3_. The α-tocopherol analogue (**7**) is the most bioactive form of vitamin E in human blood plasma due to its selective retention and transfer [47]. Our near diffusion-controlled rate constants involving this phenolic antioxidant corroborates its known role as an efficient in vitro and in vivo inhibitor of lipid peroxidation [25]. The ^•^OOR scavenging rate constant for 7, experimentally determined in chlorobenzene, is 3.2 × 10^6^ M^−1^ s^−1^ [46], which is 2.4 times less than our quantity of 7.65 × 10^6^ M^−1^ s^−1^ determined for ^•^OOCH_3_ scavenging in PE.

**Figure 4:**
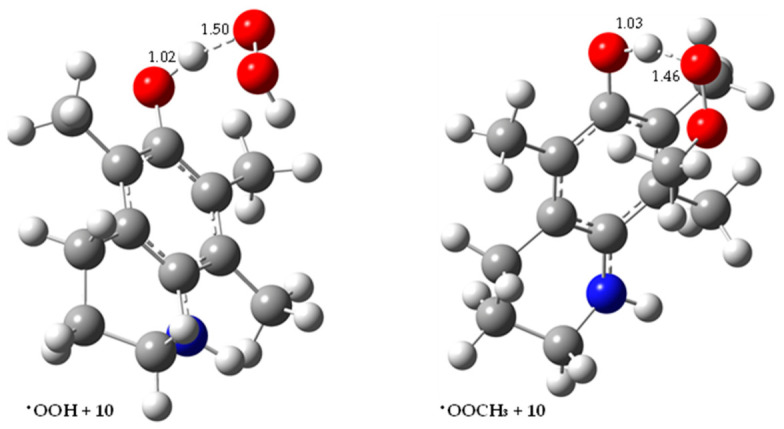
TS structures for the f-HAT scavenging of ^•^OOH and ^•^OOCH_3_ by molecule **10** in PE (important bond distances shown in Å).

The aminophenols (**8–11**), having the most exergonic reactions, displayed the highest rate constants of the f-HAT study in both solvents for both radicals, closely followed by **7**. Molecules **10** and **11** (only with ^•^OOH) in both solvents have rate constants in the diffusion limit (10^8^ M^−1^s^−1^), indicating biochemical significance. In PE, ^•^OOH scavenging was more efficient than ^•^OOCH_3_, whereas the predicted rate constants in water were larger for the latter radical. The highest rate constants were calculated for **10** with ^•^OOH (9.1 × 10^8^ M^−1^s^−1^ in PE, and 1.5 × 10^9^ M^−1^s^−1^ in water obtained via correlation) and with ^•^OOCH_3_ (4.2 × 10^8^ M^−1^s^−1^ in PE, and 2.9 × 10^9^ M^−1^s^−1^ in water obtained via correlation). Their TS structures in PE are displayed in Figure 4. Kinetic studies of f-HAT repair of a protein model found that molecule **10** was the only phenol studied with physiologically relevant tertiary antioxidant activity [11]. Our results suggest that aminophenols are more likely to engage in peroxyl radical scavenging (primary antioxidant activity) than protein repair (tertiary antioxidant activity) when considering f-HAT reactions.

At the other end of the spectrum, ^•^OOH scavenging by the stilbenes (**12** and **13**) was the least efficient of the monophenols studied, as predicted by their minimal exergonicities and in congruence with their protein repair ability. Interestingly, rate constants for OOCH_3_ scavenging by **12** (2.7 × 10^6^ M^−1^s^−1^ in water and 6.3 × 10^5^ M^−1^s^−1^ in PE) were on par with that of 4 (2.6 × 10^6^ M^−1^s^−1^ in water and 1.0 × 10^6^ M^−1^s^−1^ in PE). The ∆BDE in the gas phase [15] predict antioxidant activity near that of **2–4** for both **12** and **13**, which is only observed kinetically for **4**, **12** (and **2**, **12**) scavenging ^•^OOCH_3_ in water (in PE), and for **2**, **12** and **3**, **4**, **13** in water scavenging ^•^OOH.

When holistically comparing f-HAT primary and tertiary antioxidant activities of the monophenols, in all except one case for ^•^OOH and three cases for ^•^OOCH_3_, scavenging by the phenol was faster than protein repair of the fastest site (i.e., β, γ, or δ). For both radicals an exception was compound **1** in PE, which showed a more efficient δ-site protein repair than ^•^OOH and ^•^OOCH_3_ scavenging (1.2 × 10^7^ M^−1^s^−1^, 5.1 × 10^6^ M^−1^s^−1^, and 4.2 × 10^5^ M^−1^s^−1^, respectively). This may be attributed to the stabilizing interaction between the amide of the protein and the phenolic hydroxyl, which is not present for other reaction sites. When in a hydrophobic environment, molecules **3**, **7**, and **8** are also more efficient at donating hydrogen to a damaged protein than ^•^OOCH_3_ (showing better tertiary than primary antioxidant activity).

Alignment of the trends resulting from the thermodynamic and kinetic studies within respective solvent models and phenol groups (i.e., BHAs, tocopherols, aminophenols, and stilbenes) was near perfect for the monophenols. In agreement with our findings, analysis of Gibbs free energies of reaction and activation within a group of *para*-substituted monophenols during ^•^OOH scavenging yielded a consistent relative trend between thermodynamics and kinetic feasibility [45]. In our study, the overall order of rate constants in water and PE deviate from each other and from that of their exergonicities. The overall order regarding exergonicities is **10** > **11** > **9** > **8** > **7** > **5** ≈ **6** > **1** > **4** > **3** > **2** > **12** > **13**, except for ^•^OOCH_3_ scavenging in PE, where 1 is more exergonic than **5**. The order of ^•^OOH rate constants in water is **10** > **11** > **9** > **8** > **7** > **5** > **1** > **6** > **4** ≈ **3** > **2** > **12** > **13** and in PE is **10** > **11** > **9** > **7** > **8** > **5** ≈ **6** ≈ **1** > **4** > **2** > **3** > **12** > **13**. The kinetic trend most significantly differs from thermodynamic expectations for compounds **1** and **6** in water, and **7** and **8** in PE, in that their order is switched. For ^•^OOCH_3_, the kinetic order is **10** > **11** > **9** > **7** > **8** > **5** > **6** > **12** > **4** > **3** > **2** > **1** > **13** in water and **10** > **11** > **9** > **7** > **8** > **6** > **5** > **4** > **12** > **2** > **1** > **3** > **13** in PE, showing a greater number of differences to the thermodynamic orders. The order of exergonicities of peroxyl scavenging in water is identical to that of oxidized protein repair in water [11], whereas in PE, differences in the order of **1**, **5**, and **6** are regarded between radical types.

Notably, the calculated rate constant order of protein repair shows greater difference relative to its thermodynamic order than in this study, indicating thermodynamics may predict radical scavenging kinetics better when the radical is a less complex species (i.e., damaged protein versus ^•^OOH). Thermodynamics predicted ^•^OOCH_3_ scavenging kinetics at an intermediate level, between ^•^OOH and the damaged protein. Another significant discrepancy arises when assessing the polarity of the environment (hydrophilic versus hydrophobic) in which thermodynamic studies expect faster radical scavenging. For all monophenols when scavenging either peroxyl radical, reactions proved to be more exergonic in water. In congruence with the Bell–Evans–Polanyi principle, this would imply lower ∆G^≠^ and greater rate constants in water as well. However, for reactions involving ^•^OOH, ∆G^≠^ values were lower in PE for all the species studied and rate constants were greater in PE for 7 of the 13 species, as detailed earlier in this section. For ^•^OOCH_3_, the thermodynamically preferred solvent did correspond to the kinetically preferred solvent (water), but trends were inconsistent regarding ∆G^≠^ values. These order and solvent discrepancies add to the evidence contributing to the breakdown of the Bell–Evans–Polanyi principle.

The multi-sited nature of polyphenols, **14–20**, allows for comparisons between ^•^OOR scavenging abilities of hydroxyl groups within the same molecule and between molecules. Total rate constants were calculated to elucidate a comprehensive order of rate constants within the polyphenols and with the monophenols as well. None of the polyphenols produced biologically relevant f-HAT rate constants.

According to the total rate constants of **15** in water (2.1 × 10^6^ M^−1^s^−1^ with ^•^OOH and 2.4 × 10^6^ M^−1^s^−1^ with ^•^OOCH_3_) and in PE (1.7 × 10^6^ M^−1^s^−1^ with ^•^OOH and 3.8 × 10^6^ M^−1^s^−1^ with ^•^OOCH_3_), it is the most efficient ^•^OOR scavenger of the polyphenols studied, a standing which the thermodynamic study projected to be filled by **17** (particularly **17^(1)^**). Overall, **15** is better at scavenging ^•^OOCH_3_ than ^•^OOH. Molecule **15** was also the most efficient at protein repair (3.4 × 10^7^ M^−1^s^−1^ in PE), an order more so than ^•^OOH and ^•^OOCH_3_ scavenging. Another observation of note for ^•^OOH scavenging is that k for **15^(1)^** and **15^(4)^** in PE was an order greater than for **15^(2)^** and **15^(3)^**, as expected by structural similarities and exergonicities. However, in water, **15^(2)^** and **15^(3)^** exhibited k in the same order as **15^(1)^** and **15^(4)^** (k ranging from 3.4 to 7.3 × 10^5^ M^−1^s^−1^). Despite not being within the diffusion limit, it is noteworthy that **15^(1)^** produced the largest individual ^•^OOCH_3_ scavenging rate constants in respective solvents (8.1 × 10^5^ M^−1^s^−1^ in PE and 2.7 × 10^6^ M^−1^s^−1^ in water).

According to the thermodynamic study, rate constants for ‘internal’ hydroxyls (**14^(2)^**, 17^(1)^, and **18^(2)^**) should be greater relative to external hydroxyls. Their protein repair (only studied in PE) [11] and current ^•^OOCH_3_ rate constants in both solvents and ^•^OOH rate constants in water follow this expectation, but in PE, deviations occur. In PE, external hydroxyls of **17** were an order faster at ^•^OOH scavenging than were its internal hydroxyls. As for **14** and **18** scavenging ^•^OOH in PE, a similar trend occurred between them in which only one of the external OH (**14^(3)^** and **18^(3)^**) had a rate constants one order below that of the other OH groups. Interestingly, the rate-enhancing effect of adjacent hydroxyl groups during ^•^OOCH_3_ scavenging was more significant in PE than in water. For example, the rate constant of **18^(2)^** (6.8 × 10^5^ M^−1^s^−1^) in PE is ~1.5 log units greater than **18^(3)^** (1.9 × 10^4^ M^−1^s^−1^) in PE, but only ~1.0 log units greater in water (2.9 × 10^5^ M^−1^s^−1^ and 3.1 × 10^4^ M^−1^s^−1^, respectively). Kinetic analyses show that, via f-HAT reactions, these three polyphenols are more effective at protein repair than ^•^OOR scavenging in a hydrophobic environment.

**Figure 5:**
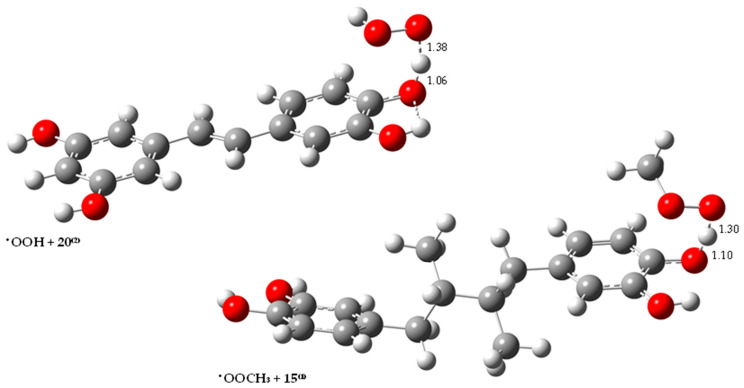
TS structures for the f-HAT scavenging of ^•^OOH and ^•^OOCH_3_ by molecules 20^(2)^ (in water) and 15^(1)^ (in PE), respectively (important bond distances shown in Å).

Another molecule with adjacent OH groups is **20**, which follows a uniform intramolecular trend between water and PE, kinetics and thermodynamics, ^•^OOH and ^•^OOCH_3_, and primary and tertiary activity. **20^(2)^** was the most efficient ^•^OOH scavenger with k values between 1.2 and 1.5 × 10^6^ M^−1^s^−1^ due to two factors: (1) the *para*-oriented conjugated system donates electron density, weakening the OH bond and, (2) the radical species is stabilized by an adjacent hydroxyl (**20^(1)^**). Molecule **19^(1)^** is structurally identical to **20^(2)^**, minus the adjacent hydroxyl, directly showing the importance of that interaction in enhancing the rate constant. The relative antioxidant activity of **19** and **20** observed in this study is consistent with experimental observations of their lipid peroxyl radical scavenging abilities [48]. Wright et al. [15] projected **20** as the most active phenol at free radical scavenging, and when considering individual hydroxyl groups rather than total rates, **20^(2)^** did show the highest ^•^OOH scavenging rate of the polyphenols studied, but not when considering the entire set of phenols. However, the same cannot be said for its ^•^OOCH_3_ scavenging rates, which were less than certain sites in **14**, **15**, **17**, and **18**. The TS structures of the polyphenols which produced the greatest rate constants for each radical (**20^(2)^** with ^•^OOH in water and **15^(1)^** with ^•^OOCH_3_ in PE) are shown in Figure 5. It is apparent from the bond distances that the reaction with these polyphenolic species (**15** and **20**) produce a later TS relative to the species shown in Figure 4 (**10**), as the ArO-H distance is lengthened and the ROO-H distance is shortened, consistent with their lower reactivity relative to aminophenols.

Molecule **20** contains the sites which are the least efficient ^•^OOR scavengers, **20^(3)^** and **20^(4)^**. *Meta*-situated hydroxyl pairs and *meta*-oriented electron donating groups (**20^(3)^** and **20^(4)^**, **19^(2)^** and **19^(3)^**, and **16^(1)^** and **16^(2)^**) have minimal rates due to strengthened OH bonds, and destabilized radical species. The presence of the *meta*-situated hydroxyls dampens the ^•^OOH scavenging effect of **19^(1)^** as shown by the fact that **13**, the monophenol with the same conjugated system, had a larger k. The total rate constant for **19** scavenging ^•^OOCH_3_ in PE (2.89 × 10^4^ M^−1^ s^−1^) is about 6.9 times less than the k for its reaction with ^•^OOR in chlorobenzene (2.0 × 10^5^ M^−1^ s^−1^) as experimentally determined in [46]. The comparison between these experimental rates [46] with **1**, **7**, and now **19** are indirect as they differ in radical species and solvent. Nevertheless, it is surprising that the sterylperoxyl scavenging rate constant is greater than that of ^•^OOCH_3_ (and even ^•^OOH), since peroxyl radicals are generally considered less reactive than simple alkyl peroxyl radicals (and especially hydroperoxyl radicals). This deviation highlights the potential environmental factors which may play a role in scavenging kinetics, which our computational work does not capture.

The ability of the thermodynamic study to predict the medium polarity of higher f-HAT activity for polyphenolic species was successful in 13 of the 21 ^•^OOH cases, and in 11 of the 21 ^•^OOCH_3_ cases (overall 57% success rate for polyphenols), further highlighting the inconsistency of such a prediction. Between hydroxyls of a single polyphenol, exergonicity trends were consistent between solvents, whereas kinetic studies showed variation between solvents for **14**, **15**, and **17**. Intramolecular polyphenolic trends of rate constants were better predicted by thermodynamics in water versus PE (71% and 57% respectively, of the seven polyphenols, considering both radicals). Thermodynamics predicted reactivity trends (regarding the different sites of a given polyphenol) more consistently for ^•^OOCH_3_ than ^•^OOH (79% versus 57% correctly predicted, respectively). The numerous differences between thermodynamic and kinetic results contribute to the breakdown of the Bell–Evans–Polanyi principle on multiple levels: within polyphenols, within phenol groups, between phenol groups, and between solvents.

While the monophenols studied were generally faster at ^•^OOR scavenging than protein repair, the polyphenols exhibited overall faster protein repair (in PE), potentially due to the greater opportunity for intermolecular interactions which may stabilize the transition state. Rate constants of repair (by the most efficient hydroxyl site) were at least an order greater than ^•^OOH scavenging for **14**, **15**, **17**, and **18**, around half an order greater than that of **16** and **19**. Furthermore, within a given phenol, ^•^OOH scavenging rates often had a wider range than for the protein repairs. For example, primary activity rates of **18** in PE ranged from 3.8 × 10^3^ M^−1^s^−1^ (**18^(3)^**) to 1.2 × 10^5^ M^−1^s^−1^ (**18^(2)^**) while analogous tertiary activity rates ranged from 3.8 × 10^6^ M^−1^s^−1^ (**18^(3)^**) to 5.0 × 10^6^ M^−1^s^−1^ (**18^(2)^**). This finding may be explained by the nature of the radical receiving the hydrogen atom, and the potential of secondary stabilizing interactions in the TS which could be more abundant when repairing a larger species.

**Figure 6:**
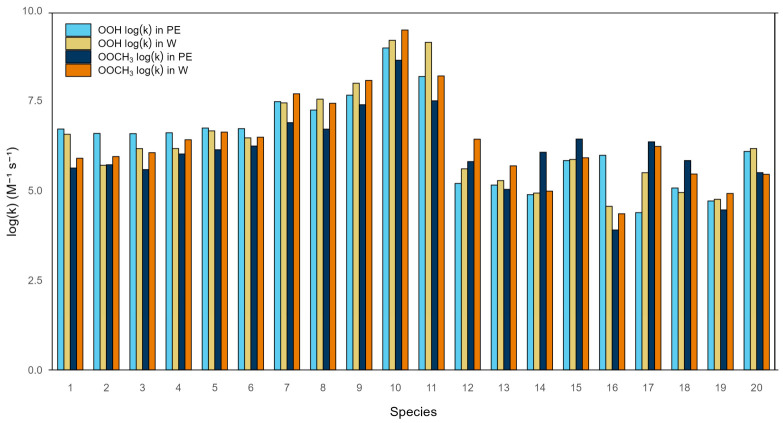
Comparison between the largest f-HAT log(k) values for primary activity with respect to ^•^OOH and ^•^OOCH3 in water and PE.

To conceptualize the kinetic results in terms of the radical species of focus, the rate constants produced by the ^•^OOH and ^•^OOCH_3_ quenching reactions (considering the most efficient site of each phenol, when applicable) in water and PE were plotted, as shown in Figure 6. Consistently for monophenols, reaction with ^•^OOCH_3_ in PE yielded the lowest relative efficiency, whereas for some polyphenols (like **14**, **15**, **17**, and **18**) the opposite was observed. Visually, the phenols which exhibited the most variation when considering the polarity of the solvent are **11**, **16**, and **17**. Molecule **11** is an impressive ^•^OOH scavenger in a polar setting, more so than in a hydrophobic environment. Bar ratios resulting for molecules **16** and **17** mirror one another, as **16** presents better quenching of OOH and **17** does so for ^•^OOCH_3_.

**Figure 7:**
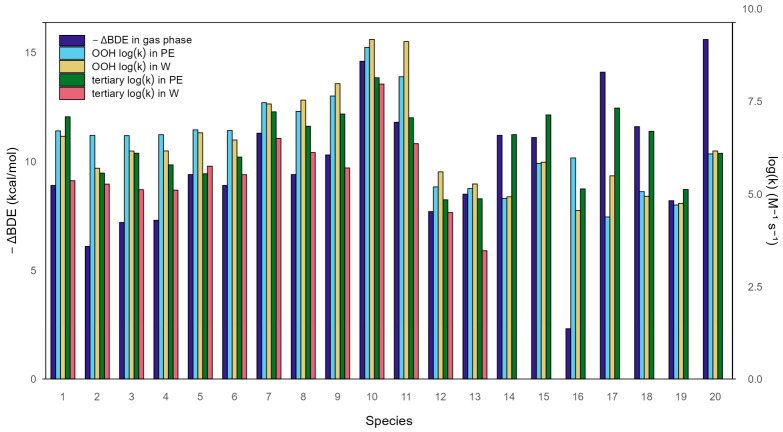
Comparison between −∆BDE in the gas phase [15], the largest f-HAT log(k) values for tertiary activity [11], and f-HAT log(k) values for primary activity with respect to ^•^OOH in water and PE.

**Figure 8:**
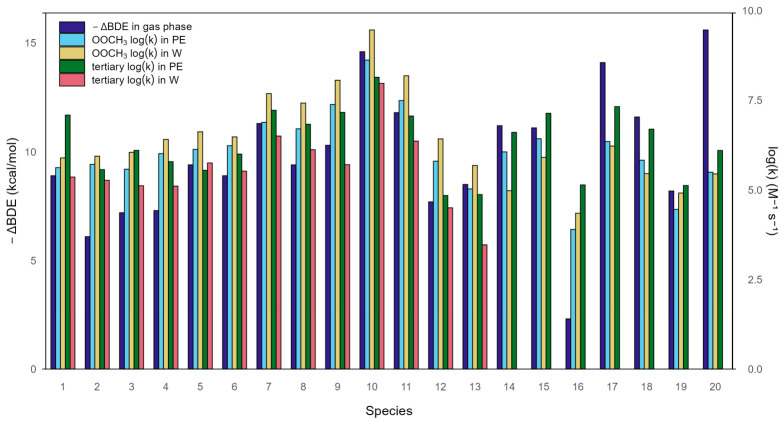
Comparison between −∆BDE in the gas phase [15], the largest f-HAT log(k) values for tertiary activity [11], and f-HAT log(k) values for primary activity with respect to ^•^OOCH_3_ in water and PE.

To provide a holistic picture of how the antioxidant activities predicted by gas phase properties calculated by Wright et al. [15], the tertiary antioxidant activity evaluated by Walton-Raaby et al. [11], and the primary antioxidant activity of these phenolic compounds correlate, −∆BDE was plotted against the logarithm of the largest k available for their primary and tertiary rate constants in water and PE, yielding a five-way comparison, as seen in Figure 7 and Figure 8 for reaction with ^•^OOH and ^•^OOCH_3_, respectively. When viewing Figure 7 and looking only at bars corresponding to the ^•^OOH and tertiary rate constants in water and PE for species **1–13**, a relatively uniform bar size ratio is observed, often led by ^•^OOH rate constants in PE (except for **8–12**, which was previously discussed), followed by ^•^OOH and tertiary rate constants in water and PE (respectively), and tailed by tertiary rate constants in water. The corresponding bar size ratio in Figure 8 is generally led by ^•^OOCH_3_ rate constants in water, however the order in which the equivalent rate constant in PE and the tertiary rate constants appear is variable between monophenols. For example, sometimes the tertiary rate constant in PE is greater than that for ^•^OOCH_3_ scavenging in PE (see species **7** and **8**). While **20** was of the fastest polyphenolic ^•^OOR scavengers in water, it is nowhere near the level that was predicted by ∆BDE. Conversely, the repair and scavenging rates by **16** are significantly faster than predictions by ∆BDE, but the slowest in the context of kinetic data. Molecules **10** and **11** exhibited similarly large rate constants of ^•^OOH scavenging; however, ∆BDE predicted a larger disparity between their activities, closer to what is seen for ^•^OOCH_3_ scavenging. 

In discussing primary versus tertiary activity, it is important to note that for compounds with high activity in both scenarios (i.e., **10**), their biological action would depend on whether the damaged protein or peroxyl radical is in its proximity [49].

In whole, the kinetic examination of peroxyl scavenging found that **9–11** have rate constants within the diffusion limit for f-HAT reactions, suggesting biologically significant ^•^OOH (**10**, **11**) or ^•^OOCH_3_ (**9–11**) scavenging abilities in hydrophobic (**10**, **11**) and hydrophilic environments. Violations of the Bell–Evans–Polanyi principle discussed give evidence that thermodynamic quantities are an inadequate measure of kinetic antioxidant activity. However, as determination of Gibbs free energies of reaction does not require optimization of transition states, thermodynamic determination of ender/exergonicity is a valuable screening tool for compounds that warrant further, kinetic evaluation. An important consideration is that endergonic reactions may be viable when the products are consumed rapidly in a subsequent step [8]. Rate constants of peroxyl radical scavenging and protein repair were contrasted to uncover each phenol’s preference toward participation in primary or tertiary antioxidant activity relative to f-HAT reactions. Furthermore, their reactivities with ^•^OOH and ^•^OOCH_3_ were compared to contribute an even greater understanding of contexts in which these antioxidants may act. We also discussed disagreements between radical scavenging ability determined by rate constants in solution with ΔBDE values calculated in the gas phase. While peroxyl scavenging by f-HAT reactions by these twenty phenols showed minimal biochemical significance, the study of SET reactions produced different results.

### 4.2. Investigating Radical Scavenging by SET Reactions

SET reactions have several forms which can add to a compound’s overall antioxidant ability. In this study, SET rate constants refer to the anionic phenol scavenging ^•^OOH or ^•^OOCH_3_ in water, as was the case when studying their protein repair via SET [11]. Calculation of SET rate constants in PE is not necessary as deprotonation is only thermodynamically viable in a protic environment. Table 3 reports the SET rate constant values excluding (k_SET_) and including (*k_SET_), a −6.1 kcal/mol correction for ∆G°_SET_, corresponding to the explicit stabilization of the OOH anion by water. The correction introduces an average rate constant increase of 0.90 (1.13) log units, ranging from 0.01 to 2.16 (0.02 to 2.63) log units for ^•^OOH (^•^OOCH_3_). An additional seven (eleven) *k_SET_ values are diffusion controlled for ^•^OOH (^•^OOCH_3_), and in such cases, changes in relative trends arise. Therefore, these values are included due to their potentially greater accuracy: however, to maintain consistency with [11] and comparability between f-HAT and SET kinetics, while avoiding the assumption that the same correction applies for ^•^OOCH_3_, the following discussion refers to non-corrected k_SET_ values.

All phenols studied showed significantly better SET activity during peroxyl radical scavenging than during protein repair. The greatest rate constant of protein repair by SET reaction was 5.8 × 10^−6^ M^−1^s^−1^ by molecule **10**, while the lowest for ^•^OOH scavenging with **20^(4)^** was 1.2 × 10^5^ M^−1^s^−1^.

Of the monophenols (**1–13**), nine (seven) of them exhibited rate constants within the diffusion limit with ^•^OOH (^•^OOCH_3_), implicating physiological relevance. For BHAs (**1–2**), molecule **1** had a higher k_app_, following expectation of industry application and trends in aqueous f-HAT kinetics, but defying the prediction of -∆IP which suggests **2** is a slightly better SET scavenger.

Tocopherols (**3–7**) have SET abilities in the same sequence as f-HAT rate constants and gas phase -∆IP, the fastest being that of **7** (2.3 × 10^8^ M^−1^s^−1^ for ^•^OOH and 1.50 × 10^8^ M^−1^s^−1^ for ^•^OOCH_3_). The less-substituted **3** and **4** were the only two tocopherols which yielded ^•^OOH scavenging rate constants just below the diffusion limit, but were still an order greater than their f-HAT counterparts. In terms of electron transfers to ^•^OOCH_3_, only those done by **5** and **7** were diffusion controlled.

Aminophenols (**8–11**) had greater SET rate constants than all other monophenols, with a k_app_ range of (3.7–8.6) × 10^8^ M^−1^s^−1^ for ^•^OOH and (2.2–7.0) × 10^8^ M^−1^s^−1^ for ^•^OOCH_3_, in orders (**11** > **10** > **8** > **9** and **11** > **8** > **10** > **9**, respectively) which did not match one another, or that of f-HAT kinetics (**10** > **11** > **9** > **8**). Each aminophenol showed greater ^•^OOH quenching abilities than for ^•^OOCH_3_. Antioxidant predictions by −∆IP place **9** ahead of **8** by a considerable amount (−8.4 kcal/mol), but it is apparent that during SET, the enhanced electron-donating effect by the methylated amino group in **9** does not improve rate constants as it does for f-HAT reactions. Study of -∆IP predicts **8** as having lower SET capacity than **4–7**, which is not reflected by its peroxyl scavenging kinetics in water. However, the calculated gas-phase ∆IP values refer to the neutral phenols, and the estimated value for the anion would be more relevant for the current comparison.

Stilbenes **12** and **13** exhibited non-biologically significant peroxyl radical scavenging rate constants similar to those of **3** and **4**. However, ^•^OOH scavenging by **12**, yielded a rate constant very close to the diffusion limit (9.8 × 10^7^ M^−1^s^−1^). Molecule **12** was more efficient than **13**, for f-HAT reactions, but SET rate constants were two orders greater than that of the latter indicating that they are more likely to react by SET in a hydrophilic environment. Another inconsistency with -∆IP presents itself for this phenol group, as it predicts superior activity by **13**, on par with that of **6**, which was not observed for either ^•^OOR species evaluated.

Of the polyprotic species (**14–20**), twenty-one monoanions can occur, nine and three of which showed biologically significant rate constants for electron transfer to ^•^OOH and ^•^OOCH_3_, respectively. Molecule **17^(1)^** provided the largest k_app_ of the entire study at 2.8 × 10^9^ M^−1^s^−1^, with its adjacent monoanion species presenting a k value which was three orders lower. Electron transfer to ^•^OOCH_3_ from **17^(1)^** was two orders lower (1.2 × 10^7^ M^−1^s^−1^), and therefore not biochemically relevant. The stabilization of internal monoanions by two adjacent hydroxyls, seen in **14^(2)^**, **17^(1)^**, and **18^(2)^**, was consistently reflected in their kinetic SET ^•^OOH scavenging trends, but for both **14** and **18**, an external site exhibited greater ^•^OOCH_3_ rate constants than that of the internal. Interestingly, while SET is the preferred pathway (over f-HAT) for reaction with ^•^OOH for all sites of these three species, f-HAT is more likely to occur during ^•^OOCH_3_ scavenging by **14^(2)^** and **18^(2)^**.

Regarding the components of EGCG (**16–18**), our data suggests a reactivity sequence of **17** > **18** > **16** with ^•^OOH and **17** > **16** > **18** with ^•^OOCH_3_ (with respect to the most efficient hydroxyl) where SET is usually the more likely ^•^OOR scavenging mechanism in water. The SET activity of **17** is in concordance with [50], but predictions via −∆IP expect **16** to exhibit greater activity. The reported biological activity of EGCG is broad, from reducing mitochondrial damage to acting in the endogenous antioxidant defence system [51]. But in the context of this work, its ability to scavenge free radicals (particularly through SET) is kinetically supported.

The overall second-most efficient polyphenolic SET reaction was by **20^(2)^** when scavenging ^•^OOH, yielding a k_app_ of 2.0 × 10^9^ M^−1^s^−1^. Its ^•^OOCH_3_ quenching ability was greater than that of all other polyphenols, with a k_app_ of 7.1 × 10^8^ M^−1^s^−1^. The adjacent monoanion, **20^(1)^**, also had a biologically significant SET reaction with ^•^OOH (not with ^•^OOCH_3_), but the resulting radical lacks further stabilization by *para*-conjugation resulting in a lower k_app_. By comparing **20^(2)^** with **19^(1)^**, it is clear that the presence of an adjacent hydroxyl significantly impacts rate constants of SET reactions as they differed significantly for each ^•^OOR species. The greater antioxidant activity by piceatannol (**20**) than resveratrol (**19**) we report is consistent with several studies reviewed in [52], where its extensive biological superiority to the latter is communicated in detail.

Each monoanion of **15** yielded an ^•^OOH scavenging rate constant within the diffusion limit, the greatest by **15^(1)^** (8.0 × 10^8^ M^−1^s^−1^), similar to (but much more efficient than) f-HAT results. Monoanions **15^(1)^** and **15^(4)^** also showed biochemically relevant ^•^OOCH_3_ SET k_app_ values of 2.7 × 10^8^ M^−1^s^−1^ and 2.0 × 10^8^ M^−1^s^−1^, respectively. Monoanions **20^(3)^**, **20^(4)^**, **19^(2)^**, **19^(3)^**, **16^(1)^**, and **16^(2)^** presented the lowest SET rate constants of the study, proving that *meta*-orientation of substituents is not a structural feature of high-efficiency peroxyl radical scavengers via f-HAT or SET. This said, in each of these cases their efficiency is still greater via SET than f-HAT.

**Figure 9:**
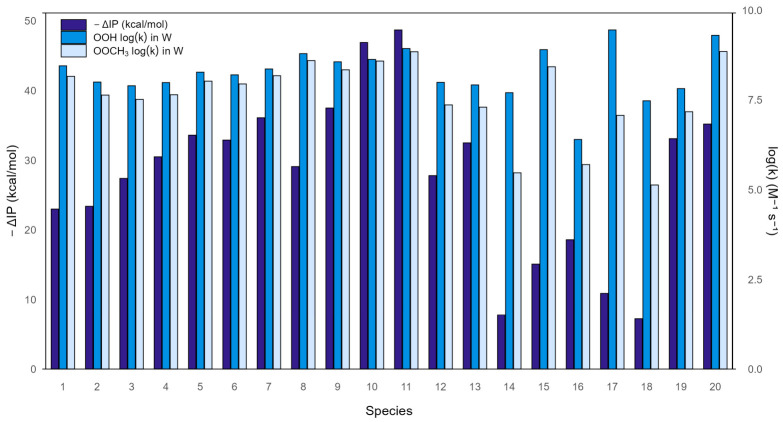
Comparison between −∆IP in the gas phase [15] and SET log(k) values for primary activity in water with respect to ^•^OOH and ^•^OOCH_3_.

To visualize the differences between SET trends of the antioxidant activity predicted by Wright et al. [15] using adiabatic gas-phase properties and the primary antioxidant activity determined by kinetic calculations, -∆IP values have been plotted relative to the logarithm of the largest k_SET_ available for ^•^OOH and ^•^OOCH_3_ scavenging as seen in Figure 9. For example, while electron transfer to ^•^OOH from **17** was the fastest reaction overall according to this study, its -∆IP ranks it as the third least active. Similarly large deviations are also observed for **14**, **15**, and **18**. The best agreement between overall −∆IP and k_SET_ trends for both radical species is found for **10** and **11**. This is likely due to their high intrinsic reactivity, leading to a similar limitation by the diffusion limit between each radical. The SET rates for ^•^OOH were dependably greater than for ^•^OOCH_3_ in each species, however greater levels of disparity are observed for those with three adjacent hydroxyl groups (**14**, **17**, and **18**). In relation to these results, the tertiary antioxidant activity trend delineated by Walton-Raaby et al. [11] showed a wide range of (negligible) rate constants which align more closely overall to ∆IP trends.

It is necessary to point out that our SET reactions consider each phenol as an anionic reactant and neutral product in aqueous solution, whereas the IP values reflect a neutral reactant and cationic product in the gas phase. Walton-Raaby et al. [11] speculated that perhaps in cases where large geometry differences exist between cationic and neutral species in the gas phase, larger discrepancies of this nature arise. However, the high efficiency and greater biological significance of ^•^OOR scavenging relative to protein repair (of amino acids without heteroatoms) by these phenols means the trend of primary antioxidant activity is controlled by the diffusion limit as opposed to structural differences. It is possible that inter-phenolic rate constant trends of SET reactions would yield better correlation with gas-phase predictions when considering a neutral reacting phenol; however, such results would be less biochemically meaningful and likely generate rate constants of lower magnitudes [53]. Wright et al. [15] predicted that f-HAT reactions are the most likely mechanism for all twenty phenols except for the substituted aminophenols, **10** and **11**, which they expect will more likely react by SET in the context of ionization of the neutral species in the gas phase.

### 4.3. Summary of Primary Antioxidant Activity Findings

When drawing conclusions on the most likely mechanism of ^•^OOH or ^•^OOCH_3_ scavenging for each of the twenty phenols using kinetic results, it is expected that majority of the phenols will act through SET reactions in hydrophilic environments aside from molecule **10** which, despite exhibiting biologically relevant rate constants via both f-HAT and SET during both ^•^OOH and ^•^OOCH_3_ scavenging, has higher f-HAT rate constants in the order of 10^9^ M^−1^s^−1^. Molecules **17** (with ^•^OOH), a component of epigallocatechin gallate, and **20** (with ^•^OOCH_3_), a stilbene named piceatannol, exhibited the largest SET rate constants (also in the order of 10^9^ M^−1^s^−1^), but far less impressive f-HAT rate constants. Furthermore, the phenols showed a greater SET reactivity toward ^•^OOH as opposed to ^•^OOCH_3_. Table 4 was constructed to provide an overview of structural aspects of the phenolic compounds in connection with dominant solvent and mechanisms of antioxidant activity during scavenging of each peroxyl radical studied.

## 5. Conclusions

This work assessed the primary antioxidant activity of a selected set of phenols which have previously been investigated for tertiary antioxidant activity [11]. The f-HAT and SET scavenging of ^•^OOH and ^•^OOCH_3_ radicals by twenty phenolic compounds of various families was evaluated. Thermodynamic and kinetic quantities were calculated at the M06-2X(SMD)/6-31++G(d,p) level of theory at physiological pH and 298.15 K in water and PE to mimic hydrophobic and hydrophilic cellular environments.

Peroxyl radicals scavenging via the SET reaction mechanism in an aqueous environment proved to be the most efficient mode of primary antioxidant action for the phenols, except for molecules **9**, **10** and **11**, which also exhibited biologically significant activity via f-HAT reactions in water (and **10** also in PE). About half of the phenols (12 of 20 for ^•^OOH and 9 of 20 for ^•^OOCH_3_) gave SET rate constants within the diffusion limit, indicating biochemical significance. SET rate constants for ^•^OOH scavenging were consistently greater than those of ^•^OOCH_3_, whereas a few phenols showed greater f-HAT scavenging of ^•^OOCH_3_ in certain microenvironments. The phenols studied are unlikely to engage in protein repair via SET reactions (unless the amino acid to be repaired contains heteroatoms); however, their primary versus tertiary antioxidant activities [11] (in terms of f-HAT reactions) show that generally, the polyphenolic compounds are more efficient at protein repair (in PE) than ^•^OOH or ^•^OOCH_3_ scavenging. On the other hand, the monophenolic compounds consistently showed greater primary antioxidant behaviour than the polyphenolic ones. Moreover, while the phenols showed greater protein repair abilities in a hydrophobic environment, the microenvironment in which they produced superior free radical scavenging abilities was variable, depending on the phenol and peroxyl radical species of focus.

The trends for rate constants in solution were also compared to antioxidant activities predicted using gas-phase thermodynamic properties (O-H bond dissociation energies, BDE, and ionization potentials, IP) by Wright et al. [15]. While several contradictions between BDE and f-HAT rate constants were found, the trends were more similar than for IP and SET rate constants. As the SET reactions studied were largely diffusion controlled, the structural aspects which determine IP are not reflected by the kinetic results. The Gibbs free energies of reaction were, in general, a good predictor of the kinetic trends. However, numerous discrepancies were identified within polyphenols, between phenols, and between the solvents considered, all of which contribute to the unreliability of the Bell–Evans–Polanyi principle (which seems to work when comparing reactions involving species with high structural similarity). While theoretical determination of thermodynamic properties can serve as a useful screening tool, these results contribute to the notion that the calculation of kinetic properties provides more biochemically relevant insight into the antioxidant activity of phenols.

In essence, this work contributes to the understanding of the antioxidant behaviour of these phenolic compounds in the context of peroxyl radical scavenging which may help to prevent (^•^OOH) or stop (^•^OOCH_3_) lipid peroxidation of biological membranes. Through this study, and the study of their ability to repair an oxidatively damaged protein model [11], their primary and tertiary antioxidant character have been defined.

## Figures and Tables

**Table 1:** Standard Gibbs free energies of reaction (∆G°) and activation (∆G^≠^) in kcal/mol and rate constants (k) in M^−1^s^−1^ for f-HAT reactions with ^•^OOH in water and pentyl ethanoate (PE) at 298.15 K.

	∆G°		∆G^≠^		k		Total k		∆BDE ^c^
Reactants	Water	PE	Water	PE	Water	PE	Water	PE	Gas
^•^OOH + **1**	−8.9	−6.3	9.8	9.1	3.64 × 10^6^	5.08 × 10^6^			−8.9
^•^OOH + **2**	−6.7	−4.6	12.4	10.0	4.99 × 10^5^	3.84 × 10^6^			−6.1
^•^OOH + **3**	−6.9	−4.9	11.7	10.0	1.45 × 10^6^	3.78 × 10^6^			−7.2
^•^OOH + **4**	−7.7	−5.3	11.9	9.9	1.46 × 10^6^	4.00 × 10^6^			−7.3
^•^OOH + **5**	−9.3	−6.9	10.1	9.1	4.51 × 10^6^	5.41 × 10^6^			−9.4
^•^OOH + **6**	−9.2	−6.7	10.5	9.3	2.88 × 10^6^	5.20 × 10^6^			−8.9
^•^OOH + **7**	−11.6	−9.0	8.2	7.8	2.71 × 10^7^	2.93 × 10^7^			−11.3
^•^OOH + **8**	−12.9	−9.2	8.8	8.5	3.44 × 10^7^	1.71 × 10^7^			−9.4
^•^OOH + **9**	−14.4	−10.2		7.7	9.54 × 10^7 d^	4.44 × 10^7^			−10.3
^•^OOH + **10**	−19.1	−14.3		5.1	1.49 × 10^9 d^	9.10 × 10^8 a^			−14.6
^•^OOH + **11**	−18.8	−13.2		6.3	1.31 × 10^8 d^	1.47 × 10^8 a^			−11.8
^•^OOH + **12**	−4.9	−2.6	13.9	13.2	3.98 × 10^5^	1.56 × 10^5^			−7.7
^•^OOH + **13**	−3.8	−2.2	14.4	13.3	1.87 × 10^5^	1.41 × 10^5^			−8.5
^•^OOH + **14^(1)^**	−2.3	−1.3	15.7	13.2	4.26 × 10^3^	7.63 × 10^4^	9.38 × 10^4^	1.39 × 10^5^	−11.2
^•^OOH + **14^(2)^**	−6.6	−7.3	13.2	13.6	8.43 × 10^4^	6.05 × 10^4^			
^•^OOH + **14^(3)^**	−2.7	−1.7	15.8	15.8	5.20 × 10^3^	2.50 × 10^3^			
^•^OOH + **15^(1)^**	−8.3	−7.2	12.1	10.9	3.42 × 10^5^	6.75 × 10^5^	2.11 × 10^6^	1.70 × 10^6^	−11.1
^•^OOH + **15^(2)^**	−7.3	−6.3	12.3	13.9	5.08 × 10^5^	3.05 × 10^4^			
^•^OOH + **15^(3)^**	−7.5	−6.4	12.4	13.8	5.36 × 10^5^	4.53 × 10^4^			
^•^OOH + **15^(4)^**	−8.1	−8.9	11.5	10.7	7.26 × 10^5^	9.45 × 10^5^			
^•^OOH + **16^(1)^**	−0.9	0.6	15.7	13.8	3.61 × 10^4^	3.94 × 10^4^	4.49 × 10^4^	5.20 × 10^4^	−2.3
^•^OOH + **16^(2)^**	−0.3	1.0	15.6	14.0	8.79 × 10^3^	1.26 × 10^4^			−1.3
^•^OOH + **17^(1)^**	−9.4	−9.4	11.9	15.8	3.11 × 10^5^	2.58 × 10^3^	5.16 × 10^5^	2.67 × 10^4^	−14.1
^•^OOH + **17^(2)^**	−4.1	−3.4	14.3	15.2	2.05 × 10^5 b^	2.41 × 10^4 b^			
^•^OOH + **18^(1)^**	−1.4	−1.3	14.7	13.2	2.10 × 10^4^	8.06 × 10^4^	1.17 × 10^5^	2.01 × 10^5^	−11.6
^•^OOH + **18^(2)^**	−6.4	−7.3	13.2	13.2	8.74 × 10^4^	1.17 × 10^5^			
^•^OOH + **18^(3)^**	−2.5	−1.8	16.0	16.0	8.74 × 10^3^	3.75 × 10^3^			
^•^OOH + **19^(1)^**	−3.2	−1.5	15.0	13.9	5.64 × 10^4^	5.09 × 10^4^	5.86 × 10^4^	6.09 × 10^4^	−8.2
^•^OOH + **19^(2)^**	1.9	3.1	17.3	14.6	1.22 × 10^3^	8.50 × 10^3^			
^•^OOH + **19^(3)^**	2.0	2.6	17.6	15.8	9.24 × 10^2^	1.55 × 10^3^			
^•^OOH + **20^(1)^**	−6.3	−5.2	13.8	15.0	2.59 × 10^5^	1.35 × 10^4^	1.73 × 10^6^	1.23 × 10^6^	−15.6
^•^OOH + **20^(2)^**	−9.1	−8.4	10.9	10.3	1.46 × 10^6^	1.21 × 10^6^			
^•^OOH + **20^(3)^**	1.0	3.0	17.0	15.0	2.58 × 10^3^	2.16 × 10^3^			
^•^OOH + **20^(4)^**	0.2	2.3	16.5	15.2	7.35 × 10^4^	3.83 × 10^3^			

^a^ These are apparent rate constants (k_app_). ^b^ This value represents the combined rate constant of degenerate sites. ^c^ Bond dissociation enthalpy changes (relative to phenol, in kcal/mol) in the gas phase calculated and reported in [15]. ^d^ These rate constants are estimated using the correlation between log(k) and ∆G° for monophenols (see Figure 2).

**Table 2:** Standard Gibbs free energies of reaction (∆G°) and activation (∆G^≠^) in kcal/mol and rate constants (k) in M^−1^s^−1^ for f-HAT reactions with ^•^OOCH_3_ in water and pentyl ethanoate (PE) at 298.15 K.

	∆G°		∆G^≠^		k		Total k		∆BDE ^c^
Reactants	Water	PE	Water	PE	Water	PE	Water	PE	Gas
^•^OOCH_3_ + **1**	−7.1	−5.1	11.0	11.3	7.82 × 10^5^	4.19 × 10^5^			−8.9
^•^OOCH_3_ + **2**	−5.0	−2.4	12.4	11.9	8.73 × 10^5^	5.17 × 10^5^			−6.1
^•^OOCH_3_ + **3**	−5.1	−2.8	12.4	12.0	1.12 × 10^6^	3.79 × 10^5^			−7.2
^•^OOCH_3_ + **4**	−5.9	−3.1	11.8	11.3	2.56 × 10^6^	1.03 × 10^6^			−7.3
^•^OOCH_3_ + **5**	−7.5	−4.7	9.9	10.4	4.17 × 10^6^	1.35 × 10^6^			−9.4
^•^OOCH_3_ + **6**	−7.4	−4.5	10.5	10.5	3.01 × 10^6^	1.71 × 10^6^			−8.9
^•^OOCH_3_ + **7**	−9.9	−6.8	7.3	8.7	4.85 × 10^7^	7.65 × 10^6^			−11.3
^•^OOCH_3_ + **8**	−11.2	−7.0	8.9	9.6	2.64 × 10^7^	5.05 × 10^6^			−9.4
^•^OOCH_3_ + **9**	−12.7	−8.0		8.3	1.14 × 10^8 d^	2.41 × 10^7^			−10.3
^•^OOCH_3_ + **10**	−17.3	−12.2		5.7	2.87 × 10^9 d^	4.16 × 10^8 a^			−14.6
^•^OOCH_3_ + **11**	−17.1	−11.1		7.3	1.52 × 10^8 d^	3.10 × 10^7^			−11.8
^•^OOCH_3_ + **12**	−3.2	−0.5	13.9	12.8	2.65 × 10^6^	6.33 × 10^5^			−7.7
^•^OOCH_3_ + **13**	−2.1	−0.1	15.0	13.8	4.81 × 10^5^	1.07 × 10^5^			−8.5
^•^OOCH_3_ + **14^(1)^**	−0.5	0.9	15.4	14.3	6.52 × 10^3^	1.45 × 10^4^	1.15 × 10^5^	1.18 × 10^6^	−11.2
^•^OOCH_3_ + **14^(2)^**	−4.8	−5.1	12.7	10.9	9.54 × 10^4^	1.15 × 10^6^			
^•^OOCH_3_ + **14^(3)^**	−1.0	0.4	15.0	14.3	1.30 × 10^4^	1.36 × 10^4^			
^•^OOCH_3_ + **15^(1)^**	−6.6	−5.0	11.2	10.2	8.07 × 10^5^	2.67 × 10^6^	2.38 × 10^6^	3.84 × 10^6^	−11.1
^•^OOCH_3_ + **15^(2)^**	−5.5	−4.1	11.5	12.3	6.99 × 10^5^	1.19 × 10^5^			
^•^OOCH_3_ + **15^(3)^**	−5.7	−4.2	12.4	11.6	1.87 × 10^5^	3.84 × 10^5^			
^•^OOCH_3_ + **15^(4)^**	−6.4	−6.7	11.2	11.0	6.85 × 10^5^	6.66 × 10^5^			
^•^OOCH_3_ + **16^(1)^**	0.8	2.8	15.6	14.8	2.24 × 10^4^	7.94 × 10^3^	2.62 × 10^4^	1.17 × 10^4^	−2.3
^•^OOCH_3_ + **16^(2)^**	1.4	3.2	16.0	14.5	3.79 × 10^3^	3.80 × 10^3^			−1.3
^•^OOCH_3_ + **17^(1)^**	−7.7	−7.2	10.0	9.7	1.67 × 10^6^	2.24 × 10^6^	1.92 × 10^6^	2.46 × 10^6^	−14.1
^•^OOCH_3_ + **17^(2)^**	−2.3	−1.2	13.2	12.9	2.48 × 10^5 b^	2.18 × 10^5 b^			
^•^OOCH_3_ + **18^(1)^**	0.3	0.9	14.8	14.3	1.83 × 10^4^	1.46 × 10^4^	3.34 × 10^5^	7.10 × 10^5^	−11.6
^•^OOCH_3_ + **18^(2)^**	−4.6	−5.2	12.0	11.2	2.85 × 10^5^	6.76 × 10^5^			
^•^OOCH_3_ + **18^(3)^**	−0.7	0.4	14.4	14.1	3.07 × 10^4^	1.94 × 10^4^			
^•^OOCH_3_ + **19^(1)^**	−1.5	0.7	16.3	14.6	8.23 × 10^4^	2.87 × 10^4^	8.25 × 10^4^	2.89 × 10^4^	−8.2
^•^OOCH_3_ + **19^(2)^**	3.6	5.2	17.7	16.5	1.38 × 10^2^	1.29 × 10^2^			
^•^OOCH_3_ + **19^(3)^**	3.7	4.8	18.4	16.9	1.01 × 10^2^	1.19 × 10^2^			
^•^OOCH_3_ + **20^(1)^**	−4.6	−3.0	13.1	12.1	1.42 × 10^5^	2.45 × 10^5^	4.25 × 10^5^	5.57 × 10^5^	−15.6
^•^OOCH_3_ + **20^(2)^**	−7.3	−6.2	12.0	11.4	2.80 × 10^5^	3.12 × 10^5^			
^•^OOCH_3_ + **20^(3)^**	2.7	5.1	16.8	16.5	7.23 × 10^2^	1.57 × 10^2^			
^•^OOCH_3_ + **20^(4)^**	2.0	4.5	16.7	16.8	1.71 × 10^3^	1.46 × 10^2^			

^a^ These are apparent rate constants (k_app_). ^b^ This value represents the combined rate constant of degenerate sites. ^c^ Bond dissociation enthalpy changes (relative to phenol, in kcal/mol) in the gas phase calculated and reported in [15]. ^d^ These rate constants are estimated using the correlation between log(k) in water versus log(k) in PE for monophenols (see Figure 3).

**Table 3:** Standard Gibbs free energies of reaction (∆G°) and activation (∆G^≠^) in kcal/mol, and rate constants (k_SET_, *k_SET_ in M^−1^s^−1^) for SET reactions with ^•^OOH and ^•^OOCH_3_.^a,b.^

Radical	^•^OOH				^•^OOCH_3_				
Phenol	∆G°_SET_	∆G^≠^_SET_	k_SET_	*k_SET_^c^	∆G°_SET_	∆G^≠^_SET_	k_SET_	*k_SET_^c^	∆IP ^d^
**1**	−6.5	1.4	2.83 × 10^8^	5.29 × 10^9^	−4.8	1.8	1.44 × 10^8^	3.38 × 10^8^	−23.0
**2**	−4.6	2.0	9.99 × 10^7^	2.53 × 10^9^	−2.9	2.5	4.34 × 10^7^	1.44 × 10^8^	−23.4
**3**	−4.4	2.2	7.87 × 10^7^	2.12 × 10^8^	−2.7	2.7	3.29 × 10^7^	1.18 × 10^8^	−27.4
**4**	−5.5	1.8	9.65 × 10^7^	2.16 × 10^8^	−3.8	2.3	4.43 × 10^7^	1.28 × 10^8^	−30.5
**5**	−8.0	1.1	1.88 × 10^8^	3.05 × 10^8^	−6.3	1.4	1.06 × 10^8^	2.05 × 10^8^	−33.6
**6**	−8.0	1.2	1.59 × 10^8^	2.66 × 10^8^	−6.3	1.5	8.86 × 10^7^	1.76 × 10^8^	−32.9
**7**	−10.6	0.7	2.31 × 10^8^	3.09 × 10^8^	−8.9	1.0	1.50 × 10^8^	2.28 × 10^8^	−36.1
**8**	−10.9	0.9	6.17 × 10^8^	8.54 × 10^8^	−9.2	1.2	3.95 × 10^8^	6.23 × 10^8^	−29.1
**9**	−12.1	1.3	3.66 × 10^8^	5.71 × 10^8^	−10.3	1.7	2.19 × 10^8^	3.89 × 10^8^	−37.5
**10**	−18.3	0.0	4.26 × 10^8^	4.33 × 10^8^	−16.6	0.1	3.84 × 10^8^	4.00 × 10^8^	−46.9
**11**	−17.5	0.2	8.55 × 10^8^	9.23 × 10^8^	−15.8	0.4	6.97 × 10^8^	7.90 × 10^8^	−48.7
**12**	1.5	4.2	9.79 × 10^7^	7.80 × 10^8^	3.2	5.0	2.31 × 10^7^	3.35 × 10^8^	−27.8
**13**	1.3	4.1	8.32 × 10^7^	6.47 × 10^8^	3.0	5.0	2.00 × 10^7^	2.78 × 10^8^	−32.5
**14^(1)^**	7.5	8.6	1.01 × 10^6^	5.59 × 10^7^	9.2	9.9	1.31 × 10^5^	1.47 × 10^7^	−7.8
**14^(2)^**	6.8	8.2	5.05 × 10^7^	1.70 × 10^9^	8.5	9.3	7.24 × 10^4^	5.96 × 10^6^	
**14^(3)^**	6.7	8.2	2.03 × 10^6^	8.31 × 10^7^	8.5	9.4	2.97 × 10^5^	2.32 × 10^7^	
**15^(1)^**	−1.0	3.4	7.98 × 10^8^	2.90 × 10^9^	0.7	4.1	2.66 × 10^8^	1.64 × 10^9^	−15.1
**15^(2)^**	−0.1	4.0	1.39 × 10^8^	8.11 × 10^8^	1.6	4.7	4.07 × 10^7^	3.70 × 10^8^	
**15^(3)^**	−0.4	3.9	1.55 × 10^8^	8.55 × 10^8^	1.3	4.7	4.67 × 10^7^	3.96 × 10^8^	
**15^(4)^**	−1.3	3.4	5.91 × 10^8 a^	2.25 × 10^9^	0.4	4.1	1.99 × 10^8^	1.22 × 10^9^	
**16^(1)^**	3.3	5.7	2.52 × 10^6^	3.88 × 10^7^	5.1	6.6	5.03 × 10^5^	1.36 × 10^7^	−18.6
**16^(2)^**	4.7	6.4	7.32 × 10^5^	1.74 × 10^7^	6.4	7.5	1.22 × 10^5^	5.55 × 10^6^	
**17^(1)^**	0.6	4.3	2.82 × 10^9^	6.31 × 10^9^	2.3	5.1	1.18 × 10^7^	1.34 × 10^8^	−10.9
**17^(2)^**	3.8	6.0	2.54 × 10^6 e^	8.86 × 10^7 e^	5.5	7.0	9.74 × 10^5 e^	3.01 × 10^7 e^	
**18^(1)^**	8.2	9.0	5.21 × 10^5^	3.92 × 10^7^	9.9	10.3	6.00 × 10^4^	9.78 × 10^6^	−7.2
**18^(2)^**	7.3	8.5	3.01 × 10^7^	1.51 × 10^9^	9.1	9.7	3.93 × 10^4^	4.29 × 10^6^	
**18^(3)^**	7.4	8.6	1.03 × 10^6^	5.53 × 10^7^	9.2	9.8	1.36 × 10^5^	1.47 × 10^7^	
**19^(1)^**	1.8	4.4	6.57 × 10^7^	5.92 × 10^8^	3.5	5.3	1.49 × 10^7^	2.45 × 10^8^	−33.1
**19^(2)^**	6.9	7.8	1.59 × 10^5^	8.77 × 10^6^	8.6	9.0	1.93 × 10^4^	2.38 × 10^6^	
**19^(3)^**	8.6	8.8	2.50 × 10^4^	3.65 × 10^6^	10.3	10.3	1.99 × 10^3^	8.46 × 10^5^	
**20^(1)^**	1.6	4.8	1.29 × 10^8^	1.09 × 10^9^	3.3	5.7	3.16 × 10^7^	4.59 × 10^8^	−35.2
**20^(2)^**	−0.4	3.5	2.00 × 10^9^	5.27 × 10^9^	1.3	4.2	7.07 × 10^8^	3.63 × 10^9^	
**20^(3)^**	6.2	7.4	1.40 × 10^5^	5.59 × 10^6^	8.0	8.6	1.94 × 10^4^	1.60 × 10^6^	
**20^(4)^**	6.7	7.7	1.21 × 10^5^	6.14 × 10^6^	8.5	8.9	1.52 × 10^4^	1.69 × 10^6^	

^a^ k_SET_ and *k_SET_ values greater than 10^8^ are apparent rate constants (k_app_). ^b^ The rate constants are multiplied by the molar fractions of the anionic phenols at pH 7.4 using pK_a_ predictions from [44] which are displayed in Table S12. ^c^ A correction of −6.1 kcal/mol is applied to ∆G°_SET_; ^d^ Ionization potential changes (kcal/mol) in the gas phase calculated and reported in [15]. ^e^ This value represents the combined rate constant of degenerate sites.

**Table 4:** Summary of the primary antioxidant activity of the phenols studied relative to reactions with ^•^OOH and ^•^OOCH_3_.

Structural Motif	Phenolic Compounds	Dominant f-HAT Solvent		Dominant Mechanism(s)		Biochemical Relevance	
Peroxyl Radical:		^•^OOH	^•^OOCH_3_	^•^OOH	^•^OOCH_3_	^•^OOH	^•^OOCH_3_
*ortho-*alkyl group(s)	**1**, **2**, **5**, **6**, **7**, **10**	variable	water	see below		see below	see below
*para* ether	**1** to **7**	PE	water	SET		**1–2**, **5–7** by SET	**1**, **5**, **6** by SET
*para*-amino	**8** to **11**	water	water	SET (**8**, **9**, **11**), f-HAT in water (**10**)		**8–11** by SET, **9** (W), **10–11** (W and PE) by f-HAT	**8–11** by SET, **9** and **11** (W), **10** (W and PE) by f-HAT
*para*-conjugation	**12**, **13**, **19^(1)^**^,^ **20^(2)^**	water	variable	SET		**20^(2)^** by SET	**20^(2)^** by SET
*para*-alkyl group	**15^(1,4)^**	PE	variable	SET		by SET	by SET
*para*-ester	**14^(2)^**, **18^(2)^**	variable	PE	SET		no	no
*ortho*-hydroxyl(s)	**14^(1–3)^**, **15^(1–4)^**, **17^(1,2)^**, **18^(1–3)^**, **20^(1,2)^**	variable	variable	SET		**15^(1–4)^**, **17^(1)^**, **20^(1,2)^** by SET	**15(^1,4)^**, **20^(2)^** by SET
*meta*-hydroxyl	**16^(1,2)^**, **19^(2,3)^**, **20^(3,4)^**	variable	variable	SET		no	no

## Data Availability

The original contributions presented in this study are included in the article/Supplementary Material. Further inquiries can be directed to the corresponding author(s).

## Supplementary Materials

The following supporting information can be downloaded at https://www.mdpi.com/article/10.3390/antiox15070868/s1, Names and corresponding labels of the studied phenols with structures shown in Figure 1 (Table S1); <S^2^> values of doublet systems involving ^•^OOH before and after spin annihilation in water and PE (Table S2); <S^2^> values of doublet systems involving ^•^OOCH_3_ before and after spin annihilation in water and PE (Table S3); Standard absolute energies, enthalpies, and Gibbs free energies (in atomic units) at 298.15 K for reactants, products, and transition states between the ^•^OOH radical and the phenolic antioxidants of interest in water at the M06-2X(SMD)/6-31++G(d,p) level of theory (Table S4); Standard absolute energies, enthalpies, and Gibbs free energies (in atomic units) at 298.15 K for reactants, products, and transition states between the ^•^OOH radical and the phenolic antioxidants of interest in PE at the M06-2X(SMD)/6-31++G(d,p) level of theory (Table S5); Standard absolute energies, enthalpies, and Gibbs free energies (in atomic units) at 298.15 K for reactants, products, and transition states between the ^•^OOCH_3_ radical and the phenolic antioxidants of interest in water at the M06-2X(SMD)/6-31++G(d,p) level of theory (Table S6); Standard absolute energies, enthalpies, and Gibbs free energies (in atomic units) at 298.15 K for reactants, products, and transition states between the ^•^OOCH_3_ radical and the phenolic antioxidants of interest in PE at the M06-2X(SMD)/6-31++G(d,p) level of theory (Table S7); Standard enthalpies of reaction (∆H°) and activation (∆H^≠^) in kcal/mol, imaginary vibrational frequencies (v^≠^) in cm-1, and tunelling factors (κ), at 298.15 K in water for the f-HAT reactions between phenols and ^•^OOH at the M06-2X(SMD)/6-31++G(d,p) level of theory (Table S8); Standard enthalpies of reaction (∆H°) and activation (∆H^≠^) in kcal/mol, imaginary vibrational frequencies (v^≠^) in cm-1, and tunelling factors (κ), at 298.15 K in PE for the f-HAT reactions between phenols and ^•^OOH at the M06-2X(SMD)/6-31++G(d,p) level of theory (Table S9); Standard enthalpies of reaction (∆H°) and activation (∆H^≠^) in kcal/mol, imaginary vibrational frequencies (v^≠^) in cm^−1^, and tunelling factors (κ), at 298.15 K in water for the f-HAT reactions between phenols and ^•^OOCH_3_ at the M06-2X(SMD)/6-31++G(d,p) level of theory (Table S10); Standard enthalpies of reaction (∆H°) and activation (∆H^≠^) in kcal/mol, imaginary vibrational frequencies (v^≠^) in cm^−1^, and tunelling factors (κ), at 298.15 K in PE for the f-HAT reactions between phenols and ^•^OOCH_3_ at the M06-2X(SMD)/6-31++G(d,p) level of theory (Table S11); Standard Gibbs free energies (G°) in au for neutral and anion forms of phenols at the M06 2X(PCM)/6-311++G(d,p) level of theory in water, molar fractions and predicted pK_a_ values for the polyphenolic antioxidants (Table S12); Plot of log(k) in water versus log(k) in PE for f-HAT reactions of monophenols with ^•^OOH at the M06-2X(SMD)/6-31++G(d,p) level of theory (Figure S1); Plot of log(k) versus the ∆G° of f-HAT reactions of monophenols with ^•^OOCH3 at the M06 2X(SMD)/6-31++G(d,p) level of theory in water (Figure S2); Cartesian coordinates of the optimized transition states between a phenol and ^•^OOH studied at the M06-2X(SMD)/6-31++G(d,p) level of theory in water and PE; Cartesian coordinates of the optimized transition states between a phenol and ^•^OOCH3 studied at the M06-2X(SMD)/6-31++G(d,p) level of theory in water and PE.
