# Supplementary material for: Computational Study of the Peroxyl Radical Scavenging Ability of Phenolic Antioxidants
Source: Antioxidants (Basel). 2026 Jul 11;15(7):868. doi: 10.3390/antiox15070868 (PMC13405240; doi:10.3390/antiox15070868)
Supplement: Supplementary file 1 [file antioxidants-15-00868-s001.zip › antioxidants-4369350-supplementary.pdf]

Computational study of the peroxyl radical scavenging ability of phenolic antioxidants

Ainsley Barisoff,[1] Max Walton-Raaby,[2] Paula Jofily,[2] Nelaine Mora-Diez [1,*]

[1]Department of Chemistry, Thompson Rivers University, Kamloops, BC V2C 0C8, Canada

[2]Department of Chemistry, University of Waterloo, Waterloo, ON N2L 3G1, Canada

Electronic Supplementary Information

(109 pages)

Contents:

Table S1. Names and corresponding labels of the studied phenols with structures shown in Figure 1.

Table S2. <S^2> values of doublet systems involving •OOH before and after spin annihilation in water and PE.

Table S3. <S^2> values of doublet systems involving $^{\bullet}OOCH_3$ before and after spin annihilation in water and PE.

Table S4. Standard absolute energies, enthalpies, and Gibbs free energies (in atomic units) at 298.15 K for reactants, products, and transition states between the •OOH radical and the phenolic antioxidants of interest in water at the M06-2X(SMD)/6-31++G(d,p) level of theory.

Table S5. Standard absolute energies, enthalpies, and Gibbs free energies (in atomic units) at 298.15 K for reactants, products, and transition states between the •OOH radical and the phenolic antioxidants of interest in PE at the M06-2X(SMD)/6-31++G(d,p) level of theory.

Table S6. Standard absolute energies, enthalpies, and Gibbs free energies (in atomic units) at 298.15 K for reactants, products, and transition states between the $^{\bullet}OOCH_3$ radical and the phenolic antioxidants of interest in water at the M06-2X(SMD)/6-31++G(d,p) level of theory.

Table S7. Standard absolute energies, enthalpies, and Gibbs free energies (in atomic units) at 298.15 K for reactants, products, and transition states between the $^{\bullet}OOCH_3$ radical and the phenolic antioxidants of interest in PE at the M06-2X(SMD)/6-31++G(d,p) level of theory.

Table S8. Standard enthalpies of reaction ($\Delta H°$) and activation ($\Delta H^{\neq}$) in kcal/mol, imaginary vibrational frequencies ($\nu^{\neq}$) in cm^{-1}, and tunelling factors (κ), at 298.15 K in water for the f-HAT reactions between phenols and •OOH at the M06-2X(SMD)/6-31++G(d,p) level of theory.

Table S9. Standard enthalpies of reaction ($\Delta H°$) and activation ($\Delta H^{\neq}$) in kcal/mol, imaginary vibrational frequencies ($\nu^{\neq}$) in cm^{-1}, and tunelling factors (κ), at 298.15 K in PE for the f-HAT reactions between phenols and •OOH at the M06-2X(SMD)/6-31++G(d,p) level of theory.

Table S10. Standard enthalpies of reaction ($\Delta H°$) and activation ($\Delta H^{\neq}$) in kcal/mol, imaginary vibrational frequencies ($\nu^{\neq}$) in cm^{-1}, and tunelling factors (κ), at 298.15 K in water for the f-HAT reactions between phenols and $^{\bullet}OOCH_3$ at the M06-2X(SMD)/6-31++G(d,p) level of theory.

Table S11. Standard enthalpies of reaction (ΔH°) and activation (ΔH≠) in kcal/mol, imaginary vibrational frequencies (v≠) in cm^{-1}, and tunelling factors (κ), at 298.15 K in PE for the f-HAT reactions between phenols and $^{\bullet}OOCH_3$ at the M06-2X(SMD)/6-31++G(d,p) level of theory.

Table S12. Standard Gibbs free energies (G°) in au for neutral and anion forms of phenols at the M06-2X(PCM)/6-311++G(d,p) level of theory in water, molar fractions and predicted pK_a values for the polyphenolic antioxidants.

Figure S1. Plot of log(k) in water versus log(k) in PE for f-HAT reactions of monophenols with $^{\bullet}OOH$ at the M06-2X(SMD)/6-31++G(d,p) level of theory.

Figure S2. Plot of log(k) versus the ΔG° of f-HAT reactions of monophenols with $^{\bullet}OOCH_3$ at the M06-2X(SMD)/6-31++G(d,p) level of theory in water.

Cartesian coordinates of the optimized transition states between a phenol and $^{\bullet}OOH$ studied at the M06-2X(SMD)/6-31++G(d,p) level of theory in water and PE.

Cartesian coordinates of the optimized transition states between a phenol and $^{\bullet}OOCH_3$ studied at the M06-2X(SMD)/6-31++G(d,p) level of theory in water and PE.

Table S1. Names and corresponding labels of the studied phenols with structures shown in Figure 1.

Number	Name
1	ortho-butylated hydroxyanisole (oBHA)
2	meta-butylated hydroxyanisole (mBHA)
3	tocol
4	δ-tocopherol
5	β-tocopherol
6	γ-tocopherol
7	α-tocopherol
8	4-aminophenol
9	N,N-dimethyl-4-aminophenol
10	6-hydroxy-5,7,8-trimethyl-1,2,3,4-tetrahydroquinoline
11	9-hydroxyjulolidine
12	4-butadienylphenol
13	4-vinylphenylphenol
14	propyl gallate
15	nordihydroguaiaretic acid (NDGA)
16	epigallocatechin-3-gallate ring 1 (EGCG1)
17	epigallocatechin-3-gallate ring 2 (EGCG2)
18	epigallocatechin-3-gallate ring 3 (EGCG3)
19	trans-resveratrol
20	piceatannol

Table S2. <S^2> values of doublet systems involving $^{\bullet}$OOH before and after spin annihilation in water and PE.

	Water		PE	
**Transition state**	**<S2> before**	**<S2> after**	**<S2> before**	**<S2> after**
1-TS	0.764	0.750	0.765	0.750
2-TS	0.764	0.750	0.766	0.750
3-TS	0.765	0.750	0.766	0.750
4-TS	0.764	0.750	0.766	0.750
5-TS	0.763	0.750	0.764	0.750
6-TS	0.763	0.750	0.764	0.750
7-TS	0.762	0.750	0.763	0.750
8-TS	0.763	0.750	0.765	0.750
9-TS			0.764	0.750
10-TS			0.763	0.750
11-TS			0.765	0.750
12-TS	0.774	0.750	0.774	0.750
13-TS	0.772	0.750	0.772	0.750
14(1)-TS	0.764	0.750	0.766	0.750
14(2)-TS	0.765	0.750	0.765	0.750
14(3)-TS	0.764	0.750	0.765	0.750
15(1)-TS	0.763	0.750	0.764	0.750
15(2)-TS	0.762	0.750	0.765	0.750
15(3)-TS	0.762	0.750	0.765	0.750
15(4)-TS	0.763	0.750	0.764	0.750
16(1)-TS	0.767	0.750	0.768	0.750
16(2)-TS	0.767	0.750	0.768	0.750
17(1)-TS	0.763	0.750	0.764	0.750
17(2)-TS	0.763	0.750	0.765	0.750
18(1)-TS	0.764	0.750	0.766	0.750
18(2)-TS	0.765	0.750	0.765	0.750
18(3)-TS	0.764	0.750	0.766	0.750
19(1)-TS	0.772	0.750	0.773	0.750
19(2)-TS	0.768	0.750	0.771	0.750
19(3)-TS	0.770	0.750	0.770	0.750
20(1)-TS	0.763	0.750	0.766	0.750
20(2)-TS	0.767	0.750	0.767	0.750
20(3)-TS	0.767	0.750	0.768	0.750
20(4)-TS	0.770	0.750	0.772	0.750

Table S3. $\langle S^2 \rangle$ values of doublet systems involving $^{\bullet}OOCH_3$ before and after spin annihilation in water and PE.

	Water		PE	
**Transition state**	**<S2> before**	**<S2> after**	**<S2> before**	**<S2> after**
1-TS	0.764	0.750	0.766	0.750
2-TS	0.765	0.750	0.767	0.750
3-TS	0.765	0.750	0.766	0.750
4-TS	0.765	0.750	0.766	0.750
5-TS	0.763	0.750	0.765	0.750
6-TS	0.764	0.750	0.765	0.750
7-TS	0.763	0.750	0.764	0.750
8-TS	0.764	0.750	0.765	0.750
9-TS			0.765	0.750
10-TS			0.763	0.750
11-TS			0.765	0.750
12-TS	0.776	0.750	0.777	0.751
13-TS	0.773	0.750	0.775	0.750
14(1)-TS	0.763	0.750	0.764	0.750
14(2)-TS	0.765	0.750	0.765	0.750
14(3)-TS	0.763	0.750	0.764	0.750
15(1)-TS	0.762	0.750	0.763	0.750
15(2)-TS	0.763	0.750	0.764	0.750
15(3)-TS	0.763	0.750	0.764	0.750
15(4)-TS	0.762	0.750	0.763	0.750
16(1)-TS	0.764	0.750	0.766	0.750
16(2)-TS	0.765	0.750	0.767	0.750
17(1)-TS	0.763	0.750	0.763	0.750
17(2)-TS	0.762	0.750	0.764	0.750
18(1)-TS	0.765	0.750	0.766	0.750
18(2)-TS	0.765	0.750	0.765	0.750
18(3)-TS	0.765	0.750	0.766	0.750
19(1)-TS	0.773	0.750	0.773	0.750
19(2)-TS	0.764	0.750	0.767	0.750
19(3)-TS	0.766	0.750	0.768	0.750
20(1)-TS	0.764	0.750	0.765	0.750
20(2)-TS	0.766	0.750	0.767	0.750
20(3)-TS	0.764	0.750	0.767	0.750
20(4)-TS	0.767	0.750	0.768	0.750

Table S4. Standard absolute energies, enthalpies, and Gibbs free energies (in atomic units) at 298.15 K for reactants, products, and transition states between the •OOH radical and the phenolic antioxidants of interest in water at the M06-2X(SMD)/6-31++G(d,p) level of theory.

Species	E(SCF) (au)	E° (au)	H° (au)	G° (au)
H_2O_2	-151.50553	-151.47545	-151.47450	-151.50098
OOH-rad	-150.85862	-150.84113	-150.84019	-150.86613
OOH anion	-151.02368	-151.00701	-151.00607	-151.03162
1	-579.02644	-578.76181	-578.76087	-578.81509
1-rad	-578.39430	-578.14213	-578.14118	-578.19434
1 anion	-578.54931	-578.29795	-578.29701	-578.34946
2	-579.02868	-578.76414	-578.7632	-578.81656
2-rad	-578.39249	-578.14008	-578.13913	-578.19239
2 anion	-578.54999	-578.29870	-578.29776	-578.35056
3	-577.85546	-577.61366	-577.61271	-577.66178
3-rad	-577.21905	-576.98953	-576.98858	-577.03785
3 anion	-577.37730	-577.14889	-577.14795	-577.19634
4	-617.15480	-616.88353	-616.88259	-616.93513
4-rad	-616.51971	-616.26070	-616.25975	-616.31248
4 anion	-616.67582	-616.41806	-616.41712	-616.46915
5	-656.45287	-656.15195	-656.15101	-656.20732
5-rad	-655.82016	-655.53173	-655.53078	-655.58723
5 anion	-655.97238	-655.68510	-655.68416	-655.73995
6	-656.45096	-656.15004	-656.14909	-656.20594
6-rad	-655.81789	-655.52946	-655.52851	-655.58572
6 anion	-655.97061	-655.68325	-655.68231	-655.73838
7	-695.74767	-695.41724	-695.41630	-695.47648
7-rad	-695.11779	-694.79985	-694.79890	-694.86010
7 anion	-695.26679	-694.94980	-694.94885	-695.00867
8	-362.70865	-362.57997	-362.57903	-362.61766
8-rad	-362.08167	-361.96541	-361.96446	-362.00341
8 anion	-362.22907	-362.11393	-362.11298	-362.15145
9	-441.27806	-441.08930	-441.08836	-441.13328
9-rad	-440.65307	-440.47629	-440.47535	-440.52145
9 anion	-440.80018	-440.62495	-440.62401	-440.66770
10	-597.28616	-597.00122	-597.00028	-597.05559
10-rad	-596.66958	-596.39665	-596.39570	-596.45110
10 anion	-596.80575	-596.53415	-596.53321	-596.58742
11	-596.08465	-595.82090	-595.81996	-595.86921
11-rad	-595.46789	-595.21602	-595.21508	-595.26437
11 anion	-595.60397	-595.35383	-595.35288	-595.40191
12	-462.10650	-461.92433	-461.92338	-461.97114
12-rad	-461.46711	-461.29796	-461.29702	-461.34411
12 anion	-461.63357	-461.46520	-461.46426	-461.51191

13	-615.71103	-615.47813	-615.47719	-615.53216
13-rad	-615.07141	-614.85107	-614.85013	-614.90334
13 anion	-615.23799	-615.01853	-615.01758	-615.07089
14	-764.18193	-763.95362	-763.95268	-764.01268
14(1)-rad	-763.53744	-763.32173	-763.32079	-763.38144
14(2)-rad	-763.54517	-763.32961	-763.32867	-763.38830
14(3)-rad	-763.53768	-763.32213	-763.32118	-763.38220
14(1) anion	-763.71569	-763.50058	-763.49964	-763.55888
14(2) anion	-763.72250	-763.50724	-763.50630	-763.56458
14(3) anion	-763.71560	-763.50050	-763.49956	-763.55842
15	-999.73342	-999.34183	-999.34089	-999.41584
15(1)-rad	-999.09931	-998.72033	-998.71939	-998.79424
15(2)-rad	-999.09741	-998.71844	-998.71750	-998.79256
15(3)-rad	-999.09732	-998.71842	-998.71748	-998.79291
15(4)-rad	-999.09908	-998.72011	-998.71917	-998.79390
15(1) anion	-999.26202	-998.88404	-998.88310	-998.95810
15(2) anion	-999.26276	-998.88469	-998.88375	-998.95783
15(3) anion	-999.26271	-998.88470	-998.88375	-998.95777
15(4) anion	-999.26210	-998.88418	-998.88324	-998.95730
16	-574.46540	-574.27646	-574.27551	-574.32168
16(1)-rad	-573.81879	-573.64298	-573.64204	-573.68833
16(2)-rad	-573.81745	-573.64192	-573.64098	-573.68737
16(1) anion	-573.99061	-573.81488	-573.81394	-573.85914
16(2) anion	-573.99140	-573.81587	-573.81492	-573.86035
17	-457.77840	-457.65716	-457.65621	-457.69708
17(1)-rad	-457.14637	-457.03772	-457.03677	-457.07725
17(2)-rad	-457.13809	-457.02918	-457.02823	-457.06872
17(1) anion	-457.31237	-457.20450	-457.20356	-457.24367
17(2) anion	-457.30900	-457.20108	-457.20014	-457.24022
18	-685.58666	-685.41756	-685.41662	-685.46877
18(1)-rad	-684.94220	-684.78557	-684.78463	-684.83617
18(2)-rad	-684.94982	-684.79346	-684.79252	-684.84406
18(3)-rad	-684.94212	-684.78610	-684.78516	-684.83784
18(1) anion	-685.12044	-684.96462	-684.96368	-685.01468
18(2) anion	-685.12719	-684.97139	-684.97044	-685.02125
18(3) anion	-685.12031	-684.96472	-684.96378	-685.01515
19	-766.13071	-765.88764	-765.88670	-765.94755
19(1)-rad	-765.49033	-765.25987	-765.25892	-765.31782
19(2)-rad	-765.48031	-765.25030	-765.24936	-765.30969
19(3)-rad	-765.48088	-765.25093	-765.24998	-765.30958
19(1) anion	-765.65788	-765.42824	-765.42730	-765.48615
19(2) anion	-765.65665	-765.42681	-765.42587	-765.48618
19(3) anion	-765.65685	-765.42713	-765.42619	-765.48873
20	-841.33763	-841.08901	-841.08806	-841.15000
20(1)-rad	-840.70042	-840.46427	-840.46333	-840.52521

20(2)-rad	-840.70562	-840.46954	-840.46860	-840.52957
20(3)-rad	-840.68728	-840.45199	-840.45104	-840.51357
20(4)-rad	-840.68771	-840.45214	-840.45119	-840.51478
20(1) anion	-840.86840	-840.63336	-840.63242	-840.69326
20(2) anion	-840.86985	-840.63473	-840.63378	-840.69439
20(3) anion	-840.86362	-840.62836	-840.62742	-840.68897
20(4) anion	-840.86393	-840.62878	-840.62784	-840.69102
1-TS	-729.87806	-729.59860	-729.59765	-729.65858
2-TS	-729.87510	-729.59564	-729.59469	-729.65590
3-TS	-728.70144	-728.44514	-728.44419	-728.50226
4-TS	-768.00156	-767.71549	-767.71455	-767.77522
5-TS	-807.30259	-806.98702	-806.98607	-807.05035
6-TS	-807.30024	-806.98450	-806.98356	-807.04827
7-TS	-846.59967	-846.25427	-846.25333	-846.32239
8-TS	-513.55862	-513.41534	-513.41439	-513.46265
12-TS	-612.94898	-612.75247	-612.75152	-612.80806
13-TS	-766.55366	-766.30641	-766.30547	-766.36827
14(1)-TS	-915.02452	-914.78130	-914.78035	-914.84676
14(2)-TS	-915.02714	-914.78401	-914.78306	-914.85074
14(3)-TS	-915.02351	-914.78042	-914.77948	-914.84651
15(1)-TS	-1150.58064	-1150.17412	-1150.17318	-1150.25566
15(2)-TS[a]	-1150.58052	-1150.17412	-1150.17317	-1150.25532
A-15(3)-TS[a]	-1150.58042	-1150.17390	-1150.17296	-1150.25520
B-15(3)-TS	-1150.25550	-1150.17380	-1150.17280	-1150.25550
15(4)-TS	-1150.58061	-1150.17446	-1150.17352	-1150.25653
16(1)-TS	-725.30559	-725.10219	-725.10125	-725.15577
16(2)-TS	-725.30637	-725.10327	-725.10232	-725.15580
17(1)-TS	-608.62511	-608.48880	-608.48786	-608.53721
A-17(2)-TS[a]	-608.62214	-608.48613	-608.48518	-608.53339
B-17(2)-TS	-608.62186	-608.48589	-608.48495	-608.53380
18(1)-TS	-836.42921	-836.24553	-836.24458	-836.30441
18(2)-TS	-836.43175	-836.24786	-836.24692	-836.30685
A-18(3)-TS[a]	-836.42809	-836.24409	-836.24315	-836.30237
B-18(3)-TS	-836.42814	-836.24424	-836.24329	-836.30257
19(1)-TS	-916.97294	-916.71525	-916.71430	-916.78270
19(2)-TS	-916.96919	-916.71160	-916.71066	-916.77898
19(3)-TS	-916.96862	-916.71130	-916.71036	-916.77854
20(1)-TS[a]	-992.18290	-991.91919	-991.91824	-991.98701
20(2)-TS	-992.18601	-991.92237	-991.92143	-991.99164
20(3)-TS	-992.17573	-991.91310	-991.91215	-991.98204
20(4)-TS	-992.17592	-991.91293	-991.91198	-991.98275

[a]Hydrogen bonding between •OOH and adjacent hydroxyl is present

Table S5. Standard absolute energies, enthalpies, and Gibbs free energies (in atomic units) at 298.15 K for reactants, products, and transition states between the •OOH radical and the phenolic antioxidants of interest in PE at the M06-2X(SMD)/6-31++G(d,p) level of theory.

Species	E° (au)	H° (au)	G° (au)
H_2O_2	-151.46941	-151.46846	-151.49483
OOH-rad	-150.83923	-150.83828	-150.86423
OOH anion	-150.95156	-150.95062	-150.97619
1	-578.76469	-578.76375	-578.81770
1-rad	-578.14631	-578.14536	-578.19875
2	-578.76714	-578.76620	-578.81931
2-rad	-578.14338	-578.14244	-578.19602
3	-577.61528	-577.61434	-577.66344
3-rad	-576.99229	-576.99135	-577.04069
4	-616.88617	-616.88523	-616.93793
4-rad	-616.26389	-616.26295	-616.31578
5	-656.15557	-656.15462	-656.21113
5-rad	-655.53590	-655.53495	-655.59148
6	-656.15394	-656.15300	-656.20985
6-rad	-655.53379	-655.53285	-655.58995
7	-695.42219	-695.42124	-695.48174
7-rad	-694.80537	-694.80442	-694.86543
8	-362.57640	-362.57546	-362.61419
8-rad	-361.95976	-361.95881	-361.99819
9	-441.08816	-441.08721	-441.13275
9-rad	-440.47368	-440.47273	-440.51834
10	-597.00411	-597.00316	-597.05806
10-rad	-596.39631	-596.39537	-596.45030
11	-595.82353	-595.82258	-595.87235
11-rad	-595.21438	-595.21344	-595.26282
12	-461.92719	-461.92625	-461.97336
12-rad	-461.30087	-461.29993	-461.34698
13	-615.48278	-615.48184	-615.53558
13-rad	-614.85624	-614.85530	-614.90853
14	-763.94910	-763.94816	-764.00724
14(1)-rad	-763.32079	-763.31985	-763.37872
14(2)-rad	-763.33050	-763.32956	-763.38827
14(3)-rad	-763.32121	-763.32027	-763.37940
15	-999.33944	-999.33850	-999.41392
15(1)-rad	-998.72090	-998.71996	-998.79478
15(2)-rad	-998.71916	-998.71821	-998.79337
15(3)-rad	-998.71921	-998.71826	-998.79345
15(4)-rad	-998.72058	-998.71963	-998.79749
16	-574.27161	-574.27067	-574.31660

16(1)-rad	-573.63971	-573.63877	-573.68501
16(2)-rad	-573.63882	-573.63788	-573.68439
17	-457.65140	-457.65045	-457.69142
17(1)-rad	-457.03655	-457.03561	-457.07575
17(2)-rad	-457.02661	-457.02566	-457.06622
18	-685.41081	-685.40986	-685.46186
18(1)-rad	-684.78252	-684.78158	-684.83336
18(2)-rad	-684.79222	-684.79128	-684.84295
18(3)-rad	-684.78299	-684.78204	-684.83411
19	-765.88143	-765.88049	-765.94032
19(1)-rad	-765.25435	-765.25341	-765.31212
19(2)-rad	-765.24646	-765.24552	-765.30483
19(3)-rad	-765.24704	-765.24610	-765.30558
20	-841.07990	-841.07896	-841.14092
20(1)-rad	-840.45838	-840.45743	-840.51857
20(2)-rad	-840.46356	-840.46262	-840.52370
20(3)-rad	-840.44508	-840.44414	-840.50559
20(4)-rad	-840.44533	-840.44438	-840.50662
1-TS	-729.60027	-729.59933	-729.66035
2-TS	-729.59751	-729.59657	-729.66053
3-TS	-728.44630	-728.44536	-728.50465
4-TS	-767.71779	-767.71685	-767.77926
5-TS	-806.98952	-806.98858	-807.05374
6-TS	-806.98715	-806.98621	-807.05223
7-TS	-846.25770	-846.25676	-846.32641
8-TS	-513.41019	-513.40925	-513.45785
9-TS	-591.92348	-591.92254	-591.97757
10-TS	-747.84426	-747.84331	-747.90716
11-TS	-746.66182	-746.66088	-746.71953
12-TS	-612.75392	-612.75297	-612.80950
13-TS	-766.30958	-766.30864	-766.37156
14(1)-TS	-914.77668	-914.77573	-914.84339
14(2)-TS[a]	-914.77813	-914.77718	-914.84276
14(3)-TS	-914.77267	-914.77173	-914.83923
15(1)-TS	-1150.17117	-1150.17023	-1150.25363
15(2)-TS[a]	-1150.16793	-1150.16699	-1150.24886
15(3)-TS[a]	-1150.16784	-1150.16689	-1150.24914
15(4)-TS	-1150.17127	-1150.17032	-1150.25398
16(1)-TS	-725.09762	-725.09667	-725.15171
16(2)-TS	-725.09838	-725.09743	-725.15146
A-17(1)-TS[a]	-608.47629	-608.47534	-608.52334
B-17(1)-TS	-608.47520	-608.47426	-608.52352
17(2)-TS[a]	-608.47714	-608.47619	-608.52433
18(1)-TS	-836.23834	-836.23739	-836.29803
18(2)-TS[a]	-836.23979	-836.23885	-836.29795

18(3)-TS[a]	-836.23492	-836.23397	-836.29346
19(1)-TS	-916.70792	-916.70698	-916.77531
19(2)-TS	-916.70555	-916.70460	-916.77418
19(3)-TS	-916.70518	-916.70424	-916.77228
A-20(1)-TS[a]	-991.90631	-991.90537	-991.97425
B-20(1)-TS	-991.90591	-991.90497	-991.97489
20(2)-TS	-991.91278	-991.91184	-991.98163
20(3)-TS	-991.90397	-991.90302	-991.97419
20(4)-TS	-991.90401	-991.90306	-991.97388

[a]Hydrogen bonding between •OOH and an adjacent hydroxyl is present

Table S6. Standard absolute energies, enthalpies, and Gibbs free energies (in atomic units) at 298.15 K for reactants, products, and transition states between the •$OOCH_3$ radical and the phenolic antioxidants of interest in water at the M06-2X(SMD)/6-31++G(d,p) level of theory.

Species	E(SCF) (au)	E° (au)	H° (au)	G° (au)
$HOOCH_3$	-190.78751	-190.72771	-190.72677	-190.75720
$OOCH_3$-rad	-190.14322	-190.09563	-190.09468	-190.12511
$OOCH_3$ anion	-190.30651	-190.25946	-190.25852	-190.28783
1-TS	-769.16177	-768.85261	-768.85167	-768.91556
2-TS	-769.16022	-768.85092	-768.84997	-768.91478
3-TS	-767.98657	-767.70021	-767.69927	-767.76012
4-TS	-807.28677	-806.97088	-806.96994	-807.03441
5-TS	-846.58826	-846.24301	-846.24206	-846.30961
6-TS	-846.58573	-846.24014	-846.23919	-846.30724
7-TS	-885.88588	-885.51086	-885.50992	-885.58292
8-TS	-552.84427	-552.67100	-552.67006	-552.72149
12-TS	-652.23426	-652.00791	-652.00696	-652.06702
13-TS	-805.83887	-805.56159	-805.56065	-805.62627
14(1)-TS	-954.30989	-954.03690	-954.03595	-954.10619
14(2)-TS	-954.31476	-954.04157	-954.04062	-954.11042
14(3)-TS	-954.30998	-954.03722	-954.03628	-954.10687
15(1)-TS	-1189.86742	-1189.43100	-1189.43005	-1189.51603
15(2)-TS	-1189.86634	-1189.43018	-1189.42924	-1189.51549
15(3)-TS	-1189.86654	-1189.42996	-1189.42901	-1189.51412
15(4)-TS	-1189.86745	-1189.43124	-1189.43030	-1189.51599
16(1)-TS	-764.59033	-764.35748	-764.35654	-764.41493
16(2)-TS	-764.59160	-764.35850	-764.35755	-764.41425
17(1)-TS	-647.91488	-647.74864	-647.74770	-647.79910
17(2)-TS	-647.90869	-647.74290	-647.74195	-647.79412
18(1)-TS	-875.71465	-875.50118	-875.50023	-875.56328
18(2)-TS	-875.71930	-875.50582	-875.50487	-875.56763
18(3)-TS	-875.71481	-875.50136	-875.50041	-875.56379
19(1)-TS	-956.25831	-955.97036	-955.96941	-956.03959
19(2)-TS	-956.25495	-955.96756	-955.96661	-956.03744
19(3)-TS	-956.25420	-955.96660	-955.96565	-956.03619
20(1)-TS	-1031.47017	-1031.17645	-1031.17551	-1031.24717
20(2)-TS	-1031.47175	-1031.17817	-1031.17722	-1031.24898
20(3)-TS	-1031.46190	-1031.16876	-1031.16782	-1031.24128
20(4)-TS	-1031.46105	-1031.16841	-1031.16746	-1031.24140

Table S7. Standard absolute energies, enthalpies, and Gibbs free energies (in atomic units) at 298.15 K for reactants, products, and transition states between the $^{\bullet}OOCH_3$ radical and the phenolic antioxidants of interest in PE at the M06-2X(SMD)/6-31++G(d,p) level of theory.

Species	E° (au)	H° (au)	G° (au)
$HOOCH_3$	-190.72512	-190.72417	-190.75465
$OOCH_3$-rad	-190.09801	-190.09707	-190.12750
1-TS	-768.85708	-768.85614	-768.92009
2-TS	-768.85604	-768.85509	-768.92076
3-TS	-767.70457	-767.70362	-767.76476
4-TS	-806.97602	-806.97507	-807.04034
5-TS	-846.24834	-846.24740	-846.31500
6-TS	-846.24588	-846.24494	-846.31355
7-TS	-885.51725	-885.51630	-885.58828
8-TS	-552.66882	-552.66788	-552.71926
9-TS	-631.18229	-631.18135	-631.24000
10-TS	-787.10390	-787.10296	-787.16942
11-TS	-785.91989	-785.91895	-785.98108
12-TS	-652.01287	-652.01192	-652.07339
13-TS	-805.56847	-805.56753	-805.63400
14(1)-TS	-954.03539	-954.03445	-954.10495
14(2)-TS	-954.04052	-954.03957	-954.11036
14(3)-TS	-954.03556	-954.03462	-954.10486
15(1)-TS	-1189.43097	-1189.43003	-1189.51803
15(2)-TS	-1189.42995	-1189.42901	-1189.51477
15(3)-TS	-1189.42986	-1189.42891	-1189.51581
15(4)-TS	-1189.43103	-1189.43009	-1189.51674
16(1)-TS	-764.35541	-764.35446	-764.41352
16(2)-TS	-764.35709	-764.35614	-764.41387
17(1)-TS	-647.74570	-647.74476	-647.79640
17(2)-TS	-647.73960	-647.73865	-647.79135
18(1)-TS	-875.49714	-875.49620	-875.55950
18(2)-TS	-875.50224	-875.50130	-875.56448
18(3)-TS	-875.49749	-875.49655	-875.55978
19(1)-TS	-955.96668	-955.96574	-956.03743
19(2)-TS	-955.96493	-955.96399	-956.03444
19(3)-TS	-955.96418	-955.96323	-956.03377
20(1)-TS	-1031.17040	-1031.16946	-1031.24214
20(2)-TS	-1031.17173	-1031.17079	-1031.24311
20(3)-TS	-1031.16338	-1031.16244	-1031.23513
20(4)-TS	-1031.16258	-1031.16163	-1031.23464

Table S8. Standard enthalpies of reaction (ΔH°) and activation (ΔH≠) in kcal/mol, imaginary vibrational frequencies (v≠) in cm^{-1}, and tunelling factors (κ), at 298.15 K in water for the f-HAT reactions between phenols and •OOH at the M06-2X(SMD)/6-31++G(d,p) level of theory.

Transition State	ΔH°	ΔH≠	v≠	κ
1-TS	-9.2	2.1	2545.9	8.5
2-TS	-6.4	5.5	2621.2	95.6
3-TS	-6.4	5.5	2530.2	82.8
4-TS	-7.2	5.2	2959.4	125.7
5-TS	-8.8	3.2	2370.9	17.2
6-TS	-8.6	3.6	2420.1	23.1
7-TS	-10.6	2.0	1814.8	4.9
8-TS	-12.4	3.0	2518.7	16.4
9-TS	-13.4			
10-TS	-18.7			
11-TS	-18.5			
12-TS	-5.0	7.6	3211.8	984.9
13-TS	-4.6	7.5	3316.1	1096.8
14(1)-TS	-1.5	7.9	2468.9	215.4
14(2)-TS	-6.5	6.2	2209.9	62.5
14(3)-TS	-1.8	8.4	2543.4	339.9
15(1)-TS	-8.0	5.0	2249.4	39.1
15(2)-TS[a]	-6.9	5.0	2745.6	83.9
A-15(3)-TS[a]	-6.8	5.1	2810.9	100.0
B-15(3)-TS	-6.8	5.2	2234.6	42.8
15(4)-TS	-7.9	4.7	2208.7	33.0
16(1)-TS	-0.5	9.1	3150.6	1799.0
16(2)-TS	0.1	8.4	2744.5	425.7
17(1)-TS	-9.3	5.4	1946.6	25.6
A-17(2)-TS[a]	-4.0	7.0	3014.8	488.2
B-17(2)-TS	-4.0	7.2	2492.2	186.1
18(1)-TS	-1.5	7.7	2477.8	202.3
18(2)-TS	-6.4	6.2	2205.9	63.4
A-18(3)-TS[a]	-1.8	8.6	2812.5	728.4
B-18(3)-TS	-1.8	8.5	2531.1	341.2
19(1)-TS	-4.1	7.9	3050.6	909.3
19(2)-TS	1.9	10.2	3029.0	1013.8
19(3)-TS	1.5	10.4	2976.3	1231.1
A-20(1)-TS[a]	-6.0	6.3	3519.2	583.3
B-20(1)-TS	-6.0	6.4	2800.4	221.3
20(2)-TS	-9.3	4.3	2165.2	24.3
20(3)-TS	1.7	10.1	3056.3	1133.5
20(4)-TS	1.6	10.2	3138.8	1511.6

[a]Hydrogen bonding between •OOH and adjacent hydroxyl is present

Table S9. Standard enthalpies of reaction (ΔH°) and activation (ΔH≠) in kcal/mol, imaginary vibrational frequencies (v≠) in cm^{-1}, and tunelling factors (κ), at 298.15 K in PE for the f-HAT reactions between phenols and •OOH at the M06-2X(SMD)/6-31++G(d,p) level of theory.

Transition State	ΔH°	ΔH≠	v≠	κ
1-TS	-7.4	1.7	1677.9	3.9
2-TS	-4.0	5.0	1688.5	13.4
3-TS	-4.5	4.6	1732.5	13.2
4-TS	-5.0	4.2	1756.1	12.4
5-TS	-6.6	2.7	1366.1	4.3
6-TS	-6.3	3.2	1422.3	5.3
7-TS	-8.4	1.7	1189.4	2.6
8-TS	-8.5	2.8	1384.5	4.5
9-TS	-9.9	1.9	1443.4	3.4
10-TS	-14.1	-1.2	1041.6	
11-TS	-13.2	-0.01	1733.2	
12-TS	-2.4	7.3	2310.2	118.2
13-TS	-2.3	7.2	2346.7	126.2
14(1)-TS	-1.2	6.7	2121.8	56.9
14(2)-TS[a]	-7.3	5.8	2466.2	88.8
14(3)-TS	-1.4	9.2	2179.9	153.7
15(1)-TS	-7.3	4.1	1744.0	11.6
15(2)-TS[a]	-6.2	6.1	2340.5	82.4
15(3)-TS[a]	-6.2	6.2	2373.0	90.7
15(4)-TS	-7.1	4.1	1738.2	11.3
16(1)-TS	1.1	7.7	2327.7	89.1
16(2)-TS	1.6	7.2	2084.7	36.9
A-17(1)-TS[a]	-9.6	8.4	2245.0	171.7
B-17(1)-TS	-9.6	9.1	1727.3	34.9
17(2)-TS[a]	-3.4	7.9	2532.0	279.2
18(1)-TS	-1.2	6.7	2135.2	59.4
18(2)-TS[a]	-7.3	5.8	2485.1	93.0
18(3)-TS[a]	-1.5	8.9	2483.8	349.5
19(1)-TS	-1.9	7.4	2335.6	130.6
19(2)-TS	3.0	8.9	2338.0	72.1
19(3)-TS	2.6	9.1	2365.8	98.4
A-20(1)-TS[a]	-5.4	7.5	2465.5	201.7
B-20(1)-TS	-5.4	7.7	1989.6	60.3
20(2)-TS	-8.7	3.4	1601.6	7.2
20(3)-TS	2.9	8.9	1978.3	34.2
20(4)-TS	2.8	8.9	2360.0	83.9

[a]Hydrogen bonding between •OOH and adjacent hydroxyl is present

Table S10. Standard enthalpies of reaction (ΔH°) and activation (ΔH≠) in kcal/mol, imaginary vibrational frequencies (ν≠) in cm^{-1}, and tunelling factors (κ), at 298.15 K in water for the f-HAT reactions between phenols and $^{\bullet}OOCH_3$ at the M06-2X(SMD)/6-31++G(d,p) level of theory.

Transition State	ΔH°	ΔH≠	ν≠	κ
1-TS	-7.8	2.4	3186.2	15.2
2-TS	-5.0	5.0	3431.2	184.6
3-TS	-5.0	5.1	3444.1	208.4
4-TS	-5.8	4.6	3777.3	175.7
5-TS	-7.4	2.3	2912.3	11.8
6-TS	-7.2	2.9	3230.9	24.3
7-TS	-9.2	0.7	2617.0	1.7
8-TS	-11.0	2.3	3809.1	14.6
9-TS	-12.0			
10-TS	-17.3			
11-TS	-17.1			
12-TS	-3.6	7.0	5641.2	6693.5
13-TS	-3.2	7.0	5790.6	7927.3
14(1)-TS	-0.1	7.2	2653.6	202.9
14(2)-TS	-5.1	4.2	2371.1	33.6
14(3)-TS	-0.4	7.0	2666.3	196.7
15(1)-TS	-6.6	3.5	2381.4	21.2
15(2)-TS	-5.5	4.0	2467.3	32.5
15(3)-TS	-5.4	4.1	2506.2	37.2
15(4)-TS	-6.5	3.3	2355.9	18.7
16(1)-TS	0.9	8.6	3221.4	920.2
16(2)-TS	1.5	7.9	3046.9	319.4
17(1)-TS	-7.9	2.0	2129.2	6.3
17(2)-TS	-2.6	5.6	2540.3	91.4
18(1)-TS	-0.1	6.9	2699.0	197.8
18(2)-TS	-5.0	4.0	2381.5	30.6
18(3)-TS	-0.4	6.8	2683.3	192.6
19(1)-TS	-2.7	7.5	5657.6	12158.8
19(2)-TS	3.3	9.3	2884.4	198.8
19(3)-TS	2.9	9.9	3125.6	549.1
20(1)-TS	-4.6	4.5	3048.5	91.6
20(2)-TS	-7.9	3.5	2637.3	26.5
20(3)-TS	3.1	9.4	2887.4	239.2
20(4)-TS	3.0	9.6	3227.1	498.2

Table S11. Standard enthalpies of reaction (ΔH°) and activation (ΔH≠) in kcal/mol, imaginary vibrational frequencies (v≠) in cm^{-1}, and tunelling factors (κ), at 298.15 K in PE for the f-HAT reactions between phenols and $^{\bullet}OOCH_3$ at the M06-2X(SMD)/6-31++G(d,p) level of theory.

Transition State	ΔH°	ΔH≠	v≠	κ
1-TS	-5.5	2.9	2234.9	13.5
2-TS	-2.1	5.1	2271.7	45.2
3-TS	-2.6	4.9	2230.8	38.0
4-TS	-3.0	4.5	2233.3	32.7
5-TS	-4.7	2.7	1954.4	9.1
6-TS	-4.4	3.2	2077.3	13.8
7-TS	-6.5	1.3	1750.1	3.0
8-TS	-6.6	2.9	1927.2	9.5
9-TS	-7.9	1.8	1780.1	4.5
10-TS	-12.1	-1.7	1343.0	1.0
11-TS	-11.3	0.4	1638.3	1.2
12-TS	-0.5	7.2	2724.6	249.2
13-TS	-0.4	7.1	2701.5	231.3
14(1)-TS	0.8	6.8	2318.9	66.7
14(2)-TS	-5.3	3.6	2132.4	17.1
14(3)-TS	0.5	6.7	2317.3	68.9
15(1)-TS	-5.4	3.5	2002.6	13.9
15(2)-TS	-4.3	4.1	2038.5	19.7
15(3)-TS	-4.3	4.2	2063.1	21.1
15(4)-TS	-5.2	3.4	1997.5	13.6
16(1)-TS	3.0	8.3	2581.4	84.4
16(2)-TS	3.6	7.3	2439.6	27.9
17(1)-TS	-7.7	1.7	1942.6	4.7
17(2)-TS	-1.5	5.6	2216.9	47.7
18(1)-TS	0.7	6.7	2361.5	72.2
18(2)-TS	-5.3	3.5	2140.7	17.1
18(3)-TS	0.4	6.5	2356.0	71.4
19(1)-TS	-0.0	7.4	2703.8	249.3
19(2)-TS	4.9	8.5	2504.6	26.8
19(3)-TS	4.6	9.0	2586.5	50.0
20(1)-TS	-3.5	4.1	2247.3	27.3
20(2)-TS	-6.8	3.3	2002.5	12.4
20(3)-TS	4.8	8.5	2530.1	29.5
20(4)-TS	4.7	9.0	2561.2	46.2

Table S12. Standard Gibbs free energies (G°) in au for neutral and anion forms of phenols at the M06-2X(PCM)/6-311++G(d,p) level of theory in water, molar fractions and predicted pK_a values for the polyphenolic antioxidants.

Species	G° (neutral)[a]	G° (anionic)	Molar fraction	pK_a [b]
14(1)[a]	-764.19445	-763.73993	0.35	7.85
14(2)		-763.74788	8.19	6.49
14(3)		-763.73993	0.36	7.85
15(1)	-999.63805	-999.17823	4.38×10^{-2}	8.76
15(2)[a]		-999.17623	2.00×10^{-2}	9.10
15(3)		-999.17623	1.99×10^{-2}	9.10
15(4)		-999.17732	3.07×10^{-2}	8.91
16(1)	-574.44984	-573.98355	6.03×10^{-3}	9.62
16(2)[a]		-573.98499	6.03×10^{-3}	9.62
17(1)[a]	-457.80125	-457.34949	1.05	7.38
17(2)		-457.33777	1.04×10^{-2}	9.38
18(1)	-685.63605	-685.18149	0.35	7.86
18(2)[a]		-685.18948	8.13	6.49
18(3)		-685.18139	0.34	7.87
19(1)	-766.10932	-765.64712	1.72×10^{-2}	9.17
19(2)		-765.64646	1.32×10^{-2}	9.28
19(3)[a]		-765.64631	1.23×10^{-2}	9.31
20(1)	-841.33471	-840.87621	7.40×10^{-2}	8.53
20(2)[a]		-840.87812	0.16	8.20
20(3)		-840.87003	6.46×10^{-3}	9.59
20(4)		-840.87086	8.94×10^{-3}	9.45

[a] Values previously reported in [11]; [b] Microscopic pK_a values predicted by using the correlation equation at the M06-2X(PCM)/6-311++G(d,p) level of theory reported in [44].

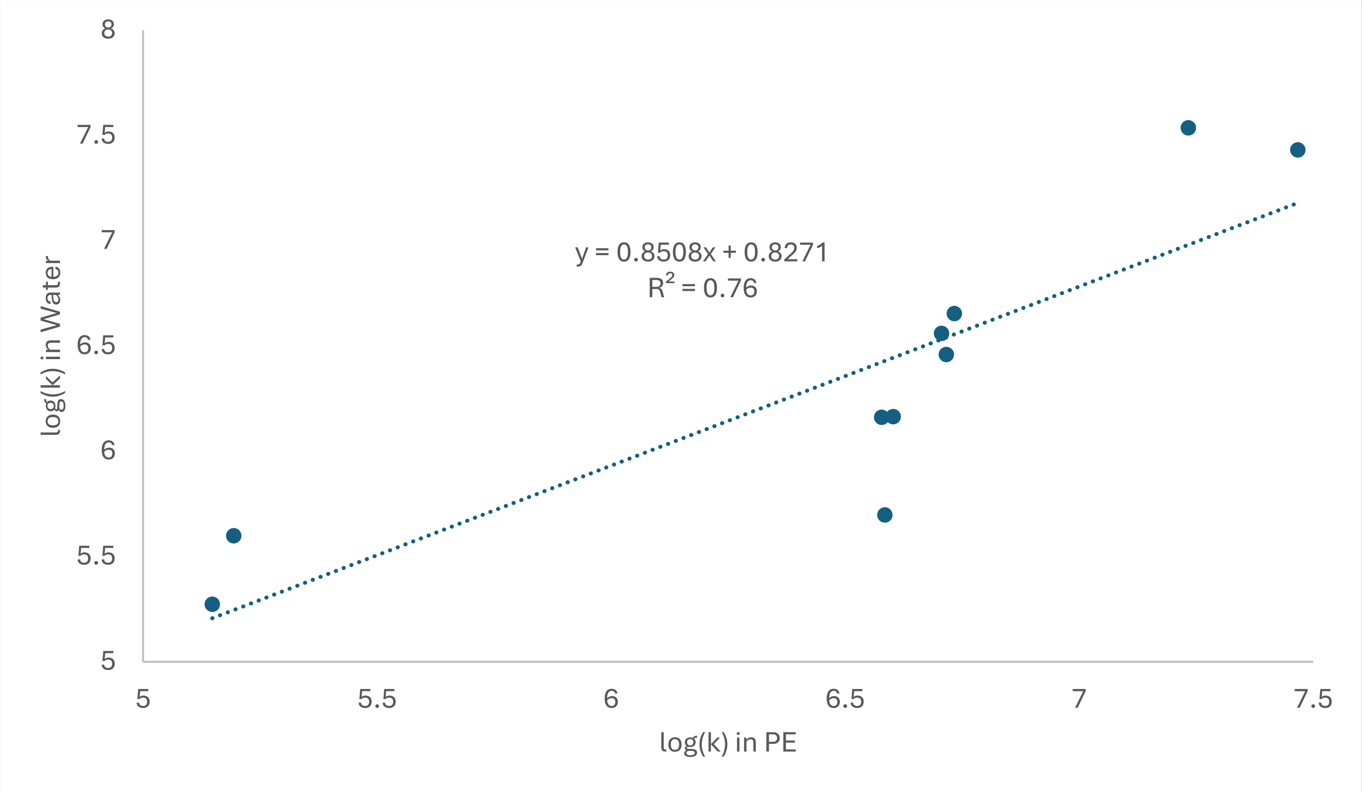

Figure S1. Plot of log(k) in water versus log(k) in PE for f-HAT reactions of monophenols with •OOH at the M06-2X(SMD)/6-31++G(d,p) level of theory.

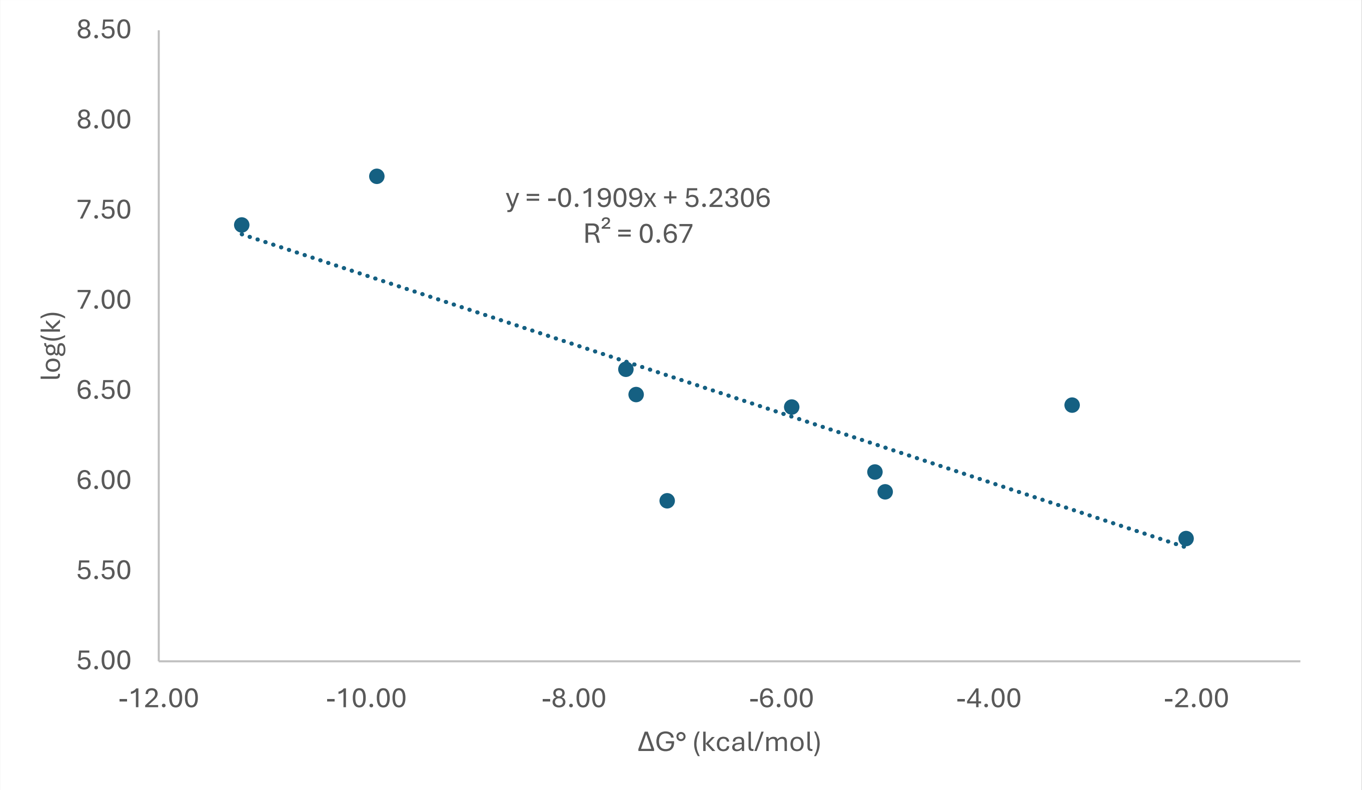

Figure S2. Plot of log(k) versus the ΔG° of f-HAT reactions of monophenols with •$OOCH_3$ at the M06-2X(SMD)/6-31++G(d,p) level of theory in water.

Cartesian coordinates of the optimized transition states between a phenol and •OOH studied at the M06-2X(SMD)/6-31++G(d,p) level of theory in water.

1-TS

Charge = 0 Multiplicity = 2

C,0,-1.9413715391,1.6564516236,0.0894999724
C,0,-2.0047461149,0.2530826713,0.0838658706
C,0,-0.8326932084,-0.517155479,0.0952561289
C,0,0.4310425692,0.074089618,0.122017625
C,0,0.4860297541,1.4975795028,0.122501672
C,0,-0.7052905333,2.2607767532,0.1031329277
H,0,-2.8600882084,2.2332766838,0.0799095108
H,0,-0.916050242,-1.5949694726,0.0870962558
H,0,-0.6128729366,3.3431380616,0.1045436679
O,0,1.6569001909,2.1587261235,0.1023872579
H,0,1.8373897719,2.5718607298,1.0619154758
O,0,-3.2499205127,-0.2784218004,0.0669887354
C,0,-3.3844621411,-1.6999570328,0.0677277755
H,0,-4.4558187955,-1.8934972368,0.0575317934
H,0,-2.9211313894,-2.1321264423,-0.8238030698
H,0,-2.9383934132,-2.1293325034,0.9693465515
C,0,1.7041650237,-0.7764711046,0.1292939253
C,0,1.3873353548,-2.2770146936,0.1174022055
H,0,0.8108203014,-2.5800600523,0.9978043377
H,0,0.8345644516,-2.5711070166,-0.7810713668
H,0,2.3297814095,-2.8330169211,0.1269789437
C,0,2.5424230536,-0.4700922868,-1.1261978818
H,0,2.8663639343,0.5713607017,-1.157125064
H,0,3.4317687364,-1.1097458504,-1.1305568287
H,0,1.9661994645,-0.6838403101,-2.0330081114
C,0,2.5242291932,-0.4859852783,1.3995134227
H,0,3.421799404,-1.1141335848,1.3980948341
H,0,2.8382517831,0.557597048,1.459878699
H,0,1.9421284845,-0.7270093485,2.2955806545
O,0,1.8127033155,2.8340200696,2.416965752
O,0,0.8093960159,2.0181925645,2.8227876429
H,0,-0.0065321775,2.5528902629,2.7659586846

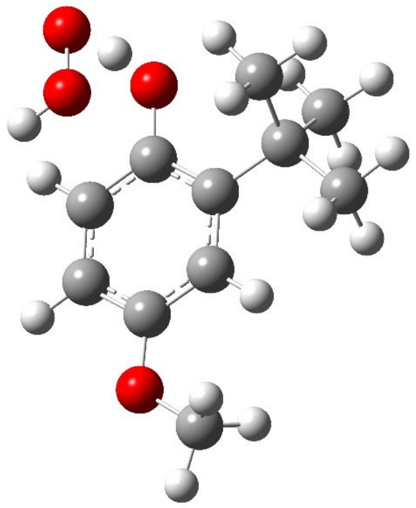

2-TS

Charge = 0 Multiplicity = 2

C,0,-1.2677506848,1.6586455423,0.0476257962
C,0,-0.0150293209,1.0230535833,0.021683502
C,0,0.097549286,-0.4015796992,0.011039945
C,0,-1.0876455241,-1.1189310098,0.0340089138
C,0,-2.3524593407,-0.4921558775,0.0518389316
C,0,-2.4319506392,0.9101159911,0.0591826655
H,0,-1.334086613,2.7391335729,0.0565557722
H,0,-3.4057562818,1.3903883141,0.0752705255
O,0,-3.4696221658,-1.2432767532,0.0254326818
H,0,-3.8996626093,-1.2557338948,0.9976433534
O,0,1.1352090592,1.7260885844,0.0070437859
C,0,1.0791926826,3.1520038304,0.0119274055
H,0,0.5880788169,3.5171902252,0.9184567097
H,0,2.1161498724,3.4836761684,-0.0047736898

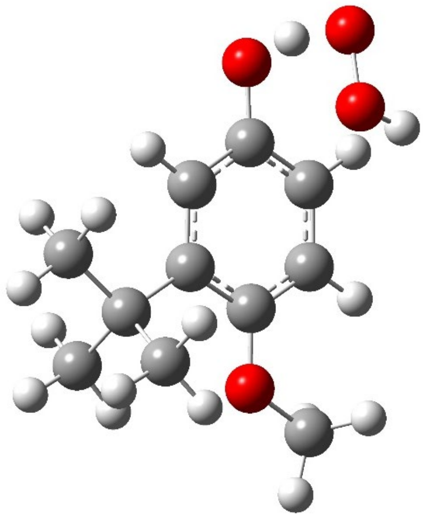

H,0,0.5574285018,3.5217586003,-0.8754420455
H,0,-1.0839927954,-2.2027071941,0.032963956
C,0,1.4609620177,-1.1048756453,-0.0171747945
C,0,1.3013889343,-2.6305454372,-0.0298691412
H,0,0.7869462806,-2.995348559,0.8651864612
H,0,0.7541630482,-2.9765368736,-0.9129956502
H,0,2.2966152152,-3.0847772403,-0.052747247
C,0,2.2373588738,-0.7161479857,-1.2891290092
H,0,2.4571434094,0.3516710841,-1.3304876773
H,0,3.1863033012,-1.262978561,-1.3121455853
H,0,1.667308475,-0.9896304177,-2.1837073687
C,0,2.2746474733,-0.7402630837,1.2385748901
H,0,3.2248334358,-1.2851900452,1.2223433507
H,0,2.4936138464,0.3271167029,1.2947510069
H,0,1.7316699019,-1.0327453236,2.1439117666
O,0,-4.0993815116,-1.1339268817,2.3518828581
O,0,-2.9616940004,-0.4938464145,2.720095343
H,0,-3.1672859446,0.4599436979,2.6697465879

3-TS

Charge = 0 Multiplicity = 2
C,0,0.1255177106,-0.58904806,-0.093547754
C,0,0.4527576165,0.7852082177,-0.0786937249
C,0,2.8064377841,0.1580337855,0.0338924177
O,0,4.1009129921,0.5284637044,0.0561184687
H,0,4.4469640691,0.5099377913,1.0621779852
C,0,-0.6360546534,1.8263379019,-0.1515346965
H,0,-0.7940688947,2.2603442486,0.8429113072
H,0,-0.3136290704,2.6428909374,-0.8037190607
C,0,-1.9285800626,1.2030298324,-0.6690418692
H,0,-1.8374328445,0.9847675594,-1.7396689643
H,0,-2.7705689706,1.8884460858,-0.5372321901
C,0,-2.2424386216,-0.1040293666,0.0557869397
C,0,-3.4617751038,-0.7844558103,-0.5398778781
H,0,-3.6221736657,-1.7591159037,-0.0697343645
H,0,-4.346401954,-0.1652585701,-0.3672190191
H,0,-3.335844354,-0.9246196353,-1.6174133153
C,0,-2.3953592988,0.0772079797,1.5624700671
H,0,-3.1829681668,0.8085088046,1.7672254576
H,0,-2.6711685562,-0.8744441664,2.0255884506
H,0,-1.4682805997,0.4291889524,2.0242855602
O,0,-1.1473169112,-1.0431401558,-0.1699565503
C,0,1.1281573949,-1.5758691987,-0.0575776113
H,0,0.8303734336,-2.6191977092,-0.0775902547
C,0,2.4565137168,-1.211003983,0.005884585
H,0,3.2445138725,-1.9573924063,0.0372000917
C,0,1.7934667115,1.1340710671,-0.0102017302
H,0,2.0809513023,2.1825466228,-0.0019060384
O,0,4.5176108432,0.4047167228,2.4278110024
O,0,3.2330226794,0.0849606885,2.721193037
H,0,3.1958036013,-0.8915029368,2.7134286513

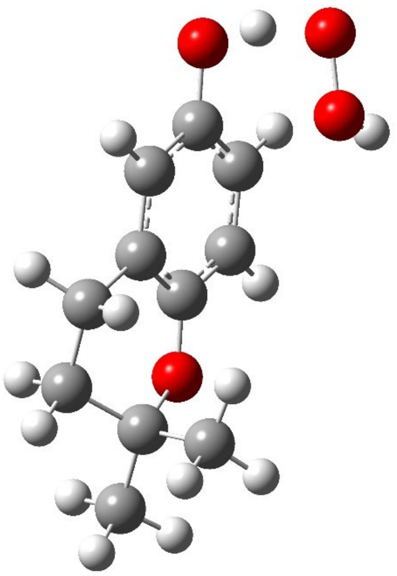

4-TS

Charge = 0 Multiplicity = 2

C,0,-0.0924082017,0.3525661346,-0.11925513
C,0,-0.3688434757,-1.0307504737,-0.1009616804
C,0,-2.7366344501,-0.4943233411,-0.0100529694
O,0,-4.0136999651,-0.9099898472,0.1037919589
H,0,-4.5114032075,-0.7590266306,-0.8172457853
C,0,0.7580948151,-2.0324624662,-0.1520392884
H,0,0.9130196501,-2.4590594309,0.8461103043
H,0,0.4753825038,-2.8612590639,-0.8073215694
C,0,2.0382833657,-1.3683358218,-0.6474820889
H,0,1.9616032018,-1.1575924162,-1.7207392395
H,0,2.9008375976,-2.0233341263,-0.49511903
C,0,2.2890592736,-0.048194028,0.0774776437
C,0,3.4959626798,0.674466189,-0.4930719266
H,0,3.6092771908,1.6561597202,-0.0237776925
H,0,4.3986230691,0.0891482223,-0.297005367
H,0,3.3902787284,0.8059175037,-1.5738825134
C,0,2.4137891316,-0.2176484622,1.5882854776
H,0,3.2217159161,-0.9197931412,1.8149237399
H,0,2.6450391751,0.745222913,2.0526470453
H,0,1.4890897275,-0.6002182329,2.0300299188
O,0,1.1659690196,0.8482120569,-0.1773014949
C,0,-1.1249905707,1.3227828153,-0.1079863718
C,0,-2.4346575083,0.8854712715,-0.0465278695
H,0,-3.2527174553,1.6012747937,-0.0220256443
C,0,-1.6953858813,-1.4358648026,-0.054944521
H,0,-1.9429373239,-2.4942452915,-0.0502553157
C,0,-0.7727378742,2.7823933502,-0.1499933327
H,0,-0.1445676583,3.0579778659,0.7029596525
H,0,-0.2093950972,3.0240023023,-1.0569359625
H,0,-1.6780867523,3.3917485562,-0.1292320822
O,0,-4.7890876102,-0.5806075684,-2.1620637641
O,0,-3.5514302078,-0.3256949227,-2.6531319097
H,0,-3.4275118062,0.6397763729,-2.5674551918

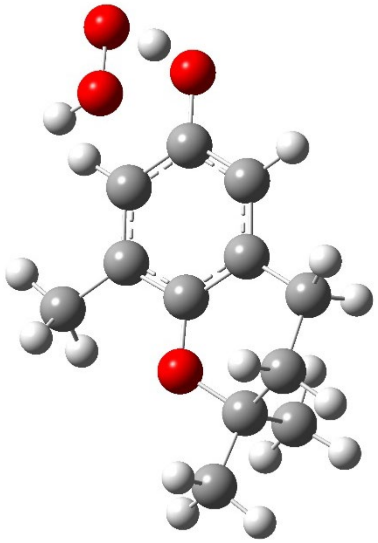

5-TS

Charge = 0 Multiplicity = 2

C,0,0.1637547077,0.6566132637,-0.0869165804
C,0,-0.3737898118,-0.6409697246,-0.1214442283
C,0,-2.5857380951,0.3388985361,0.0509909658
O,0,-3.9256462467,0.2199604915,0.1751885337
H,0,-4.3247077818,-0.1240590802,-0.7289562634
C,0,0.5388341907,-1.8346386556,-0.2711922727
H,0,0.5940217663,-2.3792263478,0.6802787773
H,0,0.1112497454,-2.5309560879,-0.9994239801
C,0,1.9305054339,-1.4035110919,-0.721802065
H,0,1.9106097442,-1.1213264012,-1.7811229684
H,0,2.6461519259,-2.2228348019,-0.6077397411
C,0,2.4216230444,-0.1996832298,0.0756653444
C,0,3.7559863164,0.3028847407,-0.4451417561
H,0,4.0505778428,1.2166822497,0.0794468537
H,0,4.5243042052,-0.4571835768,-0.2781629137
H,0,3.696038038,0.5112465815,-1.5173856036
C,0,2.4836906496,-0.4711546912,1.5755164328

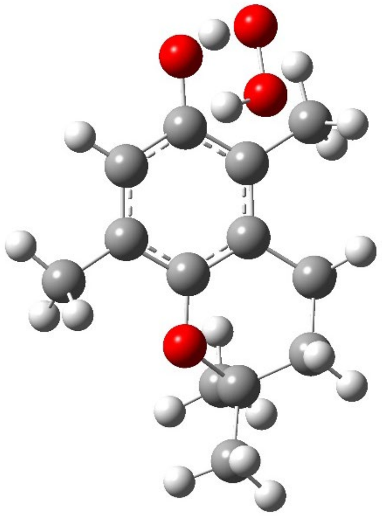

H,0,3.1428196861,-1.3227622724,1.7693420473
H,0,2.8805738609,0.405075415,2.0961950724
H,0,1.4955870474,-0.6989686448,1.9858337645
O,0,1.4973426912,0.9046396252,-0.1435675691
C,0,-0.6552775251,1.8113785849,-0.0150170567
C,0,-2.0220117433,1.6304424296,0.042440109
H,0,-2.6886058112,2.4864727714,0.1007048601
C,0,-1.7609216165,-0.80820437,-0.0332844068
C,0,-2.3673524018,-2.1811044657,-0.0612542359
H,0,-2.3068989053,-2.6090025564,-1.0693052171
H,0,-1.8320027506,-2.8579427099,0.6111841903
H,0,-3.4168837327,-2.1546600285,0.2334660822
C,0,-0.0252767045,3.1747199233,0.0012429554
H,0,0.6513979012,3.2836402825,0.8548014458
H,0,0.5674017582,3.3457855406,-0.90316195
H,0,-0.7946596278,3.9466727552,0.0646652571
O,0,-4.4888048269,-0.4020888403,-2.1103141095
O,0,-3.2083628731,-0.2799731761,-2.5319073305
H,0,-3.1053661014,0.6559235619,-2.7939164433

6-TS

Charge = 0 Multiplicity = 2

C,0,0.1493635768,0.2030945497,-0.0976760118
C,0,0.0912364694,-1.2057433195,-0.0645278158
C,0,-2.3303324829,-1.018705118,0.0398420641
O,0,-3.5162217393,-1.6519702594,0.1727196963
H,0,-4.0386504758,-1.6045033451,-0.735112685
C,0,1.3540135567,-2.0291845053,-0.1157423382
H,0,1.5799712305,-2.4165957997,0.8849567
H,0,1.1938234242,-2.8983705055,-0.7602133732
C,0,2.5161947824,-1.1857553893,-0.6284995258
H,0,2.4017715853,-1.0007786975,-1.7032489339
H,0,3.4691591604,-1.7006952551,-0.4765568202
C,0,2.5666813462,0.1652968638,0.0811276794
C,0,3.6510063765,1.053291375,-0.5025706504
H,0,3.61799132,2.0464739557,-0.0449538083
H,0,4.6316878457,0.6115136778,-0.3049245827
H,0,3.523375759,1.1550448622,-1.5842634478
C,0,2.7209040874,0.0325691895,1.5930258885
H,0,3.623348089,-0.5413402067,1.8242291259
H,0,2.8103739401,1.024179062,2.0458633825
H,0,1.8631621454,-0.4757384968,2.0430829112
O,0,1.3229529735,0.8801260574,-0.1789979362
C,0,-1.0118302033,1.007813963,-0.0874514888
C,0,-2.264675116,0.4009739966,-0.0186846854
C,0,-1.1606093224,-1.7939217722,-0.003039524
H,0,-1.2559227573,-2.8766012659,0.0182190131
C,0,-0.8374773234,2.5012432258,-0.157227752
H,0,-1.7929774362,3.0220820403,-0.108835919
H,0,-0.209221738,2.8555332914,0.6661951049
H,0,-0.3364182504,2.7898403527,-1.0873371692
C,0,-3.5334030966,1.2090024783,-0.0252749369
H,0,-4.4102431306,0.5647636119,0.0387082071
H,0,-3.5600220304,1.9059876538,0.8183247532
H,0,-3.6098709512,1.8025021291,-0.9421727824

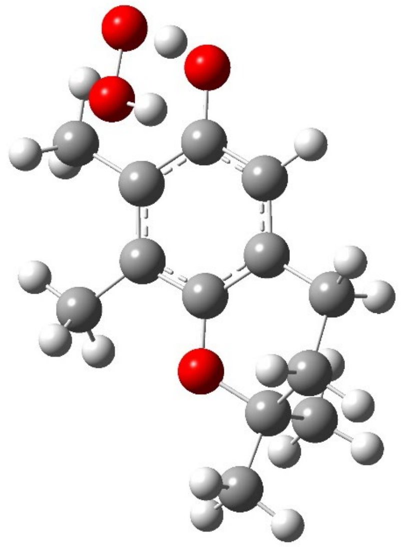

O,0,-4.3361475933,-1.4824829122,-2.1087961058
O,0,-3.2235168062,-0.8440189892,-2.5406916211
H,0,-2.6079332149,-1.5490444986,-2.821336612

7-TS

Charge = 0 Multiplicity = 2
C,0,0.2648006538,0.4662577109,-0.0856532023
C,0,0.0329453223,-0.9203465221,-0.1109870483
C,0,-2.3388987457,-0.4426344819,0.0515418047
O,0,-3.6084885799,-0.8929204598,0.1927432235
H,0,-4.0329736612,-1.0206815952,-0.7468106528
C,0,1.1865637926,-1.8846711606,-0.248927202
H,0,1.3555885844,-2.398996615,0.7061586328
H,0,0.9273253684,-2.6622573138,-0.9743426252
C,0,2.4518197161,-1.1604275655,-0.6957888334
H,0,2.377600861,-0.8972190359,-1.7576559208
H,0,3.3302913759,-1.8001336791,-0.5706713068
C,0,2.6572688458,0.1282772307,0.093736277
C,0,3.8524048722,0.9091255257,-0.4231262104
H,0,3.9327221269,1.8700747949,0.0937672912
H,0,4.7684867459,0.3398025473,-0.2424917142
H,0,3.7578923105,1.0899678708,-1.497913196
C,0,2.7686075656,-0.1119794764,1.596247692
H,0,3.5984963409,-0.7952579632,1.8003694897
H,0,2.9587577592,0.8342189643,2.1111327096
H,0,1.853011216,-0.549409686,2.0047936183
O,0,1.5150314004,0.9993405407,-0.1417714444
C,0,-0.7833855195,1.4121183515,-0.0373914376
C,0,-2.0971078934,0.9570163291,0.0181283451
C,0,-1.2825585074,-1.3788607754,-0.0283668441
C,0,-1.5779360883,-2.8518467154,-0.0644711936
H,0,-1.4455143915,-3.2437667622,-1.0805985472
H,0,-0.896225131,-3.406078865,0.5871295173
H,0,-2.6024798273,-3.0557856841,0.2450960664
C,0,-0.4266221054,2.8745291176,-0.0516939659
H,0,0.2571572126,3.112892158,0.7691124847
H,0,0.0864376551,3.1428920774,-0.9815562421
H,0,-1.309421965,3.5056662842,0.0451265526
C,0,-3.2596023791,1.9147497276,0.0226711389
H,0,-3.2530577427,2.5425889356,0.9199492763
H,0,-3.212135771,2.583749692,-0.8423293971
H,0,-4.2076446077,1.3787285834,-0.0093140999
O,0,-4.218921043,-1.0121773072,-2.1750086731
O,0,-2.9958383481,-0.5958508638,-2.5656016057
H,0,-3.0501164181,0.3794200859,-2.6136867571

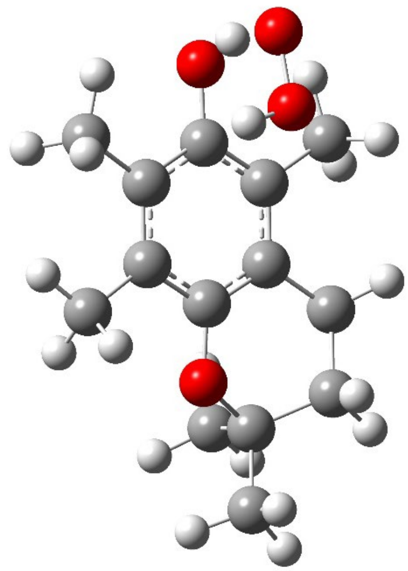

8-TS

Charge = 0 Multiplicity = 2
C,0,1.4148724848,-0.0021134832,0.0469528661
C,0,0.7131150998,1.2166864216,0.0766085469
C,0,-0.668499716,1.2293841501,0.0495180171
C,0,-1.395764562,0.0191099237,-0.0014868273
C,0,-0.6873464418,-1.2025011044,-0.0280114326
C,0,0.6948940309,-1.2106717874,-0.007916001
H,0,1.2756395106,2.1446632491,0.1158184569
H,0,-1.2108023836,2.1703860909,0.0675083145
H,0,-1.2429421947,-2.1349621882,-0.0686966927
H,0,1.2438974891,-2.1476458012,-0.0345082029
O,0,2.7749415644,-0.0067107906,0.0203488438
H,0,3.1331174852,-0.1899339249,0.9693773724
N,0,-2.7647739742,0.0297716482,0.0226762719
H,0,-3.2330910489,0.8970223274,-0.2039742398
H,0,-3.2483229657,-0.8118255319,-0.261522267
O,0,3.2237469032,-0.3822472237,2.4196438443
O,0,1.9234927664,-0.2810585882,2.7545035495
H,0,1.5604379523,-1.1878043875,2.70413658

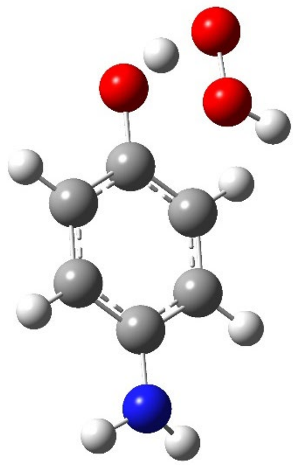

12-TS

Charge = 0 Multiplicity = 2
C,0,-2.2318084667,-1.561430547,0.4057528492
C,0,-2.8101798455,-0.3315165925,0.025889754
C,0,-1.9727088897,0.7645549431,-0.2905971943
C,0,-0.6012728557,0.6330397339,-0.222315822
C,0,-0.0101835364,-0.5934948909,0.160931265
C,0,-0.8573055335,-1.6799303826,0.4741404274
H,0,-2.8844720897,-2.3959524449,0.6424050763
H,0,-2.4342030698,1.7035358536,-0.5814811802
H,0,0.0228677948,1.4870563149,-0.4642422337
H,0,-0.4102101744,-2.62491637,0.769685664
O,0,-4.1420969237,-0.1901608597,0.0046363056
H,0,-4.4977951248,-0.3542270324,-0.9999110991
C,0,1.4299441723,-0.7913451143,0.2476656919
H,0,1.7473948219,-1.777664093,0.5851058721
C,0,2.3848329338,0.1169757722,-0.0522540451
H,0,2.1220200319,1.1152345762,-0.4001771707
C,0,3.8033977404,-0.1751510123,0.0654798298
H,0,4.0734175883,-1.1729342467,0.409540418
C,0,4.7604010889,0.7186097958,-0.2270734031
H,0,4.5070872839,1.7187917536,-0.5711572951
H,0,5.8129560589,0.4728963275,-0.1293980099
O,0,-4.5871948903,-0.6442036557,-2.3052067241
O,0,-3.2966893947,-0.9563120298,-2.5893826586
H,0,-2.8892147205,-0.1245187989,-2.9000883177

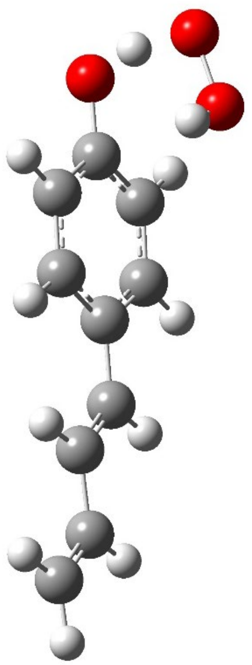

13-TS

Charge = 0 Multiplicity = 2

C,0,-2.3089393714,1.5247994368,-0.1955867966
C,0,-1.4729959988,0.3949949362,-0.0488485787
C,0,-2.0789288272,-0.8728170105,0.1116182438
C,0,-3.4521726716,-1.0029413574,0.1216274487
C,0,-4.277772917,0.1371251194,-0.0279025716
C,0,-3.685267921,1.4082917036,-0.1802910489
H,0,-1.8510328531,2.5023798597,-0.3181379118
H,0,-1.4669278024,-1.7611564878,0.2267737373
H,0,-3.9238702959,-1.973859058,0.2417868634
H,0,-4.3282929579,2.2760071747,-0.2890222124
C,0,-0.0299888616,0.5996732513,-0.0688727298
H,0,0.2792054585,1.6338980925,-0.205770518
C,0,0.9046493338,-0.3650054572,0.0643788553
H,0,0.5936304047,-1.3994757356,0.1981598456
C,0,2.3573817482,-0.167290335,0.0444718236
C,0,2.9644376704,1.0950222477,-0.0856781348
C,0,3.1831786606,-1.2975096868,0.1615315616
C,0,4.3495067419,1.2143350195,-0.0993223067
H,0,2.355899002,1.9895182869,-0.1744165498
C,0,4.5709511405,-1.1767722335,0.1471643521
H,0,2.7241461387,-2.2775179167,0.2637890902
C,0,5.1594019452,0.0805613651,0.0163886537
H,0,4.8021412599,2.19601884,-0.199872457
H,0,5.1909670095,-2.0632430506,0.2377952924
H,0,6.2402973675,0.1801311386,0.005224049
O,0,-5.6102494452,0.0077906567,-0.0627672907
H,0,-5.9798589288,-0.1046366531,0.9434358059
O,0,-6.0962108588,-0.1198253854,2.2796472612
O,0,-4.8233206706,0.1634608867,2.6591049659
H,0,-4.3837784998,-0.7024786482,2.7659562571

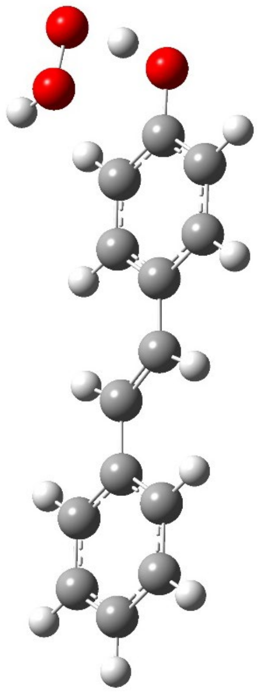

14(1)-TS

Charge = 0 Multiplicity = 2

C,0,0.5460433893,-0.9011532637,0.0416782295
C,0,1.8578000877,-1.3331963322,0.0432655413
C,0,2.8975224208,-0.3901071858,0.0129045263
C,0,2.6022739964,0.9985373062,-0.007574375
C,0,1.2611869707,1.4252631203,0.0056965373
C,0,0.2515087559,0.4800341697,0.0199947046
H,0,-0.2489533871,-1.638212959,0.0614016039
H,0,1.0452212248,2.4879504001,-0.0055893544
O,0,2.1320089565,-2.6694858687,0.0742375403
H,0,3.0905444171,-2.815971129,0.1110602351
O,0,4.1601085299,-0.8358448515,0.017317467
H,0,4.7758067844,-0.0825946896,-0.0369432622
O,0,3.6228803144,1.8481745748,-0.0495916722
H,0,4.0308906874,1.9566177506,0.9863454862
C,0,-1.1573765712,0.9609051415,0.0185990323
O,0,-1.4692517887,2.1404888033,0.0252183192
O,0,-2.0478419615,-0.0275399656,0.0082533928
C,0,-3.4416964264,0.3554762677,0.0049670327
H,0,-3.6270862058,0.9782120497,-0.8755332436
H,0,-3.639776951,0.9457963899,0.9049324841

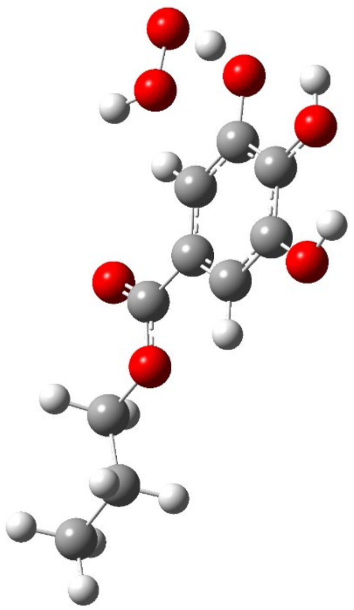

C,0,-4.2668543924,-0.9124469623,-0.0245392281
H,0,-4.0169172816,-1.5231269913,0.8496340909
H,0,-4.0044999295,-1.489377448,-0.9177886557
C,0,-5.7563682485,-0.5795188956,-0.0286674557
H,0,-6.0215935454,0.0208290816,-0.904805946
H,0,-6.3578457521,-1.4915796274,-0.0502968729
H,0,-6.0340461504,-0.013037946,0.8659713771
O,0,4.2237596263,1.8425537148,2.2418639794
O,0,3.3186611489,0.8827883464,2.5732375002
H,0,2.5138452812,1.3677999991,2.8423649858

14(2)-TS

Charge = 0 Multiplicity = 2

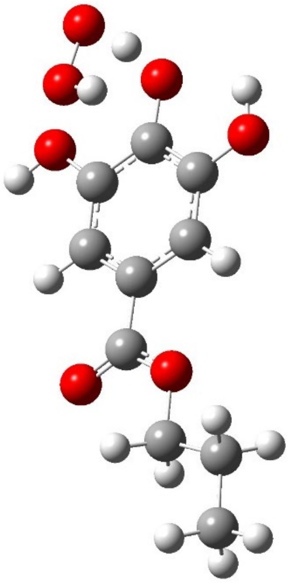

C,0,0.4932658692,-1.0247098757,-0.0330857528
C,0,1.8072300191,-1.4656395199,-0.0353301039
C,0,2.885418997,-0.542983405,0.0065880033
C,0,2.6030990221,0.8468349727,0.0761927155
C,0,1.2888428462,1.2900615986,0.078485472
C,0,0.2529454396,0.352281619,0.0221470069
H,0,-0.31709756,-1.7423566336,-0.0725518998
H,0,1.0720759503,2.3518890763,0.1302847381
O,0,2.0833667837,-2.7868900449,-0.0801800261
H,0,3.049218259,-2.9054337517,-0.1125913212
O,0,4.128149824,-1.0029472155,-0.0391621034
H,0,4.5383392483,-0.998146267,0.9811411918
O,0,3.6788370999,1.6580825288,0.1494111522
H,0,3.403429804,2.5861290558,0.2141910691
C,0,-1.1445733327,0.881407295,0.0254524977
O,0,-1.412838039,2.0704424144,0.0504182432
O,0,-2.0665345448,-0.0750015902,-0.0014213121
C,0,-3.4467671787,0.3560051178,-0.0021280532
H,0,-3.6108581582,0.9855238024,-0.8820911896
H,0,-3.6224259529,0.9521898524,0.8985271999
C,0,-4.3150384575,-0.8826876023,-0.0308672056
H,0,-4.0860633146,-1.5006227992,0.8439333289
H,0,-4.0728007536,-1.4691581681,-0.9235491691
C,0,-5.7922615273,-0.4986657502,-0.0350966569
H,0,-6.0374497386,0.1076722903,-0.9129286245
H,0,-6.4245063117,-1.3897476502,-0.0534547161
H,0,-6.0497547027,0.0797067971,0.8579679316
O,0,4.623464768,-0.9606884686,2.2834363858
O,0,3.4015790684,-0.4666334197,2.607829813
H,0,2.8504125734,-1.2472992589,2.8149833856

14(3)-TS

Charge = 0 Multiplicity = 2

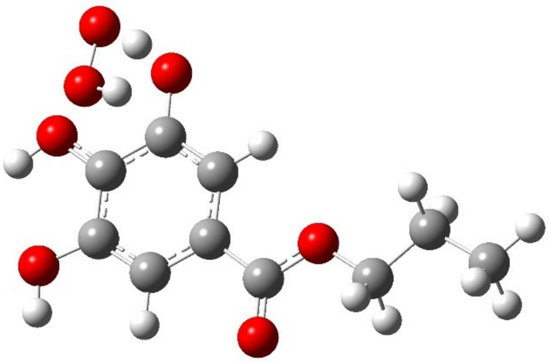

C,0,0.4266893592,-0.9821479491,0.0531436441
C,0,1.7617059547,-1.4366727902,0.054237868
C,0,2.8232277551,-0.4926065724,0.0526610828
C,0,2.5374374136,0.8856600206,0.0456995527
C,0,1.2272089638,1.3149534405,0.0309787607
C,0,0.1718571666,0.3761205366,0.028505288
H,0,-0.3697674802,-1.717268242,0.0600949686
H,0,1.0103798889,2.3788078266,0.0215040808
O,0,2.0523987677,-2.730170181,0.0474531891

H,0,2.5018592932,-2.9880062571,1.0313631274
O,0,4.0769589657,-0.9579185559,0.0657300367
H,0,4.7112215796,-0.2203761992,0.0835189035
O,0,3.6301614353,1.701624675,0.0545128821
H,0,3.3666244249,2.6344072071,0.0418559263
C,0,-1.2180780237,0.9075084554,0.0108669295
O,0,-1.4850532224,2.0981865145,0.0215306586
O,0,-2.1446335517,-0.0465961619,-0.0184772576
C,0,-3.5227789216,0.3890668342,-0.0354757485
H,0,-3.6750826918,1.0213007305,-0.9155739743
H,0,-3.7088586742,0.9831605019,0.8645597623
C,0,-4.3946043875,-0.8468787183,-0.0789914615
H,0,-4.177324033,-1.4693799141,0.7955815504
H,0,-4.1438485355,-1.4302718871,-0.971368313
C,0,-5.8706418773,-0.4588828389,-0.0983642173
H,0,-6.103952344,0.1532434548,-0.9754380984
H,0,-6.5050779799,-1.3480695726,-0.1293457406
H,0,-6.1370884905,0.1150078995,0.7949727273
O,0,2.8261292087,-2.9771620485,2.2747568262
O,0,2.604005066,-1.6738512172,2.6083234485
H,0,1.6992879704,-1.6534229917,2.9756765975

15(1)-TS

Charge = 0 Multiplicity = 2

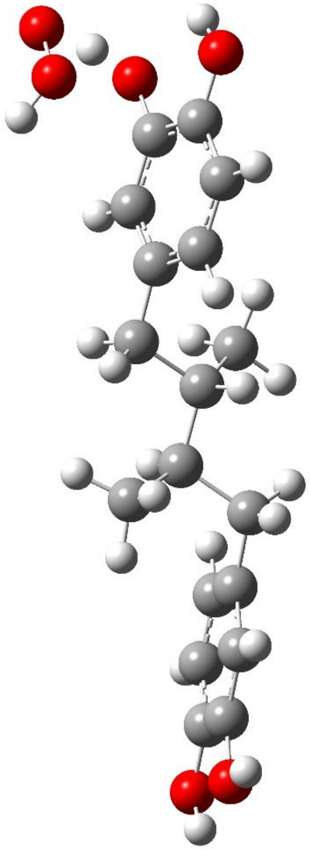

C,0,-4.0574663008,-0.9870928933,-0.4131590415
C,0,-5.382753957,-0.6888534441,-0.1140866367
C,0,-5.7855047187,0.6397739947,0.0527611347
C,0,-4.8538468002,1.6612278478,-0.0855931695
C,0,-3.5265621976,1.3576176505,-0.3897644173
C,0,-3.1106307795,0.0327898074,-0.5581070043
H,0,-3.7682874947,-2.0290251758,-0.5354425572
H,0,-5.1812294862,2.6889001951,0.0401858606
H,0,-2.8069526368,2.1645976526,-0.5031453269
O,0,-7.093620878,0.9457789492,0.3464596937
H,0,-7.6130922477,0.1275325714,0.3785809383
O,0,-6.3654609866,-1.6380501746,0.0342831558
H,0,-6.0030026356,-2.5221028171,-0.1234245455
C,0,-1.6678302087,-0.2837691987,-0.8727833981
H,0,-1.5813929915,-1.338215938,-1.1647738312
H,0,-1.3619703042,0.3142148431,-1.736835726
C,0,-0.7045791723,-0.0300926111,0.3066863362
H,0,-0.8752758518,0.9940222165,0.6740571241
C,0,0.762058891,-0.1077320281,-0.1753112239
H,0,0.8618119949,-1.0214936545,-0.7826138999
C,0,1.7516870148,-0.2238475105,1.0041884644
H,0,1.5300838062,-1.1282314788,1.5792681994
H,0,1.6128183999,0.6360642852,1.6720220058
C,0,3.1891949825,-0.2860646575,0.5556654579
C,0,3.6644109733,-1.4439881927,-0.1066514721
C,0,4.0600624597,0.7749716584,0.7385923938
C,0,4.9609691768,-1.5369752765,-0.5852514584
H,0,2.9887658179,-2.2849046241,-0.2420579566
C,0,5.3808386921,0.711463039,0.2579775275
H,0,3.7413254616,1.6838490914,1.2423287901
C,0,5.8289557242,-0.4562819901,-0.4140620414

H,0,5.3177046569,-2.4287682416,-1.0899977112
O,0,6.237484417,1.7346517982,0.3910700881
H,0,6.9260041598,1.500808654,1.1802015009
O,0,7.0934088545,-0.5312972956,-0.8784783819
H,0,7.5463574664,0.3155578064,-0.7228643125
C,0,-1.0224758437,-1.0084488273,1.4391377934
H,0,-0.4833889257,-0.7663243847,2.3584702383
H,0,-2.091954209,-0.9919112082,1.6718303372
H,0,-0.7589170735,-2.0333336847,1.1474944558
C,0,1.1266092474,1.1039737773,-1.0380293652
H,0,2.1147368692,0.9877139114,-1.4930558311
H,0,1.1476743469,2.010832114,-0.4202917411
H,0,0.4112340102,1.2657924147,-1.8480469967
O,0,7.5698055838,0.866622564,2.1968822011
O,0,6.9109931546,-0.3199508051,2.2148264985
H,0,6.1784915379,-0.2036737296,2.8514708506

15(2)-TS

Charge = 0 Multiplicity = 2

C,0,-4.06188856,-1.0137811895,-0.4137288355
C,0,-5.3927584746,-0.7392820926,-0.1165910056
C,0,-5.8239222753,0.5830016291,0.0284857529
C,0,-4.9143821587,1.6217210526,-0.126978719
C,0,-3.5810929288,1.3418522552,-0.4277316486
C,0,-3.1373178202,0.0237870333,-0.5762294456
H,0,-3.7504549622,-2.051161523,-0.5192052774
H,0,-5.2636734783,2.6439780575,-0.0170735213
H,0,-2.878854417,2.1620462578,-0.5541413183
O,0,-7.1380099593,0.8662068822,0.3182939542
H,0,-7.6425830923,0.0389139246,0.3555397002
O,0,-6.3539976314,-1.7069976252,0.0510679307
H,0,-5.9727874317,-2.5855173423,-0.0925728368
C,0,-1.6881366443,-0.2675640837,-0.8855828382
H,0,-1.5812291493,-1.3224482675,-1.1690369101
H,0,-1.3910887993,0.3298260737,-1.7530878921
C,0,-0.732822344,0.0129457306,0.2944232282
H,0,-0.9125821026,1.0417035204,0.6438673917
C,0,0.7358335637,-0.0636735998,-0.1812346752
H,0,0.8439220688,-0.9884499651,-0.7692564215
C,0,1.7202969055,-0.1497752855,1.0058987535
H,0,1.4983617907,-1.0424492126,1.5991364792
H,0,1.5792191183,0.7251449399,1.6527192893
C,0,3.1569533949,-0.2295392831,0.5647569246
C,0,3.6161738787,-1.3900542873,-0.1014502153
C,0,4.0395510865,0.8261162796,0.7809679895
C,0,4.9217132112,-1.4888052791,-0.5357717616
H,0,2.9272370045,-2.2145218365,-0.2630222701
C,0,5.3590370843,0.7415456394,0.3378930533
H,0,3.7113582174,1.7268461604,1.2945430564
C,0,5.8185451965,-0.4204579446,-0.3382900515
H,0,5.2880740787,-2.3754960968,-1.0435886178
O,0,6.2744715613,1.7204344927,0.5321690947
H,0,5.8744520658,2.4658427298,1.0069402954
O,0,7.069047027,-0.4741101318,-0.8038445033
H,0,7.7577381287,-0.423944248,0.0166435957

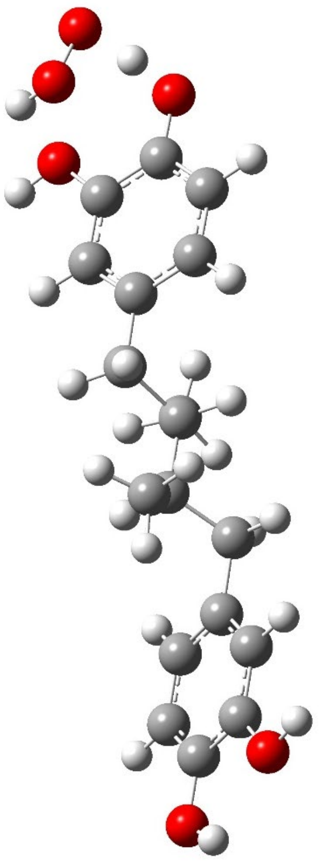

C,0,-1.0472513177,-0.9492072162,1.4415547325
H,0,-0.5124862745,-0.688435926,2.3583106749
H,0,-2.1172471349,-0.9362405296,1.6714207944
H,0,-0.7758192901,-1.9768262822,1.1671340235
C,0,1.1000312091,1.1330124867,-1.0646142719
H,0,2.0961459353,1.016461611,-1.5025282538
H,0,1.1028196097,2.0530552008,-0.4665293428
H,0,0.3955146822,1.2693526798,-1.888650061
O,0,8.3343804154,-0.5472904577,1.2358206343
O,0,7.2525857103,-0.5831978649,2.0591354265
H,0,7.0356223017,0.3549379332,2.2317619188

A-15(3)-TS

Charge = 0 Multiplicity = 2
C,0,-4.0972692858,-0.851093297,-0.5487082114
C,0,-5.4089748356,-0.5038169822,-0.227472736
C,0,-5.7550656606,0.854041406,0.0085324077
C,0,-4.7536343605,1.8388634798,-0.1064933944
C,0,-3.4572986962,1.4810502216,-0.4132789097
C,0,-3.1110299224,0.128218545,-0.6396914283
H,0,-3.8553916802,-1.896759655,-0.7239891448
H,0,-5.034073937,2.8734428572,0.0646082357
H,0,-2.6874969663,2.2440510439,-0.491989885
O,0,-6.9985717266,1.1769105678,0.3713384353
H,0,-7.68213782,0.9266669173,-0.4178239549
O,0,-6.4184420727,-1.4027737085,-0.1390565909
H,0,-6.0880223953,-2.3006465443,-0.3015755633
C,0,-1.6840996288,-0.2293941442,-0.9563784983
H,0,-1.6293534182,-1.2738605905,-1.2856380625
H,0,-1.3540890933,0.3966675377,-1.7914044245
C,0,-0.7331784549,-0.0438987743,0.2484704392
H,0,-0.8891997373,0.9669681859,0.6561326007
C,0,0.7392480715,-0.1299916829,-0.2134066043
H,0,0.8383437654,-1.0282546357,-0.8434770702
C,0,1.7032748294,-0.2922460462,0.9812051259
H,0,1.4609048321,-1.2126445731,1.5206869585
H,0,1.5472207552,0.5447257822,1.6748590317
C,0,3.1557279646,-0.3449024365,0.5716194852
C,0,3.6778219921,-1.4858292082,-0.0455221454
C,0,3.998855584,0.7551610555,0.7630562875
C,0,5.0067285046,-1.5291448492,-0.4689705038
H,0,3.0405755168,-2.3531081535,-0.1983087293
C,0,5.32457487,0.7154967678,0.3450216787
H,0,3.6251439378,1.6597593973,1.2387568324
C,0,5.8329563667,-0.4288193798,-0.2779947116
H,0,5.4159621543,-2.4136757301,-0.9477956132
O,0,6.2056681798,1.7581284797,0.5027631173
H,0,5.7580697656,2.513066383,0.9122468366
O,0,7.1410971661,-0.4801081623,-0.6995893376
H,0,7.570226959,0.3704974819,-0.5194420392
C,0,-1.0904991757,-1.0632947211,1.332329469
H,0,-0.5619169353,-0.8704159693,2.2689171639
H,0,-2.1629789739,-1.0359850197,1.550310743
H,0,-0.8413709936,-2.079601895,1.0014106289
C,0,1.1322782094,1.0998095536,-1.0365728027

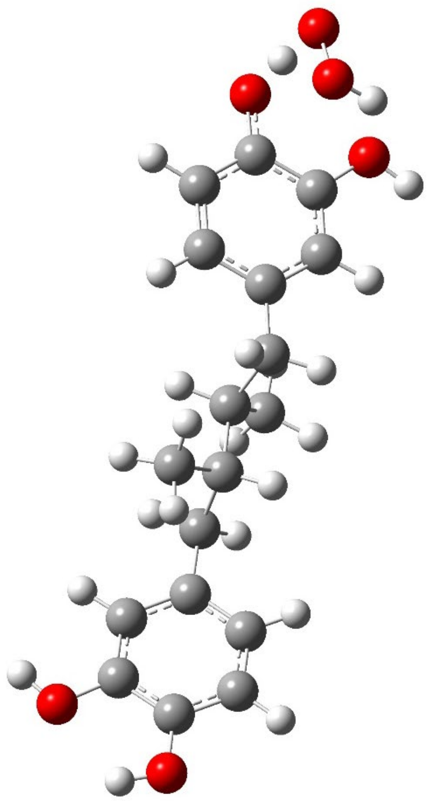

H,0,2.1315000736,0.9871545501,-1.4670382997
H,0,1.1421607061,1.9916557157,-0.396915074
H,0,0.440469324,1.2862475185,-1.8617449439
O,0,-8.2312836409,0.7009329651,-1.6312556377
O,0,-7.1467673426,0.3431646892,-2.3703561792
H,0,-7.027572774,-0.6122179432,-2.1969929813

B-15(3)-TS

Charge = 0 Multiplicity = 2

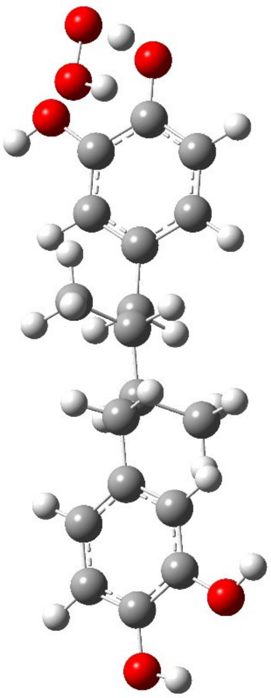

C,0,-4.0824582723,-0.8549929703,-0.4398005327
C,0,-5.4002806516,-0.5339815157,-0.1148674979
C,0,-5.7749123944,0.8198612913,0.0972369415
C,0,-4.7971752616,1.8252946888,-0.0296939343
C,0,-3.5002974702,1.4956890047,-0.3685764906
C,0,-3.1247207379,0.1475065025,-0.5722255056
H,0,-3.8128095617,-1.8983567641,-0.5859220022
H,0,-5.0993217078,2.8551783297,0.1334468392
H,0,-2.7528136097,2.2764811877,-0.4796874813
O,0,-7.0463021211,1.11877034,0.3872479464
H,0,-7.2682551079,0.7324816298,1.3571552779
O,0,-6.3787100262,-1.4537498966,0.0278231592
H,0,-6.0272399015,-2.3457873576,-0.1191406899
C,0,-1.6939466671,-0.1847667467,-0.9008503522
H,0,-1.618491453,-1.2405887235,-1.187538891
H,0,-1.3974577619,0.414032469,-1.7675396128
C,0,-0.7241041355,0.0749275522,0.2745867048
H,0,-0.8546422304,1.1178850824,0.6026522906
C,0,0.7402393099,-0.0809713127,-0.1954054524
H,0,0.8164703515,-1.0417378357,-0.7290893013
C,0,1.7192559493,-0.1349265077,0.9970503524
H,0,1.463075244,-0.9825958096,1.6392343777
H,0,1.5970635103,0.778154024,1.5949642534
C,0,3.1629099644,-0.2753705908,0.5768785455
C,0,3.6380425101,-1.4905416829,0.0739615972
C,0,4.0443463291,0.8091449789,0.6457213981
C,0,4.9589610258,-1.623541304,-0.3557623378
H,0,2.970089054,-2.3463331558,0.0182704147
C,0,5.3625272094,0.6802073222,0.2211459685
H,0,3.708248521,1.7701065052,1.0301888994
C,0,5.8239570406,-0.538953356,-0.2856220899
H,0,5.33181842,-2.5664050693,-0.7444600959
O,0,6.2822175885,1.7002791942,0.2624861409
H,0,5.8737184181,2.5030313485,0.6179235741
O,0,7.1250377191,-0.6767910932,-0.709089117
H,0,7.5870897922,0.1686747657,-0.6000256006
C,0,-1.0844960435,-0.8477654566,1.4413137521
H,0,-0.5345788979,-0.5946659594,2.3507801449
H,0,-2.151690685,-0.7778608629,1.6756185735
H,0,-0.8647278383,-1.8928257537,1.1877131165
C,0,1.1439484757,1.0475981045,-1.1481418298
H,0,2.1496927532,0.8873356394,-1.5472087694
H,0,1.1450348316,2.0069210217,-0.6148665815
H,0,0.4668431171,1.1366920376,-2.0009475457
O,0,-7.1200900521,0.2883054895,2.6466344089
O,0,-5.8050306544,-0.0559280253,2.6473991369

H,0,-5.327718892,0.7381122406,2.9574098976

15(4)-TS

Charge = 0 Multiplicity = 2

C,0,-4.0754372982,-1.0523871807,-0.4420675469
C,0,-5.405320893,-0.7285947155,-0.1154927085
C,0,-5.7767167942,0.630731899,0.0520397747
C,0,-4.8237677622,1.6380820505,-0.1119742595
C,0,-3.5208809844,1.2894163114,-0.4296744437
C,0,-3.1225677585,-0.0591676079,-0.5960276879
H,0,-3.8181137896,-2.1016299508,-0.5620774127
H,0,-5.1209182413,2.6746137616,0.0088319824
H,0,-2.7796009176,2.0743935128,-0.5591803914
O,0,-7.0513469467,0.9519098363,0.3567714022
H,0,-7.5672250989,0.1351221273,0.473164569
O,0,-6.3450266089,-1.6689457831,0.0638055151
H,0,-6.9378418706,-1.7309844961,-0.8299500449
C,0,-1.6839017753,-0.3775371707,-0.9129818932
H,0,-1.5996233403,-1.431038496,-1.2069545273
H,0,-1.3799752279,0.2258950646,-1.7739575339
C,0,-0.7276992816,-0.1234468789,0.2733735752
H,0,-0.9124047909,0.8949836406,0.650216384
C,0,0.7431756521,-0.1779959855,-0.1987617212
H,0,0.8652281293,-1.0941231889,-0.7986017141
C,0,1.7222942344,-0.2688277821,0.9910255798
H,0,1.50929149,-1.173772952,1.5676809688
H,0,1.5501750456,0.5897854185,1.6538332662
C,0,3.172362854,-0.3004171666,0.570971213
C,0,3.719107948,-1.4526294385,-0.0023601125
C,0,3.9888911138,0.8271828713,0.7111978243
C,0,5.0473796002,-1.4821814186,-0.4290307708
H,0,3.1025033262,-2.3407105003,-0.1151422132
C,0,5.3135228759,0.8017281325,0.2888019507
H,0,3.5952609521,1.7413417474,1.1511936885
C,0,5.8477362013,-0.3559069023,-0.2860201021
H,0,5.476382183,-2.3762388115,-0.8714723183
O,0,6.1701084859,1.8705318397,0.397534914
H,0,5.7119231153,2.6238955834,0.7978355728
O,0,7.1564740638,-0.3936844146,-0.7071309502
H,0,7.5729459809,0.4651149733,-0.5363575808
C,0,-1.0403853653,-1.1174779683,1.3939369914
H,0,-0.5147128926,-0.8737332248,2.3203392281
H,0,-2.1120078806,-1.1231256455,1.6171988866
H,0,-0.7550559948,-2.1344814084,1.0957301218
C,0,1.0912645101,1.0338808876,-1.0674768624
H,0,2.0929066348,0.9403200952,-1.4970193958
H,0,1.0718546213,1.9481682835,-0.4605304581
H,0,0.3916582588,1.1662616836,-1.8964469504
O,0,-7.4375233965,-1.4973938259,-2.0707324778
O,0,-6.6707272747,-0.4484778579,-2.4604262865
H,0,-5.8954570924,-0.8413199488,-2.9076770442

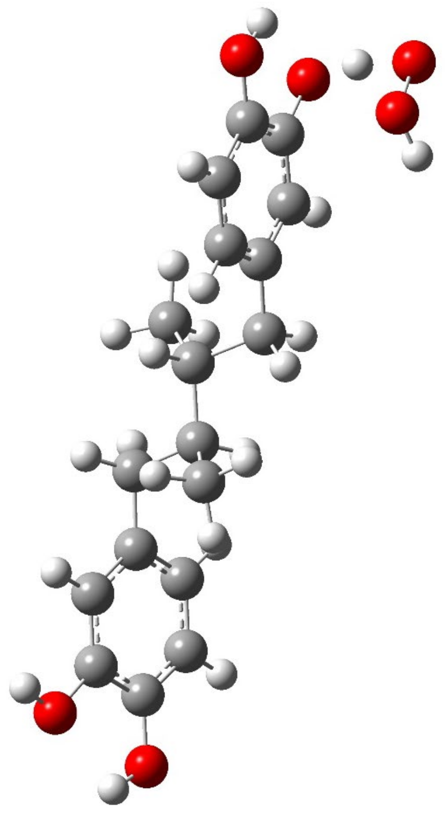

16(1)-TS

Charge = 0 Multiplicity = 2

C,0,-0.2774996211,-0.8247421469,-0.0283820969
C,0,-0.443939122,0.5669162056,0.0306766874
C,0,-1.8073615478,1.2007887448,0.0130938934
H,0,-2.0290773227,1.6020949917,1.0108905381
H,0,-1.800037403,2.0558575648,-0.6698763469
C,0,-2.8694224981,0.1821229839,-0.3939022729
H,0,-2.8366534612,0.0063749024,-1.4747106258
H,0,-3.8688026971,0.5452898028,-0.1416593195
O,0,-1.343172069,-1.6805436606,-0.0714805309
C,0,0.9861356562,-1.4045682832,-0.0644824712
H,0,1.1001606786,-2.4831821897,-0.1070494905
C,0,2.1277842922,-0.5746735978,-0.030431902
C,0,0.7225300779,1.3677296086,0.0899587736
C,0,-2.6107552954,-1.1264055108,0.3236478423
H,0,-3.3448583171,-1.8920007919,0.0722033798
H,0,-2.5986315826,-0.9803121585,1.4112822715
C,0,1.9928110729,0.8272454776,0.0438792097
H,0,2.8768856828,1.4558312755,0.0759725881
O,0,0.5062198815,2.7102838968,0.182362507
H,0,1.3515263429,3.1829222064,0.2181198039
O,0,3.3429292736,-1.1206258296,-0.0348580082
H,0,3.5375620601,-1.5629264557,-1.0305486312
O,0,3.5367247449,-1.8584885639,-2.2837948621
O,0,2.3470072792,-1.3391101472,-2.7001121268
H,0,1.6947558943,-2.0562353252,-2.58208181

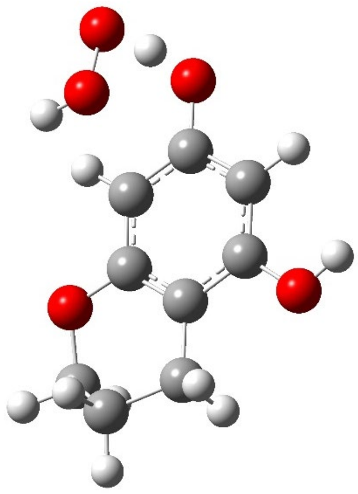

16(2)-TS

Charge = 0 Multiplicity = 2

C,0,-0.1956940389,-0.8199892552,-0.0079959991
C,0,-0.3154938299,0.5750585042,0.030890196
C,0,-1.6528434157,1.2551893127,-0.0398986238
H,0,-1.9183965171,1.6373938526,0.9545056344
H,0,-1.586133083,2.1249708895,-0.701314943
C,0,-2.7158026854,0.273127714,-0.5261830414
H,0,-2.607287473,0.0966658079,-1.6023882422
H,0,-3.719151567,0.6680354493,-0.347987188
O,0,-1.283041616,-1.6454365907,-0.0893585497
C,0,1.048904287,-1.4432599438,0.0102689561
H,0,1.1152344107,-2.5258552179,-0.0233094307
C,0,2.219032076,-0.6687814103,0.0789353386
C,0,0.8897637902,1.3291954611,0.1157415733
C,0,-2.555235983,-1.0426468516,0.2082233534
H,0,-3.2961284939,-1.7821228784,-0.0961734425
H,0,-2.6194731651,-0.8929331728,1.293494551
C,0,2.1566952338,0.71172632,0.1289983457
H,0,3.0516353857,1.3227284887,0.1872714769
O,0,0.8094011359,2.6563276229,0.191223952
H,0,0.5651496852,3.0517422074,-0.8140828688
O,0,3.3964580671,-1.3581740017,0.0957286061
H,0,4.1441711938,-0.743939792,0.1457640572
O,0,0.381674559,3.182732192,-2.0882863173
O,0,0.3343349339,1.8798783644,-2.4907623869
H,0,1.2575161098,1.6441409278,-2.7051860071

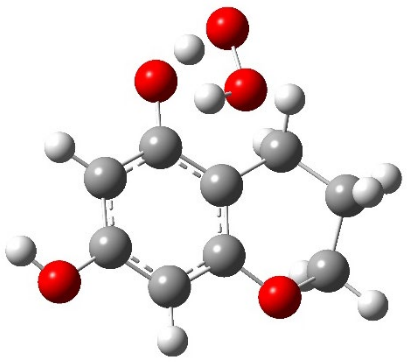

A-17(1)-TS

Charge = 0 Multiplicity = 2

C,0,-0.1150805579,2.0529004129,-0.0034065037
C,0,-1.2748686001,1.2767317996,0.0604186984
C,0,-1.1633156464,-0.1075093855,0.0727229684
C,0,0.1106953082,-0.7273868061,0.0038771693
C,0,1.268295401,0.093382924,-0.0469705031
C,0,1.1555207562,1.4786938872,-0.0534428684
H,0,-0.2057521188,3.1343040725,-0.011047527
H,0,-2.2568878359,1.7368022346,0.1030028556
H,0,2.0497996636,2.0922288062,-0.0932183627
O,0,-2.2267380783,-0.9454444017,0.1433447749
H,0,-3.0492592004,-0.4327671468,0.1720143251
O,0,2.4474106141,-0.5696652634,-0.0752925575
H,0,3.1858434351,0.0582027349,-0.1013364266
O,0,0.2185666748,-2.0550920041,-0.036568416
H,0,0.4942662002,-2.4045713986,0.9423872028
O,0,0.7173216441,-2.4515049998,2.2738133087
O,0,0.5954037129,-1.139738993,2.5952809182
H,0,-0.3435203725,-1.0158964731,2.8379659435

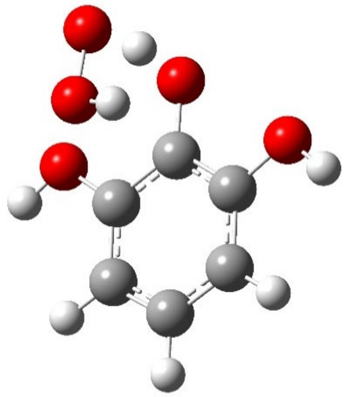

A-17(2)-TS

Charge = 0 Multiplicity = 2

C,0,-0.1293159096,2.1488146917,-0.1444940526
C,0,-1.3071336962,1.4338390796,-0.0911494151
C,0,-1.2664091123,0.0250379999,0.0036589724
C,0,-0.0092344565,-0.6311402593,0.0647285571
C,0,1.177171164,0.1187188785,0.0245111093
C,0,1.1195010909,1.496327294,-0.0831409392
H,0,-0.1524429013,3.2295728552,-0.2319484608
H,0,-2.2766767638,1.9176959496,-0.1355652647
H,0,2.044461555,2.0642351361,-0.1167020821
O,0,-2.3792115617,-0.7000834791,-0.0017430578
H,0,-2.4562346014,-1.265207625,0.9361946046
O,0,2.3272225348,-0.6157874155,0.0954701377
H,0,3.1039774255,-0.0392660685,0.0379610253
O,0,0.0040392072,-1.9756162959,0.1788802464
H,0,0.9222344152,-2.2954806939,0.1980424249
O,0,-2.3839853335,-1.6522666724,2.1790934676
O,0,-1.2519336664,-1.0325450361,2.6188618091
H,0,-0.5222213901,-1.621705339,2.3416459178

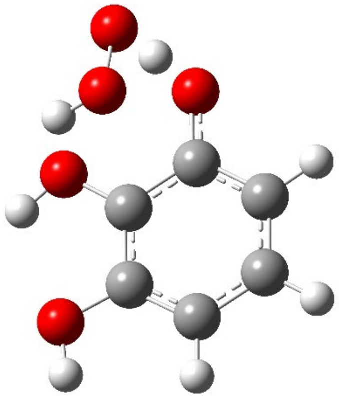

B-17(2)-TS

Charge = 0 Multiplicity = 2

C,0,-0.1671183386,2.0693593123,-0.0033663448
C,0,-1.3224549791,1.3159629672,0.0124062875
C,0,-1.2358047687,-0.0932276571,0.0123560922
C,0,0.0392511495,-0.7152432514,0.0162518106
C,0,1.2023473852,0.0732445556,0.0145608153
C,0,1.101445865,1.4523872577,0.0015456916
H,0,-0.2236345164,3.1524060939,-0.0145313327
H,0,-2.3069992665,1.7710302669,0.01385148
H,0,2.0082075393,2.0499026485,-0.0030654579
O,0,-2.3237046679,-0.8597979808,-0.0090735406
H,0,-2.3741227551,-1.4060319568,0.9319963592

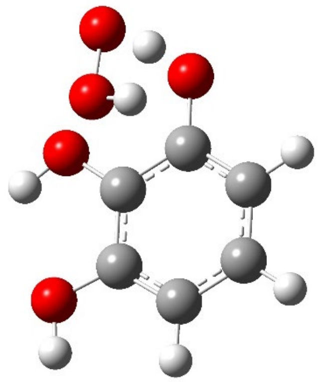

O,0,2.374723062,-0.6302745147,0.0244173122
H,0,3.1346808605,-0.0293242101,0.0187980864
O,0,0.0883471741,-2.0588914858,0.0288618566
H,0,1.0135588605,-2.3556630297,0.0557598404
O,0,-2.2281335727,-1.7453359991,2.205623763
O,0,-1.1518320602,-0.9892694006,2.5615147525
H,0,-1.5259219706,-0.157493616,2.9105695285

18(1)-TS

Charge = 0 Multiplicity = 2
C,0,0.1235637583,-1.3698447263,0.0234837559
C,0,1.5224394984,-1.2149208322,0.0124117959
C,0,2.0852980725,0.087875542,-0.0400002614
C,0,1.2525775955,1.2173346482,-0.06348224
C,0,-0.1185046552,1.0519409961,-0.0401705303
C,0,-0.6794302636,-0.2434153051,0.0046490595
H,0,-0.2976588022,-2.3686650347,0.0566038098
H,0,-0.7524469268,1.9312455394,-0.0570079069
O,0,1.7829265438,2.4731026391,-0.1123662127
H,0,2.7517308066,2.4265286263,-0.0997085116
O,0,3.4108392988,0.2720036462,-0.0771310398
H,0,3.8628804322,-0.5898218315,-0.0285633246
O,0,2.3598688145,-2.2451435424,0.0603735025
H,0,2.7174353551,-2.4559695006,-0.9782790544
C,0,-2.1549796193,-0.4332683259,0.0305554426
O,0,-2.6992417474,-1.5240173183,0.069993192
O,0,-2.8296152183,0.7152564994,0.0066535976
C,0,-4.2631191865,0.6105930678,0.0320985144
H,0,-4.5833081182,0.1084517874,0.9465402208
H,0,-4.6281784829,1.6350157792,0.0096358815
H,0,-4.6116153523,0.0593604827,-0.8428526513
O,0,2.9186132984,-2.4068835606,-2.2360684799
O,0,2.1816588018,-1.3209422816,-2.5937371186
H,0,1.2982720967,-1.6718929943,-2.8212454412

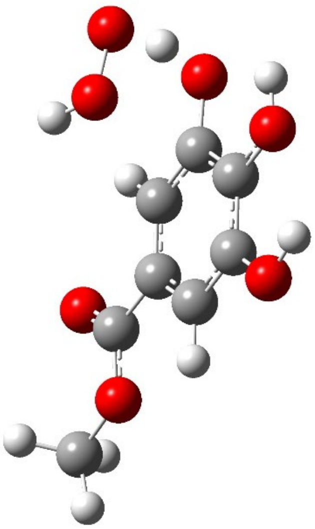

18(2)-TS

Charge = 0 Multiplicity = 2
C,0,0.1937371591,-1.3589721147,0.0558332584
C,0,1.572647164,-1.2106893751,0.0605078405
C,0,2.1508402599,0.0850096614,0.0094030408
C,0,1.2994628253,1.2181803937,-0.0698534314
C,0,-0.0787003524,1.0728488585,-0.0699842399
C,0,-0.6130989982,-0.2187413047,-0.0052435841
H,0,-0.2487328279,-2.3486214833,0.0957427405
H,0,-0.7135277901,1.9488633497,-0.1212759559
O,0,1.8555932182,2.4469673223,-0.1402820817
H,0,2.8239166986,2.3556349572,-0.0971814371
O,0,3.4632344024,0.2670333277,0.0536851554
H,0,3.8796776364,0.0457657546,-0.9398837112
O,0,2.4427855948,-2.2406951698,0.0995472473
H,0,1.9652935591,-3.0852700274,0.1142107994
C,0,-2.0916268738,-0.4302797027,-0.001202135
O,0,-2.6158975926,-1.5298067358,0.0367121748
O,0,-2.7825367191,0.7058927762,-0.0408813491
C,0,-4.2147649039,0.576555218,-0.0347635238

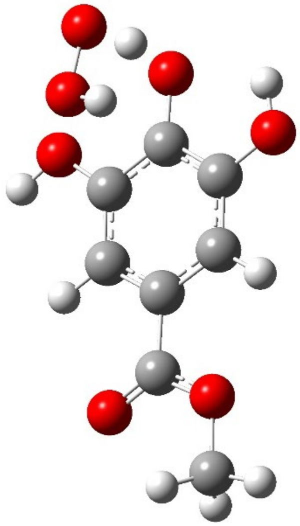

H,0,-4.5379950349,0.0726524807,0.8776435438
H,0,-4.5970711326,1.5942937598,-0.0671436899
H,0,-4.5405906061,0.0148189595,-0.9117433226
O,0,3.974064823,-0.169689438,-2.2237407719
O,0,2.6783917989,-0.4191006192,-2.5420912142
H,0,2.3177116919,0.4336971512,-2.8557233529

A-18(3)-TS

Charge = 0 Multiplicity = 2

C,0,0.1412317417,-1.2710895024,0.0128394347
C,0,1.5058371647,-1.0738177429,-0.0102407608
C,0,2.0262645221,0.2329987738,-0.0086287258
C,0,1.1487729301,1.3494438363,0.0033284295
C,0,-0.2452232997,1.1331405812,0.0101990473
C,0,-0.7321003349,-0.1603538141,0.0287135552
H,0,-0.2593336379,-2.2800658083,0.0160009801
H,0,-0.9014868645,1.9955030587,0.0149817246
O,0,1.6597015649,2.5709642481,0.033401591
H,0,2.1958278616,2.7637817409,-0.9249304864
O,0,3.344693785,0.4681031243,-0.0431738738
H,0,3.8321323041,-0.3737383292,-0.0707134834
O,0,2.4439064671,-2.0625818265,-0.0354671138
H,0,2.0260023812,-2.9370515204,-0.0362524533
C,0,-2.1935469507,-0.4396593403,0.0518917517
O,0,-2.6674172542,-1.5634756293,0.0447962358
O,0,-2.9383966407,0.6636963496,0.0807913754
C,0,-4.3622962,0.4656948108,0.1010986569
H,0,-4.6474124054,-0.0941278232,0.9933816723
H,0,-4.7924981325,1.4645742198,0.1220991578
H,0,-4.6765583357,-0.0693480553,-0.7965463502
O,0,2.5965965554,2.7428790474,-2.1382661735
O,0,2.0910561863,1.562666941,-2.5974632883
H,0,2.801641592,0.9076546599,-2.4514189031

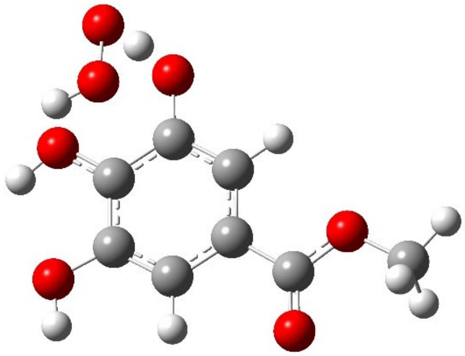

B-18(3)-TS

Charge = 0 Multiplicity = 2

C,0,0.1330047457,-1.2512282655,-0.0267458118
C,0,1.4955472154,-1.0464937915,-0.0660563827
C,0,2.0106012169,0.2633696026,-0.06652305
C,0,1.125988761,1.3740027651,-0.0377242382
C,0,-0.266364502,1.1505627251,-0.0118649595
C,0,-0.7467889349,-0.1456315814,0.0062332543
H,0,-0.2616937519,-2.2625468183,-0.0226740008
H,0,-0.9268561321,2.0096350131,0.0043822049
O,0,1.6325032381,2.5988092699,-0.0269383939
H,0,2.1113310354,2.7830944214,-1.013885365
O,0,3.3248347857,0.5041648299,-0.1076903911
H,0,3.8161134361,-0.3345009858,-0.1531753438
O,0,2.4382151055,-2.0310896801,-0.1082977076
H,0,2.0238678648,-2.907182842,-0.1137925315
C,0,-2.2056648557,-0.4328095422,0.0482564758
O,0,-2.6743703828,-1.5589607765,0.0419720466
O,0,-2.9562274567,0.6666214349,0.0931703641
C,0,-4.3785823072,0.4610907668,0.1325679999

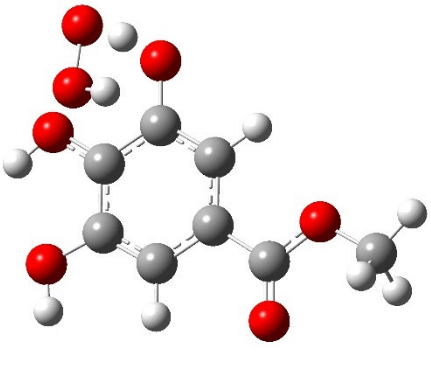

H,0,-4.6483410697,-0.1048890731,1.0257592368
H,0,-4.8135952413,1.4575847055,0.1651334247
H,0,-4.703071942,-0.0707880405,-0.7633315498
O,0,2.4152384556,2.7214339793,-2.2617051226
O,0,1.950069757,1.4847143404,-2.5982717631
H,0,1.0513779592,1.6353085429,-2.9499453957

19(1)-TS

Charge = 0 Multiplicity = 2
C,0,-4.9204135232,-0.3763346691,0.0006684192
C,0,-4.0179360302,-1.4635897464,-0.0736129194
C,0,-2.6578673961,-1.2394718695,-0.0569338855
C,0,-2.1407850338,0.0743512776,0.0384008217
C,0,-3.0512026641,1.1503944211,0.1194401822
C,0,-4.4168991071,0.9384498,0.104136057
H,0,-4.4210787257,-2.4690800777,-0.1452822144
H,0,-1.9854892141,-2.0887529043,-0.1160249085
H,0,-2.6631051148,2.162252177,0.1947825588
H,0,-5.1185536993,1.7648247172,0.1645993359
O,0,-6.2400770088,-0.5924990469,-0.0637532409
H,0,-6.6233865677,-0.737871136,0.9375279976
C,0,-0.7138028161,0.3744735111,0.0563770286
H,0,-0.4714252403,1.432546513,0.1330777967
C,0,0.2798956625,-0.5347025409,-0.0169196056
H,0,0.0383859196,-1.5926459647,-0.0948965555
C,0,1.7178086771,-0.240222877,-0.0062353086
C,0,2.2262500466,1.0643519819,0.0950038042
C,0,2.6000988883,-1.3242892908,-0.1059514719
C,0,3.6027202468,1.2597329652,0.0951327802
H,0,1.5768356866,1.9292953013,0.1724094406
C,0,3.9754808836,-1.1003804249,-0.1055480511
H,0,2.2236410812,-2.3393232465,-0.1856618364
C,0,4.4936757355,0.1882771548,-0.004982546
H,0,5.5674537316,0.357978825,-0.0051291647
O,0,4.055555194,2.5499949136,0.1960368448
H,0,5.0239214764,2.5659883688,0.1818718068
O,0,4.7911324396,-2.1965197659,-0.2096800928
H,0,5.7198902226,-1.9223355117,-0.206196457
O,0,-6.7501580969,-0.8309153878,2.26163892
O,0,-5.4609905369,-0.6645487222,2.6559631769
H,0,-5.3601741171,0.293225255,2.8208992872

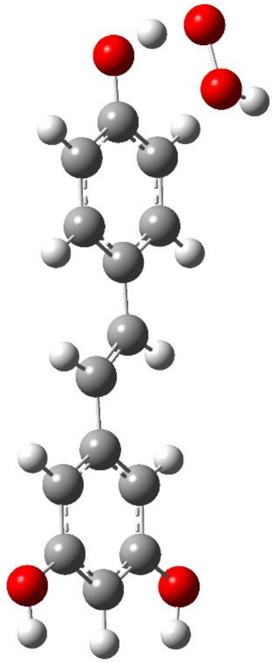

19(2)-TS

Charge = 0 Multiplicity = 2
C,0,-4.9190928775,-0.1182148562,0.0132875011
C,0,-4.0840220772,-1.2398046484,-0.0044336128
C,0,-2.7066691452,-1.0742168729,0.012823685
C,0,-2.1282617912,0.2073311871,0.0475324267
C,0,-2.9889162934,1.3142773085,0.070385111
C,0,-4.3725876726,1.1633321864,0.0534651504
H,0,-4.5263910582,-2.2306712401,-0.0325609983
H,0,-2.0779091671,-1.9592219313,-0.0017495296
H,0,-2.5652074077,2.3148422676,0.0993238376
H,0,-5.0269354459,2.0305044571,0.0690295507
O,0,-6.2715326194,-0.3376681704,-0.0110201817

H,0,-6.7455287016,0.5072158509,-0.0135319899
C,0,-0.6788191901,0.4428539505,0.0550543585
H,0,-0.3967115156,1.493567702,0.1049630805
C,0,0.2802243392,-0.4967078944,-0.0007470952
H,0,0.0093030219,-1.5493584498,-0.0511330815
C,0,1.7283637508,-0.2388614532,-0.0017141774
C,0,2.2806903676,1.0505152976,0.0190189758
C,0,2.5977313776,-1.3324925988,-0.028884187
C,0,3.6851903923,1.2234939264,0.0213644402
H,0,1.6632001608,1.9433961588,0.0154951111
C,0,3.9958779773,-1.1530067245,-0.0278199924
H,0,2.2051911942,-2.3450109751,-0.0528681007
C,0,4.5528555388,0.1102900362,0.009539298
H,0,5.6276147308,0.2611734359,0.0186253484
O,0,4.1930489315,2.4518243962,0.0277187269
H,0,3.9570175738,2.9382420611,1.0107010552
O,0,4.7474157119,-2.2891526499,-0.0586614265
H,0,5.6907205627,-2.0674530103,-0.0512965929
O,0,3.6586157262,3.1875233978,2.2150266973
O,0,2.996586632,2.0552715281,2.5918324312
H,0,2.0543179736,2.2319033273,2.4059591804

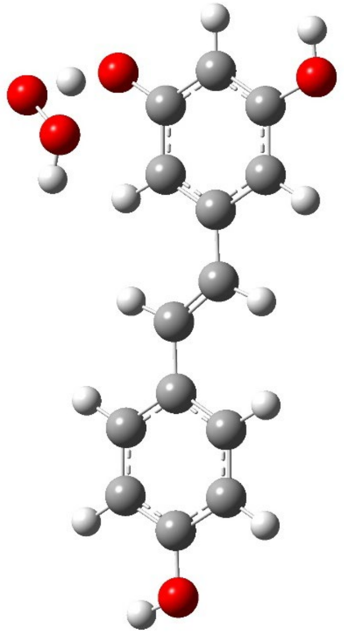

19(3)-TS
Charge = 0 Multiplicity = 2
C,0,-4.8585206241,-0.1732403882,0.1034926068
C,0,-3.9966567021,-1.2742039383,0.1275246782
C,0,-2.6247420682,-1.0775675361,0.0631247558
C,0,-2.0782553516,0.2153390471,-0.0256530328
C,0,-2.9656885155,1.3006558079,-0.057557345
C,0,-4.3443103685,1.1185709143,0.0052104269
H,0,-4.4140535166,-2.2736628148,0.1992611259
H,0,-1.9747609049,-1.94698937,0.0841036349
H,0,-2.5674459006,2.3094195435,-0.1296862962
H,0,-5.0193601805,1.9696061543,-0.017354206
O,0,-6.2038873371,-0.4231156979,0.179586263
H,0,-6.6973393027,0.4105304869,0.1831374879
C,0,-0.6352222039,0.484285793,-0.0752780713
H,0,-0.3797902889,1.5388028873,-0.1691488596
C,0,0.346272083,-0.4307330766,-0.0052186288
H,0,0.0993836913,-1.4863216703,0.0894253957
C,0,1.7893028068,-0.1462238081,-0.041683441
C,0,2.3129198325,1.1533437201,-0.1174896558
C,0,2.6806558463,-1.2210650456,0.0117516606
C,0,3.7006357717,1.368803049,-0.1356702919
H,0,1.6662045362,2.0237646365,-0.1595974783
C,0,4.0778791227,-0.9991063079,-0.0133009749
H,0,2.3198737159,-2.2435395109,0.0728371468
C,0,4.59337246,0.3117735808,-0.0746597173
H,0,5.6668546297,0.4733007367,-0.084967197
O,0,4.1106189814,2.6688591932,-0.2083709951
H,0,5.0785702966,2.7174429803,-0.2103207574
O,0,4.9123659817,-2.0354197536,0.0088200904
H,0,4.9427394947,-2.4530137794,1.0493409542
O,0,4.8574755098,-2.6282994622,2.2977578164
O,0,3.8769195216,-1.7447003033,2.6396731007

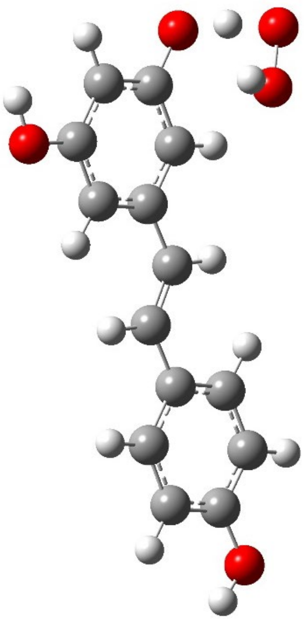

H,0,4.3473549831,-0.9300320675,2.9025858041

A-20(1)-TS

Charge = 0 Multiplicity = 2

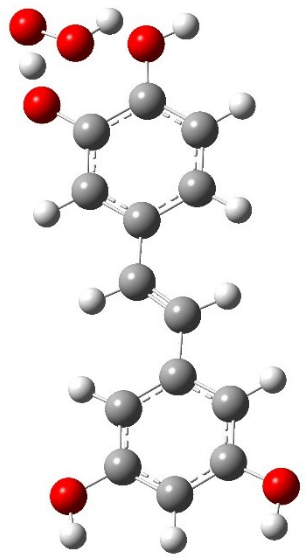

C,0,-4.479030261,-0.2438222463,-0.1450282306
C,0,-3.6014634461,-1.3333342774,-0.192067223
C,0,-2.2383422672,-1.1230615249,-0.1150529531
C,0,-1.694898666,0.1841062342,0.0026285024
C,0,-2.5719057667,1.261896415,0.0416609253
C,0,-3.962360817,1.0753342769,-0.0204678626
H,0,-4.0083305704,-2.3358879132,-0.2854610688
H,0,-1.5814669137,-1.9857307133,-0.1468014439
H,0,-2.1995206814,2.2786135406,0.1293798544
O,0,-4.8003711961,2.1092238407,0.0665289534
H,0,-5.3748906899,2.1803926071,-0.8418701332
O,0,-5.8200949013,-0.3620097778,-0.2245520842
H,0,-6.0781138583,-1.2921566691,-0.3297924221
C,0,-0.2529861516,0.4440339982,0.0682114453
H,0,0.0100349055,1.493154943,0.1892721626
C,0,3.0248442698,-1.321486159,-0.0817379964
C,0,4.4048651069,-1.1292522926,-0.0479173604
C,0,4.9512381194,0.1434198858,0.0950243259
C,0,4.0817452145,1.2312207711,0.2034005856
C,0,2.7011981985,1.0671171433,0.1722969995
C,0,2.1646232009,-0.2214513135,0.0280977489
H,0,2.6281897042,-2.3255667569,-0.1959045183
H,0,6.0282753934,0.2880982703,0.1197108131
H,0,2.0708626243,1.945617575,0.256035923
C,0,0.7176340954,-0.4819827227,-0.02167663
H,0,0.4556321454,-1.530435514,-0.1476702981
O,0,4.559927288,2.5095685505,0.3417690833
H,0,5.5284379418,2.5059734115,0.336023669
O,0,5.1976425747,-2.2414865241,-0.1643671313
H,0,6.1316350741,-1.987737435,-0.1351039023
O,0,-5.7782482731,2.1334697355,-2.1251080361
O,0,-5.0321371516,1.1054864548,-2.6197394639
H,0,-5.5430022454,0.2990321864,-2.4079482336

B-20(1)-TS

Charge = 0 Multiplicity = 2

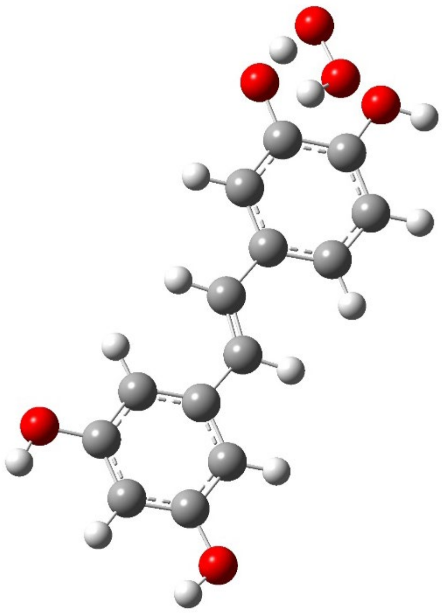

C,0,-4.5352468699,-0.3249044129,-0.0598109665
C,0,-3.6438042899,-1.4040587599,-0.0996613297
C,0,-2.282771679,-1.1751688225,-0.0427380734
C,0,-1.753361608,0.1396947491,0.0482690861
C,0,-2.6435193027,1.2076263498,0.0807040204
C,0,-4.0313726904,1.0014191403,0.0369124527
H,0,-4.038617999,-2.4130830659,-0.1729347344
H,0,-1.6154039935,-2.0299915648,-0.0694361416
H,0,-2.2828794591,2.2304547536,0.1444737693
O,0,-4.8874939617,2.0248950097,0.0979719582
H,0,-5.4189493192,2.071697562,-0.8310049513
O,0,-5.8720567277,-0.4571688546,-0.1115803677
H,0,-6.1223368791,-1.3882614371,-0.2242789496
C,0,-0.3136747186,0.4173276278,0.0874957674
H,0,-0.0604856294,1.4721317948,0.177377877

C,0,2.980892478,-1.3150372503,-0.0831006348
C,0,4.3590774924,-1.1076018994,-0.0820098045
C,0,4.8942055273,0.1730941936,0.0282001133
C,0,4.0151296335,1.2530659152,0.137990417
C,0,2.6360753403,1.0737908719,0.138049012
C,0,2.1108323276,-0.2226489116,0.0257486157
H,0,2.593198793,-2.325127796,-0.1721899686
H,0,5.9698554088,0.3298581682,0.0276920362
H,0,1.9976047898,1.9464056247,0.2219238164
C,0,0.6662074932,-0.4998650282,0.0075077429
H,0,0.4142516135,-1.5541197919,-0.0867920098
O,0,4.4823335515,2.5382542532,0.2475616371
H,0,5.4506654907,2.5439117302,0.2274293637
O,0,5.1612175479,-2.2134135073,-0.1967451006
H,0,6.092895282,-1.9496096195,-0.1951794259
O,0,-5.725900941,1.9542086373,-2.1567595355
O,0,-4.8674734556,0.9654541477,-2.53086998
H,0,-4.0631642456,1.4250731928,-2.8400997114

20(2)-TS
Charge = 0 Multiplicity = 2
C,0,-4.5699893772,-0.7724334892,-0.100805524
C,0,-3.6167535936,-1.797624374,-0.2495199541
C,0,-2.2693184313,-1.5076814397,-0.2152820652
C,0,-1.8229076025,-0.1750861698,-0.0232074529
C,0,-2.768472302,0.8468559304,0.1309232548
C,0,-4.1268942116,0.5594038714,0.0954045145
H,0,-3.9729561945,-2.8125075838,-0.3965677762
H,0,-1.5530176066,-2.3120982329,-0.3394183203
H,0,-2.4501444657,1.8740862818,0.2821855923
O,0,-5.0298928246,1.5528355841,0.258754798
H,0,-5.9260302343,1.1844782168,0.1698566192
O,0,-5.8902011961,-1.0057272266,-0.170619841
H,0,-6.2748056712,-1.1043820987,0.8163293734
C,0,-0.406741521,0.1908896122,0.0239625802
H,0,-0.2187145517,1.254094683,0.1597600314
C,0,2.9974079394,-1.3177202247,-0.1571567965
C,0,4.3578898788,-1.0156586169,-0.1179467015
C,0,4.7995638465,0.2953761217,0.0415092758
C,0,3.8454818958,1.3086804228,0.1603532809
C,0,2.4832311898,1.0348835745,0.123411092
C,0,2.0519669829,-0.2909167273,-0.0358532582
H,0,2.682257096,-2.3490998743,-0.2813810559
H,0,5.8612428182,0.5270621899,0.0723652262
H,0,1.7846649547,1.8584889731,0.219936255
C,0,0.6301520776,-0.6615013598,-0.0827466493
H,0,0.4450383519,-1.7256637743,-0.2147679254
O,0,4.2182083668,2.6186832002,0.3188876657
H,0,5.1835900814,2.6909619804,0.3361619052
O,0,5.23746005,-2.0598119557,-0.2418927921
H,0,6.1492348951,-1.7350230159,-0.2033553301
O,0,-6.3649978692,-1.1186407179,2.193805744
O,0,-5.1023118083,-0.7625150728,2.5256946846
H,0,-4.6061709633,-1.600545688,2.612815549

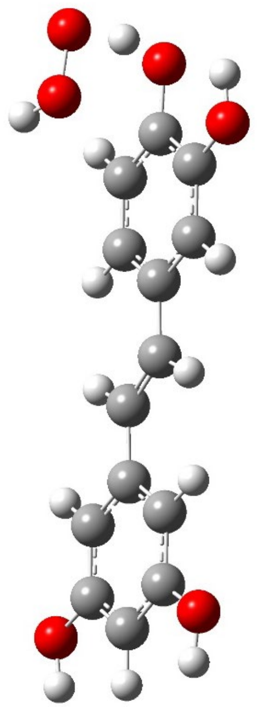

20(3)-TS

Charge = 0 Multiplicity = 2

C,0,-4.4304765216,-0.4288247222,0.0711387466
C,0,-3.5840552039,-1.4419106962,-0.3691632453
C,0,-2.2077214721,-1.2380349778,-0.404201168
C,0,-1.6557848032,-0.016223709,0.0084065452
C,0,-2.522047086,1.001231517,0.4389531292
C,0,-3.893973254,0.8000241412,0.4729938098
H,0,-4.0146622399,-2.3864067819,-0.6900231072
H,0,-1.5694330677,-2.0373798548,-0.7658929443
H,0,-2.1272488088,1.962446837,0.7568801259
O,0,-4.7142824775,1.8119246166,0.9025816324
H,0,-5.6374100751,1.5182018611,0.8529635022
O,0,-5.7947629617,-0.5384716517,0.1378210591
H,0,-6.0800240581,-1.4103673778,-0.1728748293
C,0,-0.2104230381,0.2521426522,0.0118205364
H,0,0.0616700606,1.2898623987,0.202188565
C,0,3.0989891122,-1.4267639052,-0.0164765808
C,0,4.4900262882,-1.1969449221,0.026954329
C,0,5.0067776262,0.0807982517,-0.068155916
C,0,4.1085859211,1.1544766178,-0.2438911131
C,0,2.71151326,0.9308329714,-0.296597202
C,0,2.2002676587,-0.3693170109,-0.1599167642
H,0,2.7384923873,-2.4475673027,0.0716432673
H,0,6.0752461466,0.2674720765,-0.0260400437
H,0,2.0662745701,1.7841399279,-0.4745358
C,0,0.7588555256,-0.6608011036,-0.1673507113
H,0,0.503767503,-1.710705448,-0.2985500485
O,0,4.5773630379,2.3915617026,-0.3680651251
H,0,4.2057583492,3.0102793587,0.487467149
O,0,5.2745591362,-2.3004658837,0.1786290404
H,0,6.2093794807,-2.047518934,0.215481526
O,0,3.7860319272,3.4342693799,1.6085662309
O,0,2.9536353655,2.4246272304,2.0016701622
H,0,3.4971797114,1.8560087407,2.5804672426

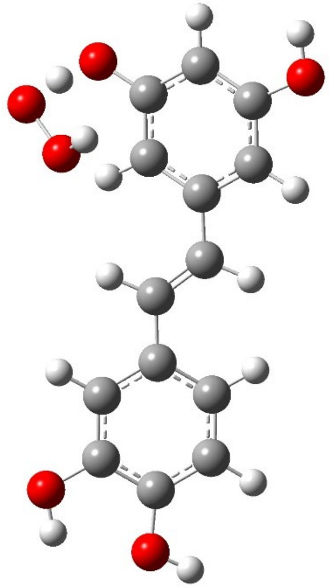

20(4)-TS

Charge = 0 Multiplicity = 2

C,0,-4.5804036801,-0.511502589,-0.0012810645
C,0,-3.6772930558,-1.5644113035,0.1119586384
C,0,-2.307952378,-1.3178734795,0.1045661592
C,0,-1.8185223937,-0.0087197465,-0.018945234
C,0,-2.7407815924,1.0437074613,-0.1331042547
C,0,-4.1061720943,0.7991487941,-0.1250578165
H,0,-4.0598157316,-2.5768354945,0.2084977737
H,0,-1.6266886537,-2.1568262895,0.1984140101
H,0,-2.3958510694,2.0697592438,-0.2285046489
O,0,-4.9804094877,1.850194372,-0.2377972597
H,0,-5.8898495237,1.5140370351,-0.2102564618
O,0,-5.9424935626,-0.6622913696,0.000018232
H,0,-6.179426132,-1.5950009491,0.112776035
C,0,-0.3863114464,0.3224671144,-0.0294550768
H,0,-0.1713003348,1.3879573689,-0.0957400301

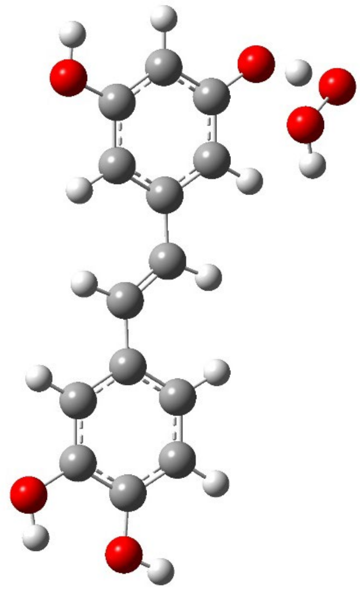

C,0,2.9918664414,-1.2565824553,0.0523913779
C,0,4.3810670889,-0.9803802785,0.065830507
C,0,4.8456124704,0.3502747358,0.0691372138
C,0,3.9127089341,1.3728434013,0.037765028
C,0,2.5330825268,1.1044783039,0.0270503981
C,0,2.0595108327,-0.2147883895,0.0359162848
H,0,2.6695232614,-2.294298255,0.049619004
H,0,5.9118080479,0.5534757285,0.0872363954
H,0,1.8540042747,1.9508511639,0.0153857912
C,0,0.6283624118,-0.5557337065,0.0346142992
H,0,0.4208288705,-1.6225855585,0.0897552705
O,0,4.2704171932,2.6891474208,0.0259118153
H,0,5.2353909978,2.777729912,0.0449010416
O,0,5.2536753779,-1.9830520425,0.0615111088
H,0,5.2035392576,-2.5168042007,1.0469284324
O,0,5.0287378973,-2.8437225581,2.2556472004
O,0,4.0753282344,-1.9581478488,2.6649884388
H,0,3.2216820176,-2.3996825413,2.4917443912

Cartesian coordinates of the optimized transition states between a phenol and OOH studied at the M06-2X(SMD)/6-31++G(d,p) level of theory in PE.

1-TS

Charge = 0 Multiplicity = 2

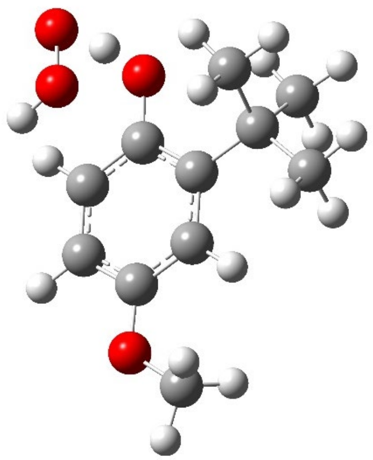

C,0,-1.9353184503,1.6567708892,0.0939922922
C,0,-2.0052346974,0.2528486392,0.0947743195
C,0,-0.8320639729,-0.5168388188,0.1021964882
C,0,0.4333857886,0.0706486341,0.1226330394
C,0,0.4967313289,1.4952618764,0.1189672942
C,0,-0.6973986538,2.2580471789,0.0976318614
H,0,-2.853906783,2.2340809755,0.0804919164
H,0,-0.9142815135,-1.5947747403,0.0975523264
H,0,-0.6029554772,3.3407703819,0.0804814573
O,0,1.6605429378,2.144907589,0.1012443142
H,0,1.8328277075,2.5776978522,1.0691129751
O,0,-3.24669537,-0.2706590687,0.0856779804
C,0,-3.3899579635,-1.686141995,0.0867281606
H,0,-4.4627124881,-1.8754549059,0.078904464
H,0,-2.9338997405,-2.1269922918,-0.8057991703
H,0,-2.9472922735,-2.1241987694,0.9873430783
C,0,1.7066080612,-0.7799037183,0.1292326348
C,0,1.3925065439,-2.2812804233,0.1001298552
H,0,0.8166682499,-2.5978300277,0.9767206027
H,0,0.8435435578,-2.5688582325,-0.8034085472
H,0,2.334729546,-2.8381288763,0.1057698926
C,0,2.5539845024,-0.4559789727,-1.1162280434
H,0,2.8758739961,0.5863626002,-1.1231013727
H,0,3.4436806954,-1.0956532103,-1.1261118582
H,0,1.9854017966,-0.6531889984,-2.0320619494
C,0,2.517279694,-0.4970569674,1.4075235924
H,0,3.4210433815,-1.117027572,1.4057314806
H,0,2.8206020402,0.5485416776,1.4793715436
H,0,1.9330988366,-0.7480133373,2.2990773849
O,0,1.775158626,2.8642634127,2.3826746581

O,0,0.810013683,2.0082271633,2.8078923449
H,0,-0.0280460398,2.4986568358,2.7070750038

2-TS

Charge = 0 Multiplicity = 2
C,0,-1.2493417496,1.7483527928,-0.0133868854
C,0,-0.013178677,1.0807244472,-0.0133690115
C,0,0.0610406827,-0.3447506773,0.0054542805
C,0,-1.1431348325,-1.0280279233,0.0187922263
C,0,-2.3977324599,-0.3726249494,0.0131598455
C,0,-2.4345940473,1.0318481116,0.0034726282
H,0,-1.2856758771,2.8306005241,-0.0300377779
H,0,-3.3952664469,1.5378088329,-0.0048037008
O,0,-3.5261932196,-1.0791420148,-0.0216937431
H,0,-3.7816268587,-1.3995047662,0.9742888181
O,0,1.15661845,1.7515087368,-0.0305344376
C,0,1.1425004422,3.1731393403,-0.0578057816
H,0,0.6573846958,3.5790443896,0.8358889922
H,0,2.1887281883,3.476869893,-0.0728701275
H,0,0.6413283017,3.5439816958,-0.957789631
H,0,-1.1682579611,-2.1115208646,0.0362041291
C,0,1.4070182944,-1.0830402267,0.0151474361
C,0,1.2086020035,-2.6043297098,0.0390039973
H,0,0.669293839,-2.9333916575,0.9335871455
H,0,0.6697444268,-2.961660812,-0.8450338958
H,0,2.1908949031,-3.0870481031,0.0466315376
C,0,2.2119656053,-0.7435072636,-1.2533542777
H,0,2.4561430459,0.3182861156,-1.3123295675
H,0,3.1489570926,-1.3117573114,-1.2521084639
H,0,1.6494702142,-1.0221950822,-2.1513735474
C,0,2.2109086404,-0.7042750901,1.2732684536
H,0,3.1490097936,-1.2704703113,1.2894667363
H,0,2.45300337,0.3593939174,1.300183845
H,0,1.6479990936,-0.9573838096,2.1785949076
O,0,-3.8772178664,-1.5044738398,2.3044030005
O,0,-2.7458430634,-0.8618550777,2.698439288
H,0,-3.0323024136,0.0489895232,2.899199251

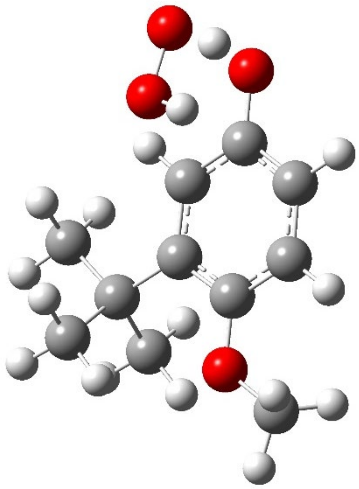

3-TS

Charge = 0 Multiplicity = 2
C,0,0.1225260553,-0.5795184465,-0.089643574
C,0,0.448898062,0.7947912346,-0.078964844
C,0,2.8110072914,0.180789251,0.0402794037
O,0,4.0886663227,0.5534697869,0.0675061607
H,0,4.4650504984,0.4536013939,1.0714226164
C,0,-0.6438733747,1.8327629466,-0.1606795255
H,0,-0.8040862154,2.2781926362,0.8287895397
H,0,-0.3258549013,2.6446804957,-0.8215252142
C,0,-1.9345200352,1.2003390552,-0.6737741337
H,0,-1.8428047692,0.9802155589,-1.7442522326
H,0,-2.7801264358,1.8830572129,-0.546550188
C,0,-2.2384756883,-0.1084890272,0.0555728829
C,0,-3.4559218141,-0.7976419889,-0.5365301891
H,0,-3.6067564552,-1.7740917057,-0.0670284695
H,0,-4.3477496856,-0.1878417064,-0.3646829279

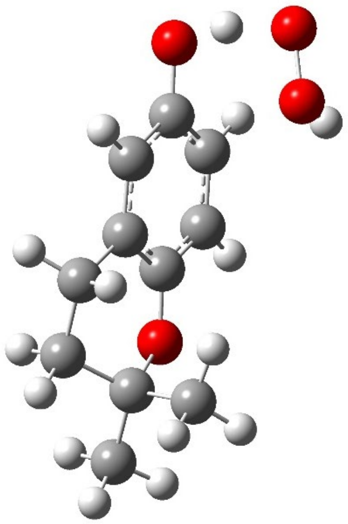

H,0,-3.3312402614,-0.9416247048,-1.6137758882
C,0,-2.3935463042,0.0809695911,1.5624081401
H,0,-3.1833439957,0.8099863002,1.7692723115
H,0,-2.6635599948,-0.8692739175,2.0319479171
H,0,-1.4668311591,0.4365892823,2.0225853735
O,0,-1.1428008315,-1.0382942349,-0.1654500628
C,0,1.1318195485,-1.5610521336,-0.0508163538
H,0,0.8371375012,-2.6054941108,-0.0738572951
C,0,2.4588219076,-1.1905717955,0.0096330857
H,0,3.2498161423,-1.9353944732,0.028117905
C,0,1.7879408891,1.1490290874,-0.0047337692
H,0,2.0733496218,2.1981320658,0.0071827094
O,0,4.5762791785,0.251628395,2.3914477437
O,0,3.2736636626,0.1144257625,2.7540121711
H,0,3.0954560201,-0.8437884414,2.7031453273

4-TS

Charge = 0 Multiplicity = 2

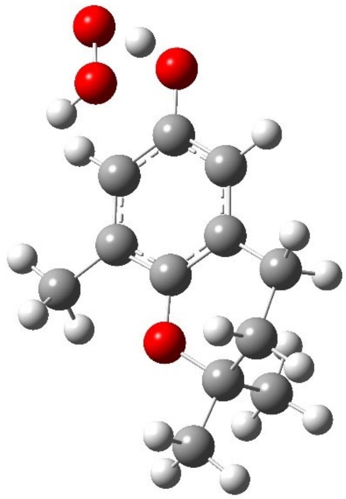

C,0,-0.1015495936,0.3624444087,-0.1079053277
C,0,-0.3878055714,-1.0180680902,-0.0922234922
C,0,-2.7598087875,-0.4733881968,0.0038274797
O,0,-4.0257282502,-0.8765697174,0.0984684335
H,0,-4.5052390171,-0.744163641,-0.8531219289
C,0,0.7349431349,-2.0252592443,-0.1566031168
H,0,0.8970573832,-2.4610647983,0.836857877
H,0,0.445669533,-2.849142307,-0.8159147283
C,0,2.013853478,-1.3625665449,-0.6593509224
H,0,1.928137431,-1.1503329924,-1.7319398047
H,0,2.8760150566,-2.0217344412,-0.5192459823
C,0,2.2726185994,-0.0426212315,0.066632046
C,0,3.4769600484,0.6787666641,-0.5138614072
H,0,3.592469483,1.6620500891,-0.0485114825
H,0,4.3841854445,0.0968855256,-0.3263308685
H,0,3.3627845389,0.8125263448,-1.593629034
C,0,2.4146094831,-0.220284005,1.5762688626
H,0,3.2209104049,-0.9269505265,1.7967974383
H,0,2.6533894336,0.7393203765,2.0437498979
H,0,1.4918329219,-0.5978117635,2.0265952812
O,0,1.1537199699,0.853756333,-0.1714252087
C,0,-1.1315565942,1.3374120719,-0.0870437263
C,0,-2.4432450836,0.906379497,-0.0237626939
H,0,-3.2569714489,1.62712872,0.0179774654
C,0,-1.7165110739,-1.416293405,-0.0451785465
H,0,-1.9725718471,-2.4727357245,-0.0505375923
C,0,-0.7674339019,2.7948897204,-0.112670596
H,0,-0.1376781575,3.0546112952,0.7441565769
H,0,-0.1966931109,3.0426063205,-1.0133333237
H,0,-1.666687341,3.4139473701,-0.08634522
O,0,-4.7404136862,-0.5306615393,-2.1616636242
O,0,-3.4786141411,-0.4852415666,-2.6646026043
H,0,-3.2111161181,0.4508883385,-2.5957102372

5-TS

Charge = 0 Multiplicity = 2

C,0,0.1708366662,0.6602193326,-0.0803136995
C,0,-0.3730462855,-0.635306764,-0.1201216482
C,0,-2.5877216701,0.3482468512,0.0461624527
O,0,-3.91552288,0.2344832875,0.1608350424
H,0,-4.32094666,-0.1261554151,-0.741424461
C,0,0.5395040902,-1.8299746566,-0.2737236642
H,0,0.5979975583,-2.3801002381,0.6748585859
H,0,0.112779084,-2.5244904093,-1.0047022055
C,0,1.9315145567,-1.396868782,-0.7235522704
H,0,1.9096306282,-1.1114425341,-1.7822889889
H,0,2.6472604999,-2.2178379501,-0.6166221329
C,0,2.421459315,-0.1933148265,0.0783124025
C,0,3.756506346,0.311266189,-0.4419491179
H,0,4.0486057905,1.226085551,0.0821486112
H,0,4.5298792578,-0.4451051721,-0.2784915298
H,0,3.6960351145,0.5248566766,-1.5132114832
C,0,2.4887583036,-0.4752953706,1.5775610887
H,0,3.1455845981,-1.3296215005,1.7700684466
H,0,2.8876417889,0.3971100393,2.1033073481
H,0,1.5007712592,-0.6999196341,1.9901913231
O,0,1.5004928532,0.9068660988,-0.132321591
C,0,-0.6469499448,1.8157014289,-0.0059423944
C,0,-2.0143313199,1.6377565815,0.045724441
H,0,-2.6806950933,2.4938913296,0.1091729963
C,0,-1.7607148938,-0.7990735069,-0.036712338
C,0,-2.3788014575,-2.1670802665,-0.0611635675
H,0,-2.4016827368,-2.5643702811,-1.0832494012
H,0,-1.809333507,-2.8694095581,0.554335539
H,0,-3.4048128213,-2.1359751025,0.3083606584
C,0,-0.0100070073,3.1762915479,0.0202732197
H,0,0.6623758665,3.2774030914,0.8781784875
H,0,0.5925310854,3.3475691422,-0.8775839663
H,0,-0.7742499316,3.9541856221,0.081913989
O,0,-4.5218812122,-0.3934866039,-2.0951906346
O,0,-3.2426518605,-0.3653942142,-2.5462362243
H,0,-3.1166511605,0.5390771769,-2.8906559932

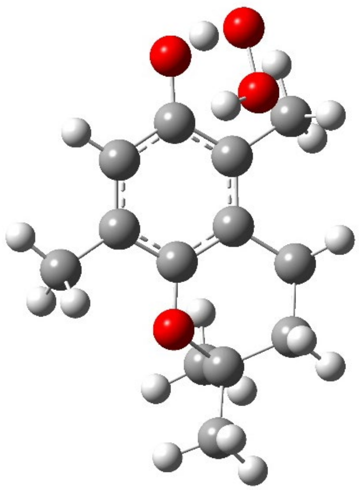

6-TS

Charge = 0 Multiplicity = 2

C,0,0.1658562844,0.2001904112,-0.1026196488
C,0,0.1016573854,-1.2101326915,-0.054254774
C,0,-2.3255633553,-1.0307694076,0.0321289201
O,0,-3.5007139033,-1.6553996121,0.1596529611
H,0,-4.0257759512,-1.6299549432,-0.7582140526
C,0,1.3642853556,-2.0375383742,-0.0839064177
H,0,1.5807177176,-2.4138773085,0.9236307065
H,0,1.2098917952,-2.9160702376,-0.7179535557
C,0,2.5352862223,-1.2056798766,-0.5965657671
H,0,2.434685974,-1.0402463072,-1.6761730837
H,0,3.4853604196,-1.7214727843,-0.4264806888
C,0,2.5750702681,0.1595461794,0.0893056407
C,0,3.668101584,1.0372178181,-0.4964880859
H,0,3.626047195,2.0397090503,-0.0604985659

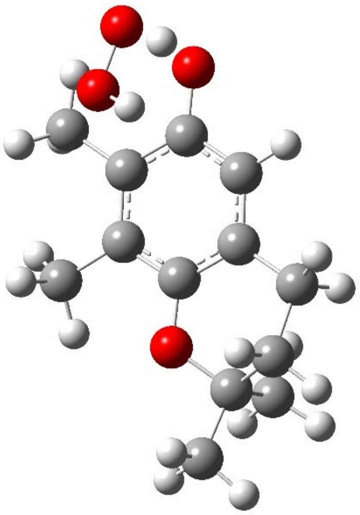

H,0,4.6492314295,0.6044371769,-0.2795362818
H,0,3.5539402467,1.1215241087,-1.5812411913
C,0,2.714810032,0.0508511095,1.6060863357
H,0,3.6129632914,-0.5203403266,1.8617074676
H,0,2.7994515826,1.0493827525,2.0446420004
H,0,1.8503212988,-0.4461324329,2.0561327928
O,0,1.3408967407,0.866020915,-0.1988895521
C,0,-0.9988657832,0.9997179808,-0.0944914796
C,0,-2.250736161,0.3880175091,-0.0395074022
C,0,-1.1489886295,-1.8003475172,0.0077285895
H,0,-1.2437222023,-2.8828545511,0.0468570067
C,0,-0.8713840404,2.4987806564,-0.1681060604
H,0,-1.6517550825,2.9887711072,0.4188058619
H,0,0.1004581775,2.826955378,0.2020235298
H,0,-0.9670342173,2.8500214056,-1.2030324858
C,0,-3.5116745854,1.204791924,-0.0618000667
H,0,-4.3905613791,0.569619694,-0.1734833337
H,0,-3.624991802,1.7763002922,0.8670122579
H,0,-3.4959132444,1.9211275159,-0.8888877505
O,0,-4.3371036533,-1.5449640827,-2.1013090451
O,0,-3.3216972127,-0.7595316028,-2.5422286043
H,0,-2.6510063074,-1.3817888589,-2.8818885371

7-TS

Charge = 0 Multiplicity = 2

C,0,0.2641543902,0.5139335555,-0.0784818225
C,0,0.0035277824,-0.8660855686,-0.1300742303
C,0,-2.3649933904,-0.3460773914,0.0249991992
O,0,-3.6362745156,-0.7605921452,0.1322248777
H,0,-3.9508635995,-1.1777255367,-0.7721754635
C,0,1.1407917575,-1.8486841891,-0.288706048
H,0,1.3074860734,-2.3846665403,0.6552120101
H,0,0.8681572601,-2.6082917649,-1.0286482058
C,0,2.4171587705,-1.136666915,-0.7259742254
H,0,2.3437981063,-0.8545661479,-1.7833473287
H,0,3.2857205078,-1.7937852191,-0.6177364996
C,0,2.642472665,0.1371009773,0.0854775892
C,0,3.8501852656,0.9081226012,-0.4197534526
H,0,3.9419623528,1.8612210665,0.1096223353
H,0,4.7614903841,0.3270423076,-0.2498387262
H,0,3.7574008306,1.1097714853,-1.4910413329
C,0,2.7559000927,-0.1349093074,1.583956612
H,0,3.5718172976,-0.8386074914,1.777497576
H,0,2.9648734775,0.7971801341,2.1171722301
H,0,1.8318488545,-0.5588036081,1.9878745156
O,0,1.5185409637,1.0257251519,-0.1303369109
C,0,-0.7685592237,1.4772674315,0.0029436146
C,0,-2.0913703474,1.0483939126,0.031602215
C,0,-1.3208888543,-1.2999829943,-0.0532862112
C,0,-1.6557594569,-2.7642513171,-0.0867350819
H,0,-1.6621514666,-3.1367135709,-1.1187263199
H,0,-0.9229079097,-3.351498454,0.4730083088
H,0,-2.6437641729,-2.9466348555,0.3383997094
C,0,-0.384106379,2.9322277134,0.049633564
H,0,0.417924567,3.093638586,0.7750277848

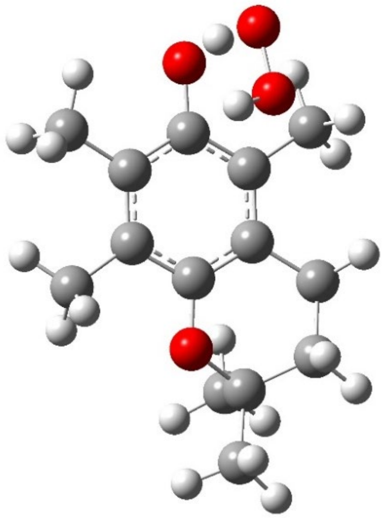

H,0,-0.0040657613,3.2711328446,-0.9213206618
H,0,-1.2297439916,3.5636696748,0.3231721124
C,0,-3.2357477818,2.0268124589,0.0862267983
H,0,-3.2625055744,2.5517534151,1.0479703732
H,0,-3.1428906299,2.7857366585,-0.6967076085
H,0,-4.1870091821,1.5099442554,-0.038906928
O,0,-4.0876583702,-1.4425301426,-2.1575918573
O,0,-2.8446952087,-1.1244248577,-2.5902398025
H,0,-2.914975313,-0.205062053,-2.9108495981

8-TS

Charge = 0 Multiplicity = 2
C,0,1.4345145117,0.0093091799,0.0511906857
C,0,0.7157441023,1.2224728798,0.0866971393
C,0,-0.664713861,1.2259748479,0.0568920767
C,0,-1.385253212,0.0119205481,0.0030839829
C,0,-0.6679336129,-1.2050101343,-0.0237758671
C,0,0.7141826053,-1.2035736698,-0.0068317578
H,0,1.2733482243,2.1528811271,0.1344473201
H,0,-1.2112347822,2.164612905,0.0806292315
H,0,-1.2155898869,-2.142431763,-0.066916046
H,0,1.2689065794,-2.137523443,-0.0455609694
O,0,2.7731491204,0.0139190186,0.0328378735
H,0,3.1316834573,-0.2385429061,0.9872203743
N,0,-2.7547702235,0.0128146942,0.0218155073
H,0,-3.2385955094,0.8766606926,-0.1794939315
H,0,-3.2422313886,-0.8299186207,-0.2485008925
O,0,3.2015028031,-0.5360479476,2.356143769
O,0,1.948514717,-0.2153126491,2.7550452649
H,0,1.4253883459,-1.0326561396,2.6460537991

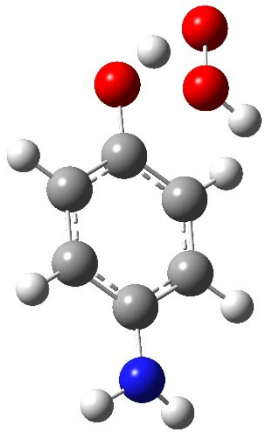

9-TS

Charge = 0 Multiplicity = 2
C,0,-2.2009247495,-0.1625796103,-0.04319807
C,0,-1.5879954314,1.0574836128,0.3072360851
C,0,-0.2271934375,1.1322079603,0.5413230993
C,0,0.5981591789,-0.018336601,0.4134904995
C,0,-0.0273972633,-1.2416029027,0.0482683196
C,0,-1.390974509,-1.3076299694,-0.1614793138
H,0,-2.2048945944,1.9496319524,0.3850596112
H,0,0.2061037113,2.0881895665,0.8090506866
H,0,0.561755715,-2.1439353506,-0.0604521749
H,0,-1.8615674668,-2.2496266091,-0.4267029969
N,0,1.9419914194,0.0499337076,0.633346111
O,0,-3.5167835472,-0.2326477676,-0.295292438
H,0,-4.0330594494,-0.0683228302,0.5892030556
C,0,2.55887003,1.3136058944,0.9957192274
H,0,3.6247848196,1.1549608323,1.1527863744
H,0,2.4325604182,2.0635322812,0.2048570765
H,0,2.131848612,1.7118358758,1.9236958428
C,0,2.7699049492,-1.1301755763,0.4562431594
H,0,3.8081053922,-0.8669634577,0.6526072705
H,0,2.4790623429,-1.9287216092,1.1491627608
H,0,2.6995852528,-1.5157111799,-0.568110044
O,0,-4.338914301,0.0313844397,1.9947071524

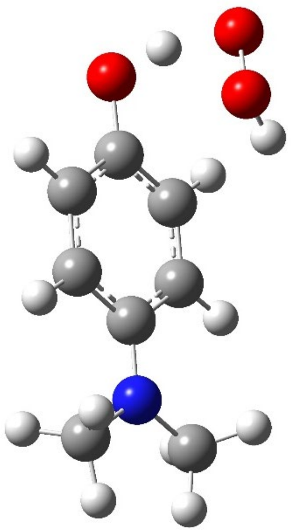

O,0,-3.1986603316,-0.4262568743,2.5586953258
H,0,-2.5911451905,0.3385255854,2.5744110498

10-TS

Charge = 0 Multiplicity = 2

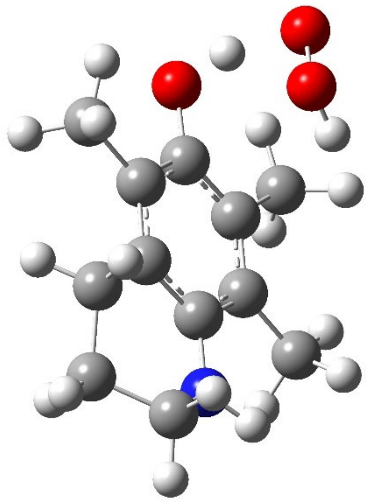

C,0,1.762222406,-0.6243583583,-0.0073956423
C,0,0.5763346044,-1.3556593927,0.0011175673
C,0,-0.6700658445,-0.6722060641,0.0029002187
C,0,-0.7215240908,0.7424726096,-0.0153423561
C,0,0.4650616668,1.4719614157,0.0160616708
C,0,1.7046446028,0.7932695282,0.0116217319
C,0,0.4388149755,2.9757708102,0.0589923455
H,0,0.1426784582,3.393432155,-0.9110300217
H,0,-0.2812636104,3.3340365651,0.8011628473
H,0,1.4236372112,3.3708317568,0.3071294728
C,0,0.5653703907,-2.8624822822,-0.0073878007
H,0,0.0800992805,-3.2657943555,0.8911809748
H,0,0.0146171507,-3.2475487051,-0.8746876639
H,0,1.5712272019,-3.2789467991,-0.0431222292
C,0,3.1081864066,-1.3081974284,-0.0319767972
H,0,3.2862530544,-1.8863810831,0.882454327
H,0,3.1794155799,-2.0038256176,-0.8739223369
H,0,3.9114789317,-0.578972287,-0.1326440031
O,0,2.8468028902,1.5144963719,-0.0147596562
H,0,3.2915724072,1.4661309539,0.9053722311
C,0,-2.0589256826,1.4545381302,-0.0517466307
H,0,-2.2934031294,1.8467049521,0.9480114353
H,0,-1.9923334602,2.3241263925,-0.7134863778
C,0,-3.1882549928,0.5361158647,-0.5154149216
H,0,-3.0900346571,0.3313857838,-1.5878481585
H,0,-4.1586585013,1.0131071556,-0.3520473785
C,0,-3.121443736,-0.7800988911,0.2427702451
H,0,-3.9037212745,-1.4683408842,-0.0863925331
H,0,-3.2616625143,-0.5937427919,1.3191356581
N,0,-1.8344340062,-1.40758425,-0.0137527767
H,0,-1.7613443933,-2.3839808801,0.2337162214
O,0,3.4688204338,1.2173331435,2.3703986291
O,0,2.2033229491,0.9348978158,2.7334137451
H,0,2.0666827418,-0.0055806743,2.5043076033

11-TS

Charge = 0 Multiplicity = 2

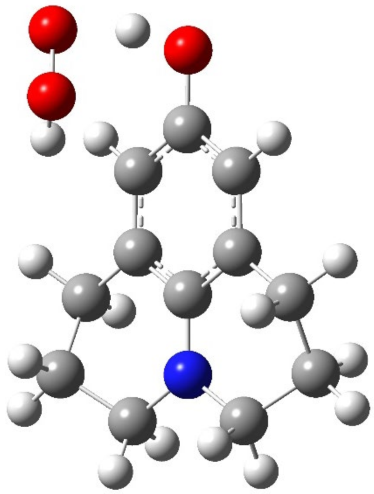

C,0,0.2038251216,-2.4796044017,-0.2402759326
H,0,-0.4451929729,-3.2967521721,0.0881038808
H,0,0.4402322019,-2.6605977443,-1.2978305159
C,0,1.5013069411,-2.4414608812,0.5622104901
H,0,2.0649783138,-3.3712738332,0.4473597073
H,0,1.27429357,-2.3162022547,1.6275605526
C,0,2.357236989,-1.2809873776,0.0819430852
H,0,2.7486984011,-1.4903303598,-0.9252950809
H,0,3.220051022,-1.1437204155,0.7444839798
C,0,2.4506527949,1.1745938951,0.044284848
H,0,2.8383843325,1.3610441946,1.0574022451
H,0,3.3126106624,0.9673523797,-0.6009687474
C,0,1.6932071988,2.3921712533,-0.4594205145

H,0,1.475811248,2.275429624,-1.5275327725
H,0,2.3236837335,3.2775467041,-0.3404679519
C,0,0.3880979688,2.5346478301,0.3182358878
H,0,-0.191196943,3.3948618074,-0.029313913
H,0,0.6179809102,2.70674507,1.3787777552
N,0,1.617069983,-0.0234018945,0.0599968299
O,0,-3.916975551,0.2043349378,-0.0110154819
H,0,-4.2782936778,-0.3522201215,0.7699901187
C,0,-1.9009341292,-1.0924335152,-0.1046971955
H,0,-2.4863063733,-2.0025962651,-0.2252990099
C,0,-1.8119007595,1.3111766469,0.1533396102
H,0,-2.3327272189,2.2601329601,0.2528947699
C,0,-0.4288313911,1.276291205,0.1695007395
C,0,0.2482860201,0.0291987289,0.0443680523
C,0,-0.5174101797,-1.1639962879,-0.0972455691
C,0,-2.5732576534,0.1384213897,0.0193039188
O,0,-4.340182333,-1.0103261273,2.1042193421
O,0,-3.1954309411,-0.6041033633,2.693976232
H,0,-2.4818369788,-1.0978409617,2.2436847294

12-TS

Charge = 0 Multiplicity = 2

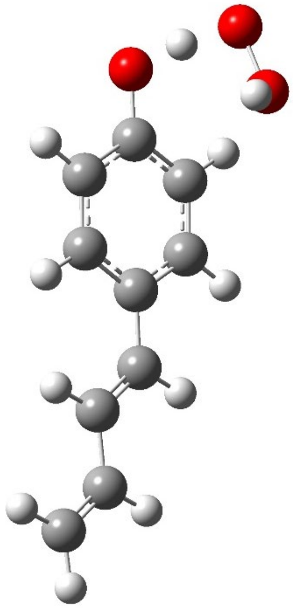

C,0,-2.2315444858,-1.396500638,0.1687511987
C,0,-2.7641399908,-0.1039301946,-0.0550489401
C,0,-1.8747729405,0.9872738202,-0.2192951727
C,0,-0.5130317419,0.793613576,-0.1412683956
C,0,0.0286314159,-0.4957906142,0.080888317
C,0,-0.8632719377,-1.5774766545,0.2325940908
H,0,-2.9159555394,-2.2305871318,0.2888392531
H,0,-2.2961255049,1.9731304321,-0.3898301363
H,0,0.1478563228,1.6469545551,-0.2549558944
H,0,-0.4599245853,-2.5720625215,0.4028546479
O,0,-4.0675399878,0.1103055438,-0.0753508975
H,0,-4.545189727,-0.4875797166,-0.8538232709
C,0,1.4601356943,-0.7539211603,0.1557250334
H,0,1.7388076414,-1.7892783478,0.3514229979
C,0,2.451557512,0.1508368981,0.0022023585
H,0,2.2266324105,1.1974044949,-0.1995334344
C,0,3.8583307582,-0.2024469598,0.0906775809
H,0,4.0905208595,-1.248036336,0.2907624317
C,0,4.8515209158,0.6861588659,-0.0598875397
H,0,4.6435506699,1.7349293348,-0.2596718789
H,0,5.8928391175,0.3890300907,0.0125408197
O,0,-4.8401658933,-1.0565438567,-1.9922368085
O,0,-3.6209802294,-1.5088845825,-2.406151897
H,0,-3.3587564541,-0.8896613072,-3.1122555736

13-TS

Charge = 0 Multiplicity = 2

C,0,-2.3162462561,1.336406857,0.0176107938
C,0,-1.4360274707,0.2341830093,0.0151802372
C,0,-1.9944709665,-1.0672314785,0.0217086595
C,0,-3.3593413551,-1.2537293332,0.0349213727
C,0,-4.2357205055,-0.140167724,0.0200778173
C,0,-3.6870866692,1.1647510588,0.018450272

H,0,-1.9013250669,2.3407945689,0.0159675072
H,0,-1.3464567726,-1.9378434935,0.0201630257
H,0,-3.7924566947,-2.2491496331,0.0401178079
H,0,-4.360658998,2.0162629519,0.0143850199
C,0,-0.0011905472,0.4909188554,0.0080743838
H,0,0.2710075362,1.5445263085,0.0084187987
C,0,0.9719228323,-0.4435192169,0.0007081592
H,0,0.6992245472,-1.4972265171,-0.0018690982
C,0,2.4165418834,-0.1912816117,-0.0066519371
C,0,2.9762737984,1.0988185046,-0.0009644823
C,0,3.2859161843,-1.2939559608,-0.0209251854
C,0,4.3552322951,1.2719648671,-0.0096150618
H,0,2.33434011,1.9743240665,0.0100498645
C,0,4.667650321,-1.1201094392,-0.0292683892
H,0,2.8672558186,-2.297367915,-0.0256393735
C,0,5.2079795798,0.1647261478,-0.0238051974
H,0,4.7696382309,2.2756667257,-0.0052824039
H,0,5.3208466058,-1.9874280544,-0.0402647095
H,0,6.2843930638,0.3059608636,-0.0305095801
O,0,-5.5402979245,-0.3405300028,-0.0329771285
H,0,-6.0397824339,0.145926742,0.8054015586
O,0,-6.3767516164,0.5431955084,2.0052651069
O,0,-5.168637909,0.8799082743,2.5446009727
H,0,-4.9655874704,0.1506820705,3.1590339895

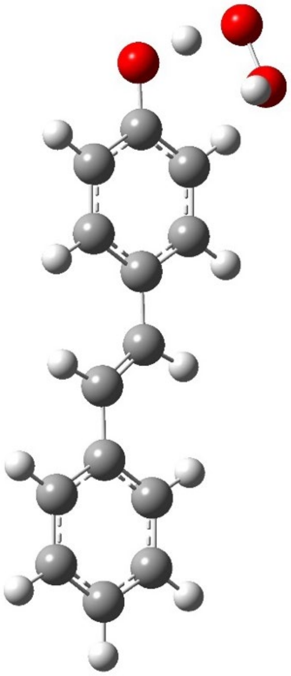

14(1)-TS
Charge = 0 Multiplicity = 2
C,0,0.5690178908,-0.9602384131,-0.0003707774
C,0,1.8775867059,-1.413001108,-0.0284093644
C,0,2.9240417646,-0.4827370685,-0.0237004306
C,0,2.6550918078,0.9088142305,0.0024250524
C,0,1.3185755991,1.3553658166,0.0320740825
C,0,0.2948099563,0.4232370344,0.0309243221
H,0,-0.2382551793,-1.6841571185,-0.0040743291
H,0,1.1080433322,2.4198997175,0.0463127007
O,0,2.1374501805,-2.7418722296,-0.0538830359
H,0,3.0951855134,-2.8887814231,-0.0656933466
O,0,4.1815226157,-0.944168411,-0.0479264487
H,0,4.7926269652,-0.1863463173,-0.0704675221
O,0,3.6970949128,1.7233500798,-0.0215110081
H,0,3.9137403537,2.0613511635,1.0289108129
C,0,-1.108576666,0.9327545135,0.0594591946
O,0,-1.3957737104,2.1097085524,0.1187267422
O,0,-2.0128042407,-0.0475126756,0.0139980458
C,0,-3.3957559733,0.3575831148,0.034439129
H,0,-3.581808606,1.010605905,-0.8247105397
H,0,-3.5792613141,0.9316094979,0.948774382
C,0,-4.246505389,-0.8937465392,-0.0201648664
H,0,-3.9994392166,-1.532191606,0.8352096795
H,0,-4.0006908626,-1.4542051403,-0.9288246574
C,0,-5.7313470397,-0.5389767171,-0.0033713636
H,0,-5.9979127305,0.0848218388,-0.862968186
H,0,-6.3472137727,-1.4412449328,-0.0404649854
H,0,-5.9959476466,0.010477718,0.9061572949
O,0,3.939415578,2.1618462301,2.284322144

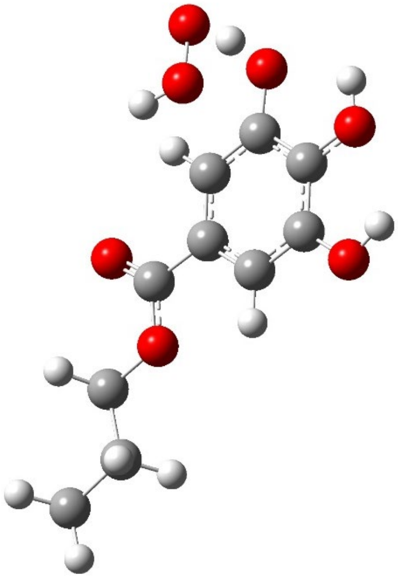

O,0,3.1213777303,1.1455120586,2.6690822847
H,0,2.2456641213,1.5604740686,2.7933386543

14(2)-TS

Charge = 0 Multiplicity = 2

C,0,0.369430116,-1.1725044558,-0.0454230602
C,0,1.6675414157,-1.6611553153,-0.0578127693
C,0,2.7793928788,-0.7870628973,0.0975065407
C,0,2.5334638956,0.5952053666,0.3093407067
C,0,1.2395932689,1.0841893726,0.3287725248
C,0,0.1717010687,0.1974652351,0.1430482937
H,0,-0.4649070115,-1.8499787582,-0.1805329407
H,0,1.0423576579,2.1395382537,0.4896395972
O,0,1.9045037188,-2.9673922102,-0.2440051316
H,0,2.8673601644,-3.1068931606,-0.2315086764
O,0,3.9867079528,-1.2957392071,-0.0146686639
H,0,4.5874256,-1.1504743399,0.9320221117
O,0,3.6349759693,1.3624817574,0.5201131411
H,0,3.3932948166,2.3015203642,0.5295534395
C,0,-1.2053689846,0.783722278,0.1648362651
O,0,-1.4259949342,1.9664251669,0.3186832612
O,0,-2.1573410663,-0.1326704244,-0.0031177539
C,0,-3.5163916156,0.3490111701,0.0014805095
H,0,-3.630547061,1.0787723141,-0.8071028188
H,0,-3.7039844253,0.857076745,0.9531293337
C,0,-4.4313060305,-0.8425886562,-0.1864142536
H,0,-4.2554507364,-1.5597412617,0.6228315963
H,0,-4.1797085767,-1.3428327732,-1.1280245923
C,0,-5.8936010027,-0.4039346924,-0.1954154699
H,0,-6.0894878015,0.3001706149,-1.0108874596
H,0,-6.5562848753,-1.262940295,-0.3282792426
H,0,-6.164711446,0.0865091653,0.7453539974
O,0,4.9596049502,-1.044048914,2.1148569484
O,0,3.9187756738,-0.402978315,2.7099943223
H,0,3.9826939503,0.5156492322,2.3792537333

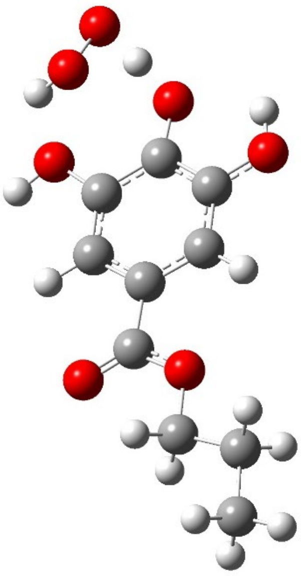

14(3)-TS

Charge = 0 Multiplicity = 2

C,0,0.4097284079,-0.9796366304,0.061966192
C,0,1.7454784519,-1.4431861938,0.0730996176
C,0,2.8037913271,-0.4946851276,0.0637759486
C,0,2.5135166344,0.8817139115,0.0477191003
C,0,1.2064106232,1.3176050744,0.026712803
C,0,0.1521854129,0.3776912247,0.0262321316
H,0,-0.3894237156,-1.7119926933,0.0674802873
H,0,0.9742091385,2.3785197438,0.0079767119
O,0,2.0247242727,-2.7271248583,0.0733870724
H,0,2.5853014999,-2.9758615429,1.0130352191
O,0,4.0598041973,-0.9410312325,0.0715143843
H,0,4.6723445514,-0.188686938,0.1174612109
O,0,3.6132969719,1.6848065325,0.0503096644
H,0,3.3633046398,2.6195001848,0.0376776032
C,0,-1.2377248581,0.9178666753,-0.0030882472
O,0,-1.4968893539,2.1038019252,-0.0074329393
O,0,-2.1651966727,-0.0407345258,-0.0234741933

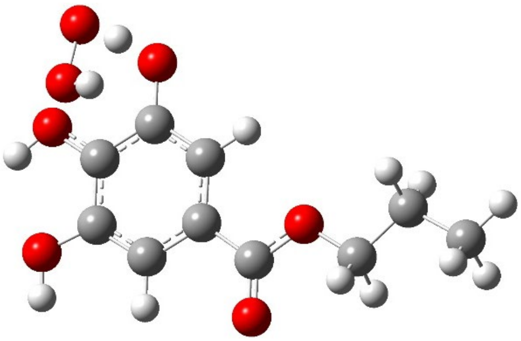

C,0,-3.5370364136,0.3987340563,-0.0563017093
H,0,-3.6849038784,1.0204110549,-0.9455859591
H,0,-3.7299052109,1.0137389874,0.8290986139
C,0,-4.4180429506,-0.8324208347,-0.0838345595
H,0,-4.2079419583,-1.4431067447,0.8011301511
H,0,-4.1648363527,-1.4337457821,-0.9638414094
C,0,-5.8933429681,-0.4409862394,-0.117515164
H,0,-6.1231620323,0.1570732877,-1.0055645973
H,0,-6.5308419309,-1.3286691454,-0.1375832169
H,0,-6.165997135,0.1478445144,0.7645980951
O,0,2.9335807647,-3.0204601405,2.2275007854
O,0,2.7400626808,-1.7282758393,2.6272868431
H,0,1.9178687567,-1.7593355243,3.15075212

15(1)-TS

Charge = 0 Multiplicity = 2

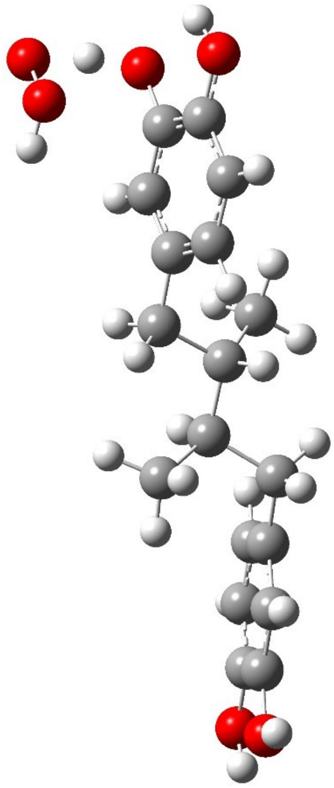

C,0,-4.0397023406,-0.9648922227,-0.3666538738
C,0,-5.3524214144,-0.652649739,-0.0338375727
C,0,-5.749045682,0.6807108813,0.1187655076
C,0,-4.8166456937,1.6932886485,-0.0697052137
C,0,-3.5003238364,1.3760070311,-0.405746991
C,0,-3.0924428444,0.0477317256,-0.5578994009
H,0,-3.7522199741,-2.0093270849,-0.4778662598
H,0,-5.135513069,2.7248090525,0.0447885655
H,0,-2.7813220086,2.177779374,-0.5561001505
O,0,-7.0350938966,0.9860254957,0.4438097452
H,0,-7.5391796944,0.1636719774,0.5301088813
O,0,-6.3389696728,-1.5794721847,0.1680062093
H,0,-5.9988605469,-2.4747777324,0.0369609101
C,0,-1.6573864023,-0.2805659992,-0.8972569823
H,0,-1.5864348967,-1.330380623,-1.2112130092
H,0,-1.3557202461,0.3269504673,-1.7560854741
C,0,-0.6741883574,-0.0596490854,0.2736661903
H,0,-0.8364424858,0.9561321152,0.6671563186
C,0,0.7870214616,-0.1299298575,-0.2295705713
H,0,0.8765804354,-1.0271062132,-0.8633035009
C,0,1.7907071958,-0.2809603131,0.9346123178
H,0,1.5688169037,-1.1967158679,1.4917543542
H,0,1.6603570182,0.5614660812,1.6267240624
C,0,3.2275005792,-0.3387507122,0.4789242294
C,0,3.7058323179,-1.4896440506,-0.19221993
C,0,4.0978328329,0.7225913712,0.6670165708
C,0,5.0052688453,-1.5817948337,-0.666186857
H,0,3.0307928304,-2.3297421188,-0.3378140969
C,0,5.4257692964,0.6574667959,0.199804239
H,0,3.7765190813,1.6323522445,1.1690342913
C,0,5.8776282902,-0.5084312601,-0.4737520783
H,0,5.3613613179,-2.4712800685,-1.1754956227
O,0,6.2934101831,1.6571288454,0.3447159118
H,0,6.708645809,1.6063488473,1.3474956663
O,0,7.1390032519,-0.5676846468,-0.9220523557
H,0,7.5737312075,0.2757477787,-0.7065816494
C,0,-0.9837259035,-1.0587843253,1.3906212154
H,0,-0.4310905823,-0.8402936758,2.3079632822
H,0,-2.0490055304,-1.0354007267,1.6402638719

H,0,-0.7349317258,-2.0814616206,1.0771041549
C,0,1.1457757075,1.1016355553,-1.0664025144
H,0,2.1327660687,0.9993930863,-1.5268249046
H,0,1.1649891204,1.9967561358,-0.431390086
H,0,0.4285569038,1.2795494317,-1.8715585502
O,0,7.0679876946,1.1596625142,2.5433073998
O,0,6.657802486,-0.1335049362,2.4823748427
H,0,5.7533047255,-0.1316766576,2.8511542875

15(2)-TS

Charge = 0 Multiplicity = 2
C,0,-4.0959404002,-0.973469098,-0.4236729619
C,0,-5.4156972049,-0.6645258028,-0.1167651372
C,0,-5.8193164573,0.6679647661,0.0254373331
C,0,-4.8865447366,1.6830337789,-0.1468555197
C,0,-3.562933978,1.3690497432,-0.4563464688
C,0,-3.1481718699,0.0417609946,-0.5979032597
H,0,-3.8035589156,-2.0173052299,-0.5274927196
H,0,-5.2108839902,2.7137416954,-0.0407479426
H,0,-2.8435107749,2.172702283,-0.5942145161
O,0,-7.1122829962,0.9699504236,0.3247979853
H,0,-7.6154780383,0.1462275931,0.4031761333
O,0,-6.4029662809,-1.5937829504,0.0684810095
H,0,-6.0582518281,-2.4883915954,-0.0552958667
C,0,-1.705372348,-0.2825868135,-0.9068795427
H,0,-1.6244776294,-1.3320508997,-1.2193566565
H,0,-1.3872136346,0.3262109381,-1.7587518926
C,0,-0.7492831425,-0.0589839049,0.285901843
H,0,-0.9217943995,0.9570623793,0.6744064093
C,0,0.7228659964,-0.128434287,-0.1832978929
H,0,0.8322243192,-1.0321887844,-0.8035364956
C,0,1.6969908233,-0.2616134709,1.0097014963
H,0,1.470024388,-1.1769817711,1.5652541762
H,0,1.5411067779,0.5842089073,1.6918095563
C,0,3.1414646564,-0.3154554599,0.5885781084
C,0,3.641235355,-1.463849168,-0.0640511556
C,0,3.9996610808,0.7610190782,0.8127851811
C,0,4.9538542009,-1.5275543701,-0.4823930981
H,0,2.9785298844,-2.3091216848,-0.2298674996
C,0,5.3226051195,0.712899794,0.3839229093
H,0,3.6362877706,1.650198834,1.3248550801
C,0,5.8285510473,-0.4337285076,-0.2909560426
H,0,5.3500640921,-2.4051874266,-0.9834780618
O,0,6.2044016679,1.7247206252,0.5977068316
H,0,5.7421076957,2.5037790513,0.9425375745
O,0,7.0543297792,-0.4549874449,-0.7707583087
H,0,7.807625715,-0.3186677423,0.0362083706
C,0,-1.08112969,-1.0579770608,1.3964963083
H,0,-0.5499429827,-0.8373640267,2.3259377991
H,0,-2.1517464386,-1.0376536699,1.6221484396
H,0,-0.8220589481,-2.0799508503,1.0892900861
C,0,1.0988939521,1.095096407,-1.0243518799
H,0,2.0990334435,0.9918671016,-1.4558188205
H,0,1.0944592173,1.9982221119,-0.4003157341
H,0,0.4043882137,1.258916346,-1.851881827

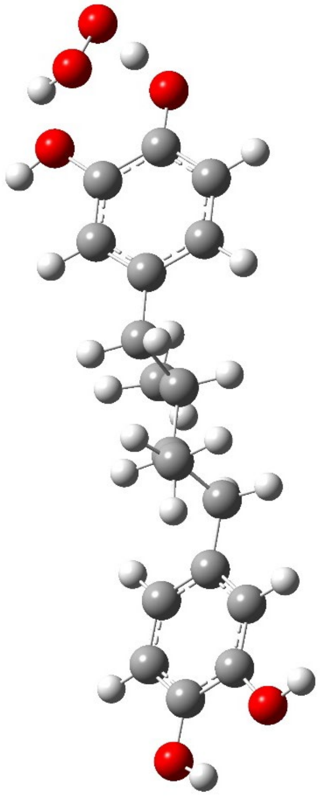

O,0,8.4720957113,-0.3526079552,1.1191638224
O,0,7.5022482644,-0.2934929795,2.077324798
H,0,7.2122061526,0.6387852926,2.0568140189

15(3)-TS

Charge = 0 Multiplicity = 2

C,0,-4.0784224728,-0.8167395595,-0.5473367563
C,0,-5.3930226359,-0.4710484372,-0.2476574767
C,0,-5.7639670097,0.8912463687,-0.0658255358
C,0,-4.76341019,1.8769095849,-0.2261511172
C,0,-3.4617765079,1.5215683714,-0.5108483796
C,0,-3.0976535329,0.1669579114,-0.6754607127
H,0,-3.8172809598,-1.8649200104,-0.6816578668
H,0,-5.0563851433,2.9145611701,-0.1005195717
H,0,-2.7012715998,2.2904919328,-0.6206523456
O,0,-6.9867694558,1.2206563064,0.2937356865
H,0,-7.7336885344,0.8871848534,-0.4601711332
O,0,-6.3904801138,-1.3875726775,-0.1363137657
H,0,-6.0233114066,-2.2845650779,-0.1477557084
C,0,-1.666201497,-0.1921596501,-0.9721074549
H,0,-1.6079703351,-1.2352409559,-1.3067485999
H,0,-1.3224350073,0.4330680323,-1.8022585445
C,0,-0.7254066804,-0.0168387483,0.2435168001
H,0,-0.8920007323,0.9875627137,0.6633168808
C,0,0.7507129019,-0.0871826626,-0.2137192608
H,0,0.8512819701,-0.9591304992,-0.8797320735
C,0,1.7159983396,-0.2984290884,0.97228574
H,0,1.4633617204,-1.230964306,1.4861324532
H,0,1.5717919768,0.5167646801,1.6945453115
C,0,3.1683421883,-0.3577942517,0.5597084514
C,0,3.6711339688,-1.4764746075,-0.1101459353
C,0,4.0333964067,0.714594476,0.8057329633
C,0,5.0002726823,-1.5289012635,-0.5317605442
H,0,3.0178873282,-2.3233990019,-0.3052736875
C,0,5.3581034579,0.6652568241,0.3889930998
H,0,3.6725873668,1.6002015915,1.3264507284
C,0,5.8501117463,-0.4579189309,-0.2858261883
H,0,5.3926292314,-2.3989155091,-1.049428572
O,0,6.268276539,1.6667370589,0.591332407
H,0,5.8605945246,2.4106654614,1.0548560245
O,0,7.1479951892,-0.505660578,-0.6931377889
H,0,7.5863413099,0.3191628698,-0.4374162393
C,0,-1.0873091919,-1.0484350644,1.3143310131
H,0,-0.5602078074,-0.8691029806,2.25456267
H,0,-2.1594990733,-1.0177108258,1.5330783098
H,0,-0.8424996378,-2.0632187699,0.9743302161
C,0,1.1470963745,1.1717470716,-0.9902892623
H,0,2.1481832058,1.074896609,-1.4198915619
H,0,1.1529517554,2.0415674981,-0.3204633765
H,0,0.4595734573,1.3888602605,-1.8120762075
O,0,-8.3620405981,0.5988816243,-1.526586912
O,0,-7.3847113771,0.0857066291,-2.3288392422
H,0,-7.2218101307,-0.802727653,-1.9574732941

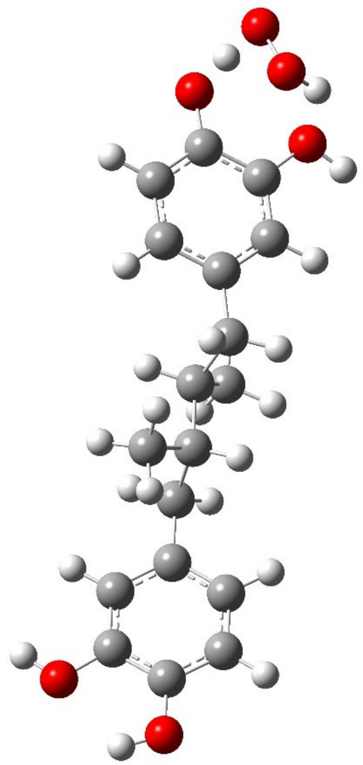

15(4)-TS

Charge = 0 Multiplicity = 2

C,0,-4.1055300512,-0.9869035222,-0.3944531806
C,0,-5.4358163264,-0.6528798758,-0.070837813
C,0,-5.8047801011,0.711864256,0.0633940324
C,0,-4.8493758003,1.7123925321,-0.1268964013
C,0,-3.5494404369,1.3528061789,-0.4468934289
C,0,-3.1527322754,0.0009660134,-0.584728162
H,0,-3.8492330439,-2.0403115979,-0.4820119739
H,0,-5.1424623767,2.752504322,-0.0288730644
H,0,-2.8084791448,2.1340783773,-0.6006827034
O,0,-7.0692070928,1.0286806791,0.374069369
H,0,-7.5681242448,0.2003749731,0.4823461645
O,0,-6.3815254693,-1.5677031396,0.1349284103
H,0,-6.7709910568,-1.8800816046,-0.8295213981
C,0,-1.7157034279,-0.3280559147,-0.902692375
H,0,-1.6403547913,-1.3802108679,-1.2052277213
H,0,-1.4045421085,0.2752576929,-1.7614297829
C,0,-0.7529395429,-0.0935370601,0.2836923451
H,0,-0.9342601432,0.9197566866,0.6763837375
C,0,0.7159704296,-0.1438572068,-0.1987888659
H,0,0.8278304253,-1.0408796404,-0.8288844811
C,0,1.7060405797,-0.2796791494,0.9779057737
H,0,1.4861841969,-1.1960673041,1.5339458476
H,0,1.5508234941,0.5607988281,1.6682942368
C,0,3.1533871925,-0.3189550826,0.5461559924
C,0,3.6773206901,-1.4537689877,-0.0789682132
C,0,3.993186499,0.7851206641,0.7323794565
C,0,5.0023995483,-1.4915396455,-0.5147192134
H,0,3.0443317614,-2.3253640281,-0.2262067528
C,0,5.3140830935,0.7501072842,0.3023401133
H,0,3.6156031654,1.6841666316,1.2169045174
C,0,5.827126314,-0.3895539981,-0.3276220035
H,0,5.4112471384,-2.3741537161,-0.9970792323
O,0,6.2008941096,1.7818467141,0.4494466771
H,0,5.7804868953,2.5340097994,0.8875832296
O,0,7.1212437367,-0.4228034563,-0.7484069534
H,0,7.5414283703,0.4227685172,-0.5333438382
C,0,-1.0659435696,-1.0993467514,1.3937798449
H,0,-0.5323501285,-0.8718919369,2.319927069
H,0,-2.1354721901,-1.0975158889,1.6263917315
H,0,-0.792912986,-2.1164330073,1.0835604044
C,0,1.0673722206,1.0899450729,-1.03512051
H,0,2.0674301093,1.0034120227,-1.4694252677
H,0,1.0527818398,1.9893144875,-0.4055161101
H,0,0.3673835353,1.2488675419,-1.8596034772
O,0,-7.0635880458,-1.9087367662,-2.1241151356
O,0,-6.5330376723,-0.7314804737,-2.5437547334
H,0,-5.6275456483,-0.9501995228,-2.8376688004

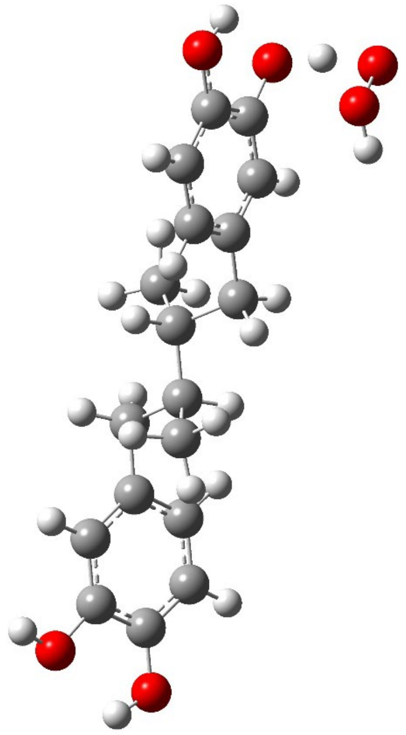

16(1)-TS

Charge = 0 Multiplicity = 2

C,0,-0.2815674243,-0.8341235907,-0.0168598266
C,0,-0.443289729,0.560946012,0.0351925125
C,0,-1.8062787074,1.1980363602,0.017953353

H,0,-2.0299168606,1.6001399342,1.0153481982
H,0,-1.7966228021,2.0559474628,-0.6615232651
C,0,-2.8678152056,0.1792184396,-0.3916346817
H,0,-2.832369093,0.0036812943,-1.4728002134
H,0,-3.8692414248,0.5421267514,-0.144408699
O,0,-1.3415878957,-1.6819655354,-0.0540586742
C,0,0.9827862265,-1.4148250472,-0.0499716621
H,0,1.090622179,-2.4952808313,-0.0658418761
C,0,2.1295430778,-0.589467757,-0.0245053855
C,0,0.7215592147,1.3605812161,0.082214624
C,0,-2.6087108342,-1.1304234798,0.3269244236
H,0,-3.3412891085,-1.8969238831,0.0707174554
H,0,-2.6122330887,-0.9843711509,1.4158878433
C,0,1.9905643252,0.8160322716,0.0375187796
H,0,2.8820425361,1.4354843318,0.054312921
O,0,0.5046024448,2.6991940517,0.1568382803
H,0,1.3488883103,3.1717984881,0.1577912708
O,0,3.3347287459,-1.1269497384,-0.0258426701
H,0,3.5322692074,-1.5858305033,-1.0328744388
O,0,3.5127896442,-1.9215732065,-2.2443352879
O,0,2.4153049853,-1.2667103543,-2.7218075822
H,0,1.6780436267,-1.8950945554,-2.6055169091

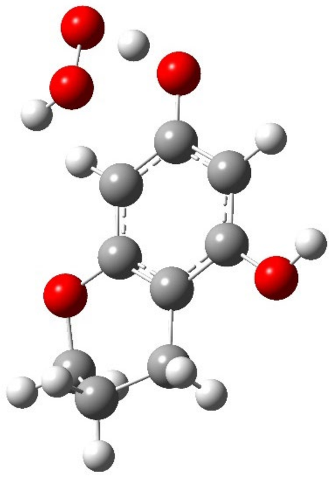

16(2)-TS

Charge = 0 Multiplicity = 2

C,0,-0.1896644992,-0.8253146935,0.0005672132
C,0,-0.3007518347,0.5720869304,0.0328331611
C,0,-1.6366490412,1.2566462435,-0.0419186285
H,0,-1.9068222751,1.6385663344,0.951724993
H,0,-1.5677584644,2.1273507383,-0.7009779334
C,0,-2.6985255436,0.2763989537,-0.5358884255
H,0,-2.5835116331,0.1020802058,-1.6119521857
H,0,-3.7034129657,0.6726445806,-0.3655288053
O,0,-1.2710257759,-1.6426108496,-0.0792430982
C,0,1.0541177777,-1.4535941638,0.026484091
H,0,1.1148937977,-2.5364665843,0.0002206829
C,0,2.2274153301,-0.6858069602,0.0980035952
C,0,0.9050123604,1.3249184534,0.11594966
C,0,-2.5427617058,-1.0417763363,0.1978858
H,0,-3.2805510418,-1.7813490977,-0.1162860692
H,0,-2.6285124511,-0.8935126083,1.2831981886
C,0,2.169028381,0.6961532768,0.1366315222
H,0,3.0624005402,1.3102964468,0.1984446608
O,0,0.8373864074,2.6432183078,0.1852410863
H,0,0.5620818781,3.0499168674,-0.8120965485
O,0,3.3955427674,-1.3755901106,0.1284933847
H,0,4.1413542557,-0.7629368534,0.1959155207
O,0,0.3705926008,3.2130948519,-2.0677615343
O,0,0.2417302196,1.924791023,-2.5021526049
H,0,1.1336802155,1.6765710541,-2.8096689765

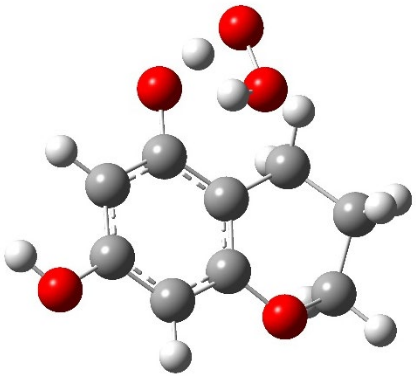

A-17(1)-TS
Charge = 0 Multiplicity = 2
C,0,-0.110932,2.087512,-0.093372
C,0,-1.264095,1.314397,0.044111
C,0,-1.140303,-0.064347,0.145301
C,0,0.131171,-0.700461,0.100329
C,0,1.285054,0.126799,-0.010773
C,0,1.157819,1.507139,-0.11679
H,0,-0.201835,3.165846,-0.174166
H,0,-2.245695,1.777545,0.079191
H,0,2.045883,2.124869,-0.214172
O,0,-2.201331,-0.89559,0.32708
H,0,-3.030112,-0.405501,0.217773
O,0,2.468577,-0.5136,-0.028986
H,0,3.188697,0.127911,-0.112856
O,0,0.248762,-2.008304,0.102277
H,0,-0.121776,-2.446708,1.052747
O,0,-0.44774,-2.676819,2.260732
O,0,-0.792107,-1.441628,2.720467
H,0,-1.660149,-1.270171,2.305735

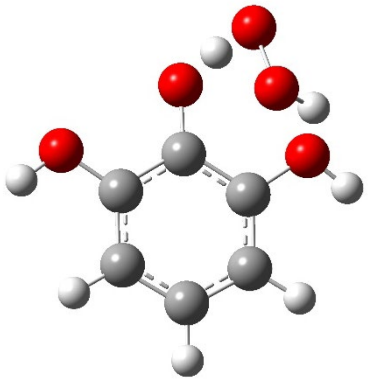

B-17(1)-TS
Charge = 0 Multiplicity = 2
C,0,-0.1347015559,2.0634330768,-0.0357574256
C,0,-1.3079955094,1.3055236679,-0.0134765141
C,0,-1.2259238705,-0.0817179382,0.0262790888
C,0,0.038757635,-0.7305301157,0.0156503611
C,0,1.2110524518,0.0756251289,-0.0044483329
C,0,1.1232466589,1.4643431917,-0.0264930133
H,0,-0.2060913536,3.14620241,-0.0597935325
H,0,-2.2797382244,1.7905013509,-0.0233605342
H,0,2.0275221946,2.0657390481,-0.0368373681
O,0,-2.3056993248,-0.8893989225,0.0698317061
H,0,-3.1135202319,-0.356563329,0.0544581295
O,0,2.3732693501,-0.6008371368,0.0043636519
H,0,3.11743447,0.0163674824,0.0514253186
O,0,0.1206089184,-2.0454591192,-0.0045305335
H,0,0.5669246834,-2.3885556489,0.9244933778
O,0,0.8561307009,-2.4878799386,2.211424285
O,0,0.8057636273,-1.1824002835,2.5944834239
H,0,-0.05933996,-1.0907215243,3.0358345517

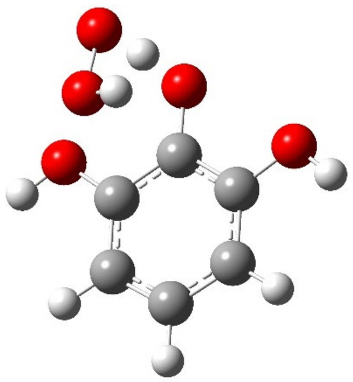

17(2)-TS
Charge = 0 Multiplicity = 2
C,0,-0.1129392246,2.172548253,-0.1942194357
C,0,-1.2894727701,1.4611472712,-0.0987516367
C,0,-1.2609264838,0.0507598055,0.0406471573
C,0,0.0006990049,-0.5970165299,0.1169474763
C,0,1.1840278733,0.1467156253,0.0325201662
C,0,1.1344824586,1.5208775413,-0.128600602
H,0,-0.1367887444,3.249892637,-0.3181819539
H,0,-2.2588071006,1.9450746319,-0.1473997595
H,0,2.0577374025,2.0893105074,-0.1951564357
O,0,-2.3680963889,-0.6530630215,0.0471915516
H,0,-2.4618731492,-1.2704117701,0.9981719547

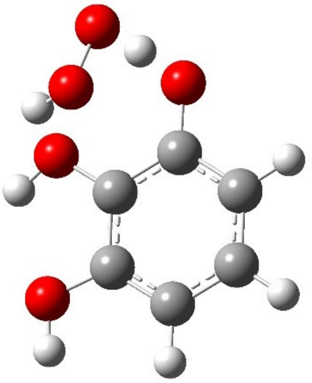

O,0,2.3240199894,-0.596900625,0.1191135146
H,0,3.1102877226,-0.044401352,0.0075506066
O,0,0.0366906745,-1.9387435817,0.2921595223
H,0,0.9556853164,-2.2448787982,0.214380001
O,0,-2.4598276563,-1.6888555351,2.1595411102
O,0,-1.2853855425,-1.2144954235,2.6702671088
H,0,-0.6057045219,-1.7824166657,2.2581237538

18(1)-TS

Charge = 0 Multiplicity = 2

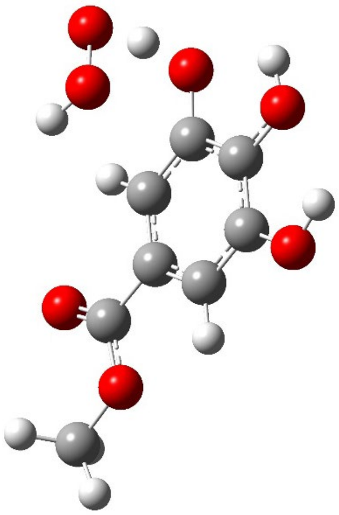

C,0,0.200764354,-1.3232749014,-0.0077593867
C,0,1.5985983958,-1.1417417367,0.005207409
C,0,2.1303053256,0.1723406621,0.0054377188
C,0,1.2830644155,1.2869211415,0.001751857
C,0,-0.0882271775,1.0940153607,-0.0095044561
C,0,-0.6238674219,-0.2109345126,-0.0164017627
H,0,-0.2103638681,-2.3276304332,-0.0020308243
H,0,-0.7416508721,1.9590229256,-0.0128213248
O,0,1.793645447,2.5411103409,0.0029714139
H,0,2.7618556622,2.5014116622,0.0061006141
O,0,3.4531472095,0.3827180005,0.0123534856
H,0,3.9066213517,-0.478485105,0.0439706112
O,0,2.4649538399,-2.1406170545,0.0365948816
H,0,2.5930170351,-2.5389819202,-1.0081983702
C,0,-2.098842106,-0.437026378,-0.0295320065
O,0,-2.6149407891,-1.5333001853,-0.0715479853
O,0,-2.7935806799,0.7031382142,0.0076076712
C,0,-4.2206620125,0.5636862409,-0.0032119891
H,0,-4.5513730713,-0.0017807643,0.8703223894
H,0,-4.6141127757,1.5782126985,0.0278148001
H,0,-4.5438352164,0.0554556431,-0.9139720626
O,0,2.5766882489,-2.6716019491,-2.2593049944
O,0,1.9653251783,-1.5248030615,-2.6610316838
H,0,1.0234782472,-1.7639310883,-2.7624311856

18(2)-TS

Charge = 0 Multiplicity = 2

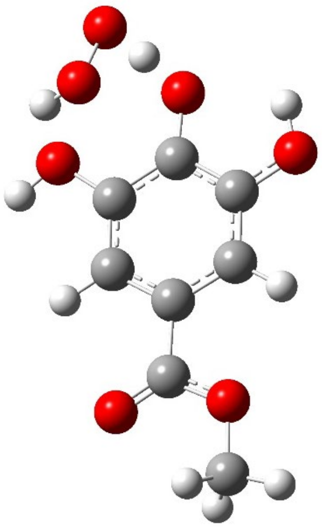

C,0,0.1597309054,-1.3243139987,0.0100521539
C,0,1.5338055473,-1.1644892728,-0.0057172161
C,0,2.1095567466,0.1334449336,-0.0205936723
C,0,1.2450805559,1.2628075688,-0.0555113798
C,0,-0.1328000661,1.105730063,-0.0344318133
C,0,-0.6588188712,-0.1878388393,0.0034434279
H,0,-0.2892548451,-2.3128456061,0.0226157304
H,0,-0.7770413095,1.9762962093,-0.0465479008
O,0,1.793515596,2.4856374452,-0.0814094968
H,0,2.761360283,2.3854052869,-0.0820666303
O,0,3.4045780488,0.3477235908,0.0502604587
H,0,3.9487237271,-0.1035773583,-0.8331660315
O,0,2.4156301411,-2.1974846819,-0.0437834584
H,0,1.9540391613,-3.0387624812,0.0960604455
C,0,-2.1366769756,-0.4177099553,0.0286329451
O,0,-2.6451558596,-1.5176340028,0.0670579907
O,0,-2.834117877,0.7183826206,0.0052351457
C,0,-4.2610375137,0.5745955759,0.0289449563

H,0,-4.5730943106,0.0619985168,0.9411167017
H,0,-4.6573394768,1.5881185145,0.0056573415
H,0,-4.5978856018,0.011518512,-0.8437529521
O,0,4.2806154356,-0.4999122128,-1.9637117715
O,0,3.1130926233,-0.9610267088,-2.4864262327
H,0,2.9488335256,-1.7963598496,-2.0042677717

18(3)-TS
Charge = 0 Multiplicity = 2
C,0,0.1022066689,-1.2815852616,0.0104376396
C,0,1.4659775752,-1.0780064066,-0.049410629
C,0,1.984878528,0.2246592138,-0.0934995613
C,0,1.1138653275,1.3461697343,-0.0739006387
C,0,-0.282590209,1.118638606,-0.0371434869
C,0,-0.769976866,-0.1725299502,0.0185573068
H,0,-0.3101731702,-2.2858627408,0.0437421575
H,0,-0.942169154,1.9784509825,-0.0320527219
O,0,1.6028021116,2.5620361805,-0.0414665425
H,0,2.2197894684,2.7801364722,-0.9807852582
O,0,3.3124856757,0.4370512256,-0.1756758974
H,0,3.780112647,-0.4105244376,-0.0844924553
O,0,2.4109584073,-2.0582259293,-0.0702322541
H,0,2.0078656257,-2.9353948309,-0.0002609179
C,0,-2.232784115,-0.4602925941,0.0825340321
O,0,-2.696629364,-1.5803513995,0.124059905
O,0,-2.9761670471,0.6470111884,0.0904757213
C,0,-4.3944822815,0.4453164187,0.1534387881
H,0,-4.6602682365,-0.0830375975,1.0713926053
H,0,-4.8319781826,1.442101204,0.1467268127
H,0,-4.7337430405,-0.1271479085,-0.7121886236
O,0,2.6691989763,2.8424324891,-2.1187332112
O,0,2.4264637403,1.5995367527,-2.6286020315
H,0,3.1317490645,1.0452120388,-2.2424987188

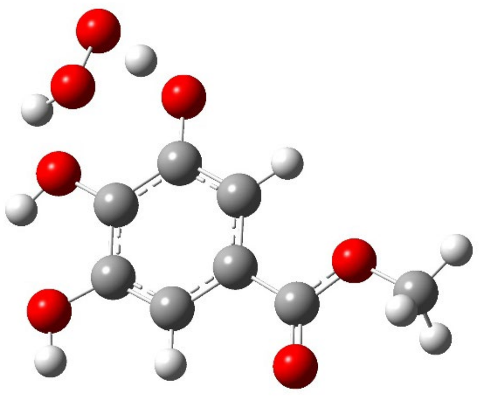

19(1)-TS
Charge = 0 Multiplicity = 2
C,0,-4.9270615502,-0.1484839045,0.0439007905
C,0,-4.0526885379,-1.2663452697,0.0414940416
C,0,-2.6863285309,-1.0834595357,0.0304969547
C,0,-2.1268192997,0.214081854,0.0195716208
C,0,-3.0056305449,1.3211018491,0.0250626504
C,0,-4.3755475452,1.1534588275,0.0484627016
H,0,-4.4834437139,-2.2630992349,0.0455786289
H,0,-2.0415894146,-1.9562596965,0.0274902335
H,0,-2.5860615096,2.3236671879,0.0147300865
H,0,-5.051443908,2.0028836263,0.0531211811
O,0,-6.237872385,-0.3031605172,-0.0015735864
H,0,-6.5860345225,-0.8891834436,0.8525680486
C,0,-0.69159863,0.4707145778,0.0050510571
H,0,-0.4173732783,1.5236049128,-0.0145700587
C,0,0.2789931253,-0.4651895983,0.015026763
H,0,0.0072458018,-1.5184392175,0.0357114522
C,0,1.7247287842,-0.2109003915,-0.000157865
C,0,2.2674875086,1.0829317712,-0.0201921293
C,0,2.5777343707,-1.3220617923,0.006321041

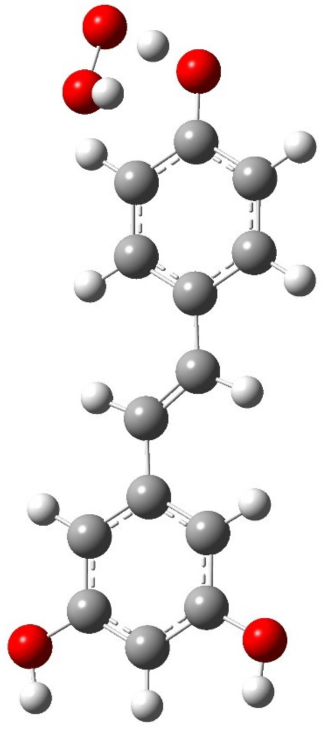

C,0,3.6481281217,1.2448697953,-0.0328536157
H,0,1.6429317564,1.9690164094,-0.0257259659
C,0,3.9591252618,-1.1383307976,-0.0072872334
H,0,2.1774584085,-2.3308400076,0.0214281964
C,0,4.5082498596,0.1425107975,-0.0259971637
H,0,5.5865445844,0.2838890186,-0.0346546922
O,0,4.1272024288,2.5180485627,-0.0516026249
H,0,5.0938982096,2.5131975611,-0.0594284013
O,0,4.7360170325,-2.2540198947,-0.0009881581
H,0,5.6719081383,-2.011413154,-0.0128479798
O,0,-6.7922312201,-1.3386729619,2.0590876293
O,0,-5.5339563274,-1.3210358238,2.5883035716
H,0,-5.5325687843,-0.5504294102,3.1856300356

19(2)-TS

Charge = 0 Multiplicity = 2
C,0,-4.9530995121,-0.1315245285,0.0112455163
C,0,-4.1075874508,-1.2382968779,0.1446704946
C,0,-2.7326856058,-1.0630823072,0.1321244168
C,0,-2.1606656379,0.213251419,-0.0153788749
C,0,-3.028948689,1.3054433954,-0.1463032516
C,0,-4.4115006399,1.1453550978,-0.1330357411
H,0,-4.5448375452,-2.22510243,0.2586811132
H,0,-2.0982027596,-1.9375924269,0.2394477788
H,0,-2.6138665366,2.3036879402,-0.2607050943
H,0,-5.0652678006,2.0078436154,-0.2357935075
O,0,-6.2922717421,-0.3627618119,0.0312570274
H,0,-6.7753292007,0.4693710088,-0.067169093
C,0,-0.7129308392,0.4555184414,-0.0364178777
H,0,-0.4365053895,1.504601973,-0.134337688
C,0,0.2563460834,-0.471523235,0.0408474771
H,0,-0.006263959,-1.5250910848,0.1141830339
C,0,1.7026305315,-0.1999322158,0.0200017265
C,0,2.244265636,1.0899229313,0.0734416564
C,0,2.5812720215,-1.2879930863,-0.0541045384
C,0,3.6461632287,1.2801605556,0.0452842387
H,0,1.6182642578,1.9757893625,0.1263794275
C,0,3.9752081238,-1.1003379541,-0.083971826
H,0,2.1959207919,-2.3026737202,-0.0951333394
C,0,4.5191607656,0.1691323939,-0.027356529
H,0,5.5918311937,0.339127576,-0.0386160577
O,0,4.1427259073,2.5011350127,0.0795202931
H,0,4.0067973242,2.9245101633,1.1259005469
O,0,4.7300066613,-2.2242718638,-0.1615237558
H,0,5.670392945,-1.9960003195,-0.1657353195
O,0,3.7719377592,3.1018813034,2.3309247922
O,0,3.3475073589,1.8710225736,2.7386017901
H,0,2.374915628,1.9041456386,2.6677979341

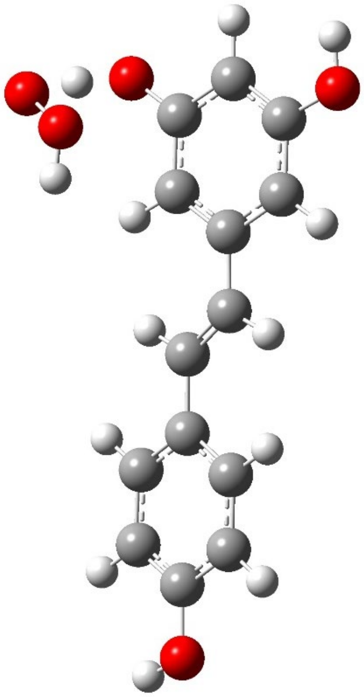

19(3)-TS

Charge = 0 Multiplicity = 2
C,0,-4.8277498973,-0.1962986863,0.11492257
C,0,-3.953186593,-1.287209899,0.0657561865
C,0,-2.5844396643,-1.0741642835,0.0202778351
C,0,-2.0470935612,0.2256391165,0.0243179131

C,0,-2.9441072625,1.3012347186,0.0720436009
C,0,-4.3213348903,1.1030278568,0.1165374422
H,0,-4.3633549747,-2.2920588268,0.0621037055
H,0,-1.9266507219,-1.937026005,-0.0202204392
H,0,-2.5562052237,2.3168215526,0.0739347111
H,0,-4.9980846504,1.9534615495,0.1525609163
O,0,-6.159513794,-0.4659068135,0.1570719136
H,0,-6.6648645081,0.3580486639,0.1904130771
C,0,-0.6063389518,0.5056605493,-0.0199449705
H,0,-0.3553913322,1.5653237672,-0.0457141532
C,0,0.382835538,-0.4030995779,-0.0237842227
H,0,0.1420840389,-1.4634206896,0.0212100594
C,0,1.8237270579,-0.108891579,-0.0709914146
C,0,2.3373552419,1.1820294915,-0.245464021
C,0,2.7236905438,-1.1730506466,0.0597368281
C,0,3.7252427559,1.4062524213,-0.2741980659
H,0,1.6858478676,2.0403323324,-0.3737598116
C,0,4.121725058,-0.9479978166,0.02815462
H,0,2.3670797624,-2.1913001363,0.1782457294
C,0,4.6234382435,0.362879348,-0.1282294182
H,0,5.6977233193,0.5213765606,-0.1488298988
O,0,4.1198959368,2.6926761097,-0.4483573173
H,0,5.0858001879,2.7462969614,-0.4691634738
O,0,4.9605682585,-1.959768202,0.1312993104
H,0,4.8825195832,-2.4346200319,1.154867389
O,0,4.7474044965,-2.6492226904,2.3735833364
O,0,3.5983186388,-1.9667609671,2.6598857368
H,0,3.9024267864,-1.1529988077,3.103406736

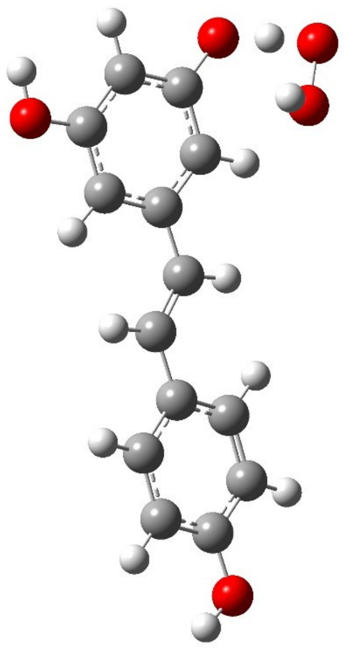

A-20(1)-TS
Charge = 0 Multiplicity = 2
C,0,-4.4571097093,-0.2071217531,-0.1538859751
C,0,-3.597003484,-1.3051453869,-0.0993573446
C,0,-2.2274537002,-1.1099957182,-0.0466637252
C,0,-1.6661118491,0.1918322844,-0.0622477618
C,0,-2.5290112267,1.2779263533,-0.1265959225
C,0,-3.9298741716,1.1162961869,-0.1546450598
H,0,-4.0084736514,-2.311143196,-0.094835321
H,0,-1.5834312196,-1.9816154678,0.0028997619
H,0,-2.1452912377,2.2941589762,-0.1366280037
O,0,-4.7301163986,2.1588096342,-0.1271047035
H,0,-5.390417401,2.1876652025,-1.0326839191
O,0,-5.8050129746,-0.3251277225,-0.2244882246
H,0,-6.0698691284,-1.2486002204,-0.0931941448
C,0,-0.2192600684,0.4374253138,-0.0106311848
H,0,0.0577975588,1.4899016421,0.0076829581
C,0,3.0576708894,-1.3144537671,-0.1786893522
C,0,4.4368383142,-1.1139957331,-0.1535118565
C,0,4.9716446246,0.1460165727,0.110372857
C,0,4.0970542614,1.2082773262,0.3565723942
C,0,2.7181938001,1.0285712168,0.3357502282
C,0,2.1911909663,-0.2403889716,0.0577015172
H,0,2.6705879568,-2.3062725627,-0.3898141418
H,0,6.0485712621,0.2996529375,0.1298347215
H,0,2.0817278836,1.8794307539,0.5509167645

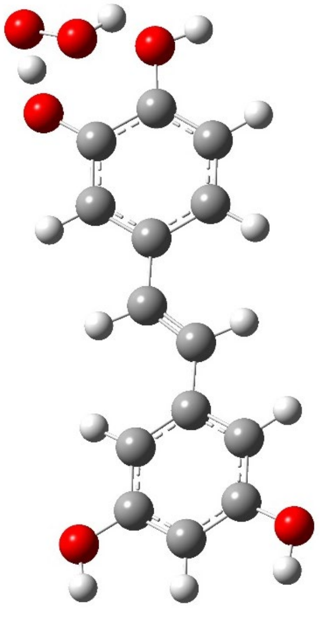

C,0,0.7432723722,-0.4993952148,0.0021418776
H,0,0.4739195656,-1.5526460311,-0.048831887
O,0,4.5572432254,2.4584659076,0.6328996023
H,0,5.5239113333,2.468445961,0.629588457
O,0,5.2277123582,-2.1936704109,-0.3970395967
H,0,6.1601900918,-1.9420077699,-0.3529661515
O,0,-5.878301231,2.1162039671,-2.1908737521
O,0,-5.3214242322,0.9735488188,-2.6905147151
H,0,-5.81974373,0.2593087712,-2.2493850063

B-20(1)-TS

Charge = 0 Multiplicity = 2

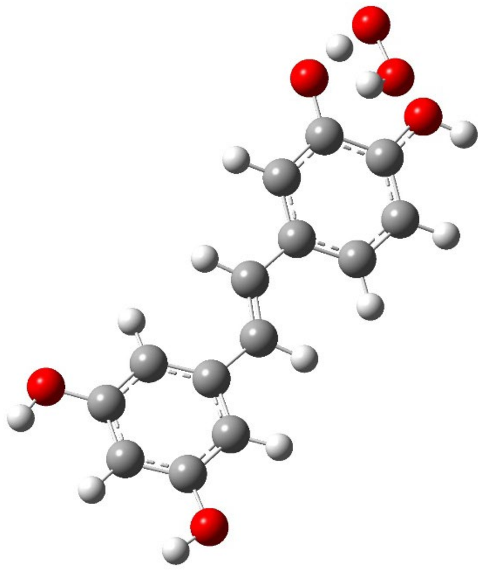

C,0,-4.529862438,-0.2522910312,-0.0025505058
C,0,-3.6559181478,-1.3433172988,0.0625544087
C,0,-2.2885993122,-1.1361282821,0.088724281
C,0,-1.7360440825,0.1692485005,0.041260192
C,0,-2.6086486829,1.2484174896,-0.0380745881
C,0,-4.0052025439,1.0715549792,-0.0568291475
H,0,-4.0596098186,-2.3515641526,0.1038818559
H,0,-1.6362192417,-2.0006766071,0.1558615785
H,0,-2.232117804,2.2667731393,-0.0802797248
O,0,-4.8250657518,2.1033500838,-0.1056914929
H,0,-5.4237018627,2.0353083862,-1.0204978098
O,0,-5.8647362904,-0.3651650814,-0.0088174114
H,0,-6.1267773111,-1.2969546147,-0.0442508449
C,0,-0.290974219,0.4254468063,0.0651702475
H,0,-0.0209047856,1.479139352,0.1114828628
C,0,2.9940787269,-1.2924911553,-0.2504678431
C,0,4.3719163745,-1.0820697948,-0.2563669069
C,0,4.9049356162,0.1739949963,0.0291334727
C,0,4.0298218768,1.2214107872,0.3310085876
C,0,2.6521937943,1.0315238736,0.3422592394
C,0,2.1264458397,-0.2328908048,0.0410663302
H,0,2.6084688349,-2.2810002125,-0.4792591643
H,0,5.9809220211,0.3351735362,0.0232062895
H,0,2.0157966476,1.8704972644,0.6002676891
C,0,0.6797630602,-0.5022024412,0.0165104182
H,0,0.4168457474,-1.5548827824,-0.0684129546
O,0,4.4887238973,2.4666294315,0.6316800184
H,0,5.4549849651,2.4829151252,0.606159234
O,0,5.1635071098,-2.148047955,-0.5524491043
H,0,6.0950891826,-1.8908521327,-0.5243473923
O,0,-5.755126128,1.85507931,-2.276608652
O,0,-5.0045266821,0.7603702254,-2.5996721693
H,0,-4.2535307922,1.1220054496,-3.1055329235

20(2)-TS

Charge = 0 Multiplicity = 2

C,0,-4.578045451,-0.7342847013,-0.0800023284
C,0,-3.6262665459,-1.7732947531,-0.096365543
C,0,-2.2783408565,-1.4846715306,-0.0522217279
C,0,-1.8282386641,-0.1407275918,-0.0023958271
C,0,-2.7711832228,0.8950952179,0.0126931721
C,0,-4.1308283053,0.6108624003,-0.0226843368
H,0,-3.9836968464,-2.7980759973,-0.1440035224

H,0,-1.5633944377,-2.299918588,-0.0563616485
H,0,-2.4514552988,1.9320449299,0.054828679
O,0,-5.0326182941,1.6051207799,-0.007334823
H,0,-5.9181931218,1.2069857619,-0.062861985
O,0,-5.8920472502,-0.9496963369,-0.1423873728
H,0,-6.2440815599,-1.230859703,0.831134013
C,0,-0.409860464,0.2192720864,0.0367780594
H,0,-0.2133919247,1.2833405923,0.1529963583
C,0,2.9802046513,-1.3032727978,-0.2356561852
C,0,4.3442779915,-1.0185295535,-0.2066793651
C,0,4.7982834791,0.2778590159,0.0294307928
C,0,3.8587010021,1.29162713,0.2410357591
C,0,2.4937756719,1.0274315686,0.2180314342
C,0,2.0480062017,-0.2798116047,-0.025454871
H,0,2.655994469,-2.3219188363,-0.4233581771
H,0,5.8638734429,0.4962895985,0.0516954175
H,0,1.8056923424,1.8464149269,0.3947084584
C,0,0.6228740757,-0.6371785763,-0.0689139125
H,0,0.4294743579,-1.6989551402,-0.2088953945
O,0,4.2400367202,2.57520377,0.4809074984
H,0,5.2042879713,2.644899786,0.479517585
O,0,5.2006598272,-2.0534555675,-0.4176499738
H,0,6.1160708705,-1.7446003477,-0.3784989424
O,0,-6.3142120231,-1.4124672694,2.1708033864
O,0,-5.1648668828,-0.8198240912,2.5744737924
H,0,-4.5044223954,-1.5387602779,2.6142889406

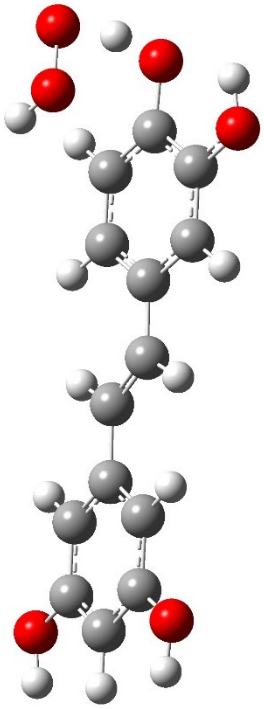

20(3)-TS

Charge = 0 Multiplicity = 2

C,0,-4.4303848492,-0.4181961007,0.0710750109
C,0,-3.588717907,-1.4783213679,-0.2468769905
C,0,-2.2090367364,-1.2946035624,-0.2623133574
C,0,-1.6517094548,-0.0456274955,0.0466442063
C,0,-2.5149622966,1.0144949406,0.3662306806
C,0,-3.8906912506,0.8367113137,0.3788209505
H,0,-4.0171440043,-2.4481512737,-0.4869925817
H,0,-1.5742917728,-2.1358119744,-0.5197385236
H,0,-2.1165772665,1.9959439901,0.6084187745
O,0,-4.7050323429,1.8786106602,0.6899942946
H,0,-5.626224441,1.5821700694,0.648316259
O,0,-5.7934297298,-0.4924792213,0.1091124618
H,0,-6.0968136286,-1.3810970549,-0.12161946
C,0,-0.2049889188,0.215778624,0.0504636396
H,0,0.0676163002,1.2457643712,0.2781582205
C,0,3.1166717771,-1.4373094174,-0.0972661913
C,0,4.5063345274,-1.2026605329,-0.0658080392
C,0,5.0094977713,0.0835142674,-0.1001684278
C,0,4.1042329676,1.1650440443,-0.2085692696
C,0,2.7091975735,0.9296076943,-0.2537533616
C,0,2.2091474044,-0.3790197912,-0.1742155237
H,0,2.7646201382,-2.4639832295,-0.0561390096
H,0,6.0757649173,0.286572143,-0.0651245113
H,0,2.0542169315,1.7833889828,-0.3849608639
C,0,0.7689455704,-0.6795560126,-0.1809781863
H,0,0.5190203424,-1.7218386935,-0.3700367413

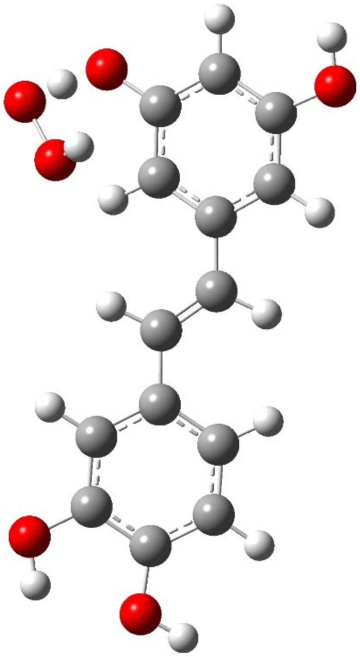

O,0,4.5687359051,2.3957466706,-0.2825980814
H,0,4.2046405611,3.0101666018,0.5955890583
O,0,5.2961662684,-2.3020696997,0.0104448059
H,0,6.228747131,-2.0438106609,0.021970438
O,0,3.8502696998,3.410012611,1.7198236269
O,0,2.901140432,2.4891278749,2.0685716588
H,0,3.3471045405,1.9144757192,2.718417414

20(4)-TS

Charge = 0 Multiplicity = 2
C,0,-4.5894784906,-0.5025804397,-0.0512613941
C,0,-3.7029677804,-1.5576399856,-0.2367332247
C,0,-2.3303002886,-1.3258226815,-0.2302595489
C,0,-1.8259151355,-0.0327599122,-0.0308001312
C,0,-2.7342505018,1.0222058641,0.1512410784
C,0,-4.1026327214,0.7961310571,0.1424850968
H,0,-4.0908375207,-2.5615047787,-0.3915780713
H,0,-1.6593041798,-2.1632076609,-0.3898484343
H,0,-2.3773657086,2.0370492706,0.3035076537
O,0,-4.9611762174,1.8333780076,0.3217537986
H,0,-5.8700720791,1.5002227813,0.286452448
O,0,-5.9495340119,-0.6216081404,-0.0459370448
H,0,-6.2162367383,-1.539522739,-0.1920518029
C,0,-0.3897987356,0.2796178711,-0.0047555926
H,0,-0.1607481663,1.3427598845,0.054296505
C,0,2.9722227459,-1.3246304121,0.1093114
C,0,4.3641387492,-1.0623170837,0.1349911393
C,0,4.8351713495,0.2654586296,0.0357110641
C,0,3.9155015137,1.291938332,-0.1102496879
C,0,2.5374561005,1.0300891959,-0.1368083338
C,0,2.0519002323,-0.2825427566,-0.0190309655
H,0,2.6397269979,-2.3575069605,0.1765905826
H,0,5.9049476955,0.450474239,0.0645798821
H,0,1.8656958423,1.8730673468,-0.2616608268
C,0,0.6172350472,-0.6082844876,-0.035333844
H,0,0.3972793051,-1.6739934351,-0.063580674
O,0,4.2837044406,2.5923147183,-0.231453024
H,0,5.2474153442,2.6719768553,-0.1982800719
O,0,5.2227800758,-2.0578658608,0.2349282419
H,0,5.194919367,-2.4625961655,1.2957995487
O,0,4.9905127974,-2.6517394315,2.5053010961
O,0,4.2396166522,-1.5682165244,2.8559119756
H,0,3.3142696899,-1.8655115675,2.7691838817

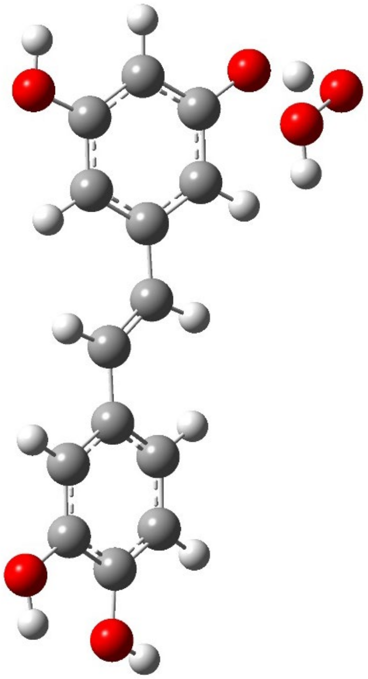

Cartesian coordinates of the optimized transition states between a phenol and •$OOCH_3$ studied at the M06-2X(SMD)/6-31++G(d,p) level of theory in water.

1-TS

Charge = 0 Multiplicity = 2
O,0,-0.2274949204,-1.615363521,3.7487799395
O,0,-1.4292901398,-2.1585826534,3.4335222237
C,0,-2.4956171512,-1.3438894338,3.9363843606
H,0,-3.4014363543,-1.7205395967,3.4588330347
H,0,-2.5502883309,-1.4681074566,5.0211532947

H,0,-2.3190176791,-0.2990919785,3.6761299596
C,0,-3.187170278,-0.5278784503,0.3571230392
C,0,-3.4428726253,-1.7702429134,-0.2512537719
C,0,-2.4498565667,-2.7595105206,-0.3051783131
C,0,-1.1871374562,-2.5603024306,0.2534629045
C,0,-0.945528116,-1.3078759631,0.896385647
C,0,-1.9500316603,-0.3110971595,0.9142611748
H,0,-3.9659094438,0.227400221,0.3770916791
H,0,-2.6730935371,-3.6958127359,-0.7969692993
H,0,-1.7155378157,0.6309391736,1.4011482436
O,0,0.234744388,-1.0104180233,1.462602199
H,0,0.1741628332,-1.2237716461,2.5154605847
O,0,-4.6807151482,-1.9235203869,-0.7727763193
C,0,-5.0073259109,-3.1629681271,-1.4029707797
H,0,-6.0400246585,-3.0612941628,-1.7322711391
H,0,-4.358260798,-3.3395909172,-2.2653555446
H,0,-4.9235310177,-3.9898362185,-0.6919546992
C,0,-0.0967129969,-3.6294639098,0.1358384121
C,0,-0.5902949556,-4.8666133645,-0.6249414296
H,0,-1.4301169171,-5.3515678429,-0.1162476302
H,0,-0.8939479647,-4.624266463,-1.6487198048
H,0,0.2288655355,-5.589802678,-0.6817994258
C,0,1.1004064105,-3.0528597936,-0.6437968351
H,0,1.5490772869,-2.2013312705,-0.1294839117
H,0,1.8637997778,-3.8302114508,-0.7582168576
H,0,0.7888710193,-2.732542294,-1.6439577886
C,0,0.3596744284,-4.0975771879,1.5291778955
H,0,1.1078148647,-4.8889703293,1.4104444208
H,0,0.8072958268,-3.290963075,2.1112478416
H,0,-0.4832889289,-4.50897444,2.0945346949

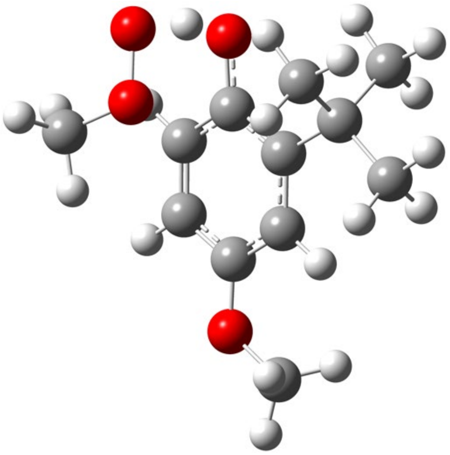

2-TS
Charge = 0 Multiplicity = 2
O,0,-1.0992107098,-2.607259064,0.3753563627
O,0,-0.7169271213,-1.7704565462,-0.6221108691
C,0,-1.8703375611,-1.2818241462,-1.3162130233
H,0,-2.342746897,-2.1105954922,-1.8498286878
H,0,-2.5663174414,-0.8391647925,-0.5994398393
H,0,-1.496343749,-0.5320213762,-2.014498797
C,0,-0.1798590205,2.1577127594,0.1459235168
C,0,1.2060444469,1.9703455753,-0.0111819704
C,0,1.8634731749,0.8092523355,0.4999162221
C,0,1.0687948867,-0.1104426666,1.1623327377
C,0,-0.325770629,0.0645091956,1.3247677804
C,0,-0.9449904872,1.2103381188,0.8006373291
H,0,-0.6588966492,3.0436931508,-0.2509986218
H,0,-2.0160383287,1.3409503836,0.9192441433
O,0,-1.0478090304,-0.8406553885,2.0053235844
H,0,-1.0779051257,-1.7443262493,1.4304178705
O,0,1.9725536059,2.8740254885,-0.6506355735
C,0,1.3684549414,4.0591990586,-1.168105989
H,0,0.9157806667,4.6450465194,-0.3632176757
H,0,2.1802076837,4.6232990393,-1.6245703971
H,0,0.6176792355,3.8123566713,-1.9238854771
H,0,1.4944611175,-1.016719605,1.5783750932

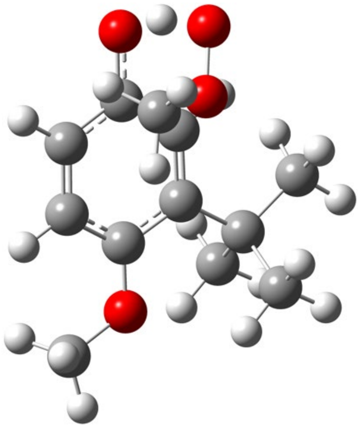

C,0,3.3708749245,0.5865036022,0.3202985162
C,0,3.8190519494,-0.7317329708,0.9640487904
H,0,3.631188667,-0.7443953429,2.0428212951
H,0,3.3212149683,-1.5964099454,0.5129404347
H,0,4.8961889244,-0.8467231534,0.8096174125
C,0,3.7198484957,0.5059060196,-1.1774114938
H,0,3.4887557327,1.4315071407,-1.7069116541
H,0,4.7913117193,0.3061845921,-1.2877305295
H,0,3.1713315438,-0.3141033671,-1.6537056
C,0,4.1669248332,1.7189859515,0.9948358428
H,0,5.2383437972,1.5127954085,0.8969191967
H,0,3.9643411178,2.6925486363,0.5461217943
H,0,3.9283463176,1.7708224592,2.0627462755

3-TS
Charge = 0 Multiplicity = 2
O,0,0.3801536058,1.0825813574,0.9158430164
O,0,-0.6892580382,0.3975900241,1.3923454567
C,0,-0.3192536681,-0.9606869989,1.6549955552
H,0,0.135838306,-1.3932888718,0.7606243191
H,0,0.3818146701,-0.9829993288,2.4934122065
H,0,-1.2476603319,-1.4733497269,1.9100980634
C,0,-4.1872570537,-0.4906310545,-0.8530196414
C,0,-3.8602904101,0.8700554427,-0.6622088106
C,0,-1.5397296144,0.3151634811,-1.1789073741
O,0,-0.2697644958,0.7084056644,-1.3677718757
H,0,0.1355409131,1.0048220828,-0.420352063
C,0,-4.9337239654,1.8673427114,-0.3045687937
H,0,-4.8634615764,2.1146717235,0.7612709843
H,0,-4.7632629786,2.7965370412,-0.8555282018
C,0,-6.3112507297,1.2966621571,-0.6262159701
H,0,-6.4641974042,1.2736903949,-1.7116996609
H,0,-7.100447625,1.915294803,-0.1896661374
C,0,-6.4575452998,-0.1286480225,-0.0972557256
C,0,-7.7822865629,-0.7391754764,-0.5168043436
H,0,-7.8342760201,-1.7869685502,-0.2068074584
H,0,-8.6021147134,-0.194418197,-0.0405169698
H,0,-7.9059868792,-0.6815517204,-1.6021067512
C,0,-6.2607722976,-0.2219104613,1.4122624576
H,0,-6.9759716276,0.4335852111,1.9180809691
H,0,-6.4287054467,-1.2495340447,1.7467897688
H,0,-5.2501800649,0.0760412762,1.7068436249
O,0,-5.4447859988,-0.9714219501,-0.7280831355
C,0,-3.2032444627,-1.4323379249,-1.2162747297
H,0,-3.5034209987,-2.4654941914,-1.3588457505
C,0,-1.8917869815,-1.0398278508,-1.3697379655
H,0,-1.1178963745,-1.7530498017,-1.6355151944
C,0,-2.5379905047,1.2491456681,-0.8328563717
H,0,-2.2517863706,2.2901071333,-0.7007604976

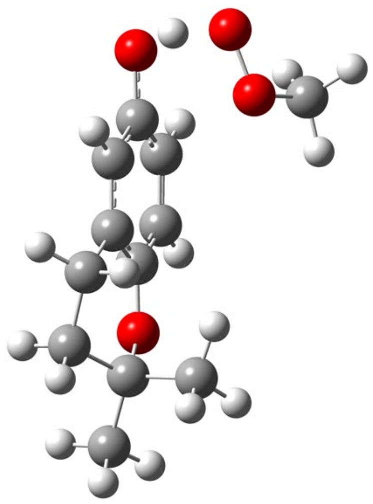

4-TS
Charge = 0 Multiplicity = 2
O,0,-2.0568300695,-0.8511116261,1.6790558124
O,0,-0.8139145116,-0.4040558195,1.9855356371
C,0,-0.858464668,0.9910434759,2.3053429933

H,0,-1.3663560753,1.5327953388,1.503888467
H,0,-1.3857876893,1.1225773833,3.2539120678
H,0,0.1828519312,1.3046302516,2.3925739286
C,0,2.3496994013,0.1903747747,-0.7975288263
C,0,1.9056103233,-1.1138804318,-0.501345702
C,0,-0.3826455016,-0.3093676154,-0.7055932124
O,0,-1.7063335329,-0.5463873746,-0.6895743007
H,0,-1.9954201481,-0.7966284451,0.3046395699
C,0,2.9019168044,-2.2193670483,-0.2514241069
H,0,2.9574130352,-2.4263020783,0.8239138204
H,0,2.5518247573,-3.137640234,-0.7313701125
C,0,4.2750526032,-1.8253249286,-0.7850141933
H,0,4.2742849457,-1.8515965328,-1.8811774012
H,0,5.0438213397,-2.5193134158,-0.4334732321
C,0,4.651188569,-0.412197373,-0.3460564569
C,0,5.9624634247,0.029666572,-0.9701623834
H,0,6.1712226932,1.0738496084,-0.7193903825
H,0,6.7772045513,-0.5901263883,-0.5855577847
H,0,5.9251150627,-0.0752958206,-2.0583495806
C,0,4.6814684299,-0.2520065596,1.1705596037
H,0,5.3835166577,-0.9709223464,1.6034368847
H,0,5.0083799661,0.7586480683,1.4313438127
H,0,3.6956778095,-0.4209824237,1.6138267126
O,0,3.658700043,0.5218797291,-0.8687858661
C,0,1.4426936965,1.24948159,-1.0686356401
C,0,0.0889531585,0.9856691139,-1.0087502144
H,0,-0.6337291303,1.7757395913,-1.1935156197
C,0,0.538370497,-1.346763935,-0.4633788045
H,0,0.1610450887,-2.3424250637,-0.2423411682
C,0,1.9763343145,2.6146325403,-1.3952277547
H,0,2.5991066333,2.9959553925,-0.5798112787
H,0,2.6028492172,2.5858462345,-2.2924318708
H,0,1.1545793724,3.3127127959,-1.565076418

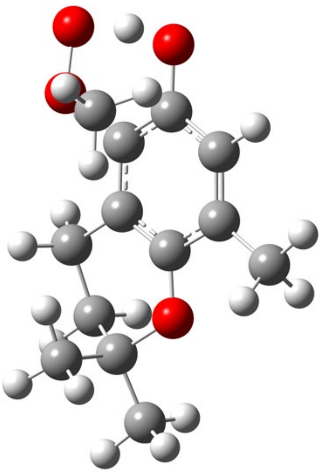

5-TS

Charge = 0 Multiplicity = 2

O,0,-1.4881725494,-0.634621295,2.7349508429
O,0,-0.1394672532,-0.6435057876,2.6012121607
C,0,0.39663811,0.6703298991,2.7963695002
H,0,-0.1811108346,1.3923257384,2.2158184442
H,0,0.36163883,0.913750574,3.8617175705
H,0,1.429360162,0.6196561119,2.4448829903
C,0,2.0749780999,-0.2039253854,-0.9257149279
C,0,1.6425447459,-1.4467134514,-0.4391725442
C,0,-0.5523308279,-0.4700579242,-0.0795215057
O,0,-1.8328437523,-0.5569513601,0.3316400243
H,0,-1.8469305265,-0.6535798582,1.3871489333
C,0,2.597027514,-2.6156561499,-0.3921355417
H,0,2.876188903,-2.8212321833,0.649506525
H,0,2.0919107846,-3.5151421316,-0.7576279082
C,0,3.8381252362,-2.3401078048,-1.2345629188
H,0,3.5927035647,-2.4195891713,-2.3002053452
H,0,4.6241804384,-3.0690061991,-1.0173448632
C,0,4.3783611972,-0.9365765985,-0.9809552094
C,0,5.5433948598,-0.6162689172,-1.8997286901

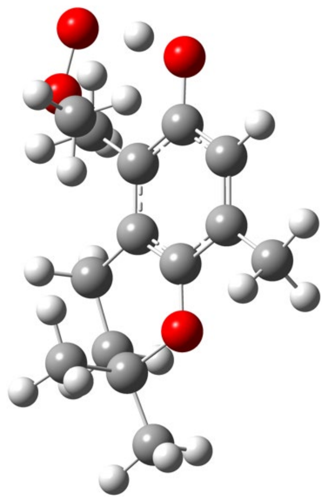

H,0,5.8584238836,0.4231657288,-1.7683284552
H,0,6.3879981299,-1.2684761505,-1.660717006
H,0,5.2629971582,-0.7741941746,-2.9452657851
C,0,4.7485471353,-0.7010902339,0.4801876128
H,0,5.4937493342,-1.4377414028,0.7949880434
H,0,5.1723324741,0.3001285009,0.6001743866
H,0,3.8776275442,-0.7883124328,1.1367928028
O,0,3.3447038329,0.0255647084,-1.3415151923
C,0,1.2002002322,0.9108098189,-1.0392367686
C,0,-0.0989724003,0.7558613658,-0.6078560921
H,0,-0.7969133827,1.5873358589,-0.654329451
C,0,0.3187292862,-1.5858823742,-0.0013531668
C,0,-0.1627612338,-2.8986936124,0.5432054543
H,0,-0.2135248475,-3.6503622055,-0.2531702352
H,0,0.5274336492,-3.2779826769,1.3033670446
H,0,-1.1537700247,-2.8062724453,0.9877438643
C,0,1.7124398969,2.2074543343,-1.5963110802
H,0,2.5434713213,2.5906243356,-0.9955082424
H,0,2.086903201,2.0760683383,-2.6165575604
H,0,0.916322108,2.9542686133,-1.6098777108

6-TS
Charge = 0 Multiplicity = 2
O,0,-2.849804274,-0.2996522033,0.9578077707
O,0,-2.0843810392,0.080267201,-0.0935468401
C,0,-1.5978356242,-1.0713037786,-0.7933326385
H,0,-1.1375727622,-1.7619651362,-0.0832821119
H,0,-2.4322171825,-1.5502727059,-1.3124457424
H,0,-0.8633216505,-0.6923614284,-1.5056821535
C,0,1.8774583505,1.5239315056,0.0192363745
C,0,2.0089627627,0.1830990074,0.4468157981
C,0,-0.07225157,0.4576879533,1.6728059205
O,0,-0.9844851246,-0.0944339123,2.4964937659
H,0,-1.9047448046,-0.1779550541,1.981203603
C,0,3.1748039947,-0.6566258304,-0.0105531072
H,0,3.9102168716,-0.7359862728,0.7989865974
H,0,2.8286071829,-1.672449838,-0.221822141
C,0,3.8177297453,-0.0378630921,-1.2467587621
H,0,3.1619515944,-0.1669832976,-2.1161535134
H,0,4.7732009334,-0.5197492004,-1.4729322426
C,0,4.0549149074,1.4575216335,-1.0499251221
C,0,4.5847135579,2.1024616094,-2.3181765147
H,0,4.6652274156,3.1859102955,-2.1898644944
H,0,5.577886417,1.7040324546,-2.5435403606
H,0,3.9219381956,1.8916549651,-3.1625434538
C,0,4.9594968996,1.7611508113,0.1400976657
H,0,5.9174892521,1.2470384956,0.0170713453
H,0,5.1440112447,2.8374625072,0.2014327843
H,0,4.5095394777,1.4342542053,1.0820173443
O,0,2.7732507634,2.1115785952,-0.8102051563
C,0,0.783563831,2.3334717997,0.3980697476
C,0,-0.1946353054,1.8100791373,1.241744114
C,0,1.0186692749,-0.3279670716,1.2646588441
H,0,1.0643663725,-1.3619728394,1.5972008867
C,0,0.7256642659,3.7381973315,-0.1404728267

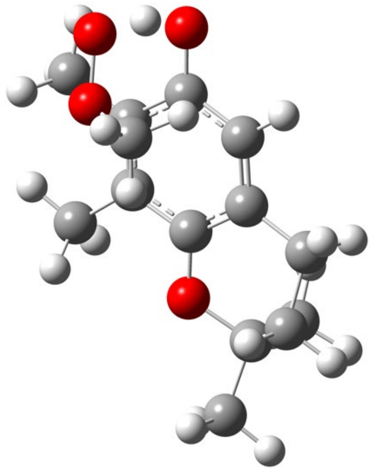

H,0,1.5902602519,4.3199652902,0.1960219471
H,0,0.752275628,3.731786624,-1.2348142648
H,0,-0.1800476297,4.2550046441,0.1744281999
C,0,-1.3768094327,2.6292785051,1.6807054537
H,0,-1.966507205,2.0993254568,2.4287184392
H,0,-1.0542723495,3.5816417897,2.1110519133
H,0,-2.0287522365,2.8574448423,0.8301109309

7-TS

Charge = 0 Multiplicity = 2

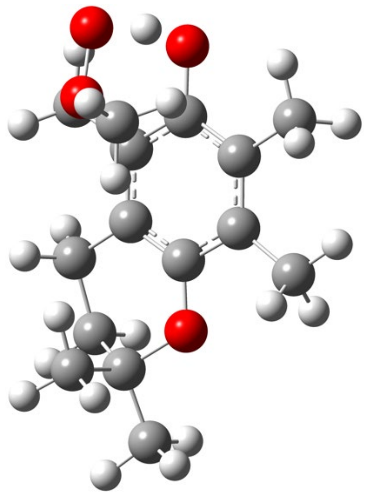

O,0,-1.5460622363,-1.261096754,2.4752134412
O,0,-0.1989568739,-1.2125134168,2.369430304
C,0,0.3301397231,-0.1800825386,3.2108306103
H,0,0.2402011814,-0.4931450742,4.2544076636
H,0,1.3785196465,-0.0768635618,2.9255901365
H,0,-0.2197118437,0.7484905917,3.0397920678
C,0,2.0915214164,0.5163540968,-0.7034851546
C,0,1.4599626961,-0.7220876341,-0.8985730941
C,0,-0.536593253,0.2339681897,0.1042502198
O,0,-1.8278095777,0.1076564171,0.4758940067
H,0,-1.884444304,-0.5268961021,1.3112554822
C,0,2.2039068593,-1.8662310679,-1.5435755187
H,0,2.4423639374,-2.6247027998,-0.7864284779
H,0,1.5566915349,-2.3579236762,-2.2765054034
C,0,3.4759613612,-1.3730106371,-2.2246864945
H,0,3.2240869875,-0.8460377888,-3.1526621779
H,0,4.1285480576,-2.2119909244,-2.4831225991
C,0,4.2409136102,-0.404948185,-1.3283066687
C,0,5.4471493604,0.1765475969,-2.0436136134
H,0,5.9323120306,0.9334092099,-1.4201018885
H,0,6.1689090449,-0.618606889,-2.2503255426
H,0,5.1481137955,0.6340298903,-2.9912725422
C,0,4.6409630773,-1.0275963906,0.0059125769
H,0,5.2543513404,-1.9162717501,-0.1707528322
H,0,5.2229427827,-0.310306421,0.5918286629
H,0,3.7655232834,-1.3232554884,0.5917777435
O,0,3.3853388626,0.7406516793,-1.0510915374
C,0,1.4238993311,1.6328049495,-0.1426067852
C,0,0.1089600739,1.4879196806,0.2809108798
C,0,0.1346584989,-0.8670491989,-0.4846407265
C,0,-0.5625791887,-2.187133843,-0.6493587657
H,0,-0.6865760168,-2.4299225743,-1.7109858734
H,0,0.0300908735,-2.9922642202,-0.2021099755
H,0,-1.5483057741,-2.1830979622,-0.1853279912
C,0,2.1852673647,2.923918403,-0.0082466148
H,0,2.6626746634,3.1910968722,-0.9554604996
H,0,1.5341596281,3.7450800707,0.2897281478
H,0,2.9837044937,2.8304771256,0.736624943
C,0,-0.627971831,2.6217164358,0.943917694
H,0,-0.0390016225,3.0385430239,1.7667137123
H,0,-0.8208685177,3.4355572055,0.2363496869
H,0,-1.5847544773,2.28414246,1.341024798

8-TS

Charge = 0 Multiplicity = 2

O,0,0.3413177869,1.4144645063,-0.7207702325
O,0,0.2150358721,0.1201110763,-0.3727310111
C,0,0.8916536594,-0.1279548271,0.8668827234
H,0,0.5356974595,0.5793753308,1.6195652026
H,0,0.640697088,-1.1536858416,1.1388209469
H,0,1.9673835781,-0.0163910584,0.7094895231
C,0,-2.3129032856,0.7042825958,0.4372134472
C,0,-2.3689714037,0.0518293477,1.6823617637
C,0,-2.9287208231,-1.2066290513,1.7897503699
C,0,-3.4454991499,-1.8607813403,0.6476821753
C,0,-3.384581181,-1.202773575,-0.6026590624
C,0,-2.8333193718,0.0590593851,-0.7023253209
H,0,-1.9664818023,0.5550130655,2.5566081483
H,0,-2.9763861334,-1.7080650146,2.7520791537
H,0,-3.7808037961,-1.7020093827,-1.4821637682
H,0,-2.7869891973,0.5688862071,-1.6603972709
O,0,-1.8053140046,1.9605377849,0.3546853337
H,0,-0.9237827654,1.9222836859,-0.1839053677
N,0,-3.95481189,-3.1245679001,0.7429705567
H,0,-4.1864288754,-3.4820668752,1.6598919027
H,0,-4.5072710842,-3.4792297991,-0.0259404332

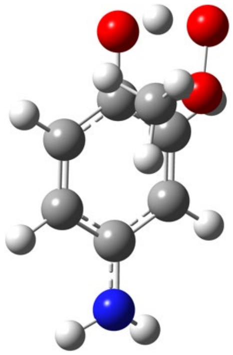

12-TS

Charge = 0 Multiplicity = 2

O,0,-0.6983298626,1.1972665753,2.2140332074
O,0,0.4439467982,0.6154500798,2.6657923796
C,0,0.1336904046,-0.3152514066,3.7082587808
H,0,-0.6126201529,-1.0286552331,3.3490284241
H,0,1.0736060916,-0.8165227463,3.9427091607
H,0,-0.2439475933,0.2320725007,4.5758403503
C,0,0.5299352868,-2.2351232401,0.8597204683
C,0,0.2872807592,-0.9133586312,0.426054073
C,0,1.3595213028,-0.1399208153,-0.0850305574
C,0,2.6278493725,-0.6730486254,-0.1576256491
C,0,2.882512183,-1.9972160883,0.2720582483
C,0,1.8024573021,-2.761991998,0.7718385587
H,0,-0.2977920037,-2.8166117615,1.2538237314
H,0,1.1575012968,0.8763385045,-0.4103141051
H,0,3.4337672937,-0.0636303733,-0.5527606433
H,0,1.9898934419,-3.7807327351,1.0995521328
O,0,-0.9511087827,-0.4170359648,0.4675878808
H,0,-0.9618030134,0.4186892099,1.1590080889
C,0,4.2008809656,-2.607930956,0.2315263782
H,0,4.2457981793,-3.6489148376,0.5501504612
C,0,5.3531741388,-2.0084507125,-0.1471187752
H,0,5.3683534903,-0.966513565,-0.4639222658
C,0,6.6274845857,-2.7051808039,-0.1507117537
H,0,6.6197848204,-3.7473824844,0.1660895926
C,0,7.7783427904,-2.122140092,-0.5207031346
H,0,7.8032506164,-1.0827299826,-0.839698312
H,0,8.7162832887,-2.6679318173,-0.513682721

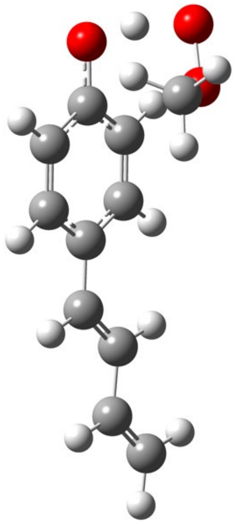

13-TS

Charge = 0 Multiplicity = 2

O,0,-2.1593430217,0.4645607255,0.540052821
O,0,-0.99872382,1.0949408417,0.2182649687
C,0,-0.84741667,2.2694812493,1.0218628222
H,0,-0.9181210549,1.9993317107,2.0787878742
H,0,0.1418495294,2.6621223693,0.7834772869
H,0,-1.6270774385,2.9880140731,0.7560324799
C,0,2.439259418,-0.0574586114,2.203682365
C,0,2.9044432357,-0.5997654339,0.9826880046
C,0,1.9761697069,-1.2622191059,0.1447524927
C,0,0.65236111,-1.3759366735,0.5093215462
C,0,0.1983185496,-0.8255962681,1.7341634685
C,0,1.1135848853,-0.155184413,2.5747106866
H,0,3.1464624911,0.4507232279,2.8534075928
H,0,2.3012876123,-1.6928038464,-0.7963713654
H,0,-0.064846721,-1.8826583813,-0.1296361758
H,0,0.7536459184,0.2685293322,3.5071329481
C,0,4.3133643107,-0.4458307827,0.6531801137
H,0,4.9088569667,0.0497249029,1.4174705149
C,0,4.8996207358,-0.852754621,-0.4939583379
H,0,4.2949888621,-1.3265830579,-1.2653455233
C,0,6.317749144,-0.7108501663,-0.8340918386
C,0,7.2801358694,-0.2099035573,0.0616457871
C,0,6.7360149585,-1.0973264184,-2.118350139
C,0,8.6106687737,-0.0937661416,-0.3241772562
H,0,6.9925643111,0.0853269451,1.0659069648
C,0,8.069453567,-0.9802333397,-2.5036890992
H,0,6.0018060034,-1.4911028459,-2.8164579148
C,0,9.011822079,-0.4762403317,-1.6078171821
H,0,9.3410893448,0.294166255,0.3791291926
H,0,8.3715843003,-1.2825039336,-3.5016012861
H,0,10.0523278403,-0.3840371143,-1.9031689905
O,0,-1.0723459574,-0.9678480278,2.116580251
H,0,-1.7057508398,-0.446178561,1.4076199271

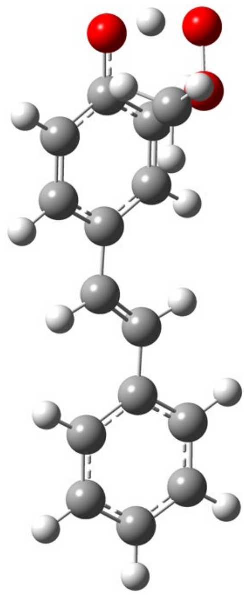

14(1)-TS

Charge = 0 Multiplicity = 2

O,0,0.1954518066,-0.7868279441,-1.4273113828
O,0,0.0037969821,0.5518822932,-1.575099509
C,0,-1.1496504859,0.8014799487,-2.3892838416
H,0,-1.3422843046,1.871495614,-2.2955771678
H,0,-0.9156744438,0.5373195355,-3.4235877144
H,0,-1.9932880471,0.214588364,-2.0204895595
C,0,-2.4302734422,0.8629666494,0.7417899041
C,0,-1.0889440438,0.4511916432,0.8519906554
C,0,-0.0742420701,1.4237283955,1.0620596823
C,0,-0.4057857315,2.7887257278,1.145445525
C,0,-1.7241907519,3.1766214579,1.0298317059
C,0,-2.7357136875,2.2072901358,0.8335597562
H,0,-3.1983726165,0.1156814857,0.575459203
H,0,-1.9698231863,4.2306680149,1.0932738036
O,0,-0.7226391268,-0.8201362547,0.7584730996
H,0,-0.2493910614,-0.9681274693,-0.2646051524
O,0,1.2123367917,1.0776507283,1.1691004943

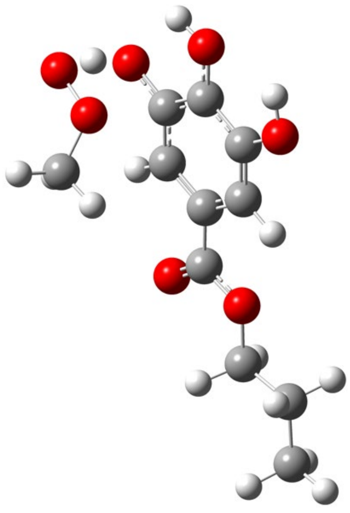

H,0,1.3004880721,0.108635465,1.1105771357
O,0,0.5663782457,3.7255723728,1.3368551319
H,0,1.4362574088,3.298904949,1.3867052914
C,0,-4.1633648972,2.6105445788,0.6988957673
O,0,-5.0746195338,1.8199114068,0.517068912
O,0,-4.3469901307,3.9247165097,0.7919887621
C,0,-5.7051476029,4.4009559682,0.655617883
H,0,-6.315946627,3.9319231265,1.4329703657
H,0,-6.08154672,4.0932908398,-0.3248709222
C,0,-5.6804798141,5.9072314928,0.7969083036
H,0,-5.0180197495,6.3278069929,0.0328195812
H,0,-5.2647300371,6.168298674,1.7759628872
C,0,-7.0875676926,6.4800370573,0.6498143509
H,0,-7.758905889,6.0722778619,1.4123262332
H,0,-7.0768850056,7.5674960754,0.7567227289
H,0,-7.507218608,6.2412093031,-0.3326389139

14(2)-TS

Charge = 0 Multiplicity = 2

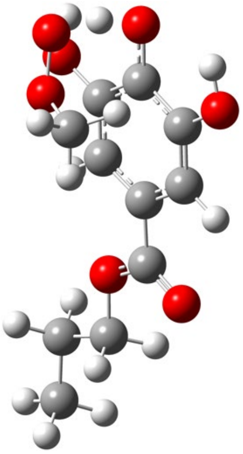

O,0,0.3576279034,-1.576441177,-0.7353709812
O,0,-0.8861804222,-1.4282780489,-1.2510037218
C,0,-0.8578691279,-0.5103905528,-2.3530160502
H,0,-0.3692577315,0.4171927116,-2.043098703
H,0,-0.3153710483,-0.9725691865,-3.1810258362
H,0,-1.9027806311,-0.3408707787,-2.6154059688
C,0,-3.6996156143,-0.6054995462,0.988227309
C,0,-2.3493493579,-0.8290575067,1.2156490824
C,0,-1.3821167791,0.1308295411,0.8157142516
C,0,-1.813128141,1.3233630966,0.1775902726
C,0,-3.1589057876,1.5505013615,-0.0492980431
C,0,-4.0849262304,0.5821412261,0.359267325
H,0,-4.4257427064,-1.3485545871,1.2943188763
H,0,-3.4842208811,2.4612062788,-0.5388157317
O,0,-1.940704895,-1.9738155131,1.799092934
H,0,-0.9747273765,-1.9563258558,1.9157549797
O,0,-0.0920462024,-0.0708740348,1.0428983555
H,0,0.2852225171,-0.8035795828,0.2811882571
O,0,-0.8879790384,2.2239674387,-0.2172342984
H,0,-0.0037327166,1.8957916883,0.0240710263
C,0,-5.5275022962,0.8656372106,0.090527836
O,0,-5.9199757879,1.8731039823,-0.4729892927
O,0,-6.3397844056,-0.0902997174,0.5285699481
C,0,-7.7551827111,0.1103802616,0.3105281117
H,0,-7.9266349458,0.2191842377,-0.764790317
H,0,-8.053046064,1.035808011,0.8130475886
C,0,-8.4817289813,-1.0912903036,0.8737534167
H,0,-8.2483993796,-1.1838115689,1.9398001865
H,0,-8.1175654985,-1.9957667872,0.3749261071
C,0,-9.9877711235,-0.9466512002,0.6723352381
H,0,-10.2353337543,-0.8670931464,-0.3910314952
H,0,-10.5179722752,-1.811626949,1.0782574155
H,0,-10.3666755096,-0.0513970028,1.1756399216

14(3)-TS

Charge = 0 Multiplicity = 2

O,0,0.3194060688,-0.7391793141,-1.4093510489
O,0,0.0392604922,0.5808850804,-1.5810718111
C,0,-1.117413792,0.7370205017,-2.4136303635
H,0,-1.3787998554,1.7944042217,-2.3487316143
H,0,-0.8546758857,0.4629788591,-3.4384037923
H,0,-1.9266634311,0.1064997214,-2.0394153799
C,0,-2.437678591,0.8000301331,0.7064672952
C,0,-1.0817979951,0.4401190434,0.8338787939
C,0,-0.1072496362,1.4493308663,1.059802667
C,0,-0.4886314734,2.8019081017,1.1333554461
C,0,-1.8186424327,3.1382218587,1.0022434204
C,0,-2.7916032994,2.1332768517,0.7961555702
H,0,-3.168961308,0.0190371185,0.533337388
H,0,-2.1136007011,4.1804579465,1.0616357345
O,0,-0.6667311485,-0.8165320664,0.7460630299
H,0,-0.1519924206,-0.9403799309,-0.2581272686
O,0,1.1898358041,1.153520665,1.1898424289
H,0,1.3167021822,0.1878209961,1.1610816815
O,0,0.4475004144,3.7722933026,1.3351369831
H,0,1.3298587022,3.3741690324,1.4014951349
C,0,-4.2094707982,2.5694806708,0.665660301
O,0,-4.5647722842,3.7349127396,0.7302638999
O,0,-5.052891415,1.5599774997,0.4687505757
C,0,-6.4506442024,1.9004029643,0.3268073111
H,0,-6.5558845981,2.5818962343,-0.5229122495
H,0,-6.7753463343,2.4148749427,1.2364779816
C,0,-7.2159614249,0.6132272838,0.1093563943
H,0,-7.0442991384,-0.0534787543,0.9611822656
H,0,-6.828883787,0.113766892,-0.7852462918
C,0,-8.7073362209,0.8973425891,-0.0475473087
H,0,-8.8937632793,1.5540916543,-0.9033059538
H,0,-9.2638285146,-0.0295984202,-0.2063860729
H,0,-9.1101826963,1.384238715,0.8463168526

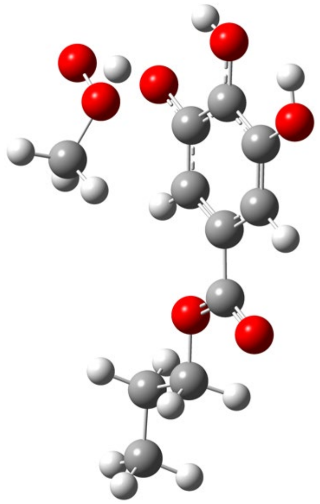

15(1)-TS

Charge = 0 Multiplicity = 2

O,0,-2.7711776717,0.1652200469,-0.2563104668
O,0,-1.6839598834,0.2575884163,-1.0620146189
C,0,-1.2625401263,1.6223262977,-1.1786828531
H,0,-0.2885892795,1.5853366824,-1.669361854
H,0,-1.9870228131,2.1642771697,-1.7918930745
H,0,-1.186306402,2.0681822209,-0.1847940733
C,0,1.3685836363,-1.5049621888,-0.5053152041
C,0,0.117802132,-1.5206547058,0.1145522781
C,0,-0.2380001827,-0.5076996867,1.0450373822
C,0,0.6950052472,0.5033174972,1.3439690579
C,0,1.9314208426,0.5026775345,0.7337820055
C,0,2.2825820326,-0.5012914895,-0.2045327831
H,0,1.6132139099,-2.2882840078,-1.2170790214
H,0,0.4077643825,1.2753239663,2.0512817341
H,0,2.6509898528,1.2842352952,0.9629089575
O,0,-1.4481290404,-0.5518988692,1.6169543931
H,0,-2.1835220391,-0.2479873258,0.8805303979

O,0,-0.7594689561,-2.5002088467,-0.1833955851
H,0,-1.5615372919,-2.3922598145,0.3569649717
C,0,3.64909189,-0.4741919497,-0.8340119638
H,0,3.6827624901,-1.1762430529,-1.6755611042
H,0,3.8193199617,0.5296666097,-1.2359216154
C,0,4.7773094912,-0.8437771501,0.1569700299
H,0,4.6495521544,-0.2349802797,1.0655534637
C,0,6.1546852987,-0.4854494773,-0.4464371312
H,0,6.1736017908,-0.8663065917,-1.4799671104
C,0,7.3114176125,-1.1632244151,0.3180645007
H,0,7.1881022332,-2.2494595073,0.2742220152
H,0,7.2554620775,-0.8693805121,1.3747251082
C,0,8.6732062948,-0.8126492561,-0.2322637819
C,0,9.1035099795,-1.3458914048,-1.4511427814
C,0,9.515716362,0.0783415325,0.441416616
C,0,10.3425273302,-0.997811373,-1.9903401307
H,0,8.465290418,-2.0438751971,-1.9870169856
C,0,10.7523302059,0.4270906982,-0.0913335036
H,0,9.2120462553,0.5135003158,1.3914971362
C,0,11.1690513536,-0.109672589,-1.31382004
H,0,10.6813626164,-1.4119229795,-2.9352261518
O,0,11.6309640871,1.2925059601,0.5148249111
H,0,11.2557659046,1.6257676296,1.3430390877
O,0,12.3897591148,0.2274676579,-1.849907393
H,0,12.8336622464,0.8565933702,-1.260612142
C,0,4.6448590456,-2.3189061143,0.5411361411
H,0,5.3142579867,-2.592261708,1.3603227995
H,0,3.6233790629,-2.5439777184,0.8639543564
H,0,4.8741201806,-2.9621046634,-0.3179863947
C,0,6.3661336225,1.0308658704,-0.4714768356
H,0,7.2760530478,1.2969234164,-1.0172410254
H,0,6.4672289588,1.4115061108,0.5529460354
H,0,5.5346195765,1.5592105759,-0.944796754

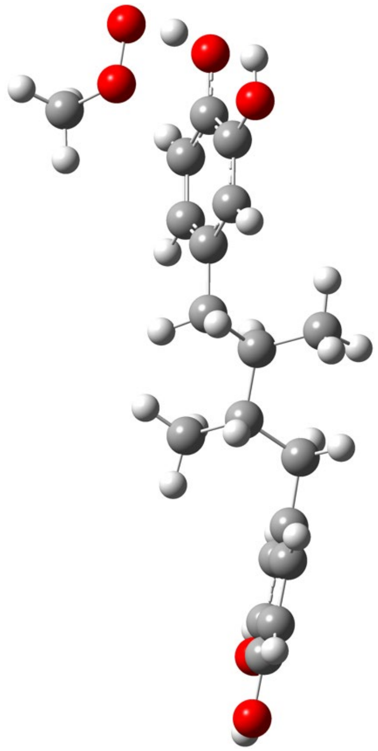

15(2)-TS
Charge = 0 Multiplicity = 2
O,0,-3.0325572306,-1.3793733733,-1.6697757756
O,0,-2.4613318153,-0.2399041074,-2.1384841343
C,0,-1.3517500945,-0.5477132827,-2.9909007296
H,0,-0.7005158445,-1.2733041588,-2.4991437617
H,0,-1.7317887096,-0.9459232312,-3.9352047796
H,0,-0.8350886134,0.4005415521,-3.1497299777
C,0,0.1868468494,-0.1670841586,-0.0253526882
C,0,-1.1992899549,-0.0833290712,0.1987482274
C,0,-1.8418639242,1.1832477135,0.1470243048
C,0,-1.1003298732,2.3327633918,-0.1464717514
C,0,0.2599134983,2.2170291705,-0.3689387406
C,0,0.9286412118,0.9666453585,-0.3067826079
H,0,0.6542604936,-1.1475607194,0.0169759115
H,0,-1.6066415729,3.2909672601,-0.1977216486
H,0,0.8384117641,3.1079134437,-0.6018054575
O,0,-3.166743178,1.2785475204,0.3670155974
H,0,-3.5212018702,0.3964805969,0.5771092573
O,0,-1.9435647881,-1.1646811595,0.4621950278
H,0,-2.5149404687,-1.4039188783,-0.4300351198

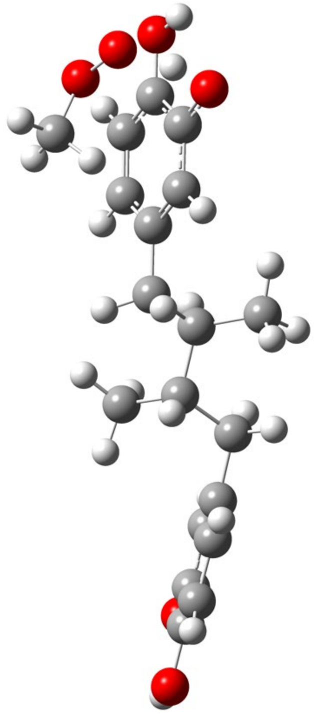

C,0,2.4160946179,0.9094163052,-0.5345859724
H,0,2.7179396283,-0.1282477749,-0.7220324056
H,0,2.6490674985,1.4843007159,-1.4366298361
C,0,3.2388932346,1.4458890193,0.6588176058
H,0,2.809647424,2.4126255281,0.9662151198
C,0,4.6970333044,1.7099939335,0.2178984735
H,0,5.0255628523,0.8424247418,-0.3762314434
C,0,5.6548459812,1.8363307424,1.4203617717
H,0,5.6381002879,0.9062420343,1.9967247919
H,0,5.2915712588,2.6362079319,2.0792138215
C,0,7.0804049277,2.1272786132,1.0155637274
C,0,7.8583341446,1.1440683191,0.396136426
C,0,7.6412468873,3.3927919412,1.2177100994
C,0,9.1629907547,1.4153208197,-0.0178529128
H,0,7.4428790301,0.1526090942,0.2347646478
C,0,8.9435089934,3.6658732776,0.812944112
H,0,7.0638840714,4.1813385681,1.6962125779
C,0,9.7084695789,2.6765679659,0.1870212131
H,0,9.7703129678,0.6539263182,-0.4980549323
O,0,9.5597585791,4.8814579183,0.9890530459
H,0,8.9619359123,5.4973102457,1.4377947148
O,0,10.9958766522,2.9376221845,-0.2201347837
H,0,11.2108077342,3.8622450211,-0.0217234784
C,0,3.1182942538,0.4707244025,1.831256199
H,0,3.5493401691,0.8758826333,2.7501160584
H,0,2.0672557781,0.2418902659,2.0348461208
H,0,3.6280867929,-0.4732689059,1.6007178538
C,0,4.7824370179,2.9681902556,-0.6508799229
H,0,5.7751345266,3.0802166515,-1.0967749751
H,0,4.5882124797,3.8581855356,-0.038760317
H,0,4.0545447813,2.9581598298,-1.4666775548

15(3)-TS

Charge = 0 Multiplicity = 2

O,0,1.8720640815,-1.3107235859,0.8762875595
O,0,1.2254672553,-2.4607979046,0.5556682731
C,0,0.3759163385,-2.8743879653,1.6324793445
H,0,-0.2413960568,-3.6766624709,1.2244919133
H,0,0.9979691634,-3.2411316427,2.4530414173
H,0,-0.238109391,-2.0322446215,1.9589339718
C,0,-9.8930843801,-2.9198559389,-1.2931031408
C,0,-11.1925991905,-2.7675510591,-0.8203979801
C,0,-11.6191874736,-1.5338319754,-0.3189270621
C,0,-10.7395491024,-0.4584650788,-0.3029553944
C,0,-9.4385636646,-0.6150364909,-0.7816049132
C,0,-8.9976300496,-1.8447423369,-1.2813456094
H,0,-9.5835029893,-3.8909428993,-1.6743049067
H,0,-11.0862951563,0.4956777295,0.0823776994
H,0,-8.7592508282,0.2337316691,-0.7690460755
O,0,-12.9006668494,-1.3720029151,0.1525923207
H,0,-13.3792735993,-2.2111329321,0.0690548955
O,0,-12.1261130522,-3.776257678,-0.8063167189
H,0,-11.7516968451,-4.5868160472,-1.1813455949
C,0,-7.5783469057,-2.0072994161,-1.7711142487
H,0,-7.4894372935,-2.9478546196,-2.3297205303

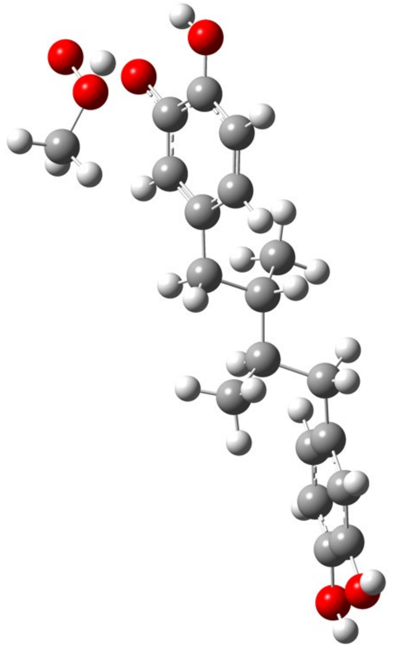

H,0,-7.3569743812,-1.1957348214,-2.4710578283
C,0,-6.5318665503,-2.0200296064,-0.6357237816
H,0,-6.7091076811,-1.139588073,0.0018570063
C,0,-5.1064283435,-1.8850018603,-1.2181048046
H,0,-5.0208391891,-2.5971873313,-2.0541885899
C,0,-4.0261612397,-2.2537444858,-0.1783849863
H,0,-4.1604379461,-3.2942474772,0.1338244562
H,0,-4.1557922699,-1.6211853091,0.709028522
C,0,-2.6240219305,-2.0948440631,-0.7051558972
C,0,-2.1422140456,-2.9904684962,-1.6948482478
C,0,-1.7907488341,-1.0804954632,-0.2683652317
C,0,-0.8796129105,-2.8628900341,-2.2443795694
H,0,-2.7873725174,-3.7990242461,-2.029311831
C,0,-0.4973841007,-0.9301782229,-0.8009789505
H,0,-2.1131836162,-0.380332614,0.4979384053
C,0,-0.0481848949,-1.824301675,-1.8105173488
H,0,-0.5162402958,-3.5492041947,-3.0021666093
O,0,0.331679808,0.0365712091,-0.3873336829
H,0,1.1103568123,-0.422088679,0.2142840245
O,0,1.1813746126,-1.6900520557,-2.3435472236
H,0,1.620064957,-0.9102357442,-1.9611332799
C,0,-6.7226449927,-3.2776289997,0.214477178
H,0,-6.1204867611,-3.2563592975,1.1262195591
H,0,-7.7697683647,-3.3820939098,0.5157082543
H,0,-6.4474291177,-4.1735504231,-0.3568524056
C,0,-4.8556786642,-0.4687485064,-1.7439218262
H,0,-3.9048792934,-0.4045315556,-2.2814632184
H,0,-4.8147661304,0.239721262,-0.9067681247
H,0,-5.6407431302,-0.1360711469,-2.4274281882

15(4)-TS

Charge = 0 Multiplicity = 2

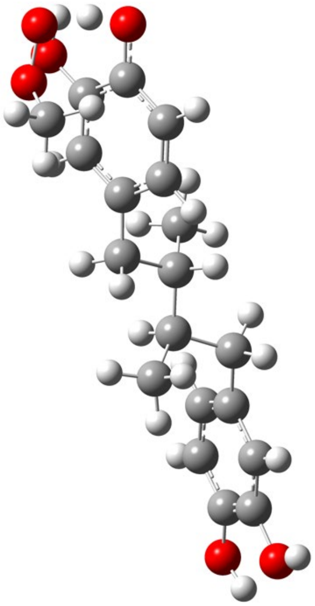

O,0,0.9299144731,-0.5209700126,1.3850470082
O,0,-0.2403736077,-0.7369885805,2.0347205734
C,0,-0.4777538432,-2.1428226889,2.1821584222
H,0,0.2154145344,-2.5417188297,2.927103411
H,0,-1.5099727178,-2.229203376,2.5253151979
H,0,-0.3394595584,-2.639922036,1.2197071052
C,0,-11.2939202793,-1.6887402006,-0.4839208774
C,0,-12.6311129976,-1.5081129639,-0.1443748232
C,0,-13.107127517,-0.2372791759,0.1919520501
C,0,-12.2367104233,0.8457215804,0.183344563
C,0,-10.8977159904,0.6606471363,-0.1610767165
C,0,-10.4086712818,-0.6053913718,-0.4997622725
H,0,-10.9482572591,-2.6882153974,-0.7403300942
H,0,-12.6206978867,1.8281853179,0.4410721498
H,0,-10.2263206478,1.5157077204,-0.1714511469
O,0,-14.4273338536,-0.046833987,0.5256918055
H,0,-14.9008368679,-0.8894108387,0.4471028586
O,0,-13.5577181657,-2.5226315454,-0.1133731253
H,0,-13.1548557076,-3.3550038677,-0.4008077633
C,0,-8.9525474084,-0.7917393435,-0.8552466019
H,0,-8.8047291929,-1.7922954111,-1.2812231949
H,0,-8.6910525415,-0.0696571027,-1.6347785613
C,0,-7.9997364319,-0.6329623654,0.3490708711

H,0,-8.2512819172,0.3083302775,0.8623863694
C,0,-6.5364404195,-0.5136512853,-0.1331018008
H,0,-6.3570058036,-1.3234387384,-0.8577335101
C,0,-5.5387298248,-0.6994278362,1.0325118199
H,0,-5.6634945532,-1.6983260171,1.4620543083
H,0,-5.7672064804,0.033912799,1.8159635307
C,0,-4.1056069523,-0.5468694445,0.600748968
C,0,-3.5088094933,-1.5529394658,-0.2003706885
C,0,-3.3689949361,0.5821122909,0.9410852637
C,0,-2.206737891,-1.4317688573,-0.6386128573
H,0,-4.0903708169,-2.4326419857,-0.4624714049
C,0,-2.0553314949,0.7224664917,0.4913167975
H,0,-3.8042232929,1.3705593332,1.5484821709
C,0,-1.452312426,-0.292458471,-0.2997759676
H,0,-1.7325009343,-2.2023638828,-1.2387571106
O,0,-1.3554237655,1.826184664,0.8234954433
H,0,-0.4842550239,1.8018213231,0.3915176725
O,0,-0.1867949283,-0.1292316766,-0.7072175089
H,0,0.4657280463,-0.2703686977,0.146767794
C,0,-8.223364396,-1.7904287031,1.3242696438
H,0,-7.7044198424,-1.6389347366,2.2740910335
H,0,-9.2891563997,-1.9034781367,1.5470793867
H,0,-7.8705995969,-2.7337339019,0.8878317377
C,0,-6.2861628002,0.8330921864,-0.8179401175
H,0,-5.2945395728,0.8687057665,-1.2789859832
H,0,-6.3416896207,1.644320347,-0.0809549477
H,0,-7.0181596917,1.0408856973,-1.6020588816

16(1)-TS

Charge = 0 Multiplicity = 2

O,0,-0.8632960941,-0.1614449814,3.9365168935
O,0,-2.2197691081,-0.1685459159,3.7822633538
C,0,-2.7509049898,1.1417439631,4.0045283065
H,0,-3.8025044775,1.0797177984,3.7192083342
H,0,-2.6510518779,1.3922354707,5.0639898023
H,0,-2.2196607402,1.868383195,3.3853052664
C,0,-3.8273217051,-0.3697492532,0.6374548604
C,0,-4.08112411,0.8676676934,0.0466278274
C,0,-5.417309567,1.1972707311,-0.5605584161
H,0,-5.9304586267,1.9314057889,0.0741921471
H,0,-5.2654187834,1.6789245596,-1.5313571402
C,0,-6.2658162887,-0.0643393027,-0.7023622006
H,0,-5.9256678096,-0.6595564354,-1.5568315659
H,0,-7.3151895062,0.1925280317,-0.8670357334
O,0,-4.7851078695,-1.3415167979,0.740658566
C,0,-2.5651400017,-0.691453597,1.1424414311
H,0,-2.3761843962,-1.6691206466,1.571357841
C,0,-1.5294699267,0.2711941815,1.0911498591
C,0,-3.0155529138,1.809787477,0.0093984529
C,0,-6.1410845685,-0.8988838693,0.5561705678
H,0,-6.7345425301,-1.8121752319,0.5111210565
H,0,-6.4363468166,-0.3190721108,1.4402294022
C,0,-1.7624055701,1.5365746289,0.5194841465
H,0,-0.9703893552,2.2783226787,0.4956167019
O,0,-3.3211135971,3.0039551576,-0.5642631483

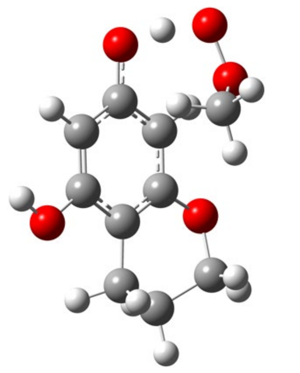

H,0,-2.5442411305,3.5838717259,-0.5640490575
O,0,-0.3441553829,-0.0127879391,1.6210154065
H,0,-0.4743822566,-0.1665250004,2.7297430388

16(2)-TS

Charge = 0 Multiplicity = 2

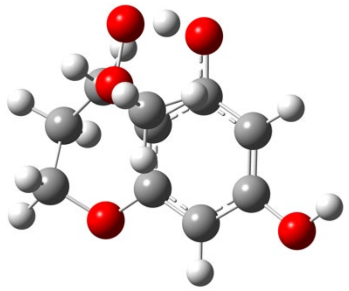

O,0,-0.1895488764,0.7605494619,-1.0244193563
O,0,-0.3740420615,0.1355948779,0.1756571377
C,0,0.8895997082,-0.1382919373,0.7892335723
H,0,1.3208168958,0.7982800739,1.1531145528
H,0,0.6694636517,-0.8116378373,1.6197427988
H,0,1.5556842155,-0.6130624093,0.0654076428
C,0,-2.0031970431,-3.002445578,0.4392857243
C,0,-1.8295882329,-2.1059934197,-0.6304456911
C,0,-2.930057518,-1.1878327903,-1.0763291703
H,0,-2.6794158208,-0.1582425015,-0.7872931545
H,0,-2.9864938696,-1.1979111484,-2.1692535654
C,0,-4.2583661798,-1.5986181694,-0.4438544517
H,0,-4.6636637089,-2.4866636636,-0.9410400417
H,0,-4.9935089112,-0.79529542,-0.535819739
O,0,-3.1586175948,-3.0461282635,1.1684214746
C,0,-1.0150881407,-3.9114367899,0.7874775711
H,0,-1.1731151836,-4.6045611613,1.6071427971
C,0,0.1963247873,-3.9372105511,0.0669044363
C,0,-0.5718683873,-2.1290412054,-1.3069512555
C,0,-4.0347774352,-1.9142357468,1.0208935995
H,0,-4.9544859975,-2.2006216975,1.5312930086
H,0,-3.583997287,-1.0589531232,1.5405775925
C,0,0.4352197296,-3.0507576888,-0.9651426603
H,0,1.3785583566,-3.0429777783,-1.5015212588
O,0,-0.3560189887,-1.2549522986,-2.2838881147
H,0,-0.313866909,-0.2311140485,-1.8299316391
O,0,1.1063942415,-4.8680665481,0.465339408
H,0,1.9053665598,-4.810532638,-0.0804532179

17(1)-TS

Charge = 0 Multiplicity = 2

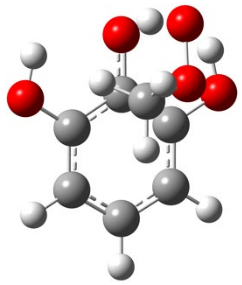

O,0,1.3230942973,-0.6526963541,0.5220183454
O,0,0.33152025,-0.5544779642,-0.3944913455
C,0,-0.7312086645,-1.4627595147,-0.0740831873
H,0,-0.3801406032,-2.4850635326,-0.2337890569
H,0,-1.0331750719,-1.3185639438,0.9666769
H,0,-1.5419170899,-1.2157235454,-0.7605189135
C,0,-2.432275592,2.4579097333,-0.8831831091
C,0,-2.7758854566,1.7840601862,0.2941023311
C,0,-1.7652835956,1.3839895218,1.1544521191
C,0,-0.408854417,1.6568063548,0.8457383979
C,0,-0.0972787374,2.3464693137,-0.3540475395
C,0,-1.1079454079,2.7441967588,-1.2192677375
H,0,-3.2233257047,2.768031021,-1.558357226
H,0,-3.8092676953,1.567617345,0.5407218092
H,0,-0.8549323405,3.263940511,-2.1365007771
O,0,-2.0537992135,0.7120989844,2.2932785164
H,0,-1.2240828217,0.5188095433,2.7637795031
O,0,1.1972509431,2.5885999563,-0.6568067153

H,0,1.7637705008,2.2812747053,0.0714800803
O,0,0.555313997,1.2837299597,1.688472603
H,0,1.0414034235,0.3882539602,1.2797970023

17(2)-TS

Charge = 0 Multiplicity = 2

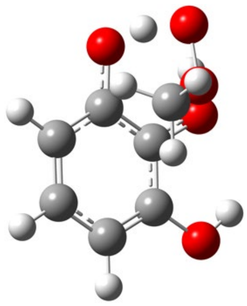

O,0,-1.7394313504,-0.8359175969,-1.1838074914
O,0,-0.5556915695,-0.2375587818,-1.4863547451
C,0,-0.7812988468,1.061510912,-2.0475082516
H,0,-1.4766293242,1.617964315,-1.4155704162
H,0,0.1988978813,1.5408070949,-2.0702647811
H,0,-1.1822141578,0.9462945446,-3.0577474586
C,0,0.8369638726,2.4441795499,1.1935747082
C,0,-0.3831568149,1.8025247362,1.2484363412
C,0,-0.4337340404,0.4016356155,1.1036043738
C,0,0.7713264868,-0.3271161871,0.91683918
C,0,2.0033545694,0.3485502287,0.8502155036
C,0,2.0328261234,1.7228046203,0.9865094081
H,0,0.8887837758,3.5218051647,1.3057923818
H,0,-1.3129631743,2.3422822258,1.3922346507
H,0,2.9882543243,2.2346254518,0.9365011272
O,0,-1.5777102045,-0.2807581215,1.1314149929
H,0,-1.7950688129,-0.6219688836,0.0975112676
O,0,3.1625382844,-0.3478778696,0.6566807882
H,0,2.9743882594,-1.2977403045,0.5981876567
O,0,0.779480522,-1.6638004169,0.7833741584
H,0,-0.1205138037,-2.0163792975,0.8976846056

18(1)-TS

Charge = 0 Multiplicity = 2

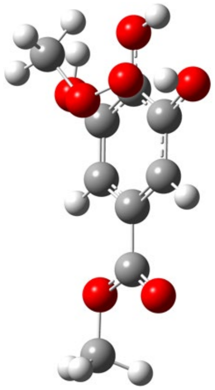

O,0,1.2120721946,-0.3852521876,-0.1646061318
O,0,0.8260875593,0.8624529326,0.2101960878
C,0,1.6696013167,1.8405562915,-0.4080364693
H,0,2.6784433895,1.7498091806,0.001850915
H,0,1.6803780737,1.6835494151,-1.4900687709
H,0,1.2291730886,2.8055652301,-0.153885278
C,0,-2.2165899411,0.2987387439,-0.3331469056
C,0,-1.2826834888,0.4020145513,-1.3837689462
C,0,-1.1453985723,1.6328290844,-2.0788557908
C,0,-1.9226803192,2.7430123304,-1.715612778
C,0,-2.830195554,2.6290301054,-0.6801497484
C,0,-2.9768293892,1.402346768,0.005736094
H,0,-2.3156271598,-0.6434493664,0.1950257987
H,0,-3.4271251925,3.4924953125,-0.409053198
O,0,-1.7874109194,3.930778908,-2.3727426999
H,0,-1.1277832611,3.8478881779,-3.0797127932
O,0,-0.2723651663,1.7666350816,-3.0849650602
H,0,0.1856376679,0.9169887563,-3.2264995295
O,0,-0.5115696768,-0.604965103,-1.767939133
H,0,0.3585767792,-0.6609029808,-1.0307723105
C,0,-3.9521993717,1.2633099789,1.1210296807
O,0,-4.1336483858,0.2277374581,1.738993201
O,0,-4.6139567703,2.3898342509,1.38124384
C,0,-5.5756514149,2.3275082142,2.4481204352
H,0,-6.0046051899,3.3252440621,2.5093056091

H,0,-6.3461936767,1.5917494893,2.2121902295
H,0,-5.0776106197,2.0655173148,3.3830966525

18(2)-TS

Charge = 0 Multiplicity = 2

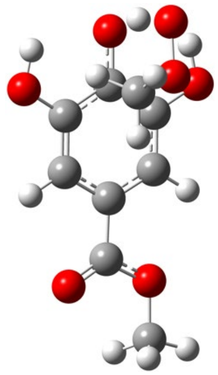

O,0,0.8596759245,0.4609775258,0.6421944102
O,0,-0.0897875785,0.5553194243,-0.3194177159
C,0,-0.0320316806,-0.587888677,-1.1841519354
H,0,-0.0655852588,-1.5013392342,-0.5842497216
H,0,-0.905724208,-0.5057568851,-1.8316735718
H,0,0.8924116178,-0.543106516,-1.7646158791
C,0,-3.5761565297,-1.2178782587,0.2142160816
C,0,-2.4078404294,-1.3378121535,0.9450268986
C,0,-1.8079353711,-0.19534483,1.5360424653
C,0,-2.4200665494,1.0748975421,1.3654159973
C,0,-3.5895854904,1.1998090036,0.6290554273
C,0,-4.151747651,0.0505744441,0.0656990783
H,0,-4.030451817,-2.092634413,-0.2364891104
H,0,-4.0426754981,2.1753512063,0.5019046265
O,0,-1.8409634021,2.1652974909,1.9068310924
H,0,-1.0620200013,1.9004028331,2.4263829724
O,0,-0.6983070793,-0.3251986433,2.2486602462
H,0,0.1599449989,0.0390565514,1.6240354876
O,0,-1.8105917842,-2.5394489713,1.0930134329
H,0,-1.0084165595,-2.4308723581,1.6338619404
C,0,-5.4105159939,0.1340974493,-0.7339056619
O,0,-5.9396438463,-0.8292070761,-1.2604523708
O,0,-5.9001748459,1.3683240117,-0.814339847
C,0,-7.1162537609,1.5174492968,-1.5674227032
H,0,-7.9097309873,0.9222732132,-1.1125651611
H,0,-7.356826156,2.577190401,-1.5211748798
H,0,-6.9545830621,1.2042916225,-2.6001965989

18(3)-TS

Charge = 0 Multiplicity = 2

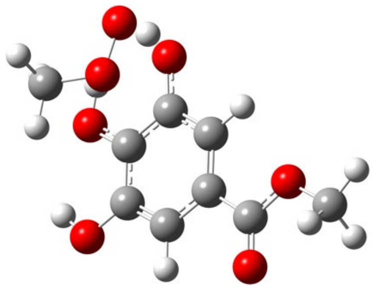

O,0,1.2874195511,-0.3608382779,-0.2310192163
O,0,0.8692477411,0.8651157282,0.1792996724
C,0,1.6795461283,1.88314811,-0.4185683753
H,0,2.6928485277,1.8126563246,-0.0157388703
H,0,1.6889947395,1.7535779736,-1.5041787517
H,0,1.2118863837,2.8279153078,-0.1382210517
C,0,-2.1658042557,0.238851631,-0.3421904649
C,0,-1.243074435,0.388186333,-1.3982662365
C,0,-1.1350552868,1.637112518,-2.0652439292
C,0,-1.9295505765,2.724219019,-1.6692221349
C,0,-2.8256361035,2.5646978831,-0.6310677631
C,0,-2.944554688,1.3205261762,0.0263788201
H,0,-2.2356874204,-0.7206543834,0.157108603
H,0,-3.4422942545,3.4040053917,-0.3272332283
O,0,-1.8221443795,3.9299682002,-2.297609873
H,0,-1.1710874706,3.8762375746,-3.015203835
O,0,-0.2738244784,1.809648286,-3.075342687
H,0,0.1937128988,0.9702312506,-3.2439965999
O,0,-0.4553957689,-0.5940966362,-1.8125066893
H,0,0.4267186038,-0.6421305657,-1.0908528384

C,0,-3.9365063455,1.2224203822,1.130881187
O,0,-4.6377800176,2.1510570558,1.4959274025
O,0,-3.9865002343,0.0125781314,1.685932922
C,0,-4.9286756734,-0.151767576,2.759261745
H,0,-4.8252237567,-1.1864758634,3.0786762925
H,0,-4.6846866758,0.5285239497,3.5768137906
H,0,-5.940916753,0.039161076,2.3990141099

19(1)-TS

Charge = 0 Multiplicity = 2

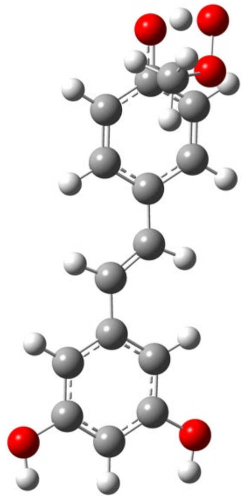

O,0,-1.4133313606,-0.4547263552,2.7396272634
O,0,-0.0898010682,-0.3316861159,3.0268418397
C,0,0.3388605414,-1.4477632939,3.8134813805
H,0,1.4179153799,-1.3318995691,3.9225427491
H,0,-0.1576499671,-1.4121593578,4.7866114039
H,0,0.0946104282,-2.3766711986,3.2911161291
C,0,0.2954959407,-1.2313021307,0.4693722464
C,0,1.2781608266,-2.2487825937,0.5234753026
C,0,2.5997928011,-1.9500628203,0.276041065
C,0,3.0028809776,-0.6234477584,-0.0168621367
C,0,2.0154459857,0.3855203253,-0.0550061505
C,0,0.6861073646,0.0971400534,0.1805916919
H,0,0.9637469606,-3.2619649597,0.7541523078
H,0,3.3344772007,-2.7477752992,0.310719108
H,0,2.3161833086,1.4061017741,-0.2740971579
H,0,-0.074157432,0.8721182492,0.1530695737
O,0,-0.9871056899,-1.5431030935,0.6569916554
H,0,-1.3500847903,-1.0112169988,1.5336143114
C,0,4.3847583435,-0.2466886589,-0.2764406057
H,0,4.533251432,0.8055352164,-0.5106175646
C,0,5.4472752861,-1.0789869591,-0.2436828091
H,0,5.3017608979,-2.1307212067,-0.0056208052
C,0,6.8398195985,-0.6988027519,-0.5061624896
C,0,7.2181334102,0.6142975721,-0.8275487591
C,0,7.8145082013,-1.7029227298,-0.4266514281
C,0,8.5579156506,0.8982772064,-1.0645316729
H,0,6.4953971057,1.4196927189,-0.8969888806
C,0,9.1514558374,-1.3907842391,-0.6665298226
H,0,7.5392360652,-2.7233423057,-0.1788667034
C,0,9.5401107832,-0.0924355668,-0.9880110051
H,0,10.5837498709,0.1461908011,-1.1764227328
O,0,8.8790250314,2.1940171065,-1.3757970376
H,0,9.832036397,2.275198164,-1.5258945774
O,0,10.0618620267,-2.4107883666,-0.5728307872
H,0,10.9559896546,-2.0771838578,-0.7386019017

19(2)-TS

Charge = 0 Multiplicity = 2

O,0,0.2045895028,-0.6234417867,0.2609474652
O,0,-1.0139382695,-0.2948753622,0.7875460281
C,0,-0.8558760136,0.6663936311,1.8365309607
H,0,-0.3057587932,1.5357781231,1.4673069207
H,0,-1.8700783294,0.9413094088,2.1311163502
H,0,-0.3238202681,0.1987202505,2.6688117455
C,0,-8.7207268228,-1.7893615556,0.5539142081

C,0,-8.6468314316,-0.4119837492,0.3241499047
C,0,-7.419852319,0.1768223132,0.0549482918
C,0,-6.2400636376,-0.5875645813,0.0120864069
C,0,-6.3439202347,-1.9676325629,0.2379474456
C,0,-7.568148505,-2.5722630899,0.5087196369
H,0,-9.5556651047,0.1807204142,0.3548424853
H,0,-7.3858202841,1.2464341728,-0.1288912967
H,0,-5.4465129987,-2.5798385228,0.203373632
H,0,-7.6332493284,-3.6424421749,0.684448962
O,0,-9.956717862,-2.319139167,0.8169041864
H,0,-9.8847874005,-3.2743306997,0.9610585163
C,0,-4.9159294393,-0.0139007258,-0.2588016269
H,0,-4.115812679,-0.7474841094,-0.354221558
C,0,-4.6248571559,1.2935475096,-0.3738260285
H,0,-5.4008515612,2.0435155276,-0.2334896104
C,0,-3.2848812485,1.8317233153,-0.6448265847
C,0,-3.0408112892,3.1836941678,-0.4284122491
C,0,-2.2287232543,1.0270692649,-1.1168181108
C,0,-1.7590628504,3.7363888851,-0.6595181604
H,0,-3.8332972282,3.8398150268,-0.0803603594
C,0,-0.9369778261,1.5816463599,-1.3051585412
H,0,-2.377311254,-0.0138708033,-1.37938906
C,0,-0.7026623944,2.9519159104,-1.0814769932
H,0,0.2899112777,3.3654767837,-1.2291832922
O,0,-1.6379226947,5.0695804338,-0.4244466819
H,0,-0.7326718393,5.3656717682,-0.6053996957
O,0,0.0639277258,0.7821427219,-1.6510844412
H,0,0.1960478113,0.0149999017,-0.8202978559

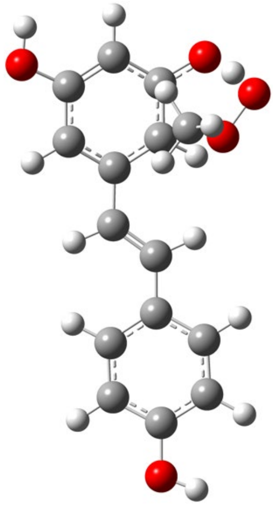

19(3)-TS
Charge = 0 Multiplicity = 2
O,0,0.1509688058,-0.6531736994,0.3320949492
O,0,-0.986212133,-0.1904307187,0.9313752607
C,0,-0.6642192196,0.8300897698,1.8823331623
H,0,-0.0342159906,1.5894619931,1.4135715245
H,0,-1.6225816912,1.2519592524,2.1899061553
H,0,-0.1508496828,0.3745409385,2.7331941219
C,0,-9.7686673936,0.5372283395,0.4732517162
C,0,-8.7118190022,-0.3697802162,0.3462383641
C,0,-7.4243366861,0.0981159475,0.1279256357
C,0,-7.158190126,1.4754515249,0.0261833086
C,0,-8.2360200422,2.3627625739,0.1575912012
C,0,-9.5331971329,1.9078974768,0.3779811309
H,0,-8.9132534204,-1.4333752915,0.4267435607
H,0,-6.6174908773,-0.6230781808,0.0403421169
H,0,-8.0539903466,3.4319125052,0.0857372561
H,0,-10.3586986405,2.6071792991,0.4775973645
O,0,-11.0182872318,0.020391884,0.6943139318
H,0,-11.6633374225,0.7365157351,0.7912245778
C,0,-5.8156587556,2.0244477125,-0.2036302658
H,0,-5.7688041982,3.1129950741,-0.2041720643
C,0,-4.6943063384,1.3145620092,-0.4153722076
H,0,-4.736964025,0.2276870278,-0.4516746863
C,0,-3.3539460846,1.8788726962,-0.6365158904
C,0,-3.0402337828,3.2224758615,-0.4297705269

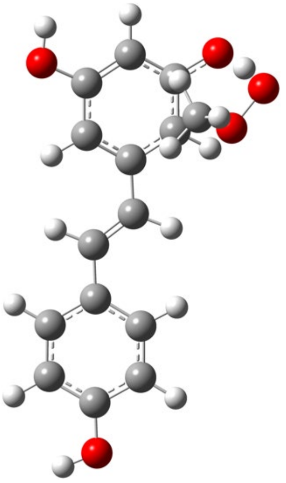

C,0,-2.3333022733,1.0149840026,-1.0665497931
C,0,-1.7354572543,3.7024369353,-0.6772286095
H,0,-3.7798335063,3.9321976838,-0.0733255533
C,0,-1.0186744178,1.4994800668,-1.2888311448
H,0,-2.5355424526,-0.0357989038,-1.2477077792
C,0,-0.7220402135,2.8624029308,-1.1002682585
H,0,0.2878288985,3.2264365785,-1.2612912955
O,0,-1.5384636581,5.0310232786,-0.4557937412
H,0,-0.6157781259,5.2695050425,-0.6343294812
O,0,-0.0598460683,0.6516647877,-1.639035949
H,0,0.1000654895,-0.0780219173,-0.7766920916

20(1)-TS

Charge = 0 Multiplicity = 2

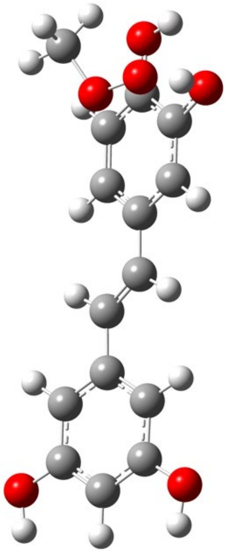

O,0,-0.9946267605,1.8132125487,-0.2930219333
O,0,-0.2539132917,1.3678103804,0.7539070752
C,0,-1.0468650668,1.351503948,1.9436617764
H,0,-1.3087799319,2.3783365719,2.2109680818
H,0,-1.9488886994,0.7572768794,1.7738295601
H,0,-0.4162763117,0.8971060593,2.7090130164
C,0,-0.6513809479,-1.671391539,0.7000950085
C,0,0.3494605319,-2.3279480298,1.4258347656
C,0,1.64162529,-2.3404317946,0.9395987991
C,0,1.9869101898,-1.7012803525,-0.2837248538
C,0,0.9871952126,-1.0480596174,-0.9952452502
C,0,-0.3324060585,-1.014442966,-0.5185167581
H,0,0.0909949338,-2.8232258946,2.3558682781
H,0,2.4030001207,-2.8623546966,1.5093394977
H,0,1.2092406653,-0.5429993281,-1.9314203726
O,0,-1.3232763999,-0.3915119981,-1.1657410958
H,0,-1.2608189039,0.6678665435,-0.9079741608
O,0,-1.9148728824,-1.6627599878,1.1536097406
H,0,-2.4735861786,-1.1843361883,0.5134746283
C,0,3.3503449051,-1.7078180221,-0.8232710119
H,0,3.4504661985,-1.2452341492,-1.8036291958
C,0,6.8374218856,-2.638551096,0.0855339418
C,0,8.1494998698,-2.6602199147,-0.3836154562
C,0,8.4542372622,-2.2810982463,-1.6884922523
C,0,7.4095803018,-1.8813835616,-2.5252774547
C,0,6.0916402288,-1.8501003417,-2.0817408726
C,0,5.8009901232,-2.2265196976,-0.7615423432
H,0,6.6307037153,-2.9392361923,1.1079742552
H,0,9.479108084,-2.3020787887,-2.0496949918
H,0,5.3146774359,-1.5442331416,-2.7740127743
C,0,4.4363964785,-2.2131272936,-0.2122960522
H,0,4.347591477,-2.6472021285,0.7816648375
O,0,7.6454387681,-1.508102285,-3.8240471651
H,0,8.5904688407,-1.5770350482,-4.0248284101
O,0,9.1214804545,-3.0689137352,0.4925495667
H,0,9.9884064598,-3.0513338961,0.0617285759

20(2)-TS

Charge = 0 Multiplicity = 2

O,0,-2.5461276927,-0.1927674666,0.0650143971
O,0,-1.3500441511,-0.2676275227,-0.5652272285

C,0,-0.7602023487,-1.5595591362,-0.3703109091
H,0,-1.3210148571,-2.2949895145,-0.9527104874
H,0,0.2633222514,-1.4705996555,-0.7377017913
H,0,-0.777517842,-1.8122010533,0.692003839
C,0,-0.3154970411,1.0452897291,1.5210219291
C,0,0.6896749875,0.2492638246,2.1042706516
C,0,1.9941364465,0.3424143872,1.6733278996
C,0,2.3492521266,1.2381452264,0.6288052554
C,0,1.3554547613,2.0305060343,0.0446474914
C,0,0.0389123565,1.9480239759,0.4839481901
H,0,0.4038417211,-0.4378641142,2.8946333085
H,0,2.7498551626,-0.2808497279,2.1382643471
H,0,1.6006982518,2.722862693,-0.7552848343
O,0,-0.9034233898,2.7248486501,-0.0929229228
H,0,-1.7574216147,2.5850574693,0.35030601
O,0,-1.5883567646,0.9912148823,1.9331459486
H,0,-2.1726321978,0.4867366886,1.1825642985
C,0,3.7179877837,1.378041187,0.1333283458
H,0,3.8321024931,2.0834574636,-0.6873146329
C,0,7.179925313,0.1629446608,0.7871196704
C,0,8.5043130268,0.2786374033,0.3679992281
C,0,8.8501319462,1.0929901537,-0.7071591402
C,0,7.8377237026,1.8017067462,-1.3580880071
C,0,6.5093161174,1.7054996323,-0.9598700691
C,0,6.1748017363,0.8792471617,0.1240573862
H,0,6.9390608117,-0.4792903662,1.6285620125
H,0,9.8842618983,1.1795133056,-1.0308563952
H,0,5.7611146691,2.2753267029,-1.4996568751
C,0,4.7969279711,0.7299841011,0.6128678519
H,0,4.6859141289,0.0321285831,1.4402959012
O,0,8.1195778733,2.6232164289,-2.4190820573
H,0,9.0711281197,2.6174233117,-2.5983482975
O,0,9.4478259083,-0.4388247679,1.0558295213
H,0,10.3271033349,-0.2691180777,0.6869221642

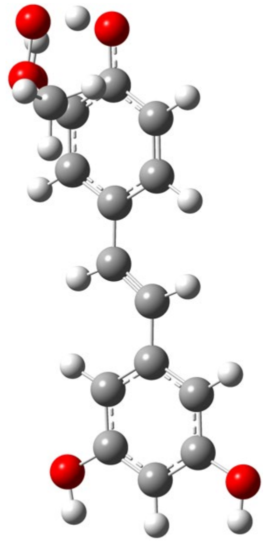

20(3)-TS

Charge = 0 Multiplicity = 2
O,0,-0.4705275057,0.355954797,-0.0357601494
O,0,-1.5063921899,-0.2882524491,0.5827548889
C,0,-1.0888016504,-0.7858830878,1.8583201402
H,0,-0.2142916645,-1.4311679568,1.740989887
H,0,-0.8583353463,0.0602526481,2.5105345013
H,0,-1.9416752784,-1.3489617795,2.2410261807
C,0,-9.0626182012,-2.1061452835,0.2582916124
C,0,-8.4430152517,-3.3460303909,0.3803267976
C,0,-7.0649520126,-3.460673281,0.2266383599
C,0,-6.2802099704,-2.3306692721,-0.0480855117
C,0,-6.9181785473,-1.0863698492,-0.1773805132
C,0,-8.2924808958,-0.9723342897,-0.0253860632
H,0,-9.0561780865,-4.2166085503,0.5914679775
H,0,-6.6068429942,-4.4403274393,0.3149208738
H,0,-6.3359972496,-0.1941799195,-0.397039787
O,0,-8.980802106,0.2095298313,-0.1356087438
H,0,-8.3705194642,0.9382163189,-0.3225484184
O,0,-10.4200919248,-2.0115919468,0.4110472429

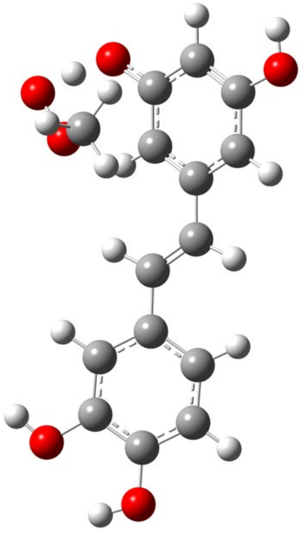

H,0,-10.6939998288,-1.0885448163,0.2927601893
C,0,-4.8205406208,-2.3787502195,-0.2068108285
H,0,-4.3686539508,-1.4440120146,-0.5373567098
C,0,-1.8273659407,-4.4699202882,0.4526958127
C,0,-0.4168576812,-4.4910668414,0.3414839383
C,0,0.2695211809,-3.4882110224,-0.3166990687
C,0,-0.4731589576,-2.446436036,-0.9040401579
C,0,-1.8894731684,-2.441072745,-0.8374890322
C,0,-2.5716438575,-3.4464415425,-0.1234413582
H,0,-2.3172530526,-5.2712804561,0.9980809421
H,0,1.3534061284,-3.4836166249,-0.3733967166
H,0,-2.4201219522,-1.663867837,-1.374800543
C,0,-4.0318673733,-3.439112062,0.0389960265
H,0,-4.4597106796,-4.3645547592,0.4194958308
O,0,0.1474629165,-1.4264933974,-1.483478649
H,0,-0.1209903667,-0.4862309164,-0.8997006911
O,0,0.2093985737,-5.5401125424,0.9363348085
H,0,1.1698229703,-5.4679969783,0.8245179312

20(4)-TS

Charge = 0 Multiplicity = 2

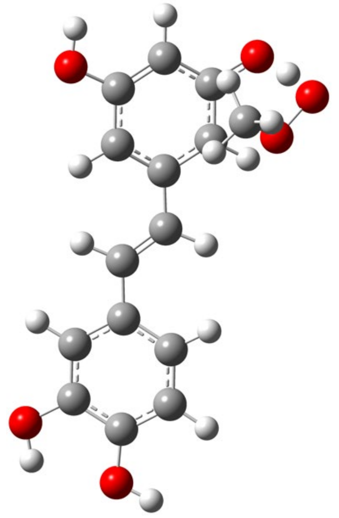

O,0,0.1973436059,-0.7883460522,-0.3245320552
O,0,-0.9189094915,-0.5281246212,0.4184363115
C,0,-0.5629426866,0.1799151924,1.6106180425
H,0,-0.029773313,-0.4983910012,2.282092353
H,0,0.0609113269,1.0403593739,1.3579812339
H,0,-1.5086996903,0.4981481139,2.0525355289
C,0,-9.7625330072,-0.1468523389,-0.2296198212
C,0,-8.631513085,-0.9484232916,-0.352666298
C,0,-7.3636243988,-0.3751239693,-0.3569807432
C,0,-7.2077611836,1.0144440035,-0.2444231989
C,0,-8.3564713066,1.8110657802,-0.1137949168
C,0,-9.6208756979,1.2404447511,-0.1091120594
H,0,-8.7553621761,-2.0245062626,-0.4376664823
H,0,-6.4969507345,-1.0223386432,-0.4406470253
H,0,-8.2699789004,2.8901579859,-0.0186184334
O,0,-10.7243616825,2.0459006182,0.0136121078
H,0,-11.5241792121,1.4974694337,-0.0054073379
O,0,-11.0469326258,-0.6241284158,-0.2110483932
H,0,-11.050535884,-1.5900511429,-0.286490614
C,0,-5.8968134407,1.6790655576,-0.2582022234
H,0,-5.9209191947,2.7422738965,-0.0219291008
C,0,-2.3352728229,1.1228570692,-1.1785976019
C,0,-1.052977147,1.7290161133,-1.2139792472
C,0,-0.8506976805,2.9981401907,-0.6384029213
C,0,-1.9250034222,3.6257412929,-0.036028057
C,0,-3.1984271081,3.0188715645,0.0229162089
C,0,-3.4163946493,1.770077541,-0.5598826449
H,0,-2.4648715045,0.1547743286,-1.6519106304
H,0,0.1349218096,3.4524308108,-0.658221767
H,0,-3.9919673378,3.5514812028,0.5373024229
C,0,-4.7234928756,1.0951758864,-0.5534940601
H,0,-4.6948819201,0.0461666692,-0.8418381244
O,0,-1.8194412514,4.8503260321,0.5488510266
H,0,-0.9129277346,5.1836651526,0.4651329298

O,0,-0.0367410844,1.0811532103,-1.7680107272
H,0,0.1335715073,0.1155069675,-1.1900686815

Cartesian coordinates of the optimized transition states between a phenol and •OOCH3 studied at the M06-2X(SMD)/6-31++G(d,p) level of theory in PE.

1-TS
Charge = 0 Multiplicity = 2
O,0,-0.1906686113,-1.5931565337,3.7232851786
O,0,-1.400244632,-2.1507537436,3.4607718483
C,0,-2.4395202152,-1.3288108314,3.9948912503
H,0,-3.3711522233,-1.7267601358,3.5877511137
H,0,-2.4340252466,-1.4066811524,5.0862499454
H,0,-2.2947291518,-0.2904971503,3.6896882205
C,0,-3.2030048045,-0.5389976989,0.3344392039
C,0,-3.4584227068,-1.7822635019,-0.272401448
C,0,-2.4607149572,-2.767606126,-0.3205714071
C,0,-1.1996786571,-2.5655006673,0.2414037657
C,0,-0.9557209017,-1.3120358277,0.8842260759
C,0,-1.9670020974,-0.318623415,0.8934196933
H,0,-3.9823092534,0.2160805922,0.3459296001
H,0,-2.6778481521,-3.7059182579,-0.8116954433
H,0,-1.7315243478,0.62885544,1.3698037813
O,0,0.2136904225,-1.016344292,1.4427466409
H,0,0.164410185,-1.230360029,2.5182315253
O,0,-4.6909253152,-1.9357579822,-0.7944292918
C,0,-5.0153369579,-3.1682574489,-1.4267109577
H,0,-6.0471503443,-3.0672190792,-1.7613014937
H,0,-4.3664843198,-3.3486722691,-2.2902506813
H,0,-4.9388715755,-4.0024514079,-0.7214206232
C,0,-0.1054346902,-3.6315982683,0.1330979621
C,0,-0.5787455467,-4.8611919779,-0.6531296318
H,0,-1.422386489,-5.3616094983,-0.165332953
H,0,-0.8667645286,-4.607826614,-1.6793597523
H,0,0.2439543322,-5.5808978534,-0.7086303417
C,0,1.109782086,-3.0427756053,-0.6093779166
H,0,1.5389733117,-2.1985283723,-0.0675265977
H,0,1.8787847332,-3.8159507602,-0.7180148462
H,0,0.8238425208,-2.7064929295,-1.6124781848
C,0,0.3174043503,-4.1100306134,1.5335722391
H,0,1.0764612921,-4.8940403252,1.4305846346
H,0,0.7382779753,-3.3039636163,2.135228023
H,0,-0.5367010537,-4.529863248,2.0749973483

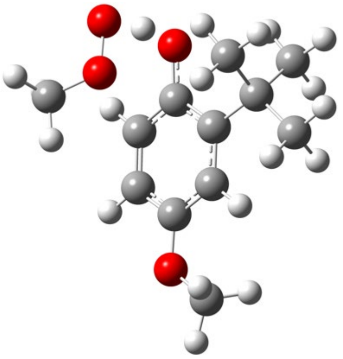

2-TS
Charge = 0 Multiplicity = 2
O,0,-1.110594457,-2.6057061535,0.4492527537
O,0,-0.7084139864,-1.8336069397,-0.5949531742
C,0,-1.8539350454,-1.3855649326,-1.3188063483
H,0,-2.3431820763,-2.2408911082,-1.7941039546
H,0,-2.5495141941,-0.8835904458,-0.6401954066
H,0,-1.4771923967,-0.6898008397,-2.0711278395
C,0,-0.1727153703,2.1939891646,0.1493594059
C,0,1.2105186967,1.9950086679,-0.0092706601

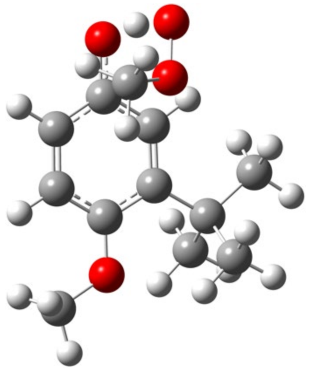

C,0,1.8541806615,0.8238217557,0.4932717551
C,0,1.050356991,-0.0921725207,1.1486050436
C,0,-0.3454183421,0.0907193412,1.31963623
C,0,-0.9481098219,1.2496291742,0.7978532908
H,0,-0.6440016456,3.08804616,-0.2397638329
H,0,-2.016921411,1.3932714319,0.9232346041
O,0,-1.0681190051,-0.7964492067,1.9893404001
H,0,-1.0856843375,-1.7373085064,1.420051212
O,0,1.9896324631,2.8948816732,-0.6419314793
C,0,1.4079696567,4.094907246,-1.1361745675
H,0,0.9659030917,4.6801658117,-0.3233477636
H,0,2.2276860439,4.6551986116,-1.5849065281
H,0,0.6517266025,3.880889852,-1.8981932484
H,0,1.4691599327,-1.0048755896,1.5570023752
C,0,3.3603334308,0.5884928179,0.3109807325
C,0,3.7973796033,-0.7400996294,0.9419556613
H,0,3.6122004047,-0.7621778422,2.0212502833
H,0,3.2923939271,-1.5970440509,0.4837430173
H,0,4.8735175262,-0.8660614184,0.7868168623
C,0,3.7069466787,0.5211861064,-1.1881958237
H,0,3.4851931445,1.4575842586,-1.7026888694
H,0,4.7758319559,0.310124805,-1.3058961105
H,0,3.1475071132,-0.285472308,-1.6748990804
C,0,4.1656561938,1.709481127,0.994351311
H,0,5.2367197398,1.5009187437,0.8932218555
H,0,3.9648048995,2.6870026919,0.553234575
H,0,3.9302002921,1.7546554009,2.0634912686

3-TS

Charge = 0 Multiplicity = 2

O,0,0.4062221775,1.0871665137,0.8642850604
O,0,-0.6766623851,0.4646848888,1.3993898285
C,0,-0.339988261,-0.8868503719,1.7117996539
H,0,0.0756766206,-1.3801381187,0.8287280067
H,0,0.3871928835,-0.9018788369,2.5291141404
H,0,-1.2736122946,-1.3634191324,2.0173121346
C,0,-4.2001297216,-0.4992645861,-0.8810834335
C,0,-3.8540041164,0.852391959,-0.6670564692
C,0,-1.5334685227,0.2859600634,-1.1992964046
O,0,-0.2731866919,0.6597725593,-1.3772279307
H,0,0.1395794098,0.9822042567,-0.4154661573
C,0,-4.9155109258,1.8558424295,-0.2868931872
H,0,-4.8348918383,2.0913237491,0.7813513271
H,0,-4.7432822471,2.7914063116,-0.8274955143
C,0,-6.3018490017,1.3027040292,-0.6049141478
H,0,-6.4655819449,1.3041028748,-1.6893370541
H,0,-7.082147137,1.9210048011,-0.1507163289
C,0,-6.4523469998,-0.1343513528,-0.1047283546
C,0,-7.7882683002,-0.7250510011,-0.5211659844
H,0,-7.8436010928,-1.7797811502,-0.2362434578
H,0,-8.6022996758,-0.1877878547,-0.025725496
H,0,-7.9234227703,-0.6462308394,-1.6038583924
C,0,-6.2434796313,-0.2562998375,1.4026545857
H,0,-6.9462625568,0.3942646414,1.9326930186
H,0,-6.4151669742,-1.2887801716,1.7203784032

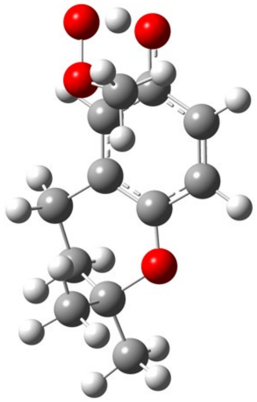

H,0,-5.2268839469,0.0255697387,1.6929194762
O,0,-5.4579637872,-0.9687208937,-0.7596662208
C,0,-3.225562736,-1.4423233028,-1.2676975716
H,0,-3.5398558932,-2.4680175749,-1.4333997414
C,0,-1.9103503657,-1.0615337863,-1.4166604591
H,0,-1.1462081394,-1.7766419231,-1.7056926113
C,0,-2.5274917749,1.2201327102,-0.8321202663
H,0,-2.2301498289,2.2549423173,-0.6771194321

4-TS

Charge = 0 Multiplicity = 2

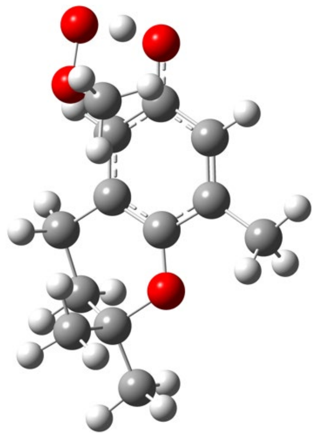

O,0,-2.1164011496,-0.8593852895,1.6146454136
O,0,-0.8513091863,-0.5367809446,1.9922612457
C,0,-0.8063337329,0.8331773858,2.3900474675
H,0,-1.2353506074,1.4634885185,1.6063699743
H,0,-1.3629405108,0.9605876136,3.3235176384
H,0,0.250587523,1.0658740885,2.5373039577
C,0,2.3523627879,0.2138891778,-0.8204348656
C,0,1.8896222107,-1.0754916519,-0.4954776457
C,0,-0.3966671627,-0.253903067,-0.7256290531
O,0,-1.7064043546,-0.4678856256,-0.7045462487
H,0,-2.0110457601,-0.75545824,0.3014774958
C,0,2.8723475852,-2.1875129456,-0.2166531624
H,0,2.9188320199,-2.3769515332,0.8628264522
H,0,2.5173141533,-3.1121296598,-0.6817180275
C,0,4.2537799782,-1.8187981853,-0.7489317168
H,0,4.2600248296,-1.8729392284,-1.8443442121
H,0,5.0137217884,-2.5131292218,-0.3776108209
C,0,4.6371325929,-0.3959814941,-0.3426567101
C,0,5.9599674789,0.0177742524,-0.964235721
H,0,6.1750965381,1.0665327475,-0.7393029029
H,0,6.7690089458,-0.5977196074,-0.5599168646
H,0,5.9312927978,-0.1098716029,-2.0503307197
C,0,4.6583473332,-0.2039416519,1.1720017251
H,0,5.3491702708,-0.9179811885,1.6313375652
H,0,4.9909179124,0.8095036703,1.4145968134
H,0,3.6669861851,-0.3524463143,1.6106334285
O,0,3.6625806977,0.5300610444,-0.8940090331
C,0,1.456329046,1.2747977621,-1.1223982357
C,0,0.1000500877,1.028199639,-1.0597808001
H,0,-0.6143022575,1.8191175357,-1.2724329111
C,0,0.5192216184,-1.2921171209,-0.4547687917
H,0,0.1314944134,-2.2779801715,-0.2094648959
C,0,2.0097885061,2.6227405162,-1.4884831243
H,0,2.6441583165,3.0166590703,-0.6881489324
H,0,2.6330695339,2.5593528959,-2.3862109982
H,0,1.199411171,3.3304548965,-1.6754731742

5-TS

Charge = 0 Multiplicity = 2

O,0,-1.5480242842,-0.6996313208,2.7085737528
O,0,-0.192506323,-0.6481565837,2.6508504923
C,0,0.2524273995,0.6835631444,2.9109547426
H,0,-0.2901507775,1.3890759432,2.2767188794
H,0,0.090400363,0.9214224672,3.9667013337

H,0,1.3188940219,0.6927353589,2.6741569584
C,0,2.1021329692,-0.2030860089,-0.9356546069
C,0,1.6630198846,-1.4404013623,-0.4379892389
C,0,-0.5315218224,-0.4549266696,-0.0705056151
O,0,-1.7995864835,-0.5359616395,0.3261435831
H,0,-1.8352298907,-0.6801864438,1.3953935576
C,0,2.6135200434,-2.613734806,-0.3937458911
H,0,2.9053535219,-2.8166502416,0.6454296569
H,0,2.1021486486,-3.5147054079,-0.7476062592
C,0,3.8463466447,-2.3493792806,-1.2529326483
H,0,3.5860240059,-2.4360276963,-2.3147109716
H,0,4.6309176561,-3.0829521178,-1.0431814739
C,0,4.3947693036,-0.9442563535,-1.0167435836
C,0,5.5490247282,-0.6356831804,-1.9548820778
H,0,5.8685235823,0.40398672,-1.836788572
H,0,6.3974104365,-1.2881041465,-1.727872919
H,0,5.252124322,-0.7955785347,-2.9956020514
C,0,4.7927010331,-0.7067351844,0.4383505895
H,0,5.5399960428,-1.4439449195,0.7484920542
H,0,5.2218097578,0.2934188542,0.5492940413
H,0,3.9320176534,-0.7857593273,1.1094096859
O,0,3.3657027587,0.017504228,-1.3638583068
C,0,1.2319321602,0.91460538,-1.0454264931
C,0,-0.0646947746,0.7679856125,-0.6042270702
H,0,-0.7620786761,1.6000434583,-0.6541976007
C,0,0.3415674466,-1.5719736105,0.0076252948
C,0,-0.1472455046,-2.8801559317,0.5577264835
H,0,-0.1947960193,-3.639484422,-0.2322159453
H,0,0.5293526862,-3.2546632528,1.3324869893
H,0,-1.1424731592,-2.7805252781,0.9901758572
C,0,1.7489043652,2.2048175707,-1.6152711231
H,0,2.5968907971,2.579553797,-1.033181693
H,0,2.1043377209,2.0669566999,-2.6415166144
H,0,0.96218775,2.9623944848,-1.6176632002

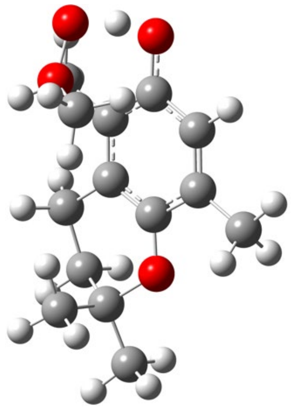

6-TS

Charge = 0 Multiplicity = 2

O,0,-2.8594620149,-0.2503727816,1.0784675698
O,0,-2.1676904386,0.1054477332,-0.0348212003
C,0,-1.7794781132,-1.0661284264,-0.7519186359
H,0,-1.262276797,-1.7577942872,-0.0814784796
H,0,-2.6671738674,-1.5448157226,-1.1763445958
H,0,-1.1113388868,-0.7226868221,-1.5445861986
C,0,1.9048177886,1.5197425977,-0.0223159272
C,0,2.0345510929,0.1765751637,0.4039573905
C,0,-0.0393652592,0.4390314687,1.6545751916
O,0,-0.9275138339,-0.0989283975,2.4856195459
H,0,-1.8915760687,-0.145013785,1.9975385677
C,0,3.2054426545,-0.6615890113,-0.0497828659
H,0,3.9298987098,-0.7558216274,0.7685973805
H,0,2.8610086647,-1.6745895946,-0.2795126244
C,0,3.8705896977,-0.0322083645,-1.2691255666
H,0,3.2331580332,-0.1590967777,-2.1527059296
H,0,4.8318074393,-0.5104669943,-1.481139585
C,0,4.0918646751,1.4645995743,-1.0554022005

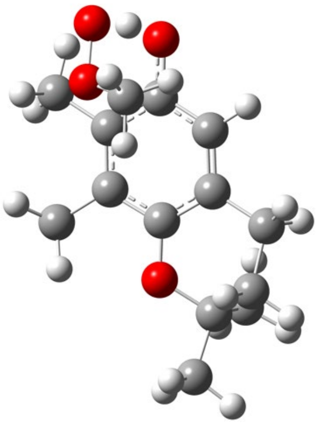

C,0,4.6490309113,2.1225818889,-2.3064680084
H,0,4.715949408,3.2058571438,-2.1693567768
H,0,5.6509695561,1.7360052934,-2.5151825977
H,0,4.0068805059,1.9167139624,-3.1679667477
C,0,4.9717670607,1.7610183729,0.1569059028
H,0,5.9364368144,1.2538792,0.0544664657
H,0,5.1493364523,2.8375854061,0.2338117782
H,0,4.5028168064,1.4264304408,1.0868693951
O,0,2.8072774503,2.1053294117,-0.8426589075
C,0,0.8037562371,2.3185391303,0.3606326244
C,0,-0.1589860493,1.7904568107,1.2183404244
C,0,1.0517284689,-0.341432196,1.2264359851
H,0,1.1067009216,-1.3740069612,1.5631861115
C,0,0.6882277432,3.7316307118,-0.1512047883
H,0,0.9933399109,4.4546534684,0.615006934
H,0,1.3215415524,3.8864679724,-1.0245807821
H,0,-0.3446221203,3.9612842768,-0.4266302207
C,0,-1.3296896645,2.6130461359,1.6731457127
H,0,-1.783573138,2.1827009878,2.5671271705
H,0,-1.0277342077,3.6400261579,1.8959618627
H,0,-2.0998581015,2.652058448,0.893166625

7-TS

Charge = 0 Multiplicity = 2

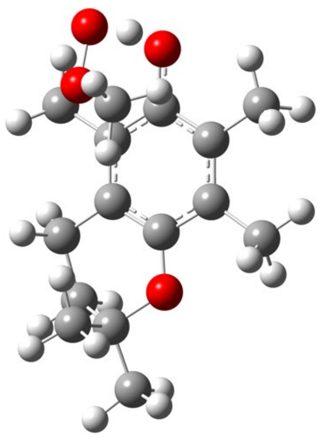

O,0,-1.6028092888,-1.2207740213,2.466126816
O,0,-0.2524489807,-1.2770396254,2.3671003829
C,0,0.3365595318,-0.2897473512,3.214876066
H,0,0.1978115173,-0.5800336405,4.2606908392
H,0,1.3981869929,-0.2706458039,2.9582932789
H,0,-0.1251957816,0.6840637551,3.0299426755
C,0,2.0989128954,0.5248083946,-0.7016426212
C,0,1.4631312941,-0.7138836355,-0.8836397753
C,0,-0.5381075419,0.2504919399,0.1113393462
O,0,-1.8140328399,0.1341268576,0.4854278955
H,0,-1.8823445485,-0.5081774286,1.3390982132
C,0,2.2059570979,-1.86190087,-1.5255438274
H,0,2.4516353777,-2.6180549846,-0.7678128807
H,0,1.5569486921,-2.359532955,-2.2534344549
C,0,3.4744007217,-1.3695461317,-2.2154469938
H,0,3.2157150525,-0.8466900928,-3.1441952468
H,0,4.1260288322,-2.2089135126,-2.4775957094
C,0,4.240846273,-0.3948711961,-1.324719388
C,0,5.4450470083,0.1854016358,-2.0467351746
H,0,5.9289688237,0.9474446594,-1.4286024965
H,0,6.1710958543,-0.6059633891,-2.2554041026
H,0,5.1422584292,0.6422064321,-2.9935903221
C,0,4.6504529263,-1.018396818,0.0081305056
H,0,5.2647777945,-1.9074531333,-0.1663329238
H,0,5.233500248,-0.3002015176,0.5920930438
H,0,3.7785774861,-1.3133115618,0.5999380973
O,0,3.3898571153,0.745289688,-1.0468751061
C,0,1.4284441924,1.6477521473,-0.1561414617
C,0,0.1144512385,1.5066228033,0.2688185075
C,0,0.1384268309,-0.8558670895,-0.465877856
C,0,-0.5652688647,-2.173618137,-0.6225959078

H,0,-0.752712938,-2.3908227136,-1.6812721279
H,0,0.0460826639,-2.9899637166,-0.2248858037
H,0,-1.5231952918,-2.1782486483,-0.1039192905
C,0,2.1867645937,2.9432128473,-0.0389449209
H,0,2.7367348273,3.1531510822,-0.9600739361
H,0,1.519341352,3.7813785727,0.1628684877
H,0,2.9276964632,2.893464809,0.7678448962
C,0,-0.6296582094,2.6469700105,0.9127095933
H,0,-0.0441053882,3.0903251798,1.7246324428
H,0,-0.8379673344,3.4436784423,0.1892426491
H,0,-1.5825651045,2.3025987247,1.3143185924

8-TS

Charge = 0 Multiplicity = 2
O,0,0.2425296179,1.5787855546,-0.5939657007
O,0,0.1515838269,0.238180464,-0.4253871401
C,0,0.8299719643,-0.1426650899,0.7719171422
H,0,0.463580764,0.4543877694,1.6114250901
H,0,0.6031442512,-1.2002982524,0.9201815143
H,0,1.9060996457,0.0079222499,0.6435735816
C,0,-2.3843593799,0.7317718183,0.5059289829
C,0,-2.5353018495,0.1055120125,1.7619898158
C,0,-3.0826561822,-1.1578022072,1.8612425079
C,0,-3.4920412166,-1.8553370596,0.700876965
C,0,-3.3337717832,-1.2351339725,-0.5585241636
C,0,-2.7968964275,0.0325930899,-0.6516050494
H,0,-2.2201684697,0.6454566537,2.6501831318
H,0,-3.204575401,-1.6298829354,2.8324476958
H,0,-3.6409018928,-1.7681598546,-1.4541466484
H,0,-2.6752981457,0.5112001702,-1.6190534902
O,0,-1.9013010428,1.9730215715,0.4346547289
H,0,-0.9804076889,1.9610210787,-0.0994629051
N,0,-3.9927753752,-3.125043652,0.7943973546
H,0,-4.2943540546,-3.464884048,1.6966281177
H,0,-4.4683737004,-3.5206993712,-0.004335191

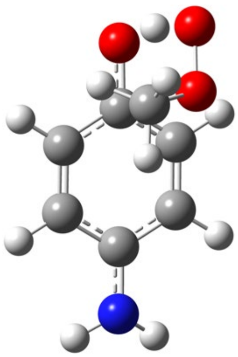

9-TS

Charge = 0 Multiplicity = 2
O,0,-2.1938763863,0.170975032,-1.4883541376
O,0,-1.1922599568,0.8676870524,-0.9104081271
C,0,-0.6658220682,1.8168709622,-1.8386687128
H,0,-0.3968721583,1.3111848898,-2.7700317528
H,0,-1.4154656779,2.5901400445,-2.0313004969
H,0,0.2171151635,2.2450551283,-1.3602879741
C,0,0.394056126,-1.323811061,-1.4178062976
C,0,1.5669371747,-0.8577098137,-2.0417186589
C,0,2.7093249353,-0.5928541065,-1.3112817051
C,0,2.7304316764,-0.7718497889,0.09931093
C,0,1.541975741,-1.2395084957,0.7235281034
C,0,0.4109856665,-1.5099242343,-0.0199728518
H,0,1.5601811141,-0.715879852,-3.1188037024
H,0,3.5944335459,-0.2455737665,-1.830643338
H,0,1.5106817132,-1.3878864769,1.7959684497
H,0,-0.4919234288,-1.8638307961,0.4698029216
N,0,3.851830912,-0.5026453631,0.8272616863

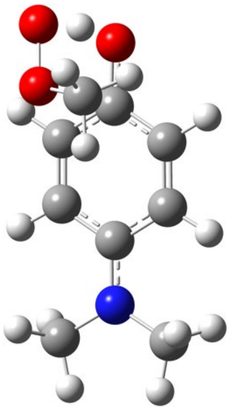

O,0,-0.684900071,-1.6219862618,-2.153690693
H,0,-1.4709704013,-0.9847346855,-1.8864018296
C,0,5.0454519974,-0.0010722328,0.170207062
H,0,5.8226811459,0.1510135002,0.9175139019
H,0,5.4190524442,-0.7136422008,-0.5747709833
H,0,4.8544850741,0.9564159533,-0.330115521
C,0,3.8455469618,-0.6794610116,2.2685885392
H,0,4.8285133683,-0.4216899189,2.6602524407
H,0,3.1005851778,-0.0321378248,2.7475265678
H,0,3.6285680305,-1.7192850119,2.5402083094

10-TS

Charge = 0 Multiplicity = 2

O,0,1.0199975045,-0.0224494331,-0.4493560554
O,0,0.2431814084,-0.8477267484,0.275096367
C,0,-0.0698805335,-0.2463134998,1.533803937
H,0,-0.827260788,-0.8858187612,1.9915774981
H,0,0.8326348283,-0.2124017013,2.1512807033
H,0,-0.4558762043,0.7638397904,1.3727170415
C,0,-1.984635225,-1.2740429645,-1.6252020985
C,0,-3.0053572979,-2.0957305289,-1.1568484409
C,0,-3.9659287867,-1.573568111,-0.2507974995
C,0,-3.9061201427,-0.2220913865,0.1731775361
C,0,-2.8652310623,0.5888087936,-0.2720750129
C,0,-1.9063895266,0.0693126712,-1.1718626341
C,0,-2.7506047368,2.0135863149,0.2010666113
H,0,-3.530748149,2.642782442,-0.2447911259
H,0,-2.8671383975,2.0791482169,1.2882267196
H,0,-1.7850228766,2.435791321,-0.0780146569
C,0,-3.1267505709,-3.5393347635,-1.57326068
H,0,-3.0342254542,-4.2136864147,-0.7112734477
H,0,-4.0995088367,-3.7383071842,-2.0405555925
H,0,-2.3536451237,-3.8252024392,-2.2852639282
C,0,-0.9410301834,-1.7848233127,-2.5841987944
H,0,-0.295920292,-2.528438375,-2.1030503419
H,0,-1.4023613696,-2.2583893397,-3.4560905389
H,0,-0.3081790347,-0.9726098219,-2.9408602872
O,0,-0.942005609,0.8897820065,-1.6333698048
H,0,-0.0216903972,0.5718912687,-1.2901417597
C,0,-4.9622251885,0.3289071908,1.1105375768
H,0,-4.5490841032,0.4025901648,2.1268883089
H,0,-5.2204143758,1.3501182705,0.8119185729
C,0,-6.2210715104,-0.5364096223,1.136744968
H,0,-6.7747277324,-0.4222265371,0.197563331
H,0,-6.8786380409,-0.2278263911,1.9542880204
C,0,-5.8343702116,-1.9976340702,1.302165031
H,0,-6.7148504712,-2.644774042,1.2985617917
H,0,-5.3144291573,-2.1355340585,2.2634980817
N,0,-4.9841014575,-2.3890144727,0.1896630077
H,0,-4.8718756442,-3.3815560617,0.0425452653

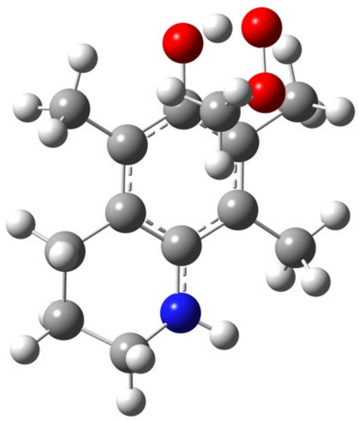

11-TS

Charge = 0 Multiplicity = 2

O,0,-2.0749888462,-0.3654343082,-1.7507429553
O,0,-0.7604782739,-0.2835418412,-2.0165006938

C,0,-0.2583033698,-1.565688747,-2.400282981
H,0,0.8261843287,-1.4564471832,-2.4608958398
H,0,-0.5340834092,-2.3076104593,-1.6464386401
H,0,-0.6759613595,-1.8401312855,-3.3735651361
C,0,3.0126856909,-2.112280969,1.3846666379
H,0,2.5460163155,-2.9667371715,1.8835930141
H,0,3.4341821731,-2.4807620645,0.4389739541
C,0,4.1354000477,-1.5195072729,2.2314909262
H,0,4.922599183,-2.2553787299,2.4175667673
H,0,3.7367246658,-1.2006009919,3.2016405407
C,0,4.7355003491,-0.3250778181,1.5079252344
H,0,5.3011537108,-0.6623639625,0.6254847999
H,0,5.4396535902,0.2016466448,2.1630903002
C,0,4.2209514346,1.9736382737,0.8092838854
H,0,4.4129317285,2.5006729159,1.7566011445
H,0,5.1848361185,1.8627061215,0.2979364775
C,0,3.2572967734,2.7766128254,-0.0494409211
H,0,3.2185996042,2.3496081942,-1.0584953734
H,0,3.6262434351,3.8026067445,-0.1336545288
C,0,1.8665034444,2.7378243752,0.5777039135
H,0,1.1430721082,3.2951127351,-0.0245386131
H,0,1.9049788267,3.2186836554,1.5648967011
N,0,3.7178970705,0.6341437862,1.0939174033
O,0,-1.636111532,-0.7269590696,0.661937023
H,0,-1.9991015883,-0.5051809194,-0.2711636054
C,0,0.6320724786,-1.3702304455,0.990071028
H,0,0.3073055195,-2.4024400083,1.1000688639
C,0,0.0859067563,0.94936568,0.5923931679
H,0,-0.6585572269,1.7138688614,0.3814183298
C,0,1.41633295,1.3054576085,0.7191055996
C,0,2.3922048942,0.3013941152,0.9758017141
C,0,1.9747979226,-1.0538279137,1.1075827361
C,0,-0.3340380543,-0.3860357858,0.7274142853

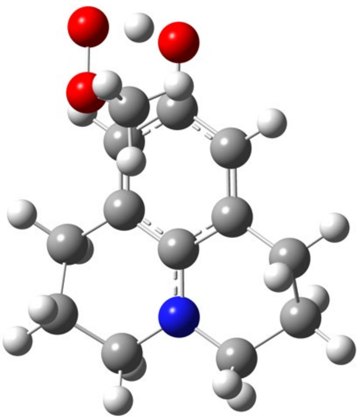

12-TS

Charge = 0 Multiplicity = 2

O,0,-0.8581938065,1.1390672184,2.1651461515
O,0,0.3463698901,0.7211137834,2.6416626719
C,0,0.1371652915,-0.1870397716,3.7225937566
H,0,-0.5053884969,-1.0107831005,3.3981656167
H,0,1.1279526104,-0.5544591985,3.9967700476
H,0,-0.3244212346,0.3427887587,4.5609565029
C,0,0.5264651873,-2.3069266915,0.7765380108
C,0,0.2813260924,-0.9560051149,0.4298810691
C,0,1.3758137631,-0.1414359622,0.033023021
C,0,2.6501031432,-0.6626154492,-0.0283360924
C,0,2.8981438176,-2.0158728176,0.2962674606
C,0,1.8048130111,-2.8185488182,0.6988631493
H,0,-0.3130211912,-2.9222530504,1.0855761687
H,0,1.1835727032,0.8981144359,-0.2152681884
H,0,3.4681145432,-0.0161988914,-0.3292229398
H,0,1.9856655822,-3.8600975685,0.9515602625
O,0,-0.949282763,-0.4865379749,0.4480198945
H,0,-1.0069303924,0.3757717671,1.1566826358
C,0,4.2192501257,-2.6232352793,0.2391325639

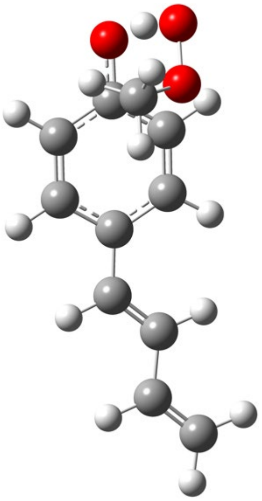

H,0,4.2663552707,-3.6688634075,0.5433247306
C,0,5.3667441351,-2.0268313672,-0.1541594862
H,0,5.3748430104,-0.9861761597,-0.4754473038
C,0,6.6432258273,-2.7200901641,-0.1791776188
H,0,6.6432492547,-3.7622657397,0.1386363279
C,0,7.7885830954,-2.1397248194,-0.5672656108
H,0,7.8131367955,-1.1009020486,-0.8884826767
H,0,8.7260576243,-2.6864510686,-0.5739366149

13-TS
Charge = 0 Multiplicity = 2
O,0,-2.2084492577,0.4183311564,0.6376122481
O,0,-1.0823043153,1.021119357,0.1675565287
C,0,-0.876916868,2.2485553917,0.8660545778
H,0,-0.8529546187,2.0641760877,1.9440245286
H,0,0.0845985231,2.6301334965,0.5174225211
H,0,-1.6821085129,2.9474574696,0.6214071615
C,0,2.4729770037,-0.0992712063,2.2652119714
C,0,2.9176859731,-0.6024476024,1.0199759649
C,0,1.9685106644,-1.2155335579,0.1707570337
C,0,0.646158672,-1.3205607337,0.5439930042
C,0,0.2067938633,-0.8117664163,1.7956314078
C,0,1.1502832037,-0.1870933663,2.6466026097
H,0,3.1954446297,0.3696354113,2.9283847763
H,0,2.2759898433,-1.6165263254,-0.789376448
H,0,-0.0834042101,-1.7914083231,-0.1082789812
H,0,0.8081273626,0.2024144437,3.6006055752
C,0,4.3273477081,-0.4625499734,0.6806419802
H,0,4.939994269,-0.0122360666,1.4594724316
C,0,4.9003460434,-0.8272971396,-0.4857061395
H,0,4.2810162471,-1.2543046465,-1.2727681656
C,0,6.3193152966,-0.6980301432,-0.8314741239
C,0,7.2990710432,-0.2619548256,0.0782220314
C,0,6.7219183102,-1.0293352462,-2.1353739234
C,0,8.6287820014,-0.1540338908,-0.3114794374
H,0,7.0253686901,-0.011423581,1.0984275953
C,0,8.0543730196,-0.9210194105,-2.5255084019
H,0,5.976278246,-1.3738500566,-2.8476180013
C,0,9.0133786381,-0.48097251,-1.6147196401
H,0,9.3718478964,0.1837232692,0.4046944253
H,0,8.3425929558,-1.1807425499,-3.5396085596
H,0,10.0536463383,-0.3959702546,-1.9136307825
O,0,-1.0449864375,-0.9428365287,2.1858861703
H,0,-1.7149190219,-0.4242371787,1.4581619214

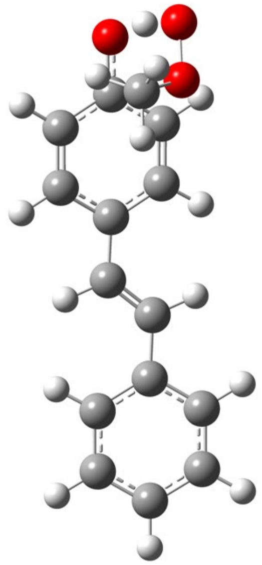

14(1)-TS
Charge = 0 Multiplicity = 2
O,0,0.2040938936,-0.8193986164,-1.4088671973
O,0,0.0115941547,0.5100498029,-1.6095371851
C,0,-1.1203257463,0.7144565802,-2.4624320372
H,0,-1.3062690125,1.7899734755,-2.4483064768
H,0,-0.8710332638,0.3789920476,-3.472725871
H,0,-1.9821729099,0.1632046373,-2.0793690993
C,0,-2.424777487,0.8797230376,0.716658465
C,0,-1.0824030737,0.4716393006,0.8486449835

C,0,-0.082730844,1.4527156324,1.0821715582
C,0,-0.4148471617,2.8141600492,1.1649823995
C,0,-1.7355668316,3.196337453,1.0297167823
C,0,-2.737364304,2.2234512602,0.8111494701
H,0,-3.1949134049,0.1355544352,0.5435877612
H,0,-1.9917862492,4.2475270646,1.09752645
O,0,-0.6968334932,-0.7886940993,0.7752859093
H,0,-0.2490688573,-0.9647420002,-0.2690870707
O,0,1.2042283806,1.1146607068,1.2222691476
H,0,1.2879587769,0.1460402638,1.1760465843
O,0,0.5486108424,3.7428611106,1.3756191741
H,0,1.4133650783,3.3099063223,1.4358340584
C,0,-4.1703017407,2.6188236564,0.6660505953
O,0,-5.0709270076,1.8322980324,0.4632043867
O,0,-4.3519486049,3.9364477054,0.7813681814
C,0,-5.706713539,4.4106242512,0.6566477543
H,0,-6.3193268185,3.9280339108,1.4253426299
H,0,-6.0923204534,4.1162400636,-0.3251322667
C,0,-5.6862512112,5.9158047012,0.8210785918
H,0,-5.0287721681,6.3499880078,0.0597641043
H,0,-5.2608341503,6.1626824019,1.8000208702
C,0,-7.0934587039,6.4940123968,0.695403214
H,0,-7.7604592443,6.0793270741,1.4584383648
H,0,-7.0797743502,7.5801869062,0.8173685944
H,0,-7.5256600553,6.2721240683,-0.2859701864

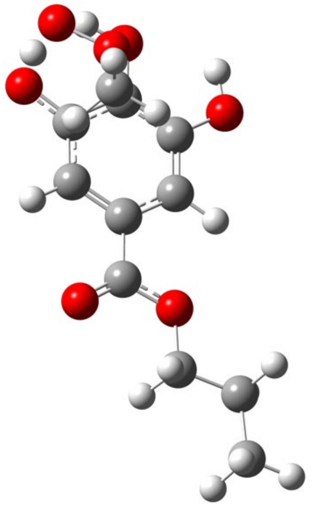

14(2)-TS

Charge = 0 Multiplicity = 2

O,0,0.4550520382,-1.3588505631,-0.8500841103
O,0,-0.8299123378,-1.388097708,-1.2710329948
C,0,-0.995159196,-0.5332643325,-2.4089769105
H,0,-0.6360182512,0.4732505584,-2.1772756765
H,0,-0.4375033933,-0.9516296531,-3.250703611
H,0,-2.0662621897,-0.5298243661,-2.6168599854
C,0,-3.6894048839,-0.6083328269,1.044306249
C,0,-2.3423598741,-0.8396846914,1.2863668099
C,0,-1.367440229,0.0999968111,0.8626861057
C,0,-1.7828544455,1.2894226531,0.2115373747
C,0,-3.1245086245,1.522540071,-0.0345175964
C,0,-4.0601885721,0.5677540924,0.3844771997
H,0,-4.4286257483,-1.3329095584,1.3631947275
H,0,-3.446757441,2.426128226,-0.5390321487
O,0,-1.9422165977,-1.9593151913,1.9080278854
H,0,-0.9791948284,-1.9240230665,2.0328228464
O,0,-0.0800643407,-0.1117322397,1.0883581176
H,0,0.3283416361,-0.7405241153,0.2362790617
O,0,-0.8410994283,2.1623434002,-0.1918638056
H,0,0.0192018437,1.8672812021,0.152826339
C,0,-5.5001414018,0.8614426569,0.0953017237
O,0,-5.8785222681,1.8617243119,-0.476199186
O,0,-6.3191145632,-0.0955053794,0.5323717224
C,0,-7.7256327683,0.1098222407,0.2940222256
H,0,-7.8858732728,0.2208074318,-0.7835798967

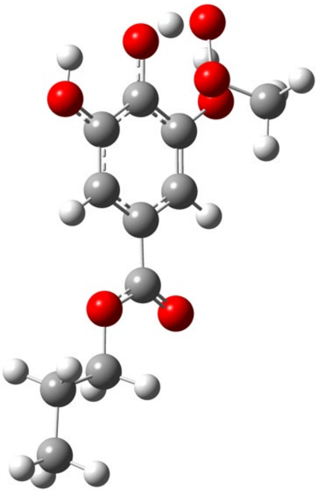

H,0,-8.0311522748,1.0398725954,0.7848559903
C,0,-8.4695859165,-1.0862686608,0.8493144184
H,0,-8.2492127389,-1.1804545726,1.918286435
H,0,-8.1037312373,-1.9946525225,0.3581733199
C,0,-9.9731008953,-0.9363452735,0.6306142774
H,0,-10.2109476939,-0.8555395512,-0.4352354516
H,0,-10.5120010417,-1.7996381075,1.0294545834
H,0,-10.3573863239,-0.0408764513,1.1301632109

14(3)-TS
Charge = 0 Multiplicity = 2
O,0,0.2193782947,-0.812235275,-1.4195390319
O,0,0.0268849968,0.5194168082,-1.6045818077
C,0,-1.1038915783,0.735258427,-2.4558208934
H,0,-1.2921754022,1.8100676351,-2.4248501776
H,0,-0.8521646848,0.4169515337,-3.4710623215
H,0,-1.9649107939,0.1758553243,-2.0829731652
C,0,-2.4291919426,0.8064793477,0.7218302267
C,0,-1.070961249,0.4509073816,0.8516483176
C,0,-0.1069383262,1.4685802544,1.0751587634
C,0,-0.4870907733,2.8187639931,1.1489531914
C,0,-1.8208355243,3.1488715358,1.0141760968
C,0,-2.7878996712,2.1399111145,0.8068656118
H,0,-3.1622696689,0.0248791018,0.558311794
H,0,-2.1278203885,4.1878465041,1.071890419
O,0,-0.639695859,-0.7952377339,0.7822859609
H,0,-0.2056880679,-0.9634398119,-0.2677089423
O,0,1.191089111,1.1772381447,1.2149870328
H,0,1.308590874,0.2118094336,1.1744797282
O,0,0.44326283,3.782052849,1.3507346835
H,0,1.3232317808,3.3806560677,1.4097381266
C,0,-4.2094792821,2.5777854825,0.6772282093
O,0,-4.5667751062,3.7345746321,0.7532498797
O,0,-5.0453624339,1.5591124981,0.4647629053
C,0,-6.4402050551,1.8952710428,0.3317509995
H,0,-6.5553440469,2.5917457594,-0.5054967899
H,0,-6.7677737178,2.4006683449,1.2463292776
C,0,-7.2064401469,0.6101654789,0.0987421535
H,0,-7.0285824254,-0.0699037958,0.9390141339
H,0,-6.8215900234,0.1226489449,-0.803839438
C,0,-8.7001885793,0.889390603,-0.0479186789
H,0,-8.8961022589,1.5571592559,-0.8934325197
H,0,-9.2542278516,-0.0374434172,-0.2176877007
H,0,-9.1039776898,1.361214935,0.8539576449

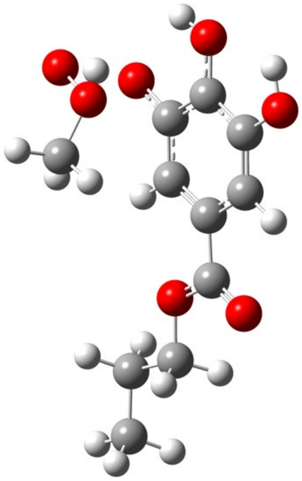

15(1)-TS
Charge = 0 Multiplicity = 2
O,0,-2.7978542556,0.2350450033,-0.16179237
O,0,-1.7558784972,0.2826938602,-1.0279461059
C,0,-1.3176461845,1.6337073246,-1.1950968234
H,0,-0.382454156,1.5727448457,-1.7551983452
H,0,-2.0716172803,2.1865461371,-1.7626026629
H,0,-1.1587532891,2.0996595281,-0.2198682179
C,0,1.385156587,-1.5176517308,-0.5132172155
C,0,0.1317207389,-1.5621190581,0.0995459999

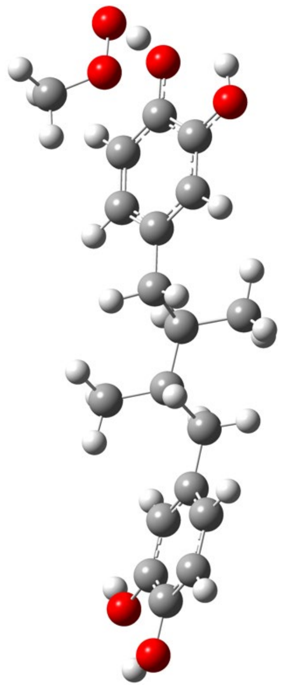

C,0,-0.2523874667,-0.5609118244,1.0341488583
C,0,0.6608829072,0.4673019791,1.3412116466
C,0,1.8998683468,0.4966056611,0.7344874579
C,0,2.2768259479,-0.4957696466,-0.204525763
H,0,1.6499594223,-2.294365001,-1.2250815466
H,0,0.3581408574,1.2261324035,2.0568771666
H,0,2.6018782554,1.2918900929,0.9724252806
O,0,-1.4539044812,-0.6502977846,1.5952130908
H,0,-2.2012422579,-0.2405467038,0.8930752671
O,0,-0.7182898591,-2.5560067911,-0.190122931
H,0,-1.5217228782,-2.4376222967,0.3447284617
C,0,3.6477637991,-0.4444228088,-0.8265258865
H,0,3.6912954715,-1.1330787579,-1.6791827939
H,0,3.8103824173,0.5652747393,-1.2172531226
C,0,4.779874098,-0.8169202292,0.1596972028
H,0,4.6539302128,-0.2101852446,1.0702417812
C,0,6.1571818556,-0.4576999366,-0.4467254946
H,0,6.1745302573,-0.8379786606,-1.4806610156
C,0,7.3167322967,-1.1353120112,0.3142938982
H,0,7.1886659959,-2.2214042746,0.2795294668
H,0,7.2660637976,-0.8383989389,1.3708854177
C,0,8.6817162609,-0.7989213246,-0.2389825133
C,0,9.1121380658,-1.3456555797,-1.4508618966
C,0,9.534977454,0.0842712791,0.4322680953
C,0,10.3591266838,-1.0217346304,-1.9870047352
H,0,8.4679858593,-2.039942113,-1.9848128308
C,0,10.77961241,0.40484008,-0.0955909112
H,0,9.2278902774,0.5294200689,1.3771818944
C,0,11.1991475984,-0.1447152832,-1.3125346735
H,0,10.6958726359,-1.4490128805,-2.9266252093
O,0,11.6752893615,1.2500342852,0.5009267709
H,0,11.3225552029,1.5891200076,1.3345748615
O,0,12.418708737,0.1700018807,-1.8290020921
H,0,12.8630254997,0.7859086952,-1.2280859
C,0,4.6472522135,-2.2917297383,0.5464533449
H,0,5.3205871052,-2.5659059987,1.362214498
H,0,3.6283494195,-2.5146288501,0.877986592
H,0,4.8691188811,-2.9394745718,-0.3116566176
C,0,6.3702032945,1.0583635246,-0.4774216294
H,0,7.2899539946,1.3193025736,-1.0087103832
H,0,6.4525746003,1.4485651051,0.5454864795
H,0,5.5490665156,1.5855814651,-0.970150766

15(2)-TS

Charge = 0 Multiplicity = 2

O,0,-2.9939956998,-1.4428511658,-1.6779868452
O,0,-2.4925619566,-0.2777544221,-2.1592241032
C,0,-1.4169446914,-0.5443733794,-3.0629358119
H,0,-0.6966820669,-1.2215972548,-2.5977602025
H,0,-1.8197611605,-0.9870523244,-3.9782789514
H,0,-0.9606841929,0.4251340689,-3.2725323471
C,0,0.1872343768,-0.1607395963,-0.0582824454
C,0,-1.1977322879,-0.0960326492,0.1918117756
C,0,-1.8452615056,1.1706283307,0.2035674031
C,0,-1.1128001447,2.3337743899,-0.0540405677

C,0,0.2452292077,2.2337320507,-0.3025046188
C,0,0.9211026443,0.9872545891,-0.3017262994
H,0,0.6601264279,-1.1398944131,-0.0541523028
H,0,-1.622596379,3.2916078694,-0.05650931
H,0,0.8146274061,3.137942445,-0.5061801525
O,0,-3.157292184,1.245048078,0.4593399198
H,0,-3.4883345096,0.3466460931,0.6301085162
O,0,-1.9319186696,-1.1754658186,0.4377104797
H,0,-2.4760635304,-1.4474797455,-0.486842051
C,0,2.4087200123,0.947481121,-0.5444687003
H,0,2.7162285622,-0.083265015,-0.7613873368
H,0,2.6291985645,1.5422223862,-1.4367640817
C,0,3.2425420457,1.4613846461,0.6517787756
H,0,2.8207841823,2.4270149346,0.9736638542
C,0,4.7036306609,1.7208048963,0.2147278056
H,0,5.0371476425,0.8449959858,-0.3648210169
C,0,5.6537413004,1.8641331036,1.4230564328
H,0,5.6263960201,0.9459518052,2.0177222476
H,0,5.2870622504,2.6760962197,2.065973046
C,0,7.0864510786,2.1390391413,1.0303001071
C,0,7.8733108236,1.1329729117,0.4630708756
C,0,7.647202163,3.4113197224,1.1901722369
C,0,9.1845598099,1.3841738782,0.0574158623
H,0,7.4598469129,0.1353723275,0.3370909454
C,0,8.9533624858,3.664680507,0.7895367631
H,0,7.060679598,4.2159149365,1.6306659905
C,0,9.7305822611,2.6515828982,0.2165242793
H,0,9.7978253129,0.6024109133,-0.3803377324
O,0,9.5762949418,4.8769174478,0.9129201537
H,0,8.9849327659,5.5251585283,1.3184939462
O,0,11.0099071458,2.9005243526,-0.1763821871
H,0,11.2212227026,3.82592911,0.0152646403
C,0,3.1165327454,0.4739309228,1.8139881694
H,0,3.5466967142,0.8662887592,2.7388227906
H,0,2.0647070428,0.2474577925,2.0151250781
H,0,3.6225418334,-0.4707920653,1.5761991934
C,0,4.8007268521,2.9636071399,-0.6748469767
H,0,5.809127963,3.0849562968,-1.0806240025
H,0,4.5640450754,3.8636507096,-0.0923333812
H,0,4.1112375764,2.9244731908,-1.5223823033

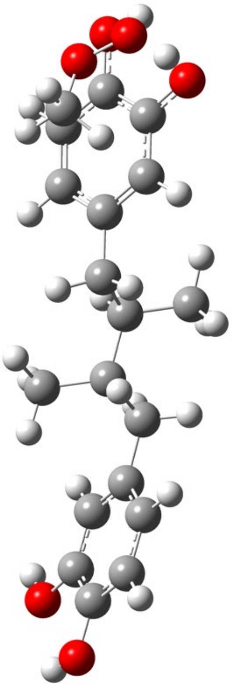

15(3)-TS
Charge = 0 Multiplicity = 2
O,0,1.7613418908,-1.2124791725,1.0061361637
O,0,1.220781206,-2.4038222423,0.6467206254
C,0,0.3569256418,-2.8818552602,1.680862601
H,0,-0.1620488753,-3.7434178666,1.2561423764
H,0,0.9607311074,-3.1818100894,2.5419750735
H,0,-0.3524618598,-2.1017859498,1.9666869843
C,0,-9.8888307526,-2.9128125903,-1.2832198371
C,0,-11.1839633723,-2.7671107463,-0.8006940383
C,0,-11.6196638484,-1.5364975765,-0.2964230073
C,0,-10.7445211031,-0.4574402259,-0.2862737875
C,0,-9.4455785514,-0.6075544468,-0.7723369111
C,0,-8.9985524338,-1.8326750696,-1.275294578

H,0,-9.5698113609,-3.8794763302,-1.6697075544
H,0,-11.094340296,0.4947730469,0.1005918797
H,0,-8.77186241,0.2459811573,-0.7633581408
O,0,-12.8880546603,-1.3925241092,0.1759428703
H,0,-13.3535574681,-2.236793414,0.0847294434
O,0,-12.1149722418,-3.7700899591,-0.7739692246
H,0,-11.7499591217,-4.5850438398,-1.1440962352
C,0,-7.5801739153,-1.9869155459,-1.7721916975
H,0,-7.4927082569,-2.9181718072,-2.3473676201
H,0,-7.3628863577,-1.1667994451,-2.463429978
C,0,-6.5255165172,-2.0166966699,-0.6436678699
H,0,-6.6995749539,-1.1456161107,0.0074901199
C,0,-5.1019349007,-1.8737488718,-1.232167981
H,0,-5.0220912952,-2.5723453215,-2.0807968318
C,0,-4.0138407677,-2.2600472594,-0.2063378756
H,0,-4.1551701509,-3.3013659875,0.1007986349
H,0,-4.1337216555,-1.6361956121,0.6892038086
C,0,-2.6108372177,-2.1083857726,-0.7382840537
C,0,-2.1324340406,-3.0105027487,-1.7219567057
C,0,-1.7740027192,-1.0916245191,-0.3127208026
C,0,-0.8696812556,-2.8911467311,-2.2766867684
H,0,-2.7798196474,-3.8201553047,-2.0506536822
C,0,-0.4793010071,-0.9471280037,-0.8483932494
H,0,-2.0946937421,-0.3787628063,0.4429313483
C,0,-0.0345751172,-1.8531239858,-1.8511137225
H,0,-0.5119010439,-3.5845037874,-3.0308657292
O,0,0.3505499828,0.0177076117,-0.4673615311
H,0,1.0666391048,-0.4058925563,0.2635070275
O,0,1.1853945533,-1.7091263818,-2.3831747781
H,0,1.6048056807,-0.9299344008,-1.9793052692
C,0,-6.7153252874,-3.2832471105,0.193912684
H,0,-6.1069109632,-3.2760193279,1.1018769117
H,0,-7.7600183443,-3.3834874836,0.5042569816
H,0,-6.4507628867,-4.176589963,-0.3874768165
C,0,-4.8524023074,-0.4509908025,-1.7411775203
H,0,-3.8972705137,-0.3764374956,-2.2691808353
H,0,-4.8226219574,0.2515414542,-0.8983487103
H,0,-5.63159116,-0.113707659,-2.4292763016

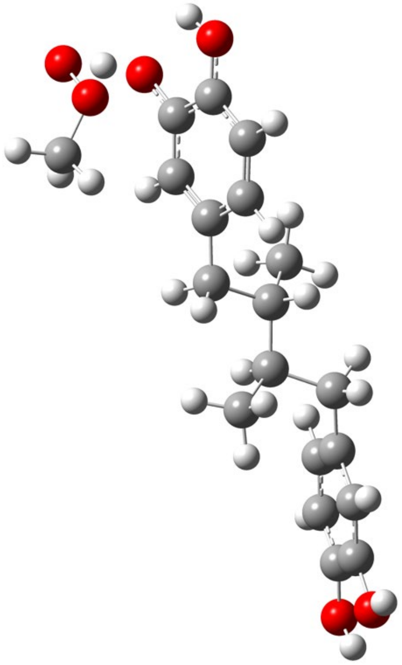

15(4)-TS
Charge = 0 Multiplicity = 2
O,0,0.9718012962,-0.7527768676,1.2818198285
O,0,-0.1717796433,-0.8441525421,2.0044442599
C,0,-0.5247525462,-2.2171326585,2.1929998256
H,0,0.1829678154,-2.6759527108,2.8892131258
H,0,-1.5305686008,-2.207403375,2.6175084564
H,0,-0.5111604231,-2.7422105353,1.2352261416
C,0,-11.2709265578,-1.7156971135,-0.4454055919
C,0,-12.6081668753,-1.5467607514,-0.1062247485
C,0,-13.1165664006,-0.2716848891,0.1661242798
C,0,-12.2707423421,0.8279651356,0.0913241523
C,0,-10.9294600867,0.6545295492,-0.2503693992
C,0,-10.4101814847,-0.6145435186,-0.5219448731
H,0,-10.8956782805,-2.7167879591,-0.6526337273
H,0,-12.6761633577,1.813696476,0.2981309363

H,0,-10.2785613813,1.5236151085,-0.3099280468
O,0,-14.426472992,-0.1059793584,0.4967943423
H,0,-14.8609025819,-0.9714928018,0.4964212911
O,0,-13.5153570495,-2.5671032829,-0.0104458934
H,0,-13.1000108892,-3.4147123276,-0.2188082051
C,0,-8.9489945747,-0.7871709485,-0.8649532948
H,0,-8.7860303835,-1.789389461,-1.2827774372
H,0,-8.6893692372,-0.0714921651,-1.6511161078
C,0,-8.0027860823,-0.6081972498,0.3431908253
H,0,-8.2717664969,0.3316913347,0.8501628363
C,0,-6.5386459277,-0.4687844106,-0.1355412838
H,0,-6.3517464991,-1.2665544327,-0.8720614734
C,0,-5.5356962178,-0.6603116809,1.0246333164
H,0,-5.6586993972,-1.6614432544,1.4503452736
H,0,-5.7650384039,0.0646644951,1.8161038279
C,0,-4.1003614542,-0.5023942606,0.5958059354
C,0,-3.5056918697,-1.4918865546,-0.2261558625
C,0,-3.359220671,0.6148506817,0.9655128954
C,0,-2.2019451212,-1.3657631086,-0.6603325762
H,0,-4.0903109092,-2.3623081031,-0.5125694221
C,0,-2.0413977228,0.7565053955,0.5273633141
H,0,-3.7918462796,1.391061595,1.5901553674
C,0,-1.4393423493,-0.240858648,-0.2891420489
H,0,-1.7330054575,-2.1202143664,-1.2852471231
O,0,-1.3341072227,1.8399802976,0.8736207974
H,0,-0.4567038757,1.7773963784,0.458994206
O,0,-0.1832256241,-0.0584245641,-0.6833384362
H,0,0.4904620965,-0.3723870467,0.133108324
C,0,-8.2173644757,-1.7595282127,1.3279433932
H,0,-7.6980259238,-1.598187798,2.2761142738
H,0,-9.2817074135,-1.8733938998,1.5561516779
H,0,-7.8626334348,-2.7069549749,0.9007593522
C,0,-6.3012216319,0.8877173882,-0.8060596576
H,0,-5.3081515529,0.9407525013,-1.2620663844
H,0,-6.3704557267,1.6927910462,-0.0633281465
H,0,-7.0317533088,1.0955117488,-1.591964336

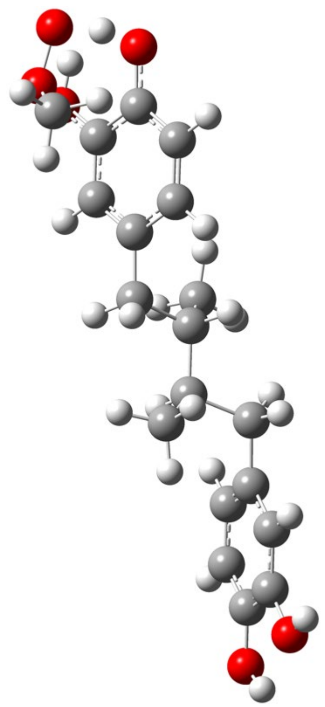

16(1)-TS

Charge = 0 Multiplicity = 2

O,0,-0.7770298856,-0.0885830921,3.9235977267
O,0,-2.1345204891,-0.1956630102,3.8555794265
C,0,-2.7294959856,1.0758506459,4.1157190945
H,0,-3.8015430034,0.9336426396,3.9652745725
H,0,-2.5221290664,1.3698155009,5.148689056
H,0,-2.3386114266,1.8250686584,3.4217606211
C,0,-3.84978902,-0.3465487055,0.6462635822
C,0,-4.0925999958,0.882207937,0.0275843312
C,0,-5.422549226,1.2026057297,-0.6015371996
H,0,-5.9492232075,1.9454067604,0.012306061
H,0,-5.2584196657,1.6728632312,-1.5760553107
C,0,-6.2636056162,-0.0652599449,-0.7362485028
H,0,-5.9044704638,-0.6740645834,-1.5736931694
H,0,-7.3116463923,0.1817089117,-0.9269479633
O,0,-4.8066144713,-1.3046474324,0.763895053
C,0,-2.589515585,-0.6649481718,1.1607374501

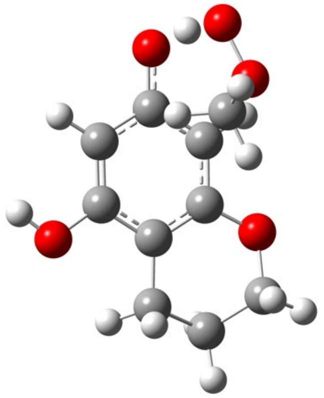

H,0,-2.4124799605,-1.6344581868,1.6117111742
C,0,-1.5430310332,0.2833279076,1.0873415632
C,0,-3.0231905683,1.815072171,-0.0308888441
C,0,-6.154832971,-0.8771811626,0.5402979731
H,0,-6.7430819338,-1.7950134468,0.5004014312
H,0,-6.4812462337,-0.2837815133,1.4061217987
C,0,-1.7736537565,1.5423303745,0.4853954994
H,0,-0.9661253897,2.2672051165,0.4454744105
O,0,-3.3229733782,2.994706956,-0.6314568179
H,0,-2.5402231586,3.5633226007,-0.6519868902
O,0,-0.3635070424,0.0075217278,1.6007408429
H,0,-0.4595026137,-0.1340991791,2.7419382103

16(2)-TS

Charge = 0 Multiplicity = 2

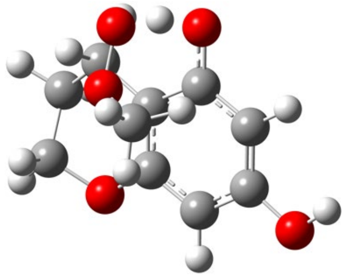

O,0,-0.212702997,0.7586273866,-1.0525839861
O,0,-0.3537964694,0.176620842,0.1726172722
C,0,0.9320805916,-0.0203592528,0.7613766987
H,0,1.3619462437,0.94905014,1.0302676017
H,0,0.7587193878,-0.625795728,1.6534455567
H,0,1.5874415975,-0.5432923737,0.0599332805
C,0,-2.0249050538,-3.0382984091,0.4318740295
C,0,-1.825849965,-2.1189787815,-0.6117857243
C,0,-2.9076969981,-1.168856848,-1.039425636
H,0,-2.6529798912,-0.1550526829,-0.7032691604
H,0,-2.9441684816,-1.1323139195,-2.1328534176
C,0,-4.2502308362,-1.5946342351,-0.4469087887
H,0,-4.6497016111,-2.4639000834,-0.9816245978
H,0,-4.9824953467,-0.786369473,-0.5261872823
O,0,-3.1795136945,-3.0891469622,1.1454507743
C,0,-1.0485531793,-3.9691755909,0.7695849515
H,0,-1.2279560576,-4.6797121698,1.569404897
C,0,0.1716331756,-3.992239079,0.0682875205
C,0,-0.5667467027,-2.1431077401,-1.2833310052
C,0,-4.0542486838,-1.9613641938,1.0116074108
H,0,-4.9849787457,-2.2652683138,1.4928326233
H,0,-3.6243041068,-1.1174382921,1.5687286749
C,0,0.4297984831,-3.0835507289,-0.9398771249
H,0,1.3715238407,-3.0706787796,-1.4805483066
O,0,-0.3364458912,-1.2797753495,-2.2502548077
H,0,-0.3256980101,-0.2287584389,-1.8069897076
O,0,1.0632654758,-4.9380780929,0.4570869173
H,0,1.864273666,-4.88031506,-0.0827108937

17(1)-TS

Charge = 0 Multiplicity = 2

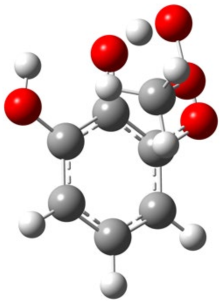

O,0,1.2057717907,-0.7531532424,0.7190508904
O,0,0.343303561,-0.6063864435,-0.3122388989
C,0,-0.7802987408,-1.4777628368,-0.1424608966
H,0,-0.4444705455,-2.5134128346,-0.239841823
H,0,-1.2362829218,-1.3167444972,0.8384076528
H,0,-1.4739345345,-1.2185929019,-0.9438666206
C,0,-2.4286772935,2.4135680764,-0.8761791475
C,0,-2.7431414439,1.7371707847,0.3078606805
C,0,-1.7116591878,1.3819083356,1.1637863007

C,0,-0.368041705,1.6881137444,0.8351847399
C,0,-0.085525722,2.3875185875,-0.3653513356
C,0,-1.1179406882,2.7467689156,-1.2230130802
H,0,-3.2346420845,2.6944437314,-1.5470069506
H,0,-3.7673114499,1.4923046286,0.5661595192
H,0,-0.8916600339,3.2753771749,-2.142127062
O,0,-1.9545971987,0.7091211304,2.3061030465
H,0,-1.120694049,0.6381200018,2.8013440941
O,0,1.1906248404,2.6822294639,-0.663279124
H,0,1.7586865161,2.3942571671,0.0703360814
O,0,0.6103805571,1.3413832909,1.6662750187
H,0,1.0230901037,0.3602698432,1.330329345

17(2)-TS

Charge = 0 Multiplicity = 2

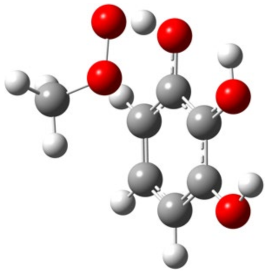

O,0,-1.832459149,-0.750362209,-1.1664686368
O,0,-0.6215307813,-0.2350410514,-1.5025714675
C,0,-0.7857886682,1.0586195942,-2.0911031138
H,0,-1.414952985,1.6828585808,-1.4526275958
H,0,0.2207012709,1.4739486439,-2.1714265133
H,0,-1.2373830503,0.9469195311,-3.0808284743
C,0,0.8303221999,2.4333296884,1.2024682448
C,0,-0.3838983099,1.7788039122,1.2545469178
C,0,-0.419166509,0.3747612685,1.1149377504
C,0,0.7975441356,-0.3327496499,0.9360295492
C,0,2.0232778569,0.3491206956,0.8735169254
C,0,2.0356002259,1.7263329342,1.0049768318
H,0,0.8693529889,3.5120075427,1.3129954049
H,0,-1.3194084302,2.3087880404,1.3994677253
H,0,2.9855224023,2.2487664519,0.9586857411
O,0,-1.5375990967,-0.3321725948,1.1481478497
H,0,-1.8246097932,-0.5886097519,0.0843266211
O,0,3.1778902405,-0.3374351408,0.687977194
H,0,2.9839548605,-1.2833432992,0.6082440508
O,0,0.8192990246,-1.6692667005,0.8165412094
H,0,-0.0882624431,-2.0094110266,0.8994711858

18(1)-TS

Charge = 0 Multiplicity = 2

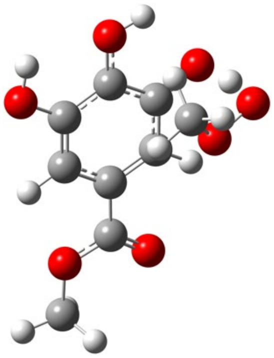

O,0,1.2583434059,-0.4022643723,-0.2655796876
O,0,0.8387126404,0.7898558079,0.2294283507
C,0,1.6464462234,1.8418207604,-0.3028237166
H,0,2.6758415011,1.7187014211,0.0438384113
H,0,1.6129380702,1.8250420914,-1.3967414379
H,0,1.2149943,2.7662871241,0.0847729965
C,0,-2.1993578412,0.3059869378,-0.3384282664
C,0,-1.280093196,0.4042159625,-1.4033650342
C,0,-1.1639708377,1.6304498554,-2.1061935396
C,0,-1.9350877798,2.741208506,-1.7508350121
C,0,-2.8313771488,2.6305004048,-0.7000906712
C,0,-2.961189785,1.4108636801,-0.001900897
H,0,-2.2927278504,-0.6277753024,0.2056784937
H,0,-3.4336031422,3.4899666378,-0.4275976571
O,0,-1.8084603532,3.913398804,-2.420095451
H,0,-1.1704006759,3.8107915498,-3.1419516108

O,0,-0.2946164062,1.7526861193,-3.1229810804
H,0,0.1026569505,0.8779211698,-3.2882254919
O,0,-0.5097990601,-0.5864626478,-1.8086567319
H,0,0.3741104761,-0.669145279,-1.0607923472
C,0,-3.9268020217,1.2718737354,1.1273925158
O,0,-4.0900085015,0.2497384628,1.7579704313
O,0,-4.599192212,2.40123572,1.3708919355
C,0,-5.5502808125,2.3395091589,2.4416070252
H,0,-5.9994078112,3.3299442233,2.4919207424
H,0,-6.3105228546,1.5850841761,2.2290658137
H,0,-5.0472975273,2.0995838128,3.3806648068

18(2)-TS

Charge = 0 Multiplicity = 2

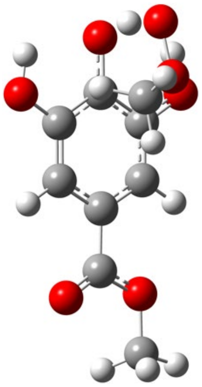

O,0,0.8990793776,0.2130118434,0.6110479791
O,0,-0.0509948057,0.4766944149,-0.3146585311
C,0,-0.105865818,-0.5820639689,-1.2785648187
H,0,-0.2488227901,-1.5415121524,-0.7737242318
H,0,-0.9554357976,-0.3463877873,-1.9213060996
H,0,0.8248545683,-0.5883065139,-1.8513645358
C,0,-3.5310487593,-1.1958939532,0.2274864167
C,0,-2.3759121891,-1.297950873,0.9827946629
C,0,-1.8036254833,-0.1461162126,1.5805643858
C,0,-2.4417891584,1.1112587857,1.4227832687
C,0,-3.6024474369,1.2173949594,0.6692110275
C,0,-4.1278768134,0.0628729843,0.0800894058
H,0,-3.9676391068,-2.071636676,-0.2387360146
H,0,-4.0799979359,2.181490952,0.545126064
O,0,-1.8982640988,2.1955600866,1.9964277363
H,0,-1.127010252,1.9224033137,2.5206608588
O,0,-0.6947537065,-0.2572674031,2.295257777
H,0,0.1853800685,-0.0246836908,1.6160819416
O,0,-1.7486777047,-2.478947034,1.1355660236
H,0,-1.0200625018,-2.3603322981,1.7689419941
C,0,-5.3731085652,0.1265385967,-0.7481426563
O,0,-5.8600787491,-0.8326429935,-1.3066821414
O,0,-5.8906598481,1.3550553083,-0.8075253144
C,0,-7.0864023551,1.4899007595,-1.5872986364
H,0,-7.8794442381,0.863318182,-1.1739809595
H,0,-7.359525513,2.5418073501,-1.5243151079
H,0,-6.8954530675,1.2052593703,-2.6240528941

18(3)-TS

Charge = 0 Multiplicity = 2

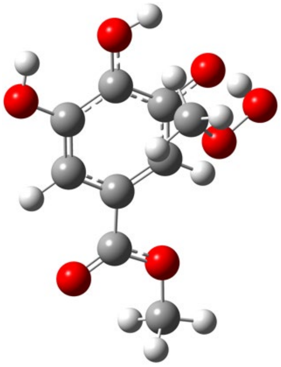

O,0,1.3274003016,-0.3868781905,-0.348584263
O,0,0.8603846288,0.7571566489,0.2129934786
C,0,1.6173598402,1.8719588854,-0.2637445882
H,0,2.653439809,1.7754618304,0.0711163557
H,0,1.5766613949,1.9150183032,-1.3565570522
H,0,1.1483624397,2.7531724362,0.1773223689
C,0,-2.1535202993,0.242318812,-0.3719487972
C,0,-1.2426352246,0.3879879108,-1.4402932609
C,0,-1.1522883796,1.6338760882,-2.1108227294
C,0,-1.9411473204,2.7214512783,-1.722893929
C,0,-2.8275550594,2.564173728,-0.6710101555

C,0,-2.9317711029,1.3260637973,-0.0035845715
H,0,-2.2170980569,-0.7106423131,0.1406314311
H,0,-3.4493684131,3.3978460386,-0.3615333125
O,0,-1.8388766819,3.9124823424,-2.3614161827
H,0,-1.2035053134,3.8402252676,-3.0892811352
O,0,-0.2875419311,1.7991595684,-3.1255366489
H,0,0.1235824431,0.9361165681,-3.3165787039
O,0,-0.4554128506,-0.5770647236,-1.8751812265
H,0,0.4398197884,-0.6553218552,-1.1427980642
C,0,-3.9107716413,1.2410649868,1.1189389215
O,0,-4.5996558833,2.169148806,1.4861094765
O,0,-3.9488029072,0.0295745633,1.6795917031
C,0,-4.865551119,-0.1219313467,2.771038154
H,0,-4.7606015383,-1.1543718307,3.1001736358
H,0,-4.6078266311,0.564966822,3.5798274001
H,0,-5.8871022425,0.0708596381,2.4368556154

19(1)-TS

Charge = 0 Multiplicity = 2

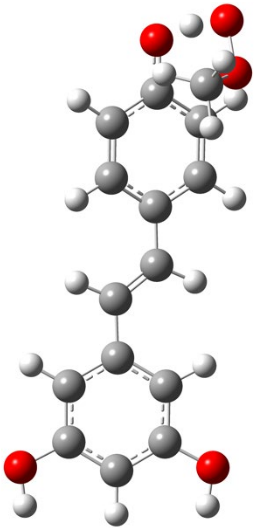

O,0,-1.563517683,-0.5297894925,2.5656816949
O,0,-0.3061443981,-0.0741073099,2.8188393725
C,0,0.3138459549,-0.9085009386,3.7971760407
H,0,1.346712666,-0.5619098095,3.868882244
H,0,-0.2004418909,-0.7911358816,4.7555951484
H,0,0.2769770388,-1.9528583824,3.4746915416
C,0,0.3159194517,-1.4505106383,0.4765941059
C,0,1.3413311207,-2.4214012839,0.6213375026
C,0,2.6568889748,-2.0878273444,0.3919113855
C,0,3.0217673149,-0.7686698974,0.0228302848
C,0,2.0008605785,0.1951251682,-0.1025414753
C,0,0.6747069498,-0.128444643,0.112211901
H,0,1.0574151953,-3.4278943714,0.9135902549
H,0,3.4213696342,-2.8486043425,0.5132839756
H,0,2.268188084,1.2120294516,-0.3765947526
H,0,-0.1091671343,0.6159473748,0.0114923157
O,0,-0.9409849895,-1.7944146412,0.6655021139
H,0,-1.3827883644,-1.1742467297,1.4910507726
C,0,4.3999575574,-0.36023494,-0.2201565896
H,0,4.5368533938,0.707100034,-0.3855296717
C,0,5.4713742692,-1.1773714477,-0.2745094927
H,0,5.337228856,-2.2515860795,-0.1617132503
C,0,6.8577065733,-0.7534476798,-0.5090099794
C,0,7.2475679818,0.5934364384,-0.4739995845
C,0,7.8103990534,-1.7461417677,-0.7710833926
C,0,8.5755910835,0.9279515281,-0.7138511767
H,0,6.5451547273,1.3877551386,-0.2483216466
C,0,9.1363450913,-1.3903299502,-1.0109153473
H,0,7.5299447551,-2.7942178511,-0.7973394752
C,0,9.5331739968,-0.0538278652,-0.9860186327
H,0,10.5703127985,0.2208311615,-1.166973751
O,0,8.9032190405,2.247366629,-0.6652594364
H,0,9.846589576,2.3659040818,-0.8404421692
O,0,10.0139066577,-2.3971961137,-1.2670318665
H,0,10.8995673451,-2.0399050547,-1.4182960546

19(2)-TS

Charge = 0 Multiplicity = 2

O,0,0.3082724775,-0.5721399122,0.2313088364
O,0,-0.9345589638,-0.3860939525,0.7615258303
C,0,-0.8588442308,0.5234409907,1.8612261261
H,0,-0.4324881869,1.4765730341,1.5357784936
H,0,-1.8880246174,0.6566094423,2.2004022248
H,0,-0.2462776339,0.0827862665,2.6526160855
C,0,-8.7223590929,-1.7817543832,0.5593677162
C,0,-8.6565425647,-0.4132197739,0.2764541594
C,0,-7.4320310141,0.1785874866,0.007307619
C,0,-6.2416011202,-0.5700088086,0.016975456
C,0,-6.3352663313,-1.9403706266,0.2948317232
C,0,-7.557422241,-2.5485730827,0.5661298809
H,0,-9.5733709325,0.1676156307,0.2649704613
H,0,-7.4077836944,1.2397530251,-0.2217911403
H,0,-5.4313019107,-2.5442450002,0.3008121911
H,0,-7.6046672247,-3.6134762633,0.7805919979
O,0,-9.9495400918,-2.3087378547,0.8136856153
H,0,-9.8739782346,-3.2566096871,0.9904956509
C,0,-4.9191221171,0.0088269977,-0.2513447468
H,0,-4.1127354184,-0.7207806575,-0.3217079659
C,0,-4.630355448,1.3138873902,-0.3904537936
H,0,-5.4125534659,2.0615414583,-0.2719270665
C,0,-3.2896317345,1.8552274175,-0.6594532582
C,0,-3.0562517483,3.2135355251,-0.4527596479
C,0,-2.2288780739,1.0533377451,-1.1167754766
C,0,-1.7793105386,3.7735722826,-0.6783229922
H,0,-3.8555486113,3.8672702396,-0.1157942562
C,0,-0.93818694,1.608166725,-1.3035006258
H,0,-2.3715208441,0.0080728798,-1.3636314808
C,0,-0.7190586936,2.9887492643,-1.0857481206
H,0,0.2754201013,3.397793709,-1.2385907588
O,0,-1.6675726323,5.105242615,-0.4529490672
H,0,-0.7641543496,5.4003670974,-0.6360411026
O,0,0.0629439684,0.8314239389,-1.6513315582
H,0,0.2352383141,0.0388693909,-0.8133083198

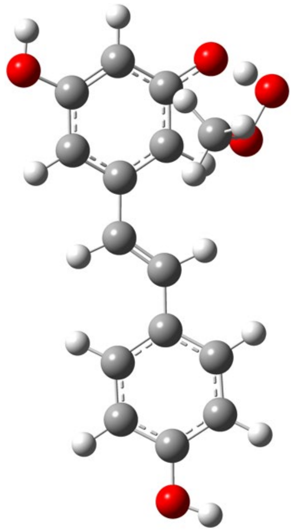

19(3)-TS

Charge = 0 Multiplicity = 2

O,0,0.2378719503,-0.6406684359,0.2260379713
O,0,-0.9178701253,-0.3130619429,0.8716297563
C,0,-0.6371289848,0.6272989289,1.9097689965
H,0,-0.1353111193,1.5066622524,1.4968316242
H,0,-1.6078592245,0.8983793723,2.3293186656
H,0,-0.0089299022,0.1524343881,2.6686766033
C,0,-9.7733572655,0.5471993282,0.4820205194
C,0,-8.694679063,-0.3437582218,0.4901264533
C,0,-7.4088840093,0.126119196,0.2715548563
C,0,-7.1604102552,1.4899766518,0.0349223135
C,0,-8.256944453,2.3625094234,0.0371089975
C,0,-9.5532922019,1.9056791856,0.2562609247
H,0,-8.8817550689,-1.3969526125,0.6742920354
H,0,-6.58686159,-0.583032326,0.2913138363
H,0,-8.0915203446,3.4222313154,-0.1402391644

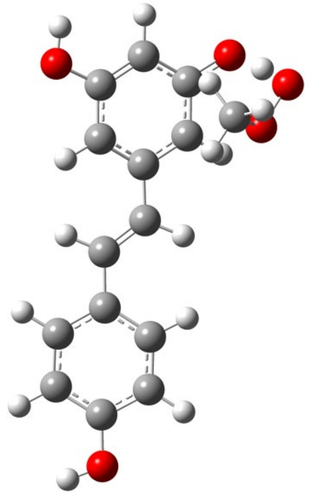

H,0,-10.3892089051,2.6008829199,0.2517682078
O,0,-11.0116257664,0.0319427462,0.7043950614
H,0,-11.6746770529,0.7353853086,0.6746364708
C,0,-5.8208952208,2.0390117091,-0.2100320636
H,0,-5.7875126518,3.1256368962,-0.284480003
C,0,-4.6849498031,1.3386232932,-0.3644993045
H,0,-4.7093662954,0.2507346672,-0.3399439139
C,0,-3.3525118913,1.9142384275,-0.6078595494
C,0,-3.0596452318,3.266718788,-0.4190459692
C,0,-2.3273919774,1.0615843602,-1.0438030298
C,0,-1.7694125237,3.7674736764,-0.6880255873
H,0,-3.8074467955,3.9652597402,-0.0581150088
C,0,-1.0220870162,1.5597711338,-1.2884375768
H,0,-2.5194678129,0.0077499084,-1.216437296
C,0,-0.7492743447,2.9349273374,-1.109652339
H,0,0.2561369352,3.3027123203,-1.2934714334
O,0,-1.5959487122,5.09760931,-0.4872256655
H,0,-0.6843008722,5.3500581367,-0.691472811
O,0,-0.0623851378,0.7434365545,-1.6607888755
H,0,0.1235466931,-0.0437511269,-0.8217176724

20(1)-TS

Charge = 0 Multiplicity = 2

O,0,-1.0573417352,1.806269347,-0.2488924615
O,0,-0.1969402517,1.3873320238,0.7133071525
C,0,-0.8616838753,1.3578809018,1.9762681861
H,0,-1.1735842615,2.3705994088,2.2451988467
H,0,-1.7311696693,0.6947739104,1.9291339736
H,0,-0.1255101642,0.9777249186,2.6867795338
C,0,-0.6911483173,-1.6990526559,0.6531440334
C,0,0.2906919355,-2.3900115688,1.3678049435
C,0,1.5890478044,-2.3999825584,0.8919191816
C,0,1.9556267682,-1.7248845487,-0.3033944015
C,0,0.9719856004,-1.0373064892,-1.0043482632
C,0,-0.3544618079,-1.003833459,-0.5406233121
H,0,0.0181841937,-2.9166597172,2.2765156847
H,0,2.3389547055,-2.9519389877,1.4488202094
H,0,1.2098748209,-0.5014361366,-1.9193586861
O,0,-1.3371547382,-0.3637953403,-1.1605270028
H,0,-1.262439639,0.719972069,-0.8834928157
O,0,-1.9605899676,-1.6737434743,1.0817729544
H,0,-2.4859726871,-1.2029732321,0.4087985201
C,0,3.327254061,-1.7283721155,-0.8253581742
H,0,3.4420375515,-1.2502563985,-1.7967209941
C,0,6.82256671,-2.5775720761,0.1178399558
C,0,8.1378637887,-2.5764622401,-0.3429806975
C,0,8.4327995488,-2.2446505944,-1.6645563258
C,0,7.3809021987,-1.9154369933,-2.524644709
C,0,6.0619601525,-1.9104667499,-2.0835971149
C,0,5.7781298737,-2.2365605305,-0.7499160528
H,0,6.6242347537,-2.8370409213,1.1529179132
H,0,9.4606323423,-2.2481346064,-2.0213779976
H,0,5.2786198046,-1.6657867782,-2.7922513279
C,0,4.4084315589,-2.2384892726,-0.2118319903
H,0,4.3118583538,-2.682042085,0.7774100416

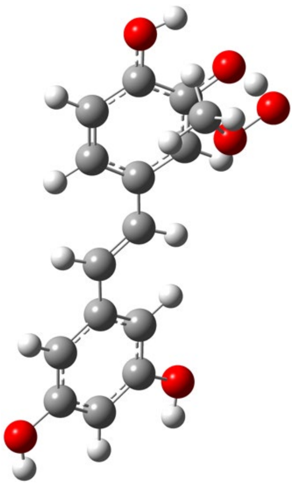

O,0,7.6023046885,-1.5938582701,-3.8283663639
H,0,8.5470456264,-1.638193427,-4.0284392647
O,0,9.1098467069,-2.9103624587,0.548497446
H,0,9.9783282754,-2.8750643136,0.1251059994

20(2)-TS

Charge = 0 Multiplicity = 2

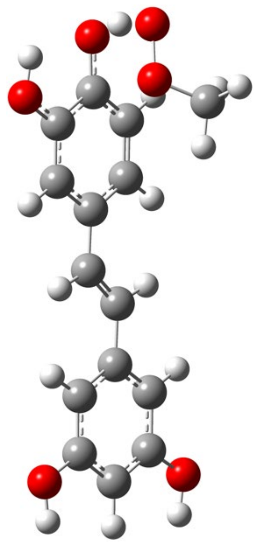

O,0,-2.5044971996,-0.3891413157,0.2006361032
O,0,-1.3516798632,-0.3768126718,-0.509496205
C,0,-0.6619169678,-1.6200222231,-0.3515291934
H,0,-1.2101066157,-2.4026260982,-0.8835206272
H,0,0.3235090225,-1.469606054,-0.7968004415
H,0,-0.5776939718,-1.8682085973,0.7089112328
C,0,-0.3433161028,1.1094846396,1.4893295102
C,0,0.662640298,0.3417453434,2.1104338029
C,0,1.9715746865,0.4319152462,1.6910324363
C,0,2.3342206647,1.2997408149,0.6264534821
C,0,1.343313918,2.072353778,0.0110120554
C,0,0.0207804794,1.9890759211,0.4335777634
H,0,0.3738049236,-0.3187768396,2.9225781828
H,0,2.7271971305,-0.1681786868,2.1857860429
H,0,1.59415064,2.7467606901,-0.8025358271
O,0,-0.9210156938,2.7402663794,-0.1557727892
H,0,-1.7692247029,2.5698825331,0.2875797212
O,0,-1.6157719074,1.0690200918,1.869926829
H,0,-2.165919689,0.401242146,1.1957401568
C,0,3.7088471824,1.429545456,0.1418038505
H,0,3.8345847041,2.1330131621,-0.6789921453
C,0,7.1417606664,0.1049735632,0.7405589582
C,0,8.4667172186,0.1886241649,0.315658281
C,0,8.8340403211,1.0576506153,-0.7102226918
C,0,7.8472831737,1.8461754304,-1.3097026504
C,0,6.5203553108,1.7763457025,-0.9009016176
C,0,6.1614168903,0.8991473973,0.1327792936
H,0,6.8852430732,-0.5793772746,1.5430462093
H,0,9.8692604175,1.1230116185,-1.0381026219
H,0,5.7935698325,2.4103276681,-1.3955693595
C,0,4.7804192211,0.7696147283,0.6194738892
H,0,4.6575608807,0.0651106219,1.4396502845
O,0,8.1438325667,2.7128078707,-2.3155526764
H,0,9.0883927013,2.6808962692,-2.5190126723
O,0,9.3730556065,-0.6085784095,0.9421805169
H,0,10.2557392641,-0.4666146014,0.5739684764

20(3)-TS

Charge = 0 Multiplicity = 2

O,0,-0.303686792,0.3561401889,-0.0419123058
O,0,-1.3951175053,-0.1747775032,0.5807711576
C,0,-1.0262232186,-0.6526094064,1.8759899476
H,0,-0.2239574584,-1.391146539,1.7923691321
H,0,-0.7042002946,0.1904431832,2.4934579532
H,0,-1.9292711155,-1.1080382712,2.2870799117
C,0,-9.0868992943,-2.1383625336,0.2478043232
C,0,-8.4575301819,-3.374630616,0.3510033321
C,0,-7.0782976698,-3.4741557095,0.1981315564

C,0,-6.2984225688,-2.3370674351,-0.0565141364
C,0,-6.9455551146,-1.0950000488,-0.1698656853
C,0,-8.319752714,-0.9966493298,-0.0170506422
H,0,-9.064289664,-4.2534958357,0.5451295891
H,0,-6.6134869985,-4.4520473883,0.2686987007
H,0,-6.3653275339,-0.1977398829,-0.3770930228
O,0,-9.0284467013,0.1687161061,-0.1053676663
H,0,-8.4427529598,0.9151151765,-0.2917857905
O,0,-10.4323046394,-2.0454675433,0.3956234168
H,0,-10.6984681341,-1.1201668865,0.2871121899
C,0,-4.8378843286,-2.377350001,-0.2090563553
H,0,-4.389364116,-1.4434226626,-0.5462510701
C,0,-1.8520320881,-4.4947715238,0.4395665218
C,0,-0.4446321681,-4.5407664311,0.3256016182
C,0,0.2513387405,-3.5342004922,-0.3133857811
C,0,-0.4730308983,-2.4606793513,-0.883266411
C,0,-1.8875199551,-2.4342000071,-0.8038809753
C,0,-2.5813514048,-3.4439985913,-0.1137435108
H,0,-2.3550992859,-5.3010562062,0.9655529995
H,0,1.3348179871,-3.5398806635,-0.3871680985
H,0,-2.4084654872,-1.6302301788,-1.3100255136
C,0,-4.0427423886,-3.4296427957,0.0472543303
H,0,-4.4721526093,-4.352646002,0.4321280195
O,0,0.167515263,-1.4704097184,-1.4623426439
H,0,-0.0506794904,-0.4856872657,-0.8779178164
O,0,0.1617078163,-5.6122940904,0.8917978487
H,0,1.1196318924,-5.5607857943,0.763215397

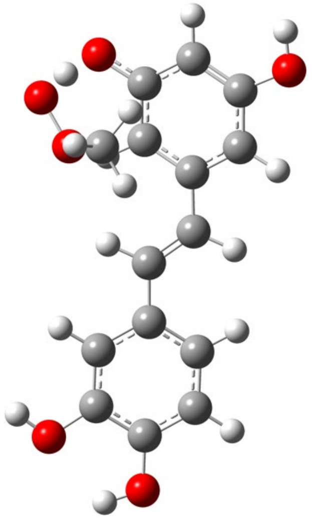

20(4)-TS

Charge = 0 Multiplicity = 2

O,0,0.2918603026,-0.7174789457,-0.2940434405
O,0,-0.8766012304,-0.5844344418,0.3959693436
C,0,-0.6272879377,0.0764533563,1.6377724219
H,0,-0.0064356061,-0.5640978803,2.2705263885
H,0,-0.1289807978,1.0336007426,1.4619653245
H,0,-1.6086910314,0.2292539402,2.0909287163
C,0,-9.7517192421,-0.1413768697,-0.2124407625
C,0,-8.6253052102,-0.9450220705,-0.3486570432
C,0,-7.3566732469,-0.3722759323,-0.3663411871
C,0,-7.1991545826,1.0164045625,-0.2542724382
C,0,-8.3456903951,1.8137122618,-0.1103210906
C,0,-9.6115573007,1.2466593027,-0.090185118
H,0,-8.7440820548,-2.0223263535,-0.4340907363
H,0,-6.4916125752,-1.0200344826,-0.4608694652
H,0,-8.2590193931,2.8927424984,-0.0160482828
O,0,-10.7061190699,2.0396659917,0.0474973146
H,0,-11.5001769635,1.4849620838,0.0384353486
O,0,-11.0362668925,-0.6037875556,-0.1792516147
H,0,-11.0582820783,-1.5662600529,-0.2695897576
C,0,-5.8887342602,1.6820750154,-0.2794192501
H,0,-5.9168258209,2.7494774366,-0.0629666197
C,0,-2.3191338311,1.1357721674,-1.1788852809
C,0,-1.0395825408,1.7463817275,-1.2194891826
C,0,-0.8563905033,3.0316820807,-0.6612600429
C,0,-1.9368220003,3.6638626179,-0.0739054917

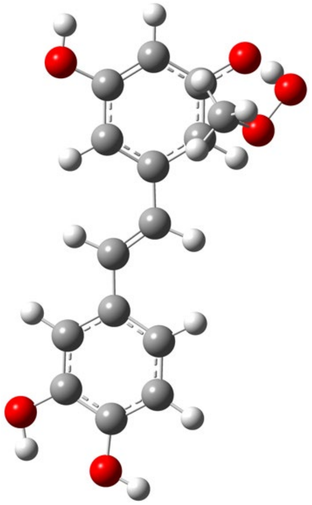

C,0,-3.2021971656,3.0454634182,-0.0113282979
C,0,-3.4065440916,1.784551546,-0.575873496
H,0,-2.440007907,0.1592262705,-1.6363568697
H,0,0.1289898649,3.4876184784,-0.6924170238
H,0,-4.0023908127,3.5780822531,0.4922065852
C,0,-4.7104578725,1.102403392,-0.5605375831
H,0,-4.6754485404,0.0481681912,-0.8288635949
O,0,-1.8486613341,4.8932133148,0.4915682146
H,0,-0.9486973236,5.2379263423,0.4033343878
O,0,-0.0215930683,1.1102191132,-1.7541582766
H,0,0.1808057632,0.1358651296,-1.1507275491
